# Pien-Tze-Huang prevents hepatocellular carcinoma by inducing ferroptosis via inhibiting SLC7A11-GSH-GPX4 axis

**DOI:** 10.1186/s12935-023-02946-2

**Published:** 2023-06-06

**Authors:** Xiangying Yan, Yudong Liu, Congchong Li, Xia Mao, Tengteng Xu, Zhixing Hu, Chu Zhang, Na Lin, Ya Lin, Yanqiong Zhang

**Affiliations:** 1 College of Pharmacy, Fujian University of Traditional Chinese Medicine, No. 1, Qiuyang Road, Shangjie Town, Minhou County, Fuzhou, 350122 China; 2 Institute of Chinese Materia Medica, China Academy of Chinese Medical Sciences, No. 16, Nanxiaojie, Dongzhimennei, Beijing, 100700 China

**Keywords:** Hepatocellular carcinoma, Pien-Tze-Huang, Ferroptosis, Solute carrier family 7 member 11, Glutathione, Glutathione peroxidase 4

## Abstract

**Background:**

Malignant transformation from hepatic fibrosis to carcinogenesis may be a therapeutic target for hepatocellular carcinoma (HCC). The aim of this study was to evaluate anti-cancer efficacy of Pien-Tze-Huang (PZH), and to investigate the underlying mechanisms by integrating transcriptional regulatory network analysis and experimental validation.

**Methods:**

A diethylnitrosamine (DEN)-induced HCC model in rats was established and used to evaluate the anti-cancer efficacy of PZH. After detecting a transcriptomic profiling, the “disease-related gene–drug effective target” interaction network was constructed, and the candidate targets of PZH against malignant transformation from hepatic fibrosis to HCC were identified and verified in vitro.

**Results:**

PZH effectively alleviated the pathological changes of hepatic fibrosis and cirrhosis, and inhibited tumor formation and growth in DEN-induced HCC rats. Additionally, the administration of PZH reduced the levels of various hepatic function-related serological indicators significantly. Mechanically, a ferroptosis-related SLC7A11-GSH-GPX4 axis might be one of potential targets of PZH against malignant transformation from hepatic fibrosis to HCC. Especially, high SLC7A11 expression may be associated with poor prognosis of HCC patients. Experimentally, the administration of PZH markedly increased the trivalent iron and ferrous ion, suppressed the expression levels of SLC7A11 and GPX4 proteins, and reduced the GSH/GSSG ratio in the liver tissues of DEN-induced HCC rats.

**Conclusions:**

Our data offer an evidence that PZH may effectively improve the hepatic fibrosis microenvironment and prevent the occurrence of HCC through promoting ferroptosis in tumor cells via inhibiting the SLC7A11-GSH-GPX4 axis, implying that PZH may be a potential candidate drug for prevention and treatment of HCC at an early stage.

**Supplementary Information:**

The online version contains supplementary material available at 10.1186/s12935-023-02946-2.

## Introduction

Liver cancer is the top 10 most prevalent cancer worldwide, with 905,677 people suffering from it in 2021, having caused 830,180 deaths and being the third leading cause of cancer deaths [1]. As one of the most important and common type of liver cancer, hepatocellular carcinoma (HCC) is often developed from hepatic fibrosis, 30% of patients with hepatic fibrosis will develop cirrhosis, and more than 80% of these patients may eventually develop HCC, posing a serious risk to the life and health of patients [2]. Liver resection, liver transplantation, ablation therapy, etc*.* are often carried out as the therapeutics for HCC at the early stage; in contrast, the targeted drug—sorafenib and interventional therapies such as radiotherapy and chemotherapy have become the main treatment methods for HCC patients at the late stage [3]. Considering the unfavorable clinical efficacy and various side effects of the exsiting therapeutics strategies, it is of great significance to develop novel drugs for controlling the aggressive progression from hepatic fibrosis to HCC at the early stage.

Drug repositioning is a research strategy to discover new indications for existing drugs, which has the advantages of low research and development cost, time-saving and labor-saving in the process of new drug development [4]. In recent years, traditional Chinese medicine (TCM) has shown unique advantages in tumor treatment [5]. Growing clinical evidence show that TCM combined with chemotherapy drugs may improve patients' survival quality, prolong survival period, reduce the side effects caused by chemotherapy and targeted drugs and alleviate patients’ pain [6]. Pien-Tze-Huang (PZH), a Chinese patent drug approved and marketed by CFDA, has been extensively used for the treatment of various inflammatory diseases and human cancers, such as colorectal cancer, osteosarcoma, etc. [7–9]*.* In particular, PZH has shown favorable efficacy in the treatment of viral hepatitis and HCC. The clinical efficiency of PZH reached 67.25–72.86% when used alone for the treatment of viral hepatitis [10–12] and increased to 59.6–85.3% when used in combination (herbal medicine, chemotherapy, radiotherapy) for the treatment of HCC [13, 14]. PZH treatment can effectively improve HCC patients' symptoms of little food, dry mouth and throat, weakness, fever, pancytopenia, hepatomegaly, jaundice, greasy coating and abdominal distension [12, 13]. However, whether PZH may interfere with the malignant transformation from hepatic fibrosis to HCC and its underlying pharmacological mechanisms have not been fully elucidated.

In the current study, we firstly evaluated the pharmacological efficacy of PZH against the malignant transformation from hepatic fibrosis to HCC based on a diethylnitrosamine (DEN)-induced HCC model in rats, and then performed the transcriptomics profiling related to the disease progression and drug efficacy using liver tissues collected from normal group, DEN model group and PZH administration group. After screening HCC-related genes and PZH effective genes, the “disease gene-drug target” interaction network was constructed and the core regulatory genes were screened by calculating the nodes’ topological features, which were further experimentally validated in vivo.

## Methods

### Animal experiments

Male Sprague–Dawley (SD) rats (n = 48, 200–220 g in weight) were purchased from Beijing Vital River Laboratory Animal Technology Co., Ltd. (Production License No. SCXK 2016–0011, Beijing, China) and housed at the Experimental Animal Center, Institute of Fundamental Research, Chinese Academy of Traditional Chinese Medicine, Beijing, China. All rats were housed under specific pathogens-free conditions, with a constant temperature of 24℃ ± 1℃ in a 12-h light/12-h dark cycle room and were fed with ad libitum water and food access. Prior to the experiments, the rats were allowed a 1-week acclimatization period.

A total of 48 SD rats were randomly divided into four groups: normal control (n = 12), DEN model (n = 12), DEN + PZH low-dose (n = 12), and DEN + PZH high-dose (n = 12) groups. Rats in the normal control group were fed a normal diet. DEN model, DEN + PZH low-dose and DEN + PZH high-dose groups were injected intraperitoneally with 0.01% DEN (catalog number N0756, Sigma Aldrich, St. Louis, MO, USA) weekly by the experimentalists for a period of 16 weeks. Besides, rats in the DEN + PZH low dose group were given with 0.01 g/kg/day PZH (Zhangzhou Pien-Tze-Huang Pharmaceutical Co., Ltd., Zhangzhou, China), while rats in the DEN + PZH high dose group were given with 0.04 g/kg/day PZH.

The body weight of all rats was recorded at least once a week during the experiment. The experiment was carried out for 16 weeks, and the rats were executed at the 11, 14 and 16 weeks, with three rats in each group. Before execution, the rats were fasted for 12 h and water was ingested normally. On the day of execution, the rats were weighed and injected intraperitoneally with 0.3% sodium pentobarbital (1 ml/100 g), and kept warm with an electric blanket during anesthesia. When the rats were deeply anesthetized, blood was collected from the abdominal aorta using a disposable blood collection needle. In addition, the liver and kidney were collected from each rat, weighed and recorded, and the ratio of liver (kidney) to body weight was calculated as the liver index (kidney index). The livers were also photographed and recorded. The blood of rats was centrifuged to obtain serum, which was quickly stored in -80℃ refrigerator together with livers and kidneys.

### Hepatic fibrosis and HCC, ferroptosis -related serum markers detection

Blood samples from rats were collected through the orbital venous system. Serum levels of alkaline phosphatase (ALP), alanine aminotransferase (ALT), aspartate aminotransferase (AST), total serum protein (TP), hyaluronic acid (HA), a marker of hepatic fibrosis, and tumor serum marker alpha fetoprotein (AFP), reduced glutathione (GSH) and oxidized glutathione (GSSG), were measured using enzyme linked immunosorbent assay (ELISA) kits (YJ003360, YJ059335, YJ059334, YJ601216, YJ358140, ml003120, LOT20220704A, Shanghai Enzyme Linked Biotechnology Co., Ltd.)

### Histopathological evaluation

After removing the liver, the liver was fixed in 10% formalin, and following routine procedures, the paraffin was cut into 3 μm sections. The slices were fished in a water bath at 43 °C, then moved to a 43 °C section extension table for spreading and placed in a 60 °C oven for 2 ~ 4 h and overnight at 37 °C. After dewaxing and rehydration, the sections were stained with hematoxylin and eosin, and finally sealed with neutral gum, observed under a light microscope and photographed for pathology.

### Gene expression profiling

Liver tissues from normal, DEN model and PZH administration groups were collected, and mRNA-Seq Illumina NovaSeq 6000 (Illumina, CA, USA) were performed to obtain the transcriptomic dataset of rat liver tissues. The data set consisted of transcriptomic data from liver tissues of three normal rats, three DEN model rats and three rats in the PZH administration group. Using |log Fold Change|> 2, *P* < 0.001 as the intergroup difference screening criteria, the intergroup difference genes between the DEN model group and the normal group was screened to obtain the HCC-related genes; the intergroup difference genes between the PZH administration group and the DEN model group was screened to obtain the PZH effective genes. The gene expression data have been uploaded and are publicly available in the NCBI GEO database (GEO No. GSE221790, https://www.ncbi.nlm.nih.gov/geo/query/acc.cgi?acc=GSE221790).

### Network construction analysis

A total of 1956 differentially expressed genes were identified by comparison of [MOD *vs.* CON] and [PZH *vs.* MOD], which were respectively indicated as HCC-related genes and PZH effective genes. Then, the “disease gene-drug target” interaction network was constructed using the links among the HCC-related genes and PZH effective genes, and was imported into Cytoscape (version:3.9.1). Cytohubbe, one of Plug-in of Cytoscape was used to calculate the three network topological features, including nodes’ degree, betweenness, and closeness, and the nodes whose topological feature values are greater than one times the median of all nodes was selected as the core nodes in the network for the next pathway enrichment analysis.

### Pathway enrichment analysis

Based on the database for Kyoto Encyclopedia of Genes and Genomes (KEGG, Encyclopedia of Genes, https://www.kegg.jp/) and the Database for Annotation, Visualization and Integrated Discovery (DAVID, Bioinformatics database, https://david.ncifcrf.gov/, version: 6.8), functional enrichment analysis was performed for the above core genes, and the correction method was chosen Bonferroni, with *P* values < 0.05 were used to select pathways.

### Evaluation on associations of SLC7A11 expression with HCC progression and patients’ prognosis

A HCC dataset [TCGA Provisional, Tumor Samples with mRNA data (RNASeqV2) (373 samples)] was collected from the TCGA database (https://cancergenome.nih.gov/), including solute carrier family 7 member 11 (SLC7A11) mRNA expression data in HCC clinical samples for statistical analysis of clinicopathological characteristics and prognostic relevance.

### Prussian blue iron stain

Iron staining was performed using the Prussian Blue Iron Stain kit (With Eosin) (G1424, Solarbio). The kit consisted of A1: Perls staining solution A and A2: Perls staining solution B. After routine dewaxing and rehydration of liver paraffin sections, A1 and A2 were mixed in equal amounts, and the sections were stained with Perls working solution dropwise for 25 min, washed with PBS for 3 min, washed twice, and then the background was lightly stained with eosin staining solution for 15 ~ 30 s, and finally rinsed with tap water for 3 s. Finally, the sections were dehydrated, transparent, and sealed. The nuclei as well as other tissues were stained red, while iron-containing heme or trivalent iron was stained blue. The staining results were quantitatively analyzed using ImageJ software.

### Fe^2+^ content testing

The Fe^2+^ content in rat liver tissues was detected using the Ferrous Content (Ferrous Zine Colorimetric Assay) Kit (ml518042, Shanghai Enzyme Linked Biotechnology Co., Ltd.). Liver tissues of rats were cut up and homogenized in an ice bath by adding a certain proportion of the extract. The extracts were centrifuged at 12,000 rpm for 5 min at 4 °C. The supernatant was removed and placed on ice for testing. The samples were incubated with the assay reagent for 15 min at room temperature and the absorbance was measured at 562 nm using a visible spectrophotometer.

### Immunohistochemistry staining

Immunohistochemical staining was performed using a rabbit/mouse two-step detection kit (Cat No. SV0002/SV0001, Boster Biological Technology Co., Ltd.) and DAB kit (Cat No. AR1027, Boster Biological Technology Co., Ltd.). The antibody was glutathione peroxidase 4 (GPX4, dilution 1:2000, 67763-1-Ig, Proteintech). The experimental method as well as the staining score were performed based on our previous studies [15].

### Western blot analysis

To evaluate the regulatory effects of PZH on the expression levels of SLC7A11 protein in the liver tissues of different groups, western blot analysis was performed according to the protocol in our previous study [15]. The detailed information on these antibodies is provided as follows: SLC7A11 (dilution 1:2000, host species: Rabbit, ab175186, Abcam), glyceraldehyde-3-xphosphate dehydrogenase (GAPDH, dilution 1:5000, host species: Mouse, 60004-1-IG, Proteintech), HRP-conjugated Affinipure Goat Anti- Mouse IgG(H + L) (dilution 1:5000, SA00001-1, Proteintech), Anti-Rabbit IgG, HRP-linked Antibody (dilution 1:5000, 7074S, Cell Signaling).

### Statistical analyses

All data were statistically analyzed using GraphPad Prism (version 9.1.2). Data are expressed as mean ± standard deviation (SD), and one-way ANOVA was used for the 3 groups, with two-way comparisons between the two groups that differed. *P* values < 0.05 were considered significant.

## Results

### PZH attenuates DEN-induced hepatic fibrosis and cirrhosis, and inhibits tumor formation and growth

**Fig. 1:**
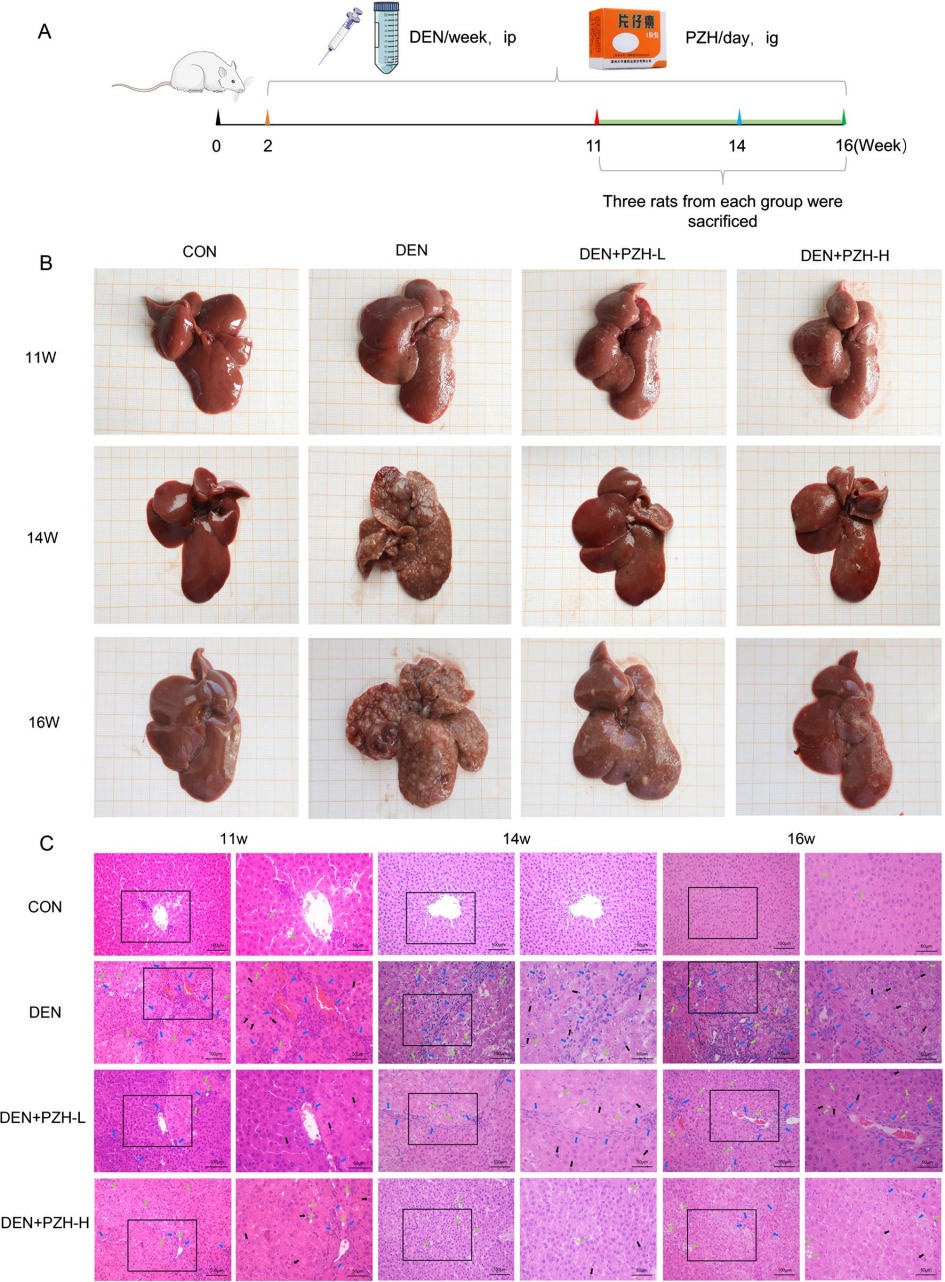
Animal experimental protocols and the pharmacological effects of PZH on tumor lesions, and pathological changes of the liver tissues obtained from different groups. **A** Animal experimental protocols. **B** Tumor lesions in the liver tissues of rats in different groups. **C** Pathological changes of the liver tissues of rats in different groups. blue arrows represent hepatic fibrosis, cirrhosis and inflammation; green arrows represent steatosis; black arrows represent enlarged nuclei, deep staining and white nodules

To verify whether PZH could play a role in reversing the malignant transformation from hepatic fibrosis to HCC, we evaluated the pharmacological effects of PZH based on a DEN-induced HCC model in rats (Fig. 1A). During the experiment, one rat in the PZH high-dose group died. As shown in Fig. 1B, DEN injury in rats led to progressive hepatic fibrosis and cirrhosis, followed by HCC. The liver surface of the control rats was smooth, red in color and soft in texture. After 11 weeks of modeling, DEN caused hepatic fibrosis in the rats, with fine particles as well as a small number of protrusions on the liver surface, advanced fibrosis and cirrhosis at 14 weeks, with a rough liver surface and uneven edges, and a large number of white bumps visible, and all rats had obvious tumor lesions at 16 weeks, with multiple white nodules on the liver surface with hemorrhage, a hard texture and an increased volume. The administration of PZH significantly inhibited fibrosis progression and tumor formation. Figure 1C displayed the histopathological changes in the liver tissues of different groups. Among them, the morphology of the normal liver tissues showed the intact hepatic lobule structure, hepatocyte cords arranged radially around the veins, and hepatocytes of uniform size. By 11 weeks of DEN injury, the liver tissues of the model rats showed fibrosis and sclerosis, with a large number of fibroblasts and fibroblasts (indicated by blue arrows in Fig. 1C) appearing, the central vein disappearing, hepatocyte cords becoming disorganized, and hepatocytes enlarging. At week 14, the liver tissues of DEN rats showed pseudo lobules, a large number of vacuoles and deformed fatty oil droplets (indicated by green arrows in Fig. 1C). HCC had formed by week 16, and the liver of DEN rats showed deep stained nuclei, increased karyoplasmicratio (indicated by black arrows in Fig. 1C), inconsistent hepatocyte size and disorganized arrangement, along with inflammatory cell infiltration. Under PZH treatment, hepatic fibrosis was reduced in DEN rats, hepatocyte morphology improved, and with increasing doses, the structure of liver lobules became intact, and vacuolation and steatosis were greatly reduced (Fig. 1C).

### PZH attenuates DEN-induced hepatic and renal pathological damage, improves hepatic function, and inhibits fibrosis and tumorigenesis

**Fig. 2:**
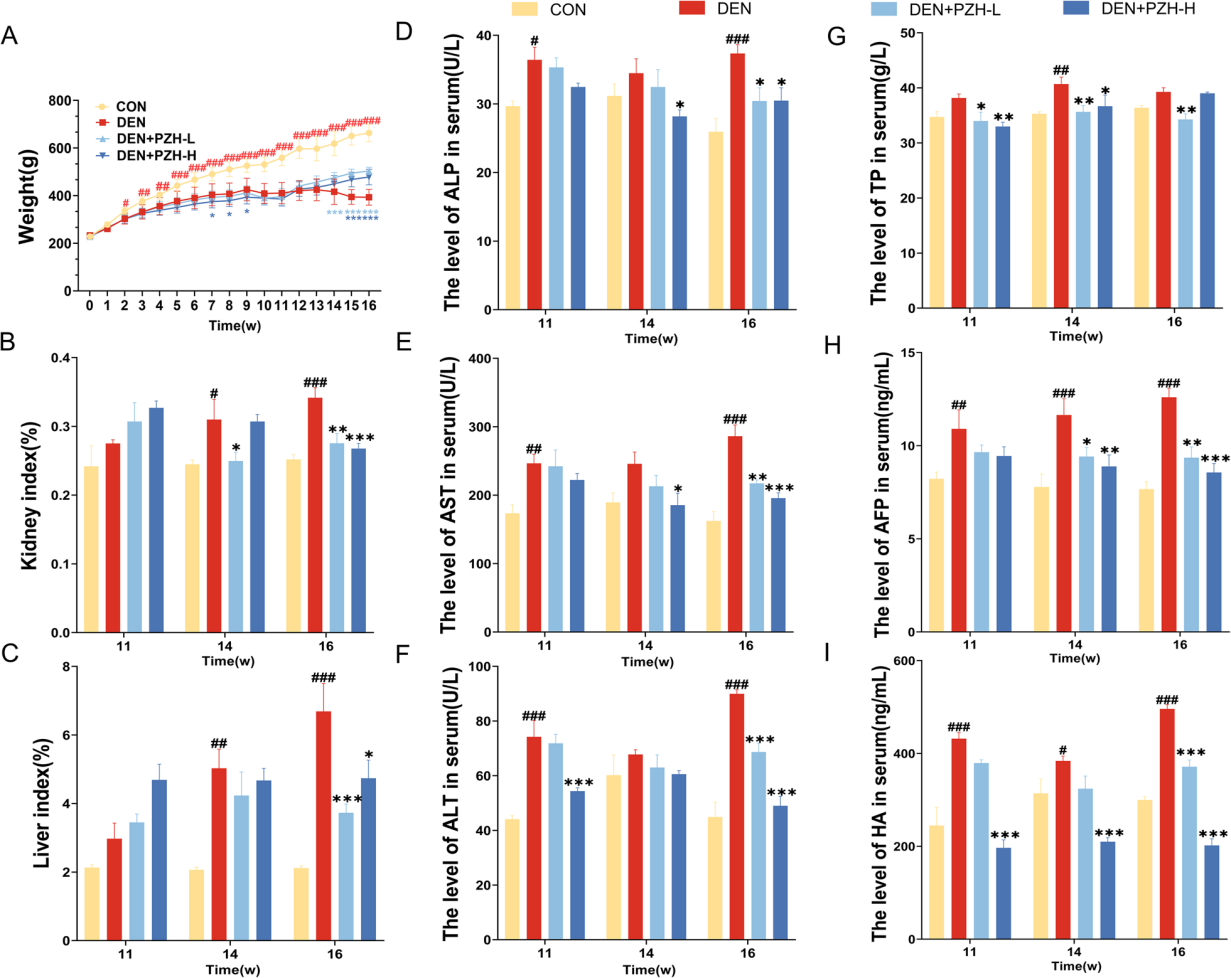
Pharmacological effects of PZH on body weight, renal index, liver index and hepatic function-related serum indices (ALP, AST, ALT, TP) as well as fibrosis (HA) and tumor-related serum markers (AFP) in rats with DEN-induced HCC. **A** Body weights of rats in different groups. **B** Renal index of rats in different groups. **C** Liver indices of different groups of rats. Serum ALP (**D**), AST **(E**), ALT (**F**), TP (**G**), AFP (**H**), and HA (**I**) levels. Data are expressed as mean ± standard deviation. ^#^*P* < 0.05, ^##^* P* < 0.01, ^###^* P* < 0.001, compared with normal group; ^*^ P < 0.05, ^**^ P < 0.01, ^***^ P < 0.001, compared with DEN group

By monitoring the body weight of rats for 16 weeks, we observed that PZH effectively improved the body weight reduction in DEN rats caused by the occurrence and progression of HCC, and further autopsy of rats showed that their liver and kidney pathological damage was also significantly alleviated. Starting from the 2nd week of administration, the body weights of the DEN model, PZH low and high dose groups were dramatically lower than the normal group (all* P* < 0.05, Fig. 2A). The body weights of the DEN model group decreased at week 9, week 13, and after PZH treatment, the body weights of the PZH low and high dose groups increased rapidly from week 11 and were markedly higher than the model group at week 14 (all* P* < 0.001). After 14 weeks, the liver index (all* P* < 0.01, Fig. 2C) and kidney index (all* P* < 0.05, Fig. 2B) of the DEN model group were higher than those of the normal control group. After 16 weeks of treatment, the PZH low and high dose groups observably reduced the liver index (all* P* < 0.05) and kidney index (all* P* < 0.01) in DEN-induced rats. We examined the hepatic function-related serum indices and fibrosis and tumor-related serum markers in rats, and found that PZH significantly improved hepatic function, and inhibited fibrosis and tumor progression in DEN-induced HCC rats (Fig. 2). ALP, ALT, and AST were significantly higher in DEN-induced HCC rats than in normal controls at weeks 11 and 16 (all *P* < 0.05, Fig. 2D–F). The hepatic fibrosis markers (HA) and tumor serum markers (AFP) were dramatically higher in the DEN model group than in the normal control group, showing a time-dependent increase (all *P* < 0.05, Fig. 2H–I). In contrast, TP of DEN model rats were statistically different only at 14 weeks compared with the normal control group (*P* < 0.01, Fig. 2G). After the treatment of low-dose PZH, ALP, AST, ALT, TP, HA, and AFP were decreased in DEN-induced rats (all *P* < 0.05, Fig. 2D–I), similar to the changes of the PZH high-dose group (all *P* < 0.05 Fig. 2D–I), except TP without any significant differences.

### PZH has a potential to reverse the malignant transformation from hepatic fibrosis to HCC

In order to identify the effective genes of PZH in the malignant transformation process of “hepatic fibrosis-HCC”, we collected liver tissues from normal control, DEN model and PZH administration groups and performed mRNA sequencing to obtain transcriptomic datasets of the rat liver tissues. Differences between groups were screened by |log Fold Change|> 2, *P* < 0.001, and genes of no specific significance were removed. As shown in Additional file 1**: **Tables S1 and S2, there were 1993 HCC-related genes with differential expression patterns by comparing between the DEN model group and the normal control group, including 1470 up-regulated genes and 523 down-regulated genes. In addition, there were 1757 PZH effective genes, including 781 up-regulated genes and 976 down-regulated genes by comparing between the PZH administration group and the DEN model group.

**Fig. 3:**
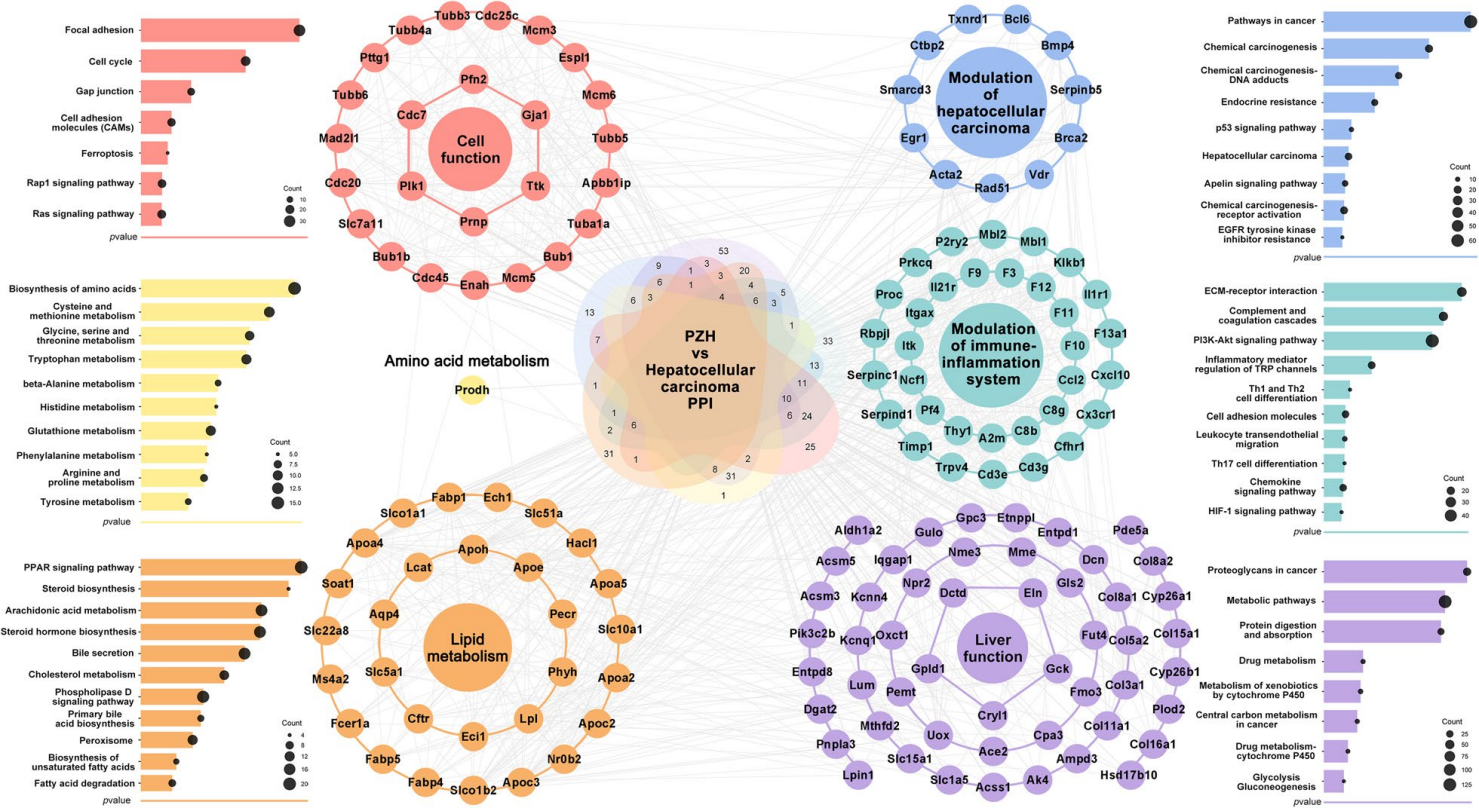
The main functional modules of the “HCC-related gene-PZH effective gene” interaction network and the enriched pathways of the PZH candidate targets against HCC. The circular nodes represent PZH candidate targets and the long bars represent the enriched pathways. The PZH candidate targets were significantly enriched into six network modules, respectively cell function, amino acid metabolism, lipid metabolism, modulation of HCC, modulation of immune inflammatory and liver function modules. *P* < 0.05 was used as the criterion for screening channels

Following the construction of the “disease gene—drug target” network based on the links among the HCC-related genes and the PZH effective genes, a total of 647 core nodes with topological features in the network were screened as candidate targets of PZH against HCC. Functionally, the PZH candidate targets were significantly enriched into six network modules (Fig. 3). In the cell functional module, focal adhesion kinase (FAK), one of the signaling molecules of focal adhesion, was highly expressed in HCC tissues, and was reported to be activated to promote metastasis and adhesion of cancer cells [16]. Recent studies have also indicated that FAK may regulate proliferation, survival and angiogenesis of cancer cells, and function as a prognostic indicator of HCC [17]. In addition, various anti-cancer drugs such as sorafenib may regulate intracellular iron metabolism and lipid peroxidation to promote ferroptosis in HCC cells, which has become a new therapeutic strategy for the treatment of HCC [18, 19]. Functional modules of amino acid metabolism included glutathione metabolism, and cysteine and methionine metabolism-related pathways. Functional modules of lipid metabolism included the classical PPAR signaling and the peroxisome pathways. The development and progression of HCC may be closely related to lipid biosynthesis and desaturation, and reversing the imbalance of lipid metabolism may be one of the therapeutic approaches for HCC [20, 21]. Moreover, the HCC-related functional module included the chemical carcinogenesis, p53 signaling pathway, and apelin signaling pathway, and the immune inflammatory system-related functional module was involved by PI3K-Akt signaling pathway, which has been reported to be highly expressed in HCC patients and closely related to cancer cell proliferation, survival, invasion and angiogenesis [22]. These data imply that PZH might have a potential to reverse the malignant transformation from hepatic fibrosis to HCC.

### PZH reverses the malignant transformation from hepatic fibrosis to HCC by promoting cellular ferroptosis via the SLC7A11-GSH-GPX4 axis

**Fig. 4:**
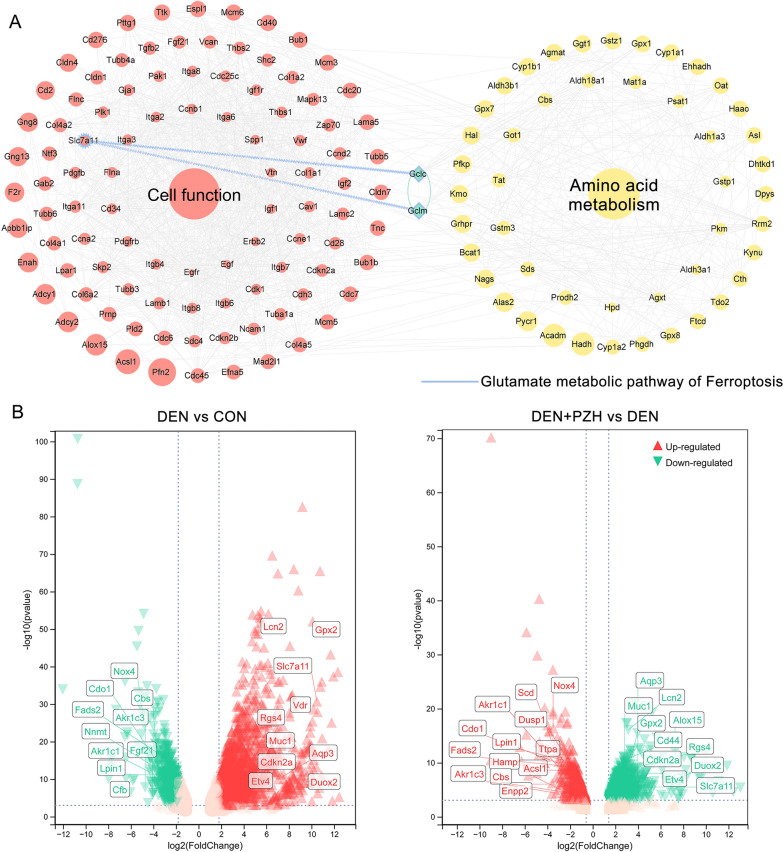
PZH might reverse the malignant transformation from hepatic fibrosis to HCC by promoting cellular ferroptosis via the SLC7A11-GSH-GPX4 axis. **A** Ferroptosis and Glutamate metabolic. **B** Volcano plot showing the expression of ferroptosis -related genes in the transcriptome data

Among the six functional modules of the “HCC-related gene-PZH effective gene” interaction network, we found that the ferroptosis signaling pathway was highly enriched by PZH candidate genes against HCC. Ferroptosis is a newly discovered mode of cell death in recent years, and promoting ferroptosis in HCC cells has become a novel therapeutic strategy [23]. Both SLC7A11 and glutamate-cysteine ligase catalytic (GCLC) and glutamate-cysteine ligase modifier subunit (GCLM) in the amino acid pathway are involved in the ferroptosis process and regulate GSH synthesis indirectly or directly (Fig. 4A) [24]. In our transcriptomic dataset, the ferroptosis related gene SLC7A11 was significantly up-regulated in the model group and dramatically down-regulated after the administration of PZH (Fig. 4B). Notably, accumulating studies have indicated SLC7A11 as a ferroptosis suppressive gene that may be overexpressed in many human cancers [25]. SLC7A11 (also known as xCT) is a member of the constituent cystine/glutamate reverse transporter proteins for uptake of cysteine for glutathione biosynthesis and antioxidant defense [26]. Upon intracellular uptake, cystine is reduced to cysteine, which is the rate-limiting precursor for GSH synthesis [27]. In the presence of GSH, GPX4 mediates the conversion of toxic lipid peroxides to nontoxic lipid alcohols [28]. Inhibition of SLC7A11 may lead to GSH depletion, which subsequently reduces the expression of GPX4, resulting in cellular/subcellular membrane damage caused by iron-dependent accumulation of lipid peroxides [29]. These findings prompt us to hypothesize that PZH might have a potential to regulate DEN-induced pathological changes from hepatic fibrosis to HCC through regulating SLC7A11-GSH-GPX4 axis.

**Fig. 5:**
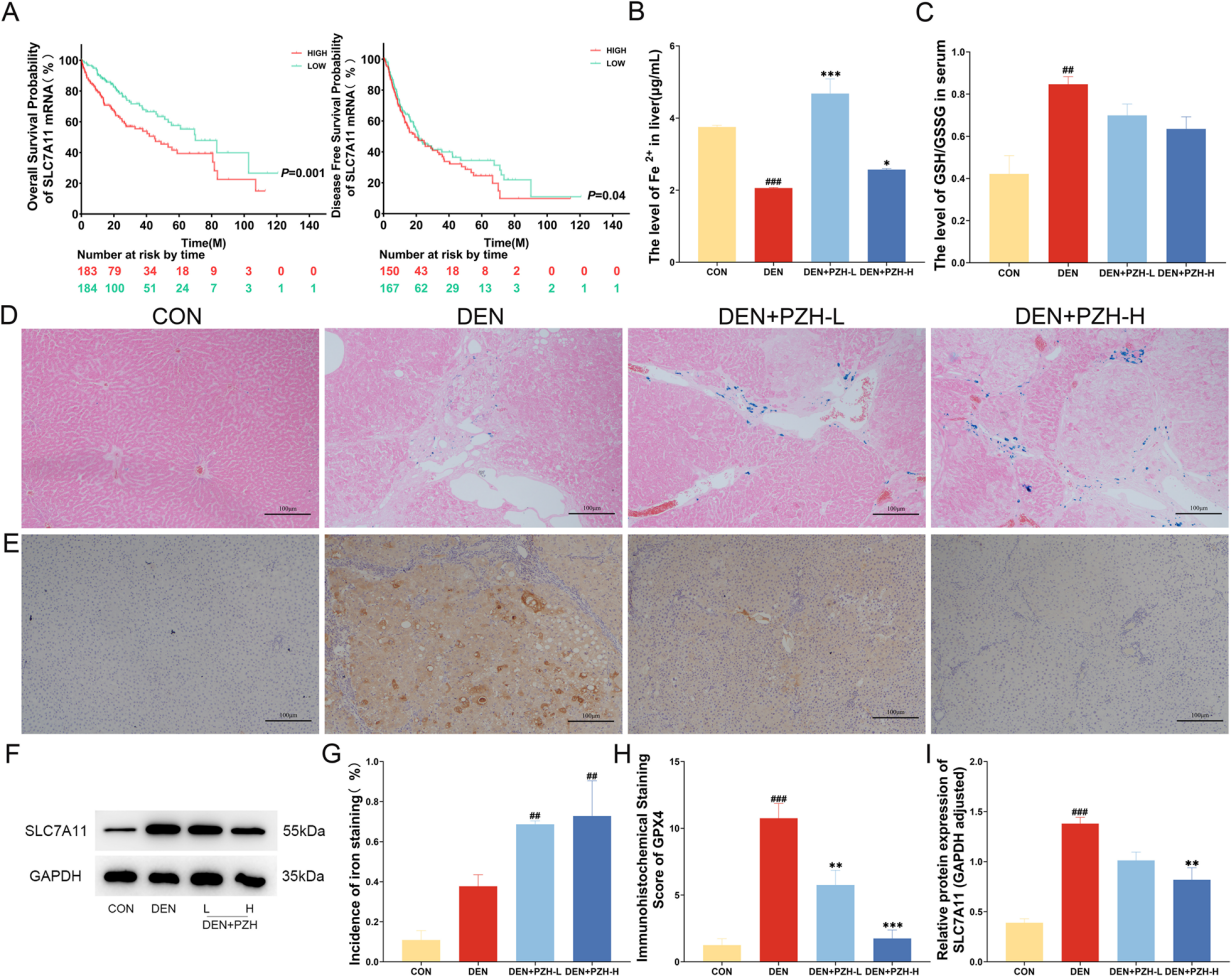
Regulatory effects of PZH on ferroptosis related pathways in the liver tissues of DEN induced HCC rats. **A** Kaplan–Meier survival curve analysis of SLC7A11 expression profile on prognosis of HCC patients. **B** Effect of PZH on Fe^2+^ content in the liver tissues of different groups. **C** Regulatory effects of PZH on serum GSH and GSSG contents of different groups. **D**, **G** Regulatory effects of PZH on liver iron deposition in different groups. Blue color is iron deposition. **E**, **H** Immunohistochemistry staining observed the effect of PZH on GPX4 expression in the liver tissues of different groups, orange color is positive expression. **F**, **I** Western blot assay detected the effects of PZH on SLC7A11 protein expression in the liver tissues of different groups. Data are expressed as mean ± standard deviation. ^#^*P* < 0.05, ^##^*P* < 0.01, ^###^*P* < 0.001, compared with the normal group; ^*^P < 0.05, ^**^P < 0.01, ^***^P < 0.001, compared with the DEN model group

To evaluate the associations of SLC7A11 expression with various clinicopathological characteristics of HCC patients, we divided 373 HCC patients into high (n = 187) and low (n = 186) expression groups according to the median of SLC7A11 expression levels (63.54). According to the statistical results, SLC7A11 overexpression was more frequently occurred in HCC patients with high preoperative serum AFP level, and unfavorable overall survival status (all 0.01 < *P* < 0.05, Table 1). According to Kaplan–Meier survival curve analysis shown in Fig. 5A, HCC patients with high SLC7A11 expression had significantly shorter disease-free survival (*P* = 0.04) and overall survival (*P* = 0.001) than those with low SLC7A11 expression.

**Table 1:** Associations between SLC7A11 expression and various clinicopathological features in HCC patients based on TCGA dataset

Features		No. of patients	SLC7A11 expression		P
			High (n,%)	Low (n,%)	
Gender	Male	252	136(53.97)	116 (46.03)	0.01 < p < 0.05
	Female	121	51 (42.15)	70 (57.85)	
Age (years)	< 61	177	84 (47.46)	93 (52.54)	0.05
	≧61	195	102 (52.31)	93 (47.69)	
Preoperative serum AFP level	Positive	148	82 (55.41)	66 (44.59)	0.01 < p < 0.05
	Negative	131	55 (41.98)	76 (58.02)	
Tumor Stage (American Joint Committee on Cancer)	T1 ~ T2	277	136 (49.10)	141 (50.90)	0.06
	T3 ~ T4	93	51 (54.84)	42 (45.16)	
Metastasis Stage (American Joint Committee on Cancer)	M0	267	134(50.19)	133 (49.81)	0.25
	M1	4	1 (25.00)	3 (75.00)	
Lymph Node Stage (American Joint Committee on Cancer)	N0	253	128(50.59)	125 (49.41)	0.26
	N1	4	3 (75.00)	1 (25.00)	
Disease Free Status	Non- Recurred	146	65 (44.52)	81 (55.48)	0.05
	Recurred	176	89 (50.57)	87 (49.43)	
Overall Survival Status	Living	243	110 (45.27)	133 (54.73)	0.01 < p < 0.05
	Deceased	130	77 (59.23)	53 (40.77)	
Hepatic Fibrosis Ishak Score	0	75	35(46.67)	40(53.33)	0.12
	1 ~ 4	59	30(50.85)	29(49.15)	
	5 ~ 6	80	38(47.50)	42 (52.50)	
Neoplasm Disease Stage (American Joint Committee on Cancer)	Stage I	172	80(46.51)	92(53.49)	> 0.05
	Stage II	87	48(55.17)	39(44.83)	
	Stage III	85	49(57.65)	38(44.71)	
	Stage IV	5	2(40.00)	3(60.00)	
Neoplasm Histologic Grade	G1	55	20(36.36)	35(63.64)	> 0.05
	G2	178	89(50.00)	89(50.00)	
	G3	123	71(57.72)	52(42.28)	
	G4	12	7(58.33)	5(41.67)	
Vascular Invasion	None	207	96(46.38)	111(53.62)	0.05
	Micro	110	58(52.73)	52(47.27)	
Adjacent Hepatic Tissue Inflammation Extent	None	118	53(44.92)	65(55.08)	> 0.05
	Mild	100	49(49.00)	51(51.00)	
	Severe	18	8(44.44)	10(55.56)	
Surgical Margin Resection Status	R0	326	158(48.47)	168(51.53)	> 0.05
	R1	17	10(58.82)	7(41.18)	
	R2	1	0(0.00)	1(100.00)

To investigate the regulatory effects of PZH on ferroptosis related pathways, we firstly examined the Fe^2+^ content in the liver tissues of DEN-induced HCC rats. Compared with the normal group, the Fe^2+^ content in the liver tissues of DEN rats in the model group was decreased significantly (*P* < 0.001, Fig. 5B), which was reversed by the treatment of PZH effectively (*P* < 0.05, Fig. 5B). In addition, prussian blue iron staining was used to detect iron deposition in the liver tissues, and Figs. 5D and G displayed that there was a small amount of blue iron deposition in the DEN model group compared to the normal control group, but a large amount of blue iron deposition appeared after the treatment of PZH (*P* < 0.01). Moreover, western blot results demonstrated that the ferroptosis inhibitor SLC7A11 was significantly up-regulated in the DEN model group (*P* < 0.001), but was dramatically reduced by PZH treatment (*P* < 0.01), as shown in Fig. 5F–I. The regulatory effects of PZH on GSH as well as GSSG levels was determined by ELISA using the serum collected from different groups. As shown in Fig. 5C, PZH treatment could reduce the ratio of GSH to GSSG. Further immunohistochemical staining indicated that the ferroptosis inhibitor GPX4 was positively stained in the cytoplasm and nucleus of tumor cells and the immunoreactive score of GPX4 protein was also markedly up-regulated in the DEN model group, which was dose-dependently reduced by the treatment of PZH (all *P* < 0.01, Fig. 5E and H).

## Discussion

HCC has become the sixth most common cancer and is a highly aggressive tumor. Patients have poor prognosis and 5-year survival rate is less than 20% [30], so it is urgent to find potential drugs that can control the progression of HCC. Drug repositioning is a research method used to discover new indications for existing drugs, greatly reducing the time and cost of new drug development [31]. PZH, a proprietary Chinese medicine [32], has been used for more than 460 years in China and is widely used clinically for cancers such as HCC [33], osteosarcoma [34] and colon cancer [35]. In this study, we performed network analysis combined with experimental validation to evaluate the efficacy of PZH in the treatment of HCC, and explain the action mechanism of PZH against HCC. On the basis of the HCC model of rats induced by DEN, we found that PZH could reduce hepatic fibrosis and cirrhosis, effectively improve the body weight reduction, reduce the pathological damage of liver and kidney, improve hepatic function and inhibit tumor. Further studies revealed that PZH inhibited SLC7A11 protein expression, down-regulated GSH/GSSG ratio, and decreased GPX4 protein expression levels in the liver. The above findings suggest that PZH may promote ferroptosis in HCC cells by inhibiting the SLC7A11-GSH-GPX4 axis.

HCC is a multi-stage malignant transformation process, which in most cases is a dynamic change from chronic hepatitis to hepatic fibrosis, cirrhosis, and finally HCC [36]. Herein, the DEN-induced rat HCC model was chosen to simulate the above malignant transformation [37]. From the general and pathological observation on the liver tissues, we observed 3 stages in DEN-induced rat HCC models, including the inflammation stage, the fibrosis stage and the HCC stage. After PZH treatment, the histological changes of liver tissues were improved, and the serum parameters related to hepatic function and tumor-related serum parameters were reversed. According to the above results, it is proved that PZH can inhibit the occurrence of HCC and precancerous lesions, and delay the malignant transformation process of liver “inflammation-cancer”.

To reveal the mechanism of action of PZH intervention in HCC, we screened 647 possible candidate targets of PZH and enriched them for functional modules. Among them, PZH candidate genes were significantly enriched in ferroptosis signaling pathway. Ferroptosis is a new form of cell death discovered in 2012 and is characterized by signature mitochondrial damage, such as reduced or absent mitochondrial cristae [38]. Accumulating studies have revealed that a variety of drugs can act by promoting ferroptosis in cancer cells, for example: erastin, sulfasalazine and sorafenib [38, 39]. Currently, the classical axis SLC7A11-GSH-GPX4 is the primary prevention system for ferroptosis [40]. In transcriptomic data, the SLC7A11 gene was significantly overexpressed in the DEN model group and was associated with poor prognosis in HCC patients. We performed western blot analysis and immunohistochemistry, and found that SLC7A11 and GPX4 protein expression were reduced in HCC cells and the GSH/GSSG ratio was decreased after PZH treatment. Iron metabolism is also one of the mechanisms of ferroptosis. In the cell, free iron exists as Fe^2+^ and Fe^3+^, while circulating iron (Fe^3+^) enters the cell by binding to transferrin receptor 1 (TFR1), and Fe^3+^ is reduced to Fe^2+^ by ferric reductase [41]. When ferroptosis occurs in cells, the excess Fe^2+^ reacts with peroxides in the cytoplasm in a Fenton reaction to produce ROS [42]. In our findings, Fe^3+^ and Fe^2+^ were significantly increased in HCC cells after PZH intervention.

**Fig. 6:**
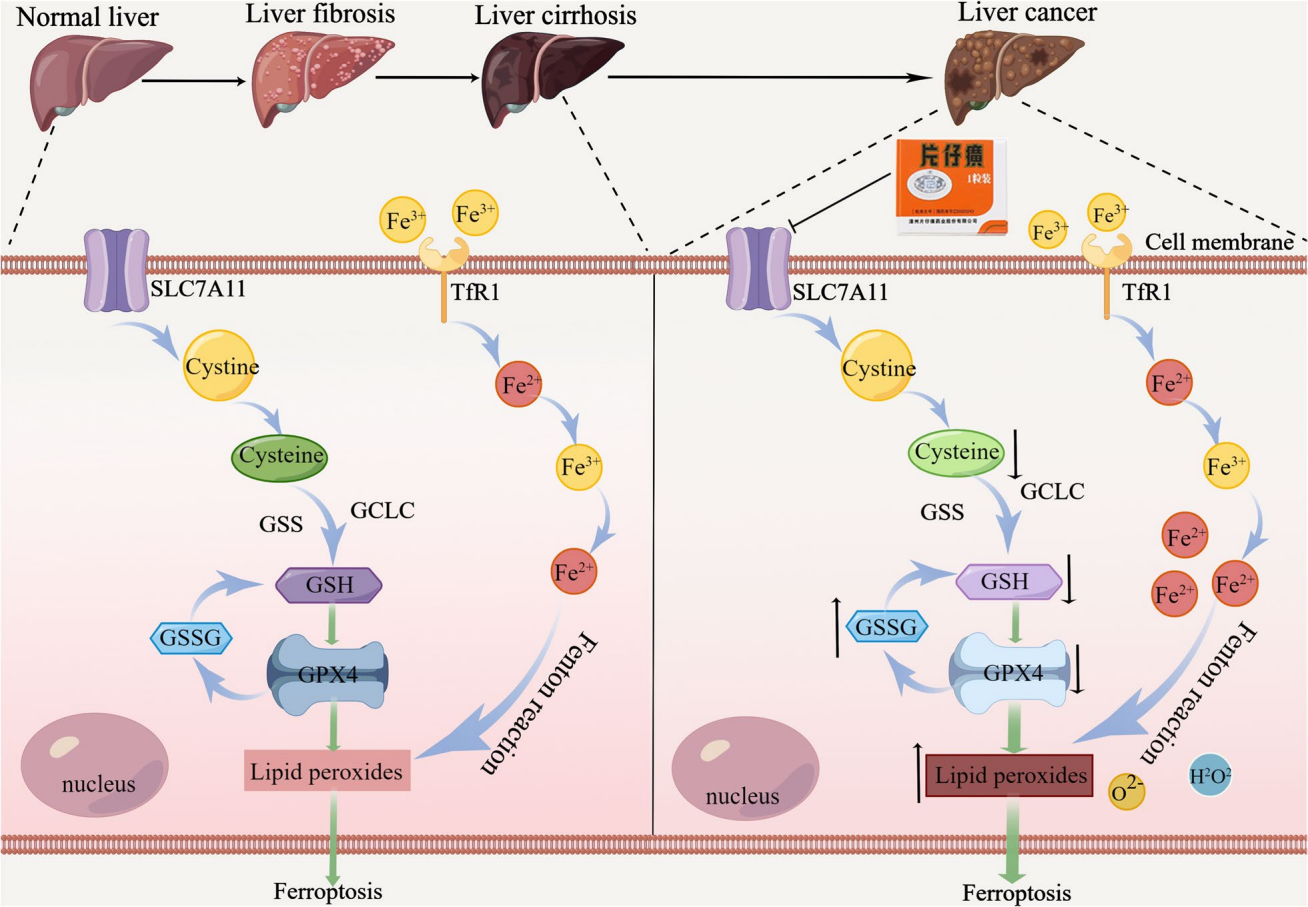
PZH may promote ferroptosis in HCC cells by inhibiting SLC7A11-GSH-GPX4 axis (by Figdraw). In HCC cells, by inhibiting SLC7A11, PZH leads to decreased cystine uptake and reduced GSH synthesis, which in turn downregulates GPX4, leading to cellular/subcellular membrane damage caused by the accumulation of iron-dependent lipid peroxides

In conclusion, our data offer an evidence that PZH may effectively improve the hepatic fibrosis microenvironment and prevent the occurrence of HCC through promoting ferroptosis in tumor cells via inhibiting the SLC7A11-GSH-GPX4 axis (Fig. 6), implying that PZH may be a potential candidate drug for prevention and treatment of HCC at an early stage.

## Supplementary Information

**Additional file 1: Table S1.** Differentially expressed genes between the DEN model group and the normal group. **Table S2.** Differentially expressed genes between the PZH administration group and the DEN model group. **Table S3.** Topological feature values of nodes in the interaction network of disease gene-drug target. **Table S4.** Functional enrichment analysis of PZH putative targets based on KEGG pathway.

## Data Availability

The data sets generated in the current study have been deposited into the NCBI GEO database (GEO No. GSE221790, https://www.ncbi.nlm.nih.gov/geo/query/acc.cgi?acc=GSE221790) and data supporting the results are included in this published article and in the Additional file.

## Abbreviations

AFP: Alpha fetoprotein
ALP: Alkaline phosphatase
ALT: Alanine aminotransferase
AST: Aspartate aminotransferase
DAVID: Database for Annotation, Visualization and Integrated Discovery
DEN: Diethylnitrosamine
ELISA: Enzyme linked immunosorbent assay
FAK: Focal adhesion kinase
GAPDH: Glyceraldehyde-3-phosphate dehydrogenase
GCLC: Glutamate-cysteine ligase catalytic
GCLM: Glutamate-cysteine ligase modifier subunit
GPX4: Glutathione peroxidase 4
GSH: Reduced glutathione
GSSG: Oxidized glutathione
HA: Hyaluronic acid
HCC: Hepatocellular carcinoma
KEGG: Kyoto Encyclopedia of Genes and Genomes
PZH: Pien-Tze-Huang
SD: Sprague–Dawley
SD: Standard deviation
SLC7A11: Solute carrier family 7 member 11
TCM: Traditional Chinese medicine
TFR1: Transferrin receptor 1
TP: Total serum protein
