# Supplementary material for: Pien-Tze-Huang prevents hepatocellular carcinoma by inducing ferroptosis via inhibiting SLC7A11-GSH-GPX4 axis
Source: Cancer Cell Int. 2023 Jun 6;23:109. doi: 10.1186/s12935-023-02946-2 (PMC10246043; doi:10.1186/s12935-023-02946-2)
Supplement: Supplementary file 1 — Additional file 1: Table S1. Differentially expressed genes between the DEN model group and the normal group. Table S2. Differentially expressed genes between the PZH administration group and the DEN model group. Table S3. Topological feature values of nodes in the interaction network of disease gene-drug target. Table S4. Functional enrichment analysis of PZH putative targets based on KEGG pathway. [file 12935_2023_2946_MOESM1_ESM.docx]

Additional file

Pien-Tze-Huang Prevents Hepatocellular Carcinoma by Inducing Ferroptosis via Inhibiting SLC7A11-GSH-GPX4 Axis

Xiangying Yan, Yudong Liu, Congchong Li, Xia Mao, Tengteng Xu, Zhixing Hu, Chu Zhang, Na Lin, Ya Lin*, Yanqiong Zhang*

**Table S1 Differentially expressed genes between the DEN model group and the normal group**

| **GENE** | **MOD** | **CON** | **log2FC** | **Absolute value** | **Pvalue** | **Qvalue** | **updown** |
| --- | --- | --- | --- | --- | --- | --- | --- |
| AABR07048474.1 | 0.190117401 | 1443.628712 | -13.71089756 | 13.71089756 | 1.05E-149 | 2.03E-145 | DOWN |
| Mup4 | 0.048046763 | 716.3084349 | -14.40514957 | 14.40514957 | 9.64E-110 | 9.30E-106 | DOWN |
| AABR07047899.1 | 0 | 425.5930299 | -15.30217357 | 15.30217357 | 1.97E-106 | 1.27E-102 | DOWN |
| LOC298111 | 0.021061179 | 109.415397 | -12.679085 | 12.679085 | 2.67E-104 | 1.29E-100 | DOWN |
| Cyp2c11 | 0.818756234 | 787.4226644 | -10.71123984 | 10.71123984 | 2.10E-101 | 8.11E-98 | DOWN |
| LOC259244 | 0.489797306 | 3676.586832 | -13.66379462 | 13.66379462 | 1.34E-89 | 4.32E-86 | DOWN |
| Mup4 | 0.056999889 | 59.21472232 | -10.72646115 | 10.72646115 | 2.38E-89 | 6.56E-86 | DOWN |
| Dnaaf1 | 33.33734396 | 0.02939348 | 9.203891501 | 9.203891501 | 2.54E-83 | 6.12E-80 | UP |
| Gstp1 | 2406.306748 | 14.80108955 | 6.519758368 | 6.519758368 | 2.52E-70 | 5.39E-67 | UP |
| RGD1566134 | 0.025070532 | 239.9482498 | -13.53984948 | 13.53984948 | 3.92E-69 | 7.56E-66 | DOWN |
| Fndc1 | 12.49165835 | 0.018786193 | 8.434896123 | 8.434896123 | 9.96E-67 | 1.75E-63 | UP |
| Aldh3a1 | 85.5758407 | 0.02320165 | 10.75861535 | 10.75861535 | 3.45E-66 | 5.54E-63 | UP |
| Cftr | 7.853243774 | 0.032921289 | 7.02232187 | 7.02232187 | 1.43E-65 | 2.13E-62 | UP |
| LOC500473 | 0.050101769 | 270.972251 | -12.97085055 | 12.97085055 | 1.16E-63 | 1.59E-60 | DOWN |
| Akr1b10 | 344.5972112 | 0.42275679 | 8.83877864 | 8.83877864 | 5.08E-61 | 6.53E-58 | UP |
| AABR07048463.1 | 0 | 72.65991946 | -12.19607444 | 12.19607444 | 4.10E-58 | 4.95E-55 | DOWN |
| Dclk1 | 3.522643445 | 0.041650902 | 5.529094878 | 5.529094878 | 1.37E-55 | 1.56E-52 | UP |
| Serpina3c | 200.5085651 | 3265.877392 | -4.866188992 | 4.866188992 | 9.49E-55 | 9.63E-52 | DOWN |
| Lama5 | 13.17278586 | 0.099388028 | 6.188229366 | 6.188229366 | 9.20E-55 | 9.63E-52 | UP |
| Tubb6 | 55.10759072 | 1.123738959 | 4.780052932 | 4.780052932 | 1.24E-54 | 1.20E-51 | UP |
| Ccdc92 | 35.42760925 | 0.512963465 | 5.247068547 | 5.247068547 | 1.88E-54 | 1.73E-51 | UP |
| Dcdc2 | 12.63630912 | 0.162801672 | 5.412047856 | 5.412047856 | 6.83E-54 | 5.99E-51 | UP |
| Lcn2 | 115.9978935 | 1.379983109 | 5.524977971 | 5.524977971 | 1.32E-53 | 1.11E-50 | UP |
| Gpx2 | 179.7040842 | 0.081418967 | 10.10902931 | 10.10902931 | 9.24E-53 | 7.12E-50 | UP |
| Ddr1 | 16.87622463 | 0.194184871 | 5.58667633 | 5.58667633 | 1.88E-52 | 1.40E-49 | UP |
| Egln3 | 24.7251688 | 0.370989238 | 5.215902791 | 5.215902791 | 5.64E-51 | 4.03E-48 | UP |
| Abcc3 | 19.19074924 | 0.346150707 | 4.958734585 | 4.958734585 | 2.62E-50 | 1.80E-47 | UP |
| Zfp354a | 1.816100072 | 40.183407 | -5.31083951 | 5.31083951 | 3.16E-50 | 2.10E-47 | DOWN |
| Sfrp1 | 25.00450042 | 0.410104345 | 5.054438663 | 5.054438663 | 1.27E-49 | 8.14E-47 | UP |
| Cd24 | 73.83060369 | 1.054061974 | 5.255065293 | 5.255065293 | 2.04E-49 | 1.27E-46 | UP |
| Cryab | 65.60048839 | 1.347817731 | 4.740603226 | 4.740603226 | 1.78E-47 | 1.07E-44 | UP |
| Epdr1 | 14.9599101 | 0.027829085 | 8.095476308 | 8.095476308 | 3.23E-46 | 1.89E-43 | UP |
| Car2 | 295.8659806 | 4.79054818 | 5.135632194 | 5.135632194 | 4.11E-46 | 2.33E-43 | UP |
| Ust5r | 2.511068999 | 63.27764432 | -5.458089507 | 5.458089507 | 4.47E-46 | 2.46E-43 | DOWN |
| Bicc1 | 27.12963673 | 0.271287898 | 5.791906718 | 5.791906718 | 9.18E-46 | 4.92E-43 | UP |
| RGD1559960 | 1.085141578 | 16.11955062 | -4.754474739 | 4.754474739 | 2.39E-44 | 1.25E-41 | DOWN |
| Nefl | 9.448546064 | 0 | 11.69880612 | 11.69880612 | 5.87E-44 | 2.98E-41 | UP |
| LOC100912195 | 35.02387971 | 0 | 11.29573505 | 11.29573505 | 3.94E-42 | 1.95E-39 | UP |
| Myh10 | 11.94596292 | 0.453818868 | 3.872007976 | 3.872007976 | 4.71E-42 | 2.27E-39 | UP |
| Mcpt1 | 17.67442105 | 0.13583958 | 6.150047875 | 6.150047875 | 1.03E-41 | 4.87E-39 | UP |
| Mki67 | 7.119510488 | 0.204920609 | 4.256895824 | 4.256895824 | 1.47E-41 | 6.76E-39 | UP |
| Ltbp2 | 8.617193703 | 0.10984105 | 5.423783456 | 5.423783456 | 3.78E-41 | 1.68E-38 | UP |
| Adam8 | 10.75937026 | 0.124664457 | 5.574263364 | 5.574263364 | 3.84E-41 | 1.68E-38 | UP |
| Spint1 | 17.39230705 | 0.160204734 | 5.879643025 | 5.879643025 | 1.26E-40 | 5.38E-38 | UP |
| Tceal9 | 138.6683831 | 4.69427888 | 4.038733528 | 4.038733528 | 2.60E-40 | 1.09E-37 | UP |
| Loxl1 | 18.93956306 | 0.322562276 | 5.011602745 | 5.011602745 | 3.58E-40 | 1.47E-37 | UP |
| Gpc3 | 65.58861652 | 0.43190451 | 6.331799658 | 6.331799658 | 6.38E-40 | 2.56E-37 | UP |
| Aplnr | 14.05050599 | 0 | 12.33620012 | 12.33620012 | 2.58E-39 | 1.01E-36 | UP |
| AABR07005844.1 | 231.8630928 | 1.685269337 | 6.188020234 | 6.188020234 | 5.63E-39 | 2.17E-36 | UP |
| Ampd3 | 15.80468498 | 0.09958711 | 6.395097371 | 6.395097371 | 8.37E-39 | 3.16E-36 | UP |
| Cpxm2 | 11.05541348 | 0.178693133 | 5.086054803 | 5.086054803 | 1.98E-38 | 7.35E-36 | UP |
| Gucy2c | 10.19120385 | 0 | 11.93834854 | 11.93834854 | 4.15E-38 | 1.51E-35 | UP |
| Sox9 | 8.995739558 | 0.101904899 | 5.587009141 | 5.587009141 | 4.97E-38 | 1.77E-35 | UP |
| Lrrc75b | 10.32229986 | 0.370957476 | 3.94823264 | 3.94823264 | 8.38E-38 | 2.94E-35 | UP |
| Ggt1 | 38.97939629 | 0.238787522 | 6.487937023 | 6.487937023 | 2.12E-37 | 7.29E-35 | UP |
| Lamc2 | 15.55293421 | 0.293372898 | 4.830335355 | 4.830335355 | 3.30E-37 | 1.12E-34 | UP |
| Sema3b | 8.602604789 | 0.024712253 | 7.48516785 | 7.48516785 | 3.73E-37 | 1.24E-34 | UP |
| Dhrs7l1 | 2.171158766 | 115.8442738 | -6.511660822 | 6.511660822 | 8.92E-37 | 2.91E-34 | DOWN |
| Ms4a4e | 31.77406908 | 0.116299174 | 7.139251423 | 7.139251423 | 9.17E-37 | 2.95E-34 | UP |
| Espn | 5.609435598 | 0 | 10.91011293 | 10.91011293 | 9.81E-37 | 3.10E-34 | UP |
| Elovl7 | 28.86874319 | 0.113501817 | 7.025244857 | 7.025244857 | 1.32E-36 | 4.11E-34 | UP |
| Cd9 | 66.58961833 | 1.998421852 | 4.184365212 | 4.184365212 | 1.35E-36 | 4.14E-34 | UP |
| Emp1 | 59.70678539 | 1.030456048 | 4.979383366 | 4.979383366 | 3.52E-36 | 1.04E-33 | UP |
| Osmr | 21.91739332 | 1.031839238 | 3.550875625 | 3.550875625 | 4.35E-36 | 1.27E-33 | UP |
| AABR07005821.1 | 85.11951041 | 1.728797719 | 4.737112807 | 4.737112807 | 7.26E-36 | 2.09E-33 | UP |
| Snx10 | 36.30364646 | 1.379955954 | 3.858695175 | 3.858695175 | 7.82E-36 | 2.22E-33 | UP |
| Olr59 | 0.417797928 | 5.629449972 | -4.571059016 | 4.571059016 | 1.73E-35 | 4.84E-33 | DOWN |
| Itgb6 | 8.687777719 | 0.070296207 | 6.071566618 | 6.071566618 | 2.21E-35 | 6.09E-33 | UP |
| Klhl14 | 6.206418758 | 0 | 10.79087466 | 10.79087466 | 2.41E-35 | 6.55E-33 | UP |
| Fblim1 | 14.26803578 | 0.122630836 | 5.944523802 | 5.944523802 | 7.48E-35 | 2.00E-32 | UP |
| Plekhb1 | 1.936738853 | 14.76506128 | -3.754828827 | 3.754828827 | 1.06E-34 | 2.79E-32 | DOWN |
| Stac3 | 0 | 15.38364257 | -12.03114495 | 12.03114495 | 1.26E-34 | 3.30E-32 | DOWN |
| Anxa2 | 245.5178595 | 4.711918019 | 4.806368541 | 4.806368541 | 1.36E-34 | 3.50E-32 | UP |
| Cd276 | 18.13395075 | 0.979852054 | 3.354615156 | 3.354615156 | 4.41E-34 | 1.12E-31 | UP |
| Hsd3b5 | 4.601849636 | 101.0403352 | -5.334207504 | 5.334207504 | 7.95E-34 | 1.99E-31 | DOWN |
| Serinc2 | 19.84483032 | 0.170728561 | 5.971261508 | 5.971261508 | 9.68E-34 | 2.39E-31 | UP |
| Ppic | 20.51545222 | 0.303491537 | 5.194688164 | 5.194688164 | 1.40E-33 | 3.42E-31 | UP |
| Ehf | 15.32832023 | 0.098527795 | 6.382737208 | 6.382737208 | 1.61E-33 | 3.88E-31 | UP |
| Gabrp | 19.43403066 | 0.029417877 | 8.316018192 | 8.316018192 | 3.14E-33 | 7.47E-31 | UP |
| Svep1 | 3.404050963 | 0.079217637 | 4.560941852 | 4.560941852 | 4.12E-33 | 9.68E-31 | UP |
| RGD1561161 | 6.178542862 | 0.274594902 | 3.631004789 | 3.631004789 | 4.22E-33 | 9.81E-31 | UP |
| Cldn4 | 14.77205916 | 0.023030519 | 8.262542361 | 8.262542361 | 5.91E-33 | 1.36E-30 | UP |
| Apoa2 | 102.6841893 | 1558.882723 | -4.70088095 | 4.70088095 | 8.18E-33 | 1.85E-30 | DOWN |
| Vdr | 5.518092222 | 0.009168007 | 8.146855095 | 8.146855095 | 1.15E-32 | 2.58E-30 | UP |
| Nlrp12 | 0.723744977 | 11.16639276 | -4.744800934 | 4.744800934 | 1.83E-32 | 4.05E-30 | DOWN |
| AABR07048487.2 | 0 | 84.68047109 | -11.98593529 | 11.98593529 | 1.87E-32 | 4.10E-30 | DOWN |
| Col12a1 | 12.03306778 | 0.345705208 | 4.223936527 | 4.223936527 | 2.81E-32 | 6.09E-30 | UP |
| Vldlr | 12.84320981 | 0.227224684 | 4.93568222 | 4.93568222 | 3.46E-32 | 7.42E-30 | UP |
| Cd44 | 26.0985411 | 0.687357492 | 4.348381261 | 4.348381261 | 3.80E-32 | 8.05E-30 | UP |
| Pla2g7 | 93.53210093 | 0.337673104 | 7.18927443 | 7.18927443 | 4.10E-32 | 8.59E-30 | UP |
| Heph | 15.33832685 | 0.242108459 | 5.09964622 | 5.09964622 | 5.53E-32 | 1.15E-29 | UP |
| Slc46a1 | 4.817590746 | 23.69086553 | -3.131963255 | 3.131963255 | 9.16E-32 | 1.88E-29 | DOWN |
| Plod2 | 19.47913496 | 0.307830282 | 5.066915928 | 5.066915928 | 1.02E-31 | 2.06E-29 | UP |
| Csmd1 | 17.55934543 | 0.346984812 | 4.865990295 | 4.865990295 | 1.82E-31 | 3.65E-29 | UP |
| Slc7a11 | 57.97816165 | 0.020580955 | 10.47792938 | 10.47792938 | 2.03E-31 | 4.03E-29 | UP |
| Slc22a8 | 14.43085614 | 103.1135004 | -3.649289117 | 3.649289117 | 2.17E-31 | 4.27E-29 | DOWN |
| Crot | 18.18758939 | 122.1771314 | -3.564338162 | 3.564338162 | 4.56E-31 | 8.89E-29 | DOWN |
| Kif12 | 16.77812112 | 0.195824056 | 5.57199744 | 5.57199744 | 6.55E-31 | 1.26E-28 | UP |
| Serpinb1a | 344.1522393 | 1.33937759 | 7.077465377 | 7.077465377 | 7.54E-31 | 1.44E-28 | UP |
| Top2a | 10.45011433 | 0.307083286 | 4.192805034 | 4.192805034 | 7.62E-31 | 1.44E-28 | UP |
| Sgpp2 | 7.473283764 | 0.082679297 | 5.585858475 | 5.585858475 | 1.23E-30 | 2.30E-28 | UP |
| Acnat2 | 2.201233575 | 48.40768396 | -5.214782495 | 5.214782495 | 1.51E-30 | 2.81E-28 | DOWN |
| Lamb1 | 27.99080278 | 1.391940056 | 3.464542682 | 3.464542682 | 1.73E-30 | 3.17E-28 | UP |
| Cdh3 | 5.026977848 | 0.050332811 | 5.747005024 | 5.747005024 | 2.13E-30 | 3.88E-28 | UP |
| Adamts2 | 22.28479908 | 0.491574097 | 4.626876134 | 4.626876134 | 2.25E-30 | 4.06E-28 | UP |
| Anxa5 | 239.9118075 | 10.5016191 | 3.644700815 | 3.644700815 | 2.89E-30 | 5.16E-28 | UP |
| Cdcp1 | 3.259002029 | 0.003954445 | 8.480204797 | 8.480204797 | 3.39E-30 | 5.99E-28 | UP |
| RGD1310819 | 4.118644601 | 0.014831506 | 7.136319673 | 7.136319673 | 3.47E-30 | 6.08E-28 | UP |
| S100a10 | 143.780421 | 7.65297 | 3.364663092 | 3.364663092 | 4.61E-30 | 8.01E-28 | UP |
| Prnp | 34.31170167 | 1.644096132 | 3.531066211 | 3.531066211 | 6.62E-30 | 1.14E-27 | UP |
| Cdh13 | 4.918049436 | 0.082752563 | 5.020968714 | 5.020968714 | 7.96E-30 | 1.36E-27 | UP |
| Kynu | 15.33194283 | 95.01664639 | -3.440850569 | 3.440850569 | 1.08E-29 | 1.82E-27 | DOWN |
| Cyp3a9 | 2.920321756 | 24.82301143 | -3.891852212 | 3.891852212 | 1.22E-29 | 2.03E-27 | DOWN |
| Sctr | 15.37742553 | 0.243796255 | 5.09542682 | 5.09542682 | 1.22E-29 | 2.03E-27 | UP |
| Defb1 | 25.61534732 | 0 | 10.1974351 | 10.1974351 | 2.32E-29 | 3.82E-27 | UP |
| Aox3 | 0.065103079 | 6.362258686 | -7.318329643 | 7.318329643 | 3.25E-29 | 5.31E-27 | DOWN |
| Prc1 | 9.266736301 | 0.223651029 | 4.489221597 | 4.489221597 | 3.77E-29 | 6.05E-27 | UP |
| Col11a1 | 3.334342163 | 0.013233517 | 7.001058611 | 7.001058611 | 4.37E-29 | 6.96E-27 | UP |
| Sorcs2 | 3.00757002 | 0.085033521 | 4.299450395 | 4.299450395 | 4.71E-29 | 7.45E-27 | UP |
| Kdelr3 | 24.66374904 | 0.782558981 | 4.094718786 | 4.094718786 | 5.00E-29 | 7.84E-27 | UP |
| Pamr1 | 39.53945827 | 1.52172323 | 3.832629197 | 3.832629197 | 6.24E-29 | 9.71E-27 | UP |
| Pqlc3 | 22.88050321 | 0.677012186 | 4.195654405 | 4.195654405 | 1.28E-28 | 1.97E-26 | UP |
| Gipc2 | 12.31491638 | 0 | 10.54169117 | 10.54169117 | 1.31E-28 | 2.00E-26 | UP |
| Trhde | 0.414414387 | 2.738329416 | -3.549101431 | 3.549101431 | 1.33E-28 | 2.02E-26 | DOWN |
| Ddit4l2 | 13.5575539 | 0.022712412 | 8.039281883 | 8.039281883 | 1.97E-28 | 2.96E-26 | UP |
| Myof | 9.83421403 | 0.358098888 | 3.878302319 | 3.878302319 | 2.34E-28 | 3.50E-26 | UP |
| Smoc2 | 14.63736277 | 0.54651464 | 3.880067309 | 3.880067309 | 2.59E-28 | 3.84E-26 | UP |
| Hao2 | 8.493707721 | 140.8614036 | -4.814105488 | 4.814105488 | 2.87E-28 | 4.22E-26 | DOWN |
| Anxa1 | 104.8827687 | 3.292351238 | 4.103075905 | 4.103075905 | 5.58E-28 | 8.15E-26 | UP |
| Sult1c3 | 4.573509026 | 84.15942468 | -4.960884807 | 4.960884807 | 1.02E-27 | 1.48E-25 | DOWN |
| Mfsd2a | 18.19184631 | 151.4604902 | -3.843498641 | 3.843498641 | 1.22E-27 | 1.76E-25 | DOWN |
| Gria3 | 5.924165736 | 0.23465757 | 3.814310414 | 3.814310414 | 1.28E-27 | 1.83E-25 | UP |
| AABR07060293.1 | 4.492659793 | 39.62488497 | -3.964307587 | 3.964307587 | 1.40E-27 | 1.99E-25 | DOWN |
| Slc25a25 | 15.94778043 | 142.5849754 | -3.956318315 | 3.956318315 | 1.49E-27 | 2.10E-25 | DOWN |
| Vwa7 | 4.317253664 | 0.006952169 | 8.081390201 | 8.081390201 | 1.98E-27 | 2.77E-25 | UP |
| Lpl | 78.18039778 | 3.449560285 | 3.639184829 | 3.639184829 | 2.67E-27 | 3.70E-25 | UP |
| Rgs4 | 13.8531084 | 0.118501554 | 5.996585681 | 5.996585681 | 3.12E-27 | 4.29E-25 | UP |
| Synpo | 7.394783235 | 0.426214062 | 3.284706835 | 3.284706835 | 3.53E-27 | 4.82E-25 | UP |
| Ctse | 37.1760186 | 0.466708483 | 5.41485234 | 5.41485234 | 3.89E-27 | 5.28E-25 | UP |
| Cxcl6 | 15.48548336 | 0.075370359 | 6.701569851 | 6.701569851 | 4.03E-27 | 5.43E-25 | UP |
| Clec7a | 16.83478728 | 0.359272285 | 4.648064776 | 4.648064776 | 4.32E-27 | 5.79E-25 | UP |
| Atp2b4 | 3.482272521 | 0.17232457 | 3.491189427 | 3.491189427 | 4.85E-27 | 6.45E-25 | UP |
| Tmbim1 | 16.9050521 | 0.878506135 | 3.387821557 | 3.387821557 | 5.05E-27 | 6.67E-25 | UP |
| Vegfd | 16.58961885 | 0.403421106 | 4.457660585 | 4.457660585 | 6.40E-27 | 8.39E-25 | UP |
| Slco1a4 | 5.443983131 | 62.81378101 | -4.315579413 | 4.315579413 | 6.51E-27 | 8.48E-25 | DOWN |
| Tes | 43.88701872 | 1.246303849 | 4.233767111 | 4.233767111 | 1.72E-26 | 2.23E-24 | UP |
| Tspan2 | 6.963035016 | 0.230284564 | 4.068604401 | 4.068604401 | 2.30E-26 | 2.96E-24 | UP |
| Vsx1 | 3.80876254 | 0 | 10.08973267 | 10.08973267 | 4.42E-26 | 5.64E-24 | UP |
| Tspan8 | 124.5769976 | 0.615068832 | 6.709825402 | 6.709825402 | 4.60E-26 | 5.79E-24 | UP |
| Rab3d | 13.87655459 | 0.619255108 | 3.600475577 | 3.600475577 | 4.58E-26 | 5.79E-24 | UP |
| G6pd | 101.5925298 | 5.591699099 | 3.301543698 | 3.301543698 | 4.76E-26 | 5.96E-24 | UP |
| Mmp12 | 19.14763094 | 0.387217394 | 4.793280713 | 4.793280713 | 5.01E-26 | 6.24E-24 | UP |
| Arhgef16 | 3.72754396 | 0.022680158 | 6.396626682 | 6.396626682 | 5.28E-26 | 6.53E-24 | UP |
| Itgbl1 | 14.38490712 | 0.509669398 | 3.944075027 | 3.944075027 | 5.73E-26 | 7.04E-24 | UP |
| Slc15a1 | 12.41830268 | 0 | 11.78435107 | 11.78435107 | 8.52E-26 | 1.04E-23 | UP |
| Itga8 | 22.93525598 | 0.184655708 | 6.11366522 | 6.11366522 | 8.87E-26 | 1.08E-23 | UP |
| Celsr1 | 2.077798488 | 0.018494557 | 5.927010701 | 5.927010701 | 1.23E-25 | 1.49E-23 | UP |
| Cd34 | 11.44796717 | 0.133825405 | 5.552538861 | 5.552538861 | 1.79E-25 | 2.15E-23 | UP |
| S100a11 | 250.5303675 | 3.231376651 | 5.387411159 | 5.387411159 | 2.86E-25 | 3.39E-23 | UP |
| St6galnac2 | 10.10052811 | 0.234194801 | 4.55206853 | 4.55206853 | 3.93E-25 | 4.61E-23 | UP |
| Cxcl12 | 15.32744713 | 70.66000818 | -3.037795202 | 3.037795202 | 3.99E-25 | 4.66E-23 | DOWN |
| B3galnt1 | 11.72289347 | 0.151092404 | 5.362136116 | 5.362136116 | 4.07E-25 | 4.73E-23 | UP |
| Flnc | 2.085709855 | 0.014862395 | 6.22136813 | 6.22136813 | 4.36E-25 | 5.03E-23 | UP |
| LOC100359539 | 10.27722766 | 0.233849965 | 4.620030747 | 4.620030747 | 4.38E-25 | 5.03E-23 | UP |
| Nox4 | 0.532644317 | 8.012211855 | -4.78762904 | 4.78762904 | 4.93E-25 | 5.62E-23 | DOWN |
| Adgrg1 | 9.117292234 | 0.236666246 | 4.408317104 | 4.408317104 | 5.28E-25 | 5.99E-23 | UP |
| Mreg | 7.266154541 | 67.75709842 | -4.001361417 | 4.001361417 | 6.63E-25 | 7.48E-23 | DOWN |
| Cav1 | 23.42304306 | 1.088653941 | 3.544080985 | 3.544080985 | 6.67E-25 | 7.48E-23 | UP |
| Krt19 | 273.1523389 | 0.817344567 | 7.456576992 | 7.456576992 | 6.87E-25 | 7.66E-23 | UP |
| Prom1 | 10.78585842 | 0.184629491 | 5.000979105 | 5.000979105 | 7.08E-25 | 7.80E-23 | UP |
| Clic5 | 8.901862 | 0.254855581 | 4.216305965 | 4.216305965 | 7.06E-25 | 7.80E-23 | UP |
| Cxcl16 | 88.08887269 | 4.157795871 | 3.510696299 | 3.510696299 | 7.38E-25 | 8.08E-23 | UP |
| Slit3 | 7.068334355 | 0.163270938 | 4.537504003 | 4.537504003 | 7.43E-25 | 8.09E-23 | UP |
| Elfn1 | 2.316133881 | 0 | 9.886452109 | 9.886452109 | 7.70E-25 | 8.34E-23 | UP |
| Spp1 | 39.39168789 | 0.34167985 | 5.928520315 | 5.928520315 | 1.37E-24 | 1.47E-22 | UP |
| Anxa13 | 28.25678231 | 0.522355368 | 4.840071043 | 4.840071043 | 1.46E-24 | 1.57E-22 | UP |
| Tril | 7.220723071 | 0.211666222 | 4.219848704 | 4.219848704 | 1.55E-24 | 1.65E-22 | UP |
| Pitpnm3 | 2.652325889 | 0.0428263 | 5.058544946 | 5.058544946 | 1.57E-24 | 1.67E-22 | UP |
| LOC297568 | 168.0974803 | 1354.694636 | -3.790505482 | 3.790505482 | 1.62E-24 | 1.71E-22 | DOWN |
| A3galt2 | 14.24858753 | 0.224832219 | 5.062131294 | 5.062131294 | 1.66E-24 | 1.74E-22 | UP |
| St14 | 24.07862891 | 0.748714805 | 4.102335576 | 4.102335576 | 1.85E-24 | 1.93E-22 | UP |
| Slc25a4 | 72.33329931 | 3.506403685 | 3.538961284 | 3.538961284 | 1.94E-24 | 2.01E-22 | UP |
| Bex3 | 46.51002514 | 2.375079448 | 3.43821571 | 3.43821571 | 2.24E-24 | 2.31E-22 | UP |
| Mroh2a | 4.851753974 | 0.182742312 | 3.859226962 | 3.859226962 | 2.66E-24 | 2.72E-22 | UP |
| Olfml2b | 13.6357972 | 0.387411702 | 4.245785743 | 4.245785743 | 2.81E-24 | 2.85E-22 | UP |
| Tmc4 | 4.415681372 | 0.015852163 | 7.11031678 | 7.11031678 | 3.13E-24 | 3.16E-22 | UP |
| LOC100364435 | 273.440904 | 15.91076126 | 3.266545453 | 3.266545453 | 3.53E-24 | 3.54E-22 | UP |
| Errfi1 | 121.272167 | 539.423169 | -2.986488601 | 2.986488601 | 3.55E-24 | 3.55E-22 | DOWN |
| Itpr3 | 6.499017869 | 0.102409805 | 5.106172054 | 5.106172054 | 3.98E-24 | 3.96E-22 | UP |
| Degs2 | 14.91529766 | 0.063854283 | 6.885654785 | 6.885654785 | 4.47E-24 | 4.42E-22 | UP |
| Slc6a6 | 14.47051571 | 0.749907166 | 3.402005053 | 3.402005053 | 5.68E-24 | 5.59E-22 | UP |
| Nqo1 | 354.3194278 | 14.60354046 | 3.786298959 | 3.786298959 | 6.94E-24 | 6.79E-22 | UP |
| Pdgfd | 5.761187029 | 0.1438289 | 4.440894319 | 4.440894319 | 7.02E-24 | 6.84E-22 | UP |
| Kitlg | 14.45275777 | 0.740952613 | 3.388300028 | 3.388300028 | 7.97E-24 | 7.73E-22 | UP |
| RGD1311575 | 1.592627096 | 0.005996471 | 7.024098741 | 7.024098741 | 8.02E-24 | 7.74E-22 | UP |
| LOC100911545 | 35.01239994 | 0.726185436 | 4.800986168 | 4.800986168 | 1.43E-23 | 1.36E-21 | UP |
| Ezr | 42.40472743 | 1.88940831 | 3.599966925 | 3.599966925 | 1.55E-23 | 1.47E-21 | UP |
| LOC103692165 | 4.010763236 | 0.016094479 | 6.93480918 | 6.93480918 | 1.76E-23 | 1.66E-21 | UP |
| Ednrb | 44.0443056 | 3.179595973 | 2.964080265 | 2.964080265 | 2.02E-23 | 1.90E-21 | UP |
| Anxa7 | 80.04641858 | 5.872782703 | 2.907844692 | 2.907844692 | 2.11E-23 | 1.97E-21 | UP |
| Kifc3 | 17.388681 | 1.281766285 | 2.943099494 | 2.943099494 | 2.54E-23 | 2.37E-21 | UP |
| Nol3 | 9.639692489 | 0.14937196 | 5.138124743 | 5.138124743 | 2.70E-23 | 2.50E-21 | UP |
| LOC100910235 | 11.29579604 | 215.7135166 | -5.002922726 | 5.002922726 | 2.82E-23 | 2.60E-21 | DOWN |
| Vim | 292.0831909 | 16.23365747 | 3.280974664 | 3.280974664 | 3.29E-23 | 3.02E-21 | UP |
| Lrtm2 | 0.174124459 | 8.632112517 | -6.316817061 | 6.316817061 | 3.49E-23 | 3.18E-21 | DOWN |
| Fhl2 | 22.97186881 | 0.751997568 | 4.066004172 | 4.066004172 | 3.50E-23 | 3.18E-21 | UP |
| Muc1 | 17.06220012 | 0.141867181 | 6.032807196 | 6.032807196 | 3.74E-23 | 3.39E-21 | UP |
| Bcas1 | 2.610171573 | 0 | 9.89458521 | 9.89458521 | 4.07E-23 | 3.67E-21 | UP |
| Pdlim4 | 14.19888642 | 0.190921963 | 5.3592177 | 5.3592177 | 4.58E-23 | 4.10E-21 | UP |
| Cyp4a1 | 55.63598198 | 246.8951468 | -2.972637945 | 2.972637945 | 4.60E-23 | 4.10E-21 | DOWN |
| Ckap2 | 7.053208512 | 0.169292279 | 4.51523577 | 4.51523577 | 4.87E-23 | 4.33E-21 | UP |
| Carmil1 | 5.433244288 | 0.276039671 | 3.447879764 | 3.447879764 | 4.94E-23 | 4.37E-21 | UP |
| Kif23 | 5.124886423 | 0.060425209 | 5.481078572 | 5.481078572 | 5.38E-23 | 4.73E-21 | UP |
| Chrdl1 | 5.080373793 | 0.094566124 | 4.914378041 | 4.914378041 | 6.17E-23 | 5.41E-21 | UP |
| Ehhadh | 79.73906502 | 378.3863772 | -3.079394291 | 3.079394291 | 8.28E-23 | 7.22E-21 | DOWN |
| Cx3cl1 | 12.59402934 | 0.208639802 | 5.055328631 | 5.055328631 | 8.78E-23 | 7.63E-21 | UP |
| Olfml2a | 7.768299387 | 0.351027537 | 3.659001565 | 3.659001565 | 1.06E-22 | 9.16E-21 | UP |
| AABR07067023.1 | 9.207155282 | 0.220676411 | 4.564799813 | 4.564799813 | 1.14E-22 | 9.76E-21 | UP |
| Cyp2c13 | 34.71862188 | 654.5280083 | -5.036777445 | 5.036777445 | 1.15E-22 | 9.85E-21 | DOWN |
| Paqr9 | 7.825496866 | 73.80937243 | -3.995391243 | 3.995391243 | 1.27E-22 | 1.08E-20 | DOWN |
| Hamp | 264.0745045 | 1127.276851 | -2.971798481 | 2.971798481 | 1.54E-22 | 1.30E-20 | DOWN |
| Cyp2c7 | 57.38813167 | 2641.41599 | -6.297358976 | 6.297358976 | 1.60E-22 | 1.35E-20 | DOWN |
| Ccna2 | 6.684544651 | 0.129451605 | 4.815324262 | 4.815324262 | 1.76E-22 | 1.47E-20 | UP |
| Ahnak | 9.843140354 | 0.539366002 | 3.305058577 | 3.305058577 | 1.87E-22 | 1.56E-20 | UP |
| Slc25a24 | 32.54120335 | 0.946367426 | 4.186750762 | 4.186750762 | 2.00E-22 | 1.67E-20 | UP |
| LOC100360095 | 1.885382883 | 1629.553175 | -10.41189756 | 10.41189756 | 2.19E-22 | 1.81E-20 | DOWN |
| 1-Mar | 25.13007068 | 180.4859201 | -3.624460019 | 3.624460019 | 2.23E-22 | 1.84E-20 | DOWN |
| Fxyd3 | 39.47230315 | 0.181391372 | 6.789621945 | 6.789621945 | 2.38E-22 | 1.95E-20 | UP |
| Flrt3 | 10.47954334 | 0.374691758 | 3.942114393 | 3.942114393 | 2.43E-22 | 1.99E-20 | UP |
| Dkk3 | 6.894701164 | 0.281600265 | 3.748618914 | 3.748618914 | 2.51E-22 | 2.05E-20 | UP |
| Klf5 | 17.62810835 | 0.033515225 | 8.04393577 | 8.04393577 | 2.60E-22 | 2.11E-20 | UP |
| Ildr1 | 5.593112641 | 0.008200685 | 8.188620954 | 8.188620954 | 4.85E-22 | 3.91E-20 | UP |
| Nrbf2 | 5.718787089 | 22.2321334 | -2.778192446 | 2.778192446 | 5.43E-22 | 4.36E-20 | DOWN |
| Spta1 | 1.786821535 | 0.002564626 | 8.237660538 | 8.237660538 | 5.69E-22 | 4.55E-20 | UP |
| Cyp4a2 | 15.08403697 | 800.4728778 | -6.52782617 | 6.52782617 | 5.75E-22 | 4.58E-20 | DOWN |
| Cdk1 | 7.738401895 | 0.202430826 | 4.40355984 | 4.40355984 | 6.27E-22 | 4.97E-20 | UP |
| Fbn1 | 29.33632083 | 1.265385607 | 3.631079636 | 3.631079636 | 6.34E-22 | 5.01E-20 | UP |
| Atp10a | 3.29882267 | 0.104396953 | 4.121219847 | 4.121219847 | 6.48E-22 | 5.10E-20 | UP |
| Olr1 | 17.85324796 | 0.15153798 | 5.933944098 | 5.933944098 | 8.14E-22 | 6.36E-20 | UP |
| Dio1 | 10.27849732 | 71.85408442 | -3.614691109 | 3.614691109 | 8.12E-22 | 6.36E-20 | DOWN |
| Aoc1 | 4.846648149 | 0.041948969 | 5.903608084 | 5.903608084 | 9.93E-22 | 7.72E-20 | UP |
| Rap1gap | 6.776334395 | 0.265088334 | 3.821592925 | 3.821592925 | 1.18E-21 | 9.17E-20 | UP |
| Cdhr5 | 7.495850699 | 0 | 10.94622814 | 10.94622814 | 1.21E-21 | 9.32E-20 | UP |
| Insig1 | 37.81190087 | 286.7949294 | -3.744953725 | 3.744953725 | 1.32E-21 | 1.02E-19 | DOWN |
| Slc16a5 | 6.16384044 | 0.01461896 | 7.623424358 | 7.623424358 | 1.38E-21 | 1.05E-19 | UP |
| RGD1565367 | 8.080270094 | 0.072789386 | 5.911054923 | 5.911054923 | 1.86E-21 | 1.42E-19 | UP |
| A2m | 14.96875977 | 0.353282114 | 4.598718304 | 4.598718304 | 2.33E-21 | 1.77E-19 | UP |
| Arhgap11a | 6.730080086 | 0.314364439 | 3.545048098 | 3.545048098 | 2.55E-21 | 1.93E-19 | UP |
| Btnl2 | 3.933327904 | 0 | 9.587087286 | 9.587087286 | 2.89E-21 | 2.18E-19 | UP |
| Cygb | 53.01853947 | 3.183212752 | 3.212431766 | 3.212431766 | 3.29E-21 | 2.47E-19 | UP |
| 8-Sep | 7.711042649 | 0.531292077 | 3.000160127 | 3.000160127 | 3.40E-21 | 2.54E-19 | UP |
| Cenpe | 2.001572131 | 0.029567521 | 5.15617023 | 5.15617023 | 3.41E-21 | 2.54E-19 | UP |
| Fxyd5 | 37.1743303 | 2.432405981 | 3.053917805 | 3.053917805 | 3.48E-21 | 2.58E-19 | UP |
| Il17re | 4.868738724 | 0.039766037 | 6.002825064 | 6.002825064 | 3.75E-21 | 2.77E-19 | UP |
| Fam198b | 12.02969393 | 0.83840157 | 2.977278834 | 2.977278834 | 3.91E-21 | 2.88E-19 | UP |
| Inhbb | 5.908770269 | 0.137666615 | 4.546454517 | 4.546454517 | 4.03E-21 | 2.95E-19 | UP |
| Nid1 | 73.30976811 | 5.406902538 | 2.887237855 | 2.887237855 | 4.32E-21 | 3.15E-19 | UP |
| Ltbp1 | 19.53674448 | 1.651593958 | 2.702894055 | 2.702894055 | 4.47E-21 | 3.24E-19 | UP |
| Wisp2 | 6.908438714 | 0.11315174 | 5.039182125 | 5.039182125 | 4.88E-21 | 3.53E-19 | UP |
| Prag1 | 5.347971398 | 0.154811027 | 4.210400671 | 4.210400671 | 5.30E-21 | 3.80E-19 | UP |
| LOC100912405 | 8.675862196 | 430.832342 | -6.344430934 | 6.344430934 | 5.28E-21 | 3.80E-19 | DOWN |
| Plxdc2 | 15.94892484 | 0.664320889 | 3.705908059 | 3.705908059 | 5.65E-21 | 4.04E-19 | UP |
| Lyz2 | 413.1567917 | 18.37284369 | 3.59058878 | 3.59058878 | 7.11E-21 | 5.06E-19 | UP |
| Wnt5a | 3.039744584 | 0.049001746 | 5.07046786 | 5.07046786 | 7.56E-21 | 5.36E-19 | UP |
| Gsta3 | 111.6886988 | 3.710158152 | 4.070854729 | 4.070854729 | 7.98E-21 | 5.64E-19 | UP |
| Npdc1 | 40.8211615 | 3.33613016 | 2.748379009 | 2.748379009 | 8.35E-21 | 5.88E-19 | UP |
| Pkd2 | 12.30587709 | 1.067849856 | 2.672444259 | 2.672444259 | 1.06E-20 | 7.44E-19 | UP |
| Tnfrsf21 | 17.70991239 | 1.259877542 | 2.929986195 | 2.929986195 | 1.55E-20 | 1.08E-18 | UP |
| LOC680406 | 109.5640575 | 2494.67361 | -5.254685487 | 5.254685487 | 1.57E-20 | 1.09E-18 | DOWN |
| Ptprz1 | 2.831694382 | 0.036320659 | 5.352847957 | 5.352847957 | 1.78E-20 | 1.23E-18 | UP |
| Colec12 | 11.70315941 | 0.701862345 | 3.180393114 | 3.180393114 | 2.02E-20 | 1.39E-18 | UP |
| Trim16 | 3.655160074 | 0.040923745 | 5.549694726 | 5.549694726 | 2.15E-20 | 1.47E-18 | UP |
| Serpina6 | 88.31142544 | 484.0299087 | -3.251848574 | 3.251848574 | 2.45E-20 | 1.67E-18 | DOWN |
| Myo5c | 1.819209085 | 0.020371482 | 5.5656211 | 5.5656211 | 2.65E-20 | 1.80E-18 | UP |
| Aqp7 | 7.52845107 | 0 | 9.87235182 | 9.87235182 | 2.98E-20 | 2.02E-18 | UP |
| Ehd4 | 28.8812779 | 2.676858434 | 2.572299758 | 2.572299758 | 3.17E-20 | 2.14E-18 | UP |
| Ttr | 604.3258113 | 5188.760962 | -3.868550078 | 3.868550078 | 3.41E-20 | 2.29E-18 | DOWN |
| Mgp | 269.7964082 | 12.2776116 | 3.600094827 | 3.600094827 | 4.22E-20 | 2.83E-18 | UP |
| Pim3 | 11.33524819 | 50.58268126 | -2.981498915 | 2.981498915 | 4.39E-20 | 2.93E-18 | DOWN |
| Ica1 | 9.621261244 | 0.392585009 | 3.756460939 | 3.756460939 | 4.70E-20 | 3.13E-18 | UP |
| Cyp2d3 | 42.94688931 | 253.1445505 | -3.335668248 | 3.335668248 | 5.48E-20 | 3.63E-18 | DOWN |
| Apon | 21.4773775 | 173.2199742 | -3.783157621 | 3.783157621 | 5.78E-20 | 3.82E-18 | DOWN |
| AABR07048475.1 | 0 | 12.37813311 | -9.413672998 | 9.413672998 | 5.88E-20 | 3.87E-18 | DOWN |
| Lect2 | 19.12704467 | 70.12107428 | -2.687023158 | 2.687023158 | 6.18E-20 | 4.05E-18 | DOWN |
| Ppl | 6.93319228 | 0.310354497 | 3.654144506 | 3.654144506 | 7.99E-20 | 5.23E-18 | UP |
| Aqp4 | 1.609264855 | 0.015798801 | 5.810992181 | 5.810992181 | 8.05E-20 | 5.25E-18 | UP |
| Nusap1 | 2.518239487 | 0.044147379 | 4.917464608 | 4.917464608 | 8.63E-20 | 5.60E-18 | UP |
| Impdh1 | 13.67823445 | 0.814316556 | 3.199508939 | 3.199508939 | 8.70E-20 | 5.63E-18 | UP |
| Egfr | 12.4698764 | 53.92808869 | -2.910642519 | 2.910642519 | 8.87E-20 | 5.72E-18 | DOWN |
| Fam83b | 1.640515045 | 0 | 9.992639861 | 9.992639861 | 9.98E-20 | 6.41E-18 | UP |
| Pkia | 11.78816214 | 0.572351215 | 3.518685456 | 3.518685456 | 1.03E-19 | 6.62E-18 | UP |
| Akr7a3 | 611.3095043 | 25.42326234 | 3.824565646 | 3.824565646 | 1.08E-19 | 6.89E-18 | UP |
| Serpina4 | 73.52570457 | 575.254228 | -3.729784138 | 3.729784138 | 1.16E-19 | 7.38E-18 | DOWN |
| Dcn | 92.28404025 | 5.195407107 | 3.291243023 | 3.291243023 | 1.17E-19 | 7.39E-18 | UP |
| Cenpf | 2.388018735 | 0.056995666 | 4.477198258 | 4.477198258 | 1.18E-19 | 7.43E-18 | UP |
| Elovl5 | 69.24045398 | 264.9717862 | -2.755871864 | 2.755871864 | 1.21E-19 | 7.61E-18 | DOWN |
| B4galt6 | 5.589675988 | 0.351625392 | 3.098055854 | 3.098055854 | 1.30E-19 | 8.14E-18 | UP |
| Pglyrp1 | 8.982506161 | 0.023860043 | 7.367538548 | 7.367538548 | 1.36E-19 | 8.49E-18 | UP |
| Gckr | 13.61125504 | 52.94175576 | -2.764177662 | 2.764177662 | 1.36E-19 | 8.49E-18 | DOWN |
| Acnat1 | 1.583513181 | 37.96707333 | -5.333809353 | 5.333809353 | 1.40E-19 | 8.71E-18 | DOWN |
| Agmo | 47.22017952 | 169.8556062 | -2.674054123 | 2.674054123 | 1.50E-19 | 9.29E-18 | DOWN |
| AABR07034648.1 | 6.522925523 | 0 | 9.786253882 | 9.786253882 | 1.73E-19 | 1.07E-17 | UP |
| Gpx3 | 170.1330402 | 14.77786226 | 2.653038553 | 2.653038553 | 1.77E-19 | 1.09E-17 | UP |
| Mmp2 | 38.19863303 | 2.312136153 | 3.194964642 | 3.194964642 | 1.79E-19 | 1.10E-17 | UP |
| Gas2l3 | 2.304449065 | 0.053442327 | 4.547565675 | 4.547565675 | 1.95E-19 | 1.19E-17 | UP |
| Acsl1 | 170.682412 | 783.6226474 | -2.998853811 | 2.998853811 | 1.97E-19 | 1.20E-17 | DOWN |
| S100a6 | 212.5938747 | 2.858696097 | 5.279483632 | 5.279483632 | 2.04E-19 | 1.24E-17 | UP |
| Mmrn1 | 10.41136803 | 0.097126413 | 5.880628374 | 5.880628374 | 2.28E-19 | 1.38E-17 | UP |
| Prdm6 | 7.012927781 | 0.205539606 | 4.243477523 | 4.243477523 | 2.32E-19 | 1.40E-17 | UP |
| Ap1m2 | 7.551797248 | 0 | 9.877560235 | 9.877560235 | 2.34E-19 | 1.41E-17 | UP |
| Tat | 167.8261281 | 968.0776779 | -3.31022721 | 3.31022721 | 2.49E-19 | 1.49E-17 | DOWN |
| Rpp21 | 3.137071783 | 11.32382294 | -2.669242777 | 2.669242777 | 2.90E-19 | 1.73E-17 | DOWN |
| Ndrg1 | 54.59197308 | 1.994895875 | 3.88577572 | 3.88577572 | 2.95E-19 | 1.76E-17 | UP |
| Krt7 | 12.41840621 | 0.01800561 | 8.353042495 | 8.353042495 | 3.18E-19 | 1.89E-17 | UP |
| Bub1 | 4.385132746 | 0.177932795 | 3.739386217 | 3.739386217 | 3.40E-19 | 2.01E-17 | UP |
| Sema5a | 2.829852257 | 0.181356063 | 3.08566099 | 3.08566099 | 3.66E-19 | 2.16E-17 | UP |
| Pcdh17 | 3.663779241 | 0.02197575 | 6.491289973 | 6.491289973 | 4.16E-19 | 2.45E-17 | UP |
| Trim59 | 7.275749554 | 0.295561255 | 3.749425751 | 3.749425751 | 4.30E-19 | 2.52E-17 | UP |
| Tspan3 | 74.60576596 | 5.705193006 | 2.828054212 | 2.828054212 | 4.40E-19 | 2.57E-17 | UP |
| Kcnq1 | 4.974488068 | 0.257411144 | 3.419882554 | 3.419882554 | 5.06E-19 | 2.95E-17 | UP |
| Epcam | 115.9151467 | 6.60725691 | 3.231943711 | 3.231943711 | 5.21E-19 | 3.02E-17 | UP |
| Fam129b | 20.73380877 | 2.148422096 | 2.40280784 | 2.40280784 | 5.36E-19 | 3.10E-17 | UP |
| Cldn6 | 5.746450634 | 0 | 9.765524863 | 9.765524863 | 5.55E-19 | 3.20E-17 | UP |
| Fhl1 | 11.78649598 | 0.506861038 | 3.645862076 | 3.645862076 | 5.98E-19 | 3.44E-17 | UP |
| Cldn7 | 18.58305837 | 0.070075888 | 7.058706907 | 7.058706907 | 6.06E-19 | 3.48E-17 | UP |
| Akr1b7 | 34.61943527 | 0.233084104 | 6.48345574 | 6.48345574 | 6.32E-19 | 3.61E-17 | UP |
| Tnnt2 | 5.27447705 | 0 | 9.410751797 | 9.410751797 | 6.92E-19 | 3.95E-17 | UP |
| Knl1 | 2.528024871 | 0.063479717 | 4.42206395 | 4.42206395 | 7.30E-19 | 4.15E-17 | UP |
| Slc17a2 | 7.546462081 | 28.99230671 | -2.740786458 | 2.740786458 | 7.44E-19 | 4.22E-17 | DOWN |
| LOC100361547 | 7.355345229 | 281.0926232 | -6.002265215 | 6.002265215 | 7.55E-19 | 4.27E-17 | DOWN |
| Rasl11b | 6.477868503 | 0.055745938 | 5.908938665 | 5.908938665 | 7.59E-19 | 4.28E-17 | UP |
| Il6r | 13.15801303 | 53.48193869 | -2.825822354 | 2.825822354 | 8.09E-19 | 4.55E-17 | DOWN |
| Aebp1 | 26.49386476 | 1.928327212 | 2.929258507 | 2.929258507 | 8.28E-19 | 4.64E-17 | UP |
| Scd2 | 60.29870426 | 3.087472809 | 3.391010371 | 3.391010371 | 8.59E-19 | 4.80E-17 | UP |
| Notum | 1.197031961 | 13.64257855 | -4.262574602 | 4.262574602 | 8.68E-19 | 4.84E-17 | DOWN |
| Plet1 | 21.55115315 | 0.027302186 | 8.554708987 | 8.554708987 | 9.00E-19 | 5.00E-17 | UP |
| Hk1 | 15.5554572 | 1.068292891 | 2.996559806 | 2.996559806 | 9.08E-19 | 5.03E-17 | UP |
| Flna | 40.04364969 | 3.173026659 | 2.788156006 | 2.788156006 | 9.75E-19 | 5.39E-17 | UP |
| Cpa1 | 17.62684974 | 0 | 11.48785266 | 11.48785266 | 1.10E-18 | 6.06E-17 | UP |
| Mmd | 8.793669301 | 0.283437612 | 4.028695909 | 4.028695909 | 1.10E-18 | 6.07E-17 | UP |
| Dab2 | 34.31870172 | 3.537781167 | 2.414600641 | 2.414600641 | 1.15E-18 | 6.29E-17 | UP |
| Tox | 6.862395632 | 0.079280273 | 5.510907722 | 5.510907722 | 1.19E-18 | 6.50E-17 | UP |
| Bub1b | 2.312848081 | 0.045165095 | 4.774532542 | 4.774532542 | 1.23E-18 | 6.68E-17 | UP |
| Cldn8 | 3.356370604 | 0.018185118 | 6.490165495 | 6.490165495 | 1.40E-18 | 7.62E-17 | UP |
| Fstl1 | 39.05618426 | 3.573455184 | 2.574526057 | 2.574526057 | 1.42E-18 | 7.71E-17 | UP |
| AABR07044711.1 | 56.26659699 | 3.217029406 | 3.236765938 | 3.236765938 | 1.46E-18 | 7.89E-17 | UP |
| Col4a1 | 107.3534065 | 7.172926718 | 3.046658608 | 3.046658608 | 1.57E-18 | 8.47E-17 | UP |
| Rup2 | 329.7284429 | 4731.886399 | -4.595549308 | 4.595549308 | 1.62E-18 | 8.68E-17 | DOWN |
| Trim54 | 5.132735203 | 0 | 9.635821668 | 9.635821668 | 1.66E-18 | 8.87E-17 | UP |
| Gprc5b | 2.440006916 | 0.013641701 | 6.442993623 | 6.442993623 | 1.73E-18 | 9.20E-17 | UP |
| AABR07037307.1 | 0.05946168 | 7.826479808 | -7.564039448 | 7.564039448 | 1.83E-18 | 9.70E-17 | DOWN |
| AABR07031918.1 | 0.32682673 | 6.662325738 | -5.084128784 | 5.084128784 | 1.85E-18 | 9.82E-17 | DOWN |
| C8g | 23.81925722 | 175.4962954 | -3.635576081 | 3.635576081 | 1.89E-18 | 9.96E-17 | DOWN |
| St3gal2 | 12.0935109 | 1.009858551 | 2.736218853 | 2.736218853 | 1.93E-18 | 1.02E-16 | UP |
| Cdhr2 | 6.64017683 | 0.052278456 | 6.035434054 | 6.035434054 | 2.03E-18 | 1.06E-16 | UP |
| AC105531.1 | 9.690980971 | 30.52639867 | -2.478291795 | 2.478291795 | 2.06E-18 | 1.08E-16 | DOWN |
| Kirrel1 | 5.35571676 | 0.395854447 | 2.894964757 | 2.894964757 | 2.16E-18 | 1.13E-16 | UP |
| AC130970.1 | 11.94810958 | 0.094195617 | 6.002137297 | 6.002137297 | 2.21E-18 | 1.15E-16 | UP |
| Pdk4 | 54.48963722 | 261.2403072 | -3.045620337 | 3.045620337 | 2.34E-18 | 1.22E-16 | DOWN |
| Sulf1 | 4.981634346 | 0.122529035 | 4.417662241 | 4.417662241 | 2.37E-18 | 1.23E-16 | UP |
| Hacl1 | 28.05060546 | 134.6737334 | -3.083211776 | 3.083211776 | 2.50E-18 | 1.29E-16 | DOWN |
| Col1a1 | 141.4741431 | 1.774227651 | 5.410608125 | 5.410608125 | 2.85E-18 | 1.47E-16 | UP |
| Fbln2 | 5.895220102 | 0.289661874 | 3.466079905 | 3.466079905 | 2.89E-18 | 1.48E-16 | UP |
| Kcnn2 | 0.345681137 | 3.430400453 | -4.125475567 | 4.125475567 | 2.90E-18 | 1.49E-16 | DOWN |
| Krt20 | 17.88012655 | 0.249948177 | 5.235390281 | 5.235390281 | 3.09E-18 | 1.58E-16 | UP |
| Myo5a | 5.793239021 | 0.465082612 | 2.773341608 | 2.773341608 | 3.30E-18 | 1.68E-16 | UP |
| Plat | 33.1788553 | 1.055622621 | 4.069173842 | 4.069173842 | 3.36E-18 | 1.70E-16 | UP |
| Panx2 | 17.92252507 | 1.757946249 | 2.519680206 | 2.519680206 | 3.37E-18 | 1.71E-16 | UP |
| Slco1b2 | 35.42721032 | 256.8481292 | -3.627943946 | 3.627943946 | 3.39E-18 | 1.71E-16 | DOWN |
| Lgals2 | 48.6290372 | 1.209780593 | 4.427893167 | 4.427893167 | 3.64E-18 | 1.83E-16 | UP |
| Akr1b1 | 61.21997897 | 5.663388666 | 2.560916795 | 2.560916795 | 3.93E-18 | 1.97E-16 | UP |
| Gclm | 520.0558386 | 53.37052524 | 2.434563681 | 2.434563681 | 3.94E-18 | 1.97E-16 | UP |
| Papss1 | 11.44602247 | 0.945240643 | 2.726688061 | 2.726688061 | 3.98E-18 | 1.99E-16 | UP |
| Podxl | 11.08626546 | 1.074163816 | 2.515578107 | 2.515578107 | 4.02E-18 | 2.00E-16 | UP |
| Tspan17 | 3.457056257 | 0 | 9.429665016 | 9.429665016 | 4.24E-18 | 2.10E-16 | UP |
| Gck | 0.209724085 | 3.998194298 | -4.957338048 | 4.957338048 | 4.29E-18 | 2.13E-16 | DOWN |
| Mbl2 | 10.29327039 | 71.37432193 | -3.55475757 | 3.55475757 | 4.36E-18 | 2.16E-16 | DOWN |
| Trim35 | 21.50615711 | 2.211409153 | 2.410662274 | 2.410662274 | 4.42E-18 | 2.18E-16 | UP |
| Agtpbp1 | 13.93066035 | 1.042308952 | 2.858341796 | 2.858341796 | 4.45E-18 | 2.19E-16 | UP |
| Tuba1a | 103.4830753 | 10.07297435 | 2.488363833 | 2.488363833 | 4.60E-18 | 2.26E-16 | UP |
| Itga6 | 29.83240544 | 2.640816333 | 2.63048572 | 2.63048572 | 4.66E-18 | 2.28E-16 | UP |
| Ccnb1 | 13.75890887 | 0.457950344 | 4.013994577 | 4.013994577 | 4.78E-18 | 2.33E-16 | UP |
| Art4 | 10.49127699 | 0.472306116 | 3.616106823 | 3.616106823 | 5.05E-18 | 2.46E-16 | UP |
| Fzd1 | 7.809284829 | 21.1790898 | -2.280795605 | 2.280795605 | 5.80E-18 | 2.82E-16 | DOWN |
| Scara3 | 1.566939884 | 0 | 9.037792614 | 9.037792614 | 6.06E-18 | 2.94E-16 | UP |
| Lipc | 35.20476618 | 154.3401885 | -2.922284714 | 2.922284714 | 6.32E-18 | 3.05E-16 | DOWN |
| Pof1b | 3.072733506 | 0 | 10.15692495 | 10.15692495 | 6.44E-18 | 3.10E-16 | UP |
| Tbx4 | 2.666855456 | 0 | 9.695652495 | 9.695652495 | 6.98E-18 | 3.36E-16 | UP |
| Tpx2 | 6.333731449 | 0.300575189 | 3.532050234 | 3.532050234 | 7.55E-18 | 3.62E-16 | UP |
| Rab31 | 16.58869226 | 1.640434552 | 2.471074936 | 2.471074936 | 7.79E-18 | 3.73E-16 | UP |
| Igfbp5 | 9.875504012 | 0.346249007 | 4.064011761 | 4.064011761 | 8.46E-18 | 4.04E-16 | UP |
| Oat | 39.05108531 | 145.9021707 | -2.722372338 | 2.722372338 | 8.88E-18 | 4.23E-16 | DOWN |
| Tsku | 20.54926207 | 141.1188637 | -3.568249919 | 3.568249919 | 1.03E-17 | 4.89E-16 | DOWN |
| Slc44a3 | 7.651046044 | 0.225269285 | 4.196228074 | 4.196228074 | 1.04E-17 | 4.93E-16 | UP |
| Hap1 | 2.308119131 | 0.015226969 | 6.25878935 | 6.25878935 | 1.06E-17 | 5.00E-16 | UP |
| Prr11 | 2.952616107 | 0 | 9.113004125 | 9.113004125 | 1.38E-17 | 6.51E-16 | UP |
| Abcc1 | 6.196568176 | 0.499293968 | 2.775317382 | 2.775317382 | 1.41E-17 | 6.65E-16 | UP |
| Dyrk3 | 1.439010501 | 0 | 9.225049451 | 9.225049451 | 1.53E-17 | 7.20E-16 | UP |
| Kif20a | 5.961324966 | 0.301615999 | 3.437459425 | 3.437459425 | 1.64E-17 | 7.67E-16 | UP |
| Cldn1 | 17.75237972 | 53.88413825 | -2.427654832 | 2.427654832 | 1.65E-17 | 7.69E-16 | DOWN |
| Vgll3 | 5.817260828 | 0.016670687 | 7.214233723 | 7.214233723 | 1.65E-17 | 7.69E-16 | UP |
| Mcm6 | 10.00435651 | 0.624130626 | 3.13748942 | 3.13748942 | 1.69E-17 | 7.85E-16 | UP |
| Slc22a12 | 1.873531891 | 0.009443573 | 6.655512133 | 6.655512133 | 1.71E-17 | 7.94E-16 | UP |
| Wls | 16.97361693 | 1.449579046 | 2.67261217 | 2.67261217 | 1.79E-17 | 8.27E-16 | UP |
| Fxyd1 | 16.66257971 | 67.94998553 | -2.826948662 | 2.826948662 | 1.81E-17 | 8.33E-16 | DOWN |
| Iqgap3 | 2.632844976 | 0.088313304 | 4.016783939 | 4.016783939 | 2.01E-17 | 9.24E-16 | UP |
| Plp2 | 47.46367324 | 3.874619329 | 2.723712521 | 2.723712521 | 2.38E-17 | 1.09E-15 | UP |
| Adcy1 | 2.35182458 | 8.69614535 | -2.688798443 | 2.688798443 | 2.50E-17 | 1.14E-15 | DOWN |
| Cyp4a3 | 145.1652902 | 606.8042975 | -2.879347226 | 2.879347226 | 2.64E-17 | 1.20E-15 | DOWN |
| Tubb5 | 144.3902514 | 12.27858459 | 2.674358007 | 2.674358007 | 2.72E-17 | 1.24E-15 | UP |
| Igsf3 | 1.353689283 | 0.020676012 | 5.116492337 | 5.116492337 | 3.10E-17 | 1.41E-15 | UP |
| Lgals3bp | 139.6526651 | 12.35629427 | 2.652754091 | 2.652754091 | 3.13E-17 | 1.42E-15 | UP |
| B4galnt4 | 2.634015226 | 0.010094694 | 6.93843113 | 6.93843113 | 3.17E-17 | 1.43E-15 | UP |
| RGD1565355 | 38.05203429 | 3.599725992 | 2.571460854 | 2.571460854 | 3.20E-17 | 1.45E-15 | UP |
| Cpq | 49.08479115 | 138.3286787 | -2.335601739 | 2.335601739 | 3.36E-17 | 1.51E-15 | DOWN |
| Cyp3a18 | 19.44303205 | 235.573347 | -4.341514637 | 4.341514637 | 3.45E-17 | 1.55E-15 | DOWN |
| Filip1l | 14.52783242 | 1.369696019 | 2.545144298 | 2.545144298 | 3.45E-17 | 1.55E-15 | UP |
| Gpnmb | 139.1143166 | 9.957885609 | 2.910191371 | 2.910191371 | 3.58E-17 | 1.60E-15 | UP |
| Plk1 | 5.376268756 | 0.073527688 | 5.262427837 | 5.262427837 | 3.63E-17 | 1.62E-15 | UP |
| Plxna3 | 2.458314917 | 0.141910256 | 3.269393751 | 3.269393751 | 3.73E-17 | 1.66E-15 | UP |
| Tmc5 | 3.113237087 | 0 | 10.23734338 | 10.23734338 | 3.94E-17 | 1.75E-15 | UP |
| Pygb | 10.5474389 | 0.882331298 | 2.716426532 | 2.716426532 | 4.06E-17 | 1.80E-15 | UP |
| Ppp1r3b | 4.544656281 | 25.76098522 | -3.262332084 | 3.262332084 | 4.23E-17 | 1.87E-15 | DOWN |
| Arhgef28 | 2.35462662 | 0.062999497 | 4.35031059 | 4.35031059 | 4.47E-17 | 1.97E-15 | UP |
| Ccdc88c | 1.882493889 | 0.053853814 | 4.24691759 | 4.24691759 | 4.61E-17 | 2.03E-15 | UP |
| Fjx1 | 4.092462012 | 0.029051456 | 6.136831654 | 6.136831654 | 4.78E-17 | 2.10E-15 | UP |
| Lhx8 | 0.01198715 | 2.08106095 | -7.732597157 | 7.732597157 | 5.34E-17 | 2.34E-15 | DOWN |
| Prodh1 | 2.157661202 | 18.02341334 | -3.804556898 | 3.804556898 | 5.68E-17 | 2.48E-15 | DOWN |
| Ptgr1 | 442.1236524 | 35.03290886 | 2.782626439 | 2.782626439 | 6.07E-17 | 2.65E-15 | UP |
| Noct | 1.954653497 | 13.99318358 | -3.714874823 | 3.714874823 | 6.26E-17 | 2.72E-15 | DOWN |
| Ncaph | 3.199701228 | 0.057630877 | 4.874779743 | 4.874779743 | 6.55E-17 | 2.84E-15 | UP |
| Inhbc | 7.525565066 | 54.07854349 | -3.594278435 | 3.594278435 | 6.64E-17 | 2.88E-15 | DOWN |
| Aldh1a2 | 8.672701911 | 0.180656744 | 4.699171802 | 4.699171802 | 7.12E-17 | 3.08E-15 | UP |
| Plscr1 | 23.23530059 | 1.712282214 | 2.860111459 | 2.860111459 | 7.34E-17 | 3.17E-15 | UP |
| Fads2 | 40.16981091 | 208.2671411 | -3.209905637 | 3.209905637 | 7.74E-17 | 3.33E-15 | DOWN |
| Fabp4 | 37.55224059 | 1.070345679 | 4.32193462 | 4.32193462 | 8.12E-17 | 3.49E-15 | UP |
| Zbtb16 | 2.38193492 | 12.83744533 | -3.260695213 | 3.260695213 | 8.23E-17 | 3.53E-15 | DOWN |
| Rasgef1b | 6.019341658 | 18.09008147 | -2.422449384 | 2.422449384 | 8.57E-17 | 3.66E-15 | DOWN |
| Ifnlr1 | 1.723601615 | 0.005173392 | 7.145058095 | 7.145058095 | 8.84E-17 | 3.77E-15 | UP |
| Bcl6 | 4.594919134 | 18.9924484 | -2.932968211 | 2.932968211 | 8.87E-17 | 3.78E-15 | DOWN |
| Cpz | 23.33446797 | 1.774820938 | 2.877957365 | 2.877957365 | 9.60E-17 | 4.08E-15 | UP |
| Adtrp | 57.17867225 | 191.2482097 | -2.557958322 | 2.557958322 | 1.05E-16 | 4.46E-15 | DOWN |
| G6pc | 61.92267484 | 320.1663698 | -3.165762332 | 3.165762332 | 1.12E-16 | 4.72E-15 | DOWN |
| Cx3cr1 | 10.17612108 | 0.369971568 | 3.918071191 | 3.918071191 | 1.14E-16 | 4.79E-15 | UP |
| Apoa4 | 57.29811506 | 693.3773817 | -4.325012537 | 4.325012537 | 1.22E-16 | 5.14E-15 | DOWN |
| Kif11 | 5.196315979 | 0.342166218 | 3.051125002 | 3.051125002 | 1.27E-16 | 5.34E-15 | UP |
| Tm7sf2 | 11.57900995 | 53.08713186 | -2.988408424 | 2.988408424 | 1.49E-16 | 6.26E-15 | DOWN |
| Akr1c12 | 139.359981 | 428.0477038 | -2.444691578 | 2.444691578 | 1.50E-16 | 6.29E-15 | DOWN |
| Gpx1 | 324.4063944 | 1068.081565 | -2.591142151 | 2.591142151 | 1.53E-16 | 6.40E-15 | DOWN |
| Rflnb | 12.58970237 | 0.211114845 | 4.960940032 | 4.960940032 | 1.55E-16 | 6.44E-15 | UP |
| Lgals3 | 61.54196052 | 3.930157752 | 3.084597313 | 3.084597313 | 1.65E-16 | 6.83E-15 | UP |
| Ect2 | 3.621326292 | 0.096022233 | 4.343764634 | 4.343764634 | 1.65E-16 | 6.83E-15 | UP |
| Gpld1 | 20.40787356 | 92.20491341 | -2.953772166 | 2.953772166 | 1.67E-16 | 6.90E-15 | DOWN |
| Fabp1 | 142.456571 | 1748.407146 | -4.333725915 | 4.333725915 | 1.84E-16 | 7.60E-15 | DOWN |
| Itga3 | 10.65925942 | 0.543400213 | 3.388806336 | 3.388806336 | 1.95E-16 | 8.02E-15 | UP |
| Avpr1a | 0.574043708 | 30.21101588 | -6.401051144 | 6.401051144 | 1.98E-16 | 8.13E-15 | DOWN |
| Efs | 1.447790074 | 0.009476578 | 6.206824797 | 6.206824797 | 2.22E-16 | 9.07E-15 | UP |
| Angptl4 | 33.88727502 | 141.630745 | -2.867791562 | 2.867791562 | 2.28E-16 | 9.32E-15 | DOWN |
| Krt8 | 468.1293433 | 49.71175966 | 2.369251984 | 2.369251984 | 2.30E-16 | 9.39E-15 | UP |
| Angptl6 | 2.145943335 | 12.39773731 | -3.356637064 | 3.356637064 | 2.36E-16 | 9.60E-15 | DOWN |
| Cdca7 | 5.89193713 | 0.254569378 | 3.662697107 | 3.662697107 | 2.42E-16 | 9.81E-15 | UP |
| Mlph | 2.873668502 | 0.012347756 | 6.789421702 | 6.789421702 | 2.45E-16 | 9.94E-15 | UP |
| Htra3 | 12.85034853 | 0.225808931 | 4.954538704 | 4.954538704 | 2.58E-16 | 1.04E-14 | UP |
| Jag1 | 5.965655897 | 0.540612842 | 2.634871769 | 2.634871769 | 2.59E-16 | 1.05E-14 | UP |
| B4galt5 | 21.17799106 | 2.040410399 | 2.499928173 | 2.499928173 | 2.64E-16 | 1.06E-14 | UP |
| Col4a5 | 12.19467642 | 1.577960976 | 2.094749702 | 2.094749702 | 2.73E-16 | 1.10E-14 | UP |
| Sema3e | 2.097488229 | 0.010936021 | 6.560165553 | 6.560165553 | 3.02E-16 | 1.21E-14 | UP |
| Dab1 | 0.7998725 | 3.384898401 | -2.95596125 | 2.95596125 | 3.05E-16 | 1.22E-14 | DOWN |
| Ppp1r1b | 6.903608394 | 0.165226978 | 4.521772121 | 4.521772121 | 3.18E-16 | 1.27E-14 | UP |
| Mtmr7 | 0.520039853 | 5.595281252 | -4.233930537 | 4.233930537 | 3.21E-16 | 1.28E-14 | DOWN |
| AABR07061950.1 | 76.02061182 | 334.2307475 | -2.936791673 | 2.936791673 | 3.43E-16 | 1.36E-14 | DOWN |
| Dram1 | 10.90636929 | 0.574133988 | 3.386718243 | 3.386718243 | 3.45E-16 | 1.37E-14 | UP |
| Adamts15 | 2.536624304 | 0.104778511 | 3.746362196 | 3.746362196 | 3.54E-16 | 1.40E-14 | UP |
| Hid1 | 16.36429721 | 1.506372325 | 2.609501929 | 2.609501929 | 3.59E-16 | 1.42E-14 | UP |
| Fat1 | 21.51786082 | 2.551546351 | 2.214873807 | 2.214873807 | 3.76E-16 | 1.48E-14 | UP |
| AABR07059663.1 | 9.010180046 | 0.943433096 | 2.394890982 | 2.394890982 | 3.82E-16 | 1.50E-14 | UP |
| LOC100365958 | 13.10926015 | 50.70762392 | -2.769026111 | 2.769026111 | 3.99E-16 | 1.56E-14 | DOWN |
| Rab25 | 12.40413669 | 0.13402342 | 5.618794066 | 5.618794066 | 4.03E-16 | 1.58E-14 | UP |
| Ptk7 | 4.356347434 | 0.113730908 | 4.363444224 | 4.363444224 | 4.08E-16 | 1.59E-14 | UP |
| Gca | 12.16813156 | 0.819522289 | 3.02282198 | 3.02282198 | 4.13E-16 | 1.61E-14 | UP |
| Frem1 | 2.044303199 | 0.108292002 | 3.382137051 | 3.382137051 | 4.16E-16 | 1.62E-14 | UP |
| Fbln1 | 15.08704526 | 0.494256837 | 4.077704725 | 4.077704725 | 4.18E-16 | 1.62E-14 | UP |
| Rtn4 | 24.84492692 | 2.63370759 | 2.366581854 | 2.366581854 | 4.24E-16 | 1.64E-14 | UP |
| Mxra8 | 17.1479835 | 1.551289809 | 2.597109909 | 2.597109909 | 4.63E-16 | 1.79E-14 | UP |
| Rasef | 3.303296315 | 0.016931155 | 6.556907454 | 6.556907454 | 4.93E-16 | 1.90E-14 | UP |
| Shroom3 | 11.10455812 | 1.075122343 | 2.496706485 | 2.496706485 | 4.94E-16 | 1.90E-14 | UP |
| Vtcn1 | 1.341811895 | 0 | 9.202438047 | 9.202438047 | 4.97E-16 | 1.91E-14 | UP |
| Klf15 | 12.48482874 | 53.91880321 | -2.896297554 | 2.896297554 | 5.42E-16 | 2.07E-14 | DOWN |
| Fkbp10 | 5.130325765 | 0.27142204 | 3.371636715 | 3.371636715 | 5.69E-16 | 2.17E-14 | UP |
| Clec10a | 15.06950838 | 0.870943454 | 3.21091574 | 3.21091574 | 6.01E-16 | 2.29E-14 | UP |
| Lrrc8e | 2.256900199 | 0.02045904 | 5.801966819 | 5.801966819 | 6.16E-16 | 2.34E-14 | UP |
| Slc5a1 | 9.905027006 | 0.36057262 | 3.901282778 | 3.901282778 | 6.53E-16 | 2.47E-14 | UP |
| Adamtsl2 | 32.73276534 | 2.86825321 | 2.675840011 | 2.675840011 | 6.52E-16 | 2.47E-14 | UP |
| Cyp3a23/3a1 | 4.09577722 | 545.1839657 | -7.726247121 | 7.726247121 | 6.59E-16 | 2.49E-14 | DOWN |
| Sema6a | 22.36908525 | 2.100787753 | 2.561607357 | 2.561607357 | 7.09E-16 | 2.68E-14 | UP |
| Kif20b | 2.53111213 | 0.082412945 | 4.060826177 | 4.060826177 | 7.15E-16 | 2.69E-14 | UP |
| Map2 | 1.283479056 | 0.040763343 | 4.091578968 | 4.091578968 | 7.40E-16 | 2.78E-14 | UP |
| Prss22 | 10.00262734 | 0 | 10.21719618 | 10.21719618 | 7.49E-16 | 2.81E-14 | UP |
| LOC685716 | 5.571609009 | 0.13183901 | 4.494530717 | 4.494530717 | 7.76E-16 | 2.91E-14 | UP |
| Pdk3 | 6.781170501 | 0.458063719 | 3.019474389 | 3.019474389 | 8.02E-16 | 3.00E-14 | UP |
| Mcpt8l2 | 10.02099754 | 0.18381169 | 4.897110707 | 4.897110707 | 8.31E-16 | 3.10E-14 | UP |
| Smlr1 | 11.87666655 | 55.74373533 | -2.996431756 | 2.996431756 | 8.40E-16 | 3.13E-14 | DOWN |
| Slc13a3 | 2.097137808 | 8.672020824 | -2.864220929 | 2.864220929 | 9.56E-16 | 3.55E-14 | DOWN |
| Pxmp4 | 27.47924126 | 94.36639023 | -2.579717544 | 2.579717544 | 1.06E-15 | 3.93E-14 | DOWN |
| Ptpn13 | 6.607657305 | 0.709049755 | 2.375082621 | 2.375082621 | 1.06E-15 | 3.94E-14 | UP |
| Gmds | 12.96498326 | 0.349097814 | 4.281582105 | 4.281582105 | 1.09E-15 | 4.03E-14 | UP |
| AABR07028352.1 | 3.944088604 | 0.119448267 | 4.148996604 | 4.148996604 | 1.12E-15 | 4.14E-14 | UP |
| Pla1a | 22.95767986 | 2.415158414 | 2.415778992 | 2.415778992 | 1.13E-15 | 4.17E-14 | UP |
| Aurkb | 2.263865432 | 0.093869886 | 3.711267996 | 3.711267996 | 1.14E-15 | 4.19E-14 | UP |
| Timp1 | 47.8892539 | 3.316653998 | 2.955645927 | 2.955645927 | 1.19E-15 | 4.35E-14 | UP |
| Pkm | 22.12485391 | 1.327764461 | 3.161911091 | 3.161911091 | 1.19E-15 | 4.35E-14 | UP |
| Gja1 | 54.27835417 | 4.946931286 | 2.59327917 | 2.59327917 | 1.21E-15 | 4.41E-14 | UP |
| Tfcp2l1 | 5.424272018 | 0.447799105 | 2.724177295 | 2.724177295 | 1.26E-15 | 4.59E-14 | UP |
| Kif22 | 4.246905221 | 0.138739549 | 4.068188781 | 4.068188781 | 1.27E-15 | 4.63E-14 | UP |
| Pdzk1ip1 | 10.48463371 | 0.106190694 | 5.693825836 | 5.693825836 | 1.31E-15 | 4.77E-14 | UP |
| Agxt | 13.24439617 | 86.76742445 | -3.456299653 | 3.456299653 | 1.39E-15 | 5.02E-14 | DOWN |
| Pdgfrb | 16.47238961 | 1.755699458 | 2.378532724 | 2.378532724 | 1.53E-15 | 5.51E-14 | UP |
| Vil1 | 7.411289615 | 0.055598206 | 6.103058492 | 6.103058492 | 1.59E-15 | 5.75E-14 | UP |
| Slco1a1 | 31.42189041 | 176.4740125 | -3.263419036 | 3.263419036 | 1.60E-15 | 5.76E-14 | DOWN |
| Pmp22 | 14.37748637 | 0.579581647 | 3.742445582 | 3.742445582 | 1.71E-15 | 6.16E-14 | UP |
| Cblc | 6.808099646 | 0.065725906 | 5.72969545 | 5.72969545 | 2.00E-15 | 7.18E-14 | UP |
| Sfn | 13.46773735 | 0.218666213 | 5.028443489 | 5.028443489 | 2.25E-15 | 8.06E-14 | UP |
| Itgax | 5.532324585 | 0.241475686 | 3.676892215 | 3.676892215 | 2.29E-15 | 8.17E-14 | UP |
| Eci1 | 90.95363817 | 324.4318192 | -2.640055661 | 2.640055661 | 2.33E-15 | 8.29E-14 | DOWN |
| Hmmr | 5.4254648 | 0.198088674 | 3.898789909 | 3.898789909 | 2.35E-15 | 8.35E-14 | UP |
| Ces2 | 16.04222879 | 1.247384229 | 2.884690254 | 2.884690254 | 2.58E-15 | 9.15E-14 | UP |
| Rbm3 | 41.63083281 | 4.043255293 | 2.474953053 | 2.474953053 | 2.65E-15 | 9.36E-14 | UP |
| Ncapg | 4.057968265 | 0.158436352 | 3.802988345 | 3.802988345 | 2.65E-15 | 9.37E-14 | UP |
| Lad1 | 10.41130995 | 0.213855063 | 4.70505125 | 4.70505125 | 2.72E-15 | 9.58E-14 | UP |
| Adgrg2 | 6.691716521 | 0.151801451 | 4.639314984 | 4.639314984 | 2.78E-15 | 9.75E-14 | UP |
| Itgb4 | 8.657841861 | 0.321788235 | 3.859927987 | 3.859927987 | 2.83E-15 | 9.93E-14 | UP |
| Lrp3 | 8.624300675 | 31.7975057 | -2.665163921 | 2.665163921 | 2.84E-15 | 9.95E-14 | DOWN |
| Hadh | 71.36891551 | 181.9182431 | -2.18221254 | 2.18221254 | 2.89E-15 | 1.01E-13 | DOWN |
| Irx1 | 0.227447284 | 2.850462043 | -4.386958343 | 4.386958343 | 2.96E-15 | 1.03E-13 | DOWN |
| Ptk2b | 15.75243527 | 1.787290984 | 2.269356147 | 2.269356147 | 2.98E-15 | 1.04E-13 | UP |
| Itgb8 | 3.274997347 | 0.126408736 | 3.840200235 | 3.840200235 | 3.05E-15 | 1.06E-13 | UP |
| Flrt2 | 4.527716245 | 0.182922876 | 3.760508432 | 3.760508432 | 3.10E-15 | 1.07E-13 | UP |
| Abhd2 | 46.13699553 | 131.634729 | -2.334667321 | 2.334667321 | 3.24E-15 | 1.12E-13 | DOWN |
| Col4a2 | 44.89909458 | 3.807315497 | 2.714052308 | 2.714052308 | 3.30E-15 | 1.14E-13 | UP |
| Slc16a2 | 13.76599403 | 51.99933335 | -2.698896935 | 2.698896935 | 3.36E-15 | 1.16E-13 | DOWN |
| Tm6sf1 | 7.881513245 | 0.570770457 | 2.922072621 | 2.922072621 | 3.51E-15 | 1.21E-13 | UP |
| Insc | 0.894682723 | 6.494533699 | -3.617514655 | 3.617514655 | 3.54E-15 | 1.22E-13 | DOWN |
| Ctgf | 46.56353038 | 3.134470189 | 2.99684373 | 2.99684373 | 3.64E-15 | 1.25E-13 | UP |
| F2rl1 | 5.211546916 | 0.020481003 | 6.933907478 | 6.933907478 | 3.73E-15 | 1.28E-13 | UP |
| Mug2 | 32.84701323 | 152.7268967 | -3.007589116 | 3.007589116 | 4.24E-15 | 1.45E-13 | DOWN |
| Ttc39a | 3.921463094 | 0.114742753 | 4.185738796 | 4.185738796 | 4.33E-15 | 1.48E-13 | UP |
| AABR07069219.1 | 9.815096744 | 0.370479383 | 3.825611898 | 3.825611898 | 4.40E-15 | 1.50E-13 | UP |
| Grem1 | 2.20069989 | 0.021327351 | 5.703187554 | 5.703187554 | 4.40E-15 | 1.50E-13 | UP |
| Acta2 | 59.82393706 | 2.055269738 | 3.959161403 | 3.959161403 | 4.55E-15 | 1.54E-13 | UP |
| Cyp2c24 | 21.47929452 | 0.896904234 | 3.692445727 | 3.692445727 | 4.68E-15 | 1.59E-13 | UP |
| Prrg4 | 14.43852353 | 1.261443215 | 2.642632567 | 2.642632567 | 4.89E-15 | 1.65E-13 | UP |
| Pfkp | 12.83643213 | 0.581515257 | 3.57798596 | 3.57798596 | 4.95E-15 | 1.67E-13 | UP |
| Diaph3 | 2.460194791 | 0.025257058 | 5.672514762 | 5.672514762 | 4.96E-15 | 1.67E-13 | UP |
| Srsf12 | 5.076279881 | 0.217388279 | 3.657792395 | 3.657792395 | 5.01E-15 | 1.69E-13 | UP |
| Entpd1 | 10.88330319 | 1.148618982 | 2.364739459 | 2.364739459 | 5.56E-15 | 1.87E-13 | UP |
| Rgs5 | 52.39868953 | 4.762772487 | 2.587928845 | 2.587928845 | 5.70E-15 | 1.91E-13 | UP |
| Ace | 4.066131452 | 0.146441536 | 3.895554851 | 3.895554851 | 5.82E-15 | 1.95E-13 | UP |
| Gcnt3 | 20.92189642 | 0.209404105 | 5.715104647 | 5.715104647 | 5.92E-15 | 1.98E-13 | UP |
| Klhdc8a | 0.299375017 | 2.816822596 | -4.007192726 | 4.007192726 | 7.00E-15 | 2.34E-13 | DOWN |
| Cacna2d1 | 2.374786719 | 0.087943055 | 3.869335113 | 3.869335113 | 7.32E-15 | 2.44E-13 | UP |
| Ccl20 | 5.855709129 | 0 | 8.817215165 | 8.817215165 | 7.37E-15 | 2.45E-13 | UP |
| Steap2 | 3.022489381 | 0.100221181 | 4.012318674 | 4.012318674 | 7.67E-15 | 2.54E-13 | UP |
| Thbs1 | 22.84053938 | 1.896665752 | 2.725441486 | 2.725441486 | 7.82E-15 | 2.59E-13 | UP |
| AABR07069878.1 | 0.953810189 | 36.80604365 | -5.907919739 | 5.907919739 | 8.11E-15 | 2.68E-13 | DOWN |
| Rapgef4 | 6.69008622 | 22.24200784 | -2.521223787 | 2.521223787 | 8.40E-15 | 2.77E-13 | DOWN |
| Eps8l3 | 3.92199241 | 0 | 9.963711433 | 9.963711433 | 8.42E-15 | 2.77E-13 | UP |
| Fermt1 | 2.516265344 | 0.016266228 | 6.285932679 | 6.285932679 | 9.07E-15 | 2.98E-13 | UP |
| Boc | 2.310631922 | 0.04427793 | 4.779944772 | 4.779944772 | 9.77E-15 | 3.21E-13 | UP |
| Nov | 9.217149204 | 0.234278476 | 4.381399825 | 4.381399825 | 9.89E-15 | 3.24E-13 | UP |
| Vcan | 1.897802833 | 0.010425095 | 6.499057852 | 6.499057852 | 1.02E-14 | 3.33E-13 | UP |
| Sat2 | 0.593681 | 4.678652498 | -3.802561357 | 3.802561357 | 1.05E-14 | 3.42E-13 | DOWN |
| Pxdn | 16.36708961 | 1.768945301 | 2.350717297 | 2.350717297 | 1.06E-14 | 3.45E-13 | UP |
| Fbln5 | 25.62707616 | 2.936916846 | 2.242599243 | 2.242599243 | 1.11E-14 | 3.63E-13 | UP |
| Vwa1 | 9.024220427 | 0.430377465 | 3.527008678 | 3.527008678 | 1.15E-14 | 3.72E-13 | UP |
| As3mt | 48.63527496 | 130.7153216 | -2.23857379 | 2.23857379 | 1.15E-14 | 3.72E-13 | DOWN |
| AABR07073181.1 | 15.89642641 | 1.791902763 | 2.322271808 | 2.322271808 | 1.29E-14 | 4.17E-13 | UP |
| Ptgs2 | 1.891773538 | 0.023281758 | 5.368386453 | 5.368386453 | 1.30E-14 | 4.20E-13 | UP |
| Mcam | 14.69832645 | 1.304398314 | 2.66674991 | 2.66674991 | 1.34E-14 | 4.32E-13 | UP |
| Ckb | 82.62843393 | 7.900896898 | 2.513391667 | 2.513391667 | 1.41E-14 | 4.55E-13 | UP |
| Cdo1 | 135.3782395 | 904.9993528 | -3.505867076 | 3.505867076 | 1.43E-14 | 4.58E-13 | DOWN |
| Kif1a | 1.761964029 | 0.021334198 | 5.426492268 | 5.426492268 | 1.43E-14 | 4.58E-13 | UP |
| Gjb1 | 80.51157035 | 269.6045209 | -2.535860435 | 2.535860435 | 1.43E-14 | 4.59E-13 | DOWN |
| Tp53inp2 | 29.9452486 | 91.01413068 | -2.405829171 | 2.405829171 | 1.45E-14 | 4.64E-13 | DOWN |
| Itga2 | 2.551121081 | 0.015963132 | 6.27333696 | 6.27333696 | 1.58E-14 | 5.05E-13 | UP |
| NEWGENE_1308171 | 59.14452671 | 4.501265484 | 2.804802464 | 2.804802464 | 1.58E-14 | 5.05E-13 | UP |
| LOC100912026 | 0.075747727 | 4.37683522 | -6.301418616 | 6.301418616 | 1.62E-14 | 5.18E-13 | DOWN |
| Ttk | 2.250748933 | 0.049144824 | 4.63248029 | 4.63248029 | 1.63E-14 | 5.20E-13 | UP |
| Anln | 2.60868163 | 0.041283681 | 5.037829509 | 5.037829509 | 1.66E-14 | 5.26E-13 | UP |
| Itga11 | 1.182276841 | 0.004396903 | 6.833722938 | 6.833722938 | 1.67E-14 | 5.30E-13 | UP |
| Dynlt1 | 23.50028291 | 0.758208625 | 4.070695679 | 4.070695679 | 1.79E-14 | 5.67E-13 | UP |
| Dync2h1 | 2.282361492 | 0.249149458 | 2.347500899 | 2.347500899 | 1.80E-14 | 5.68E-13 | UP |
| Rps6ka3 | 15.60273858 | 1.757291504 | 2.264639852 | 2.264639852 | 1.94E-14 | 6.13E-13 | UP |
| Dusp1 | 31.96382893 | 146.9694905 | -3.061270001 | 3.061270001 | 1.96E-14 | 6.19E-13 | DOWN |
| Pcolce | 21.96222182 | 1.857400933 | 2.73223542 | 2.73223542 | 1.98E-14 | 6.23E-13 | UP |
| Fam19a4 | 1.864885096 | 0 | 8.612406812 | 8.612406812 | 2.12E-14 | 6.67E-13 | UP |
| Il1rl1 | 3.045619875 | 0.055284064 | 4.864709648 | 4.864709648 | 2.14E-14 | 6.72E-13 | UP |
| Ms4a18 | 5.13238037 | 0 | 8.97870149 | 8.97870149 | 2.21E-14 | 6.92E-13 | UP |
| Adamts8 | 2.271638392 | 0.013438997 | 6.315639993 | 6.315639993 | 2.28E-14 | 7.13E-13 | UP |
| Tmem86b | 11.12753481 | 71.07486508 | -3.421892207 | 3.421892207 | 2.47E-14 | 7.71E-13 | DOWN |
| Bex4 | 14.21526495 | 0.115862217 | 5.93988149 | 5.93988149 | 2.58E-14 | 8.03E-13 | UP |
| Abcc4 | 14.1591215 | 1.179892131 | 2.683425177 | 2.683425177 | 2.60E-14 | 8.06E-13 | UP |
| Apoa5 | 156.748319 | 664.7234368 | -2.860739617 | 2.860739617 | 2.61E-14 | 8.10E-13 | DOWN |
| Esrp1 | 4.543664897 | 0.029439873 | 6.29411689 | 6.29411689 | 2.62E-14 | 8.12E-13 | UP |
| Adhfe1 | 22.17912848 | 109.2644585 | -3.067365085 | 3.067365085 | 2.70E-14 | 8.36E-13 | DOWN |
| Insig2 | 87.6488469 | 290.3455345 | -2.520835223 | 2.520835223 | 2.75E-14 | 8.49E-13 | DOWN |
| Eln | 12.20605604 | 0.563926655 | 3.596054285 | 3.596054285 | 2.80E-14 | 8.62E-13 | UP |
| Rin1 | 1.499335689 | 0.024953117 | 4.984318011 | 4.984318011 | 2.84E-14 | 8.70E-13 | UP |
| Klf9 | 12.63878351 | 37.13973547 | -2.35512901 | 2.35512901 | 2.92E-14 | 8.95E-13 | DOWN |
| Emp2 | 8.297511633 | 0.791893221 | 2.523081673 | 2.523081673 | 3.13E-14 | 9.56E-13 | UP |
| Mcm5 | 4.57747475 | 0.262183323 | 3.256609989 | 3.256609989 | 3.16E-14 | 9.63E-13 | UP |
| Arhgap28 | 2.11657722 | 0.046515251 | 4.593237251 | 4.593237251 | 3.18E-14 | 9.67E-13 | UP |
| Lipo1 | 1.222845079 | 9.844448258 | -3.777124275 | 3.777124275 | 3.25E-14 | 9.88E-13 | DOWN |
| Smo | 5.67007434 | 0.458927638 | 2.77233503 | 2.77233503 | 3.27E-14 | 9.92E-13 | UP |
| S100a8 | 18.63590707 | 0.327288512 | 4.905916416 | 4.905916416 | 3.51E-14 | 1.06E-12 | UP |
| Ncam1 | 1.916982041 | 0.017590981 | 5.789122559 | 5.789122559 | 3.58E-14 | 1.08E-12 | UP |
| Abhd1 | 4.4142119 | 13.44588621 | -2.414449148 | 2.414449148 | 3.71E-14 | 1.12E-12 | DOWN |
| LOC102547056 | 1.881165966 | 0.097325586 | 3.421057858 | 3.421057858 | 3.77E-14 | 1.14E-12 | UP |
| Sult1e1 | 7.335427523 | 920.2926308 | -7.709877138 | 7.709877138 | 3.93E-14 | 1.18E-12 | DOWN |
| Id4 | 13.00486814 | 37.86143629 | -2.351667404 | 2.351667404 | 3.93E-14 | 1.18E-12 | DOWN |
| Mcm3 | 7.245613111 | 0.489634431 | 3.035730338 | 3.035730338 | 4.06E-14 | 1.22E-12 | UP |
| Fscn1 | 13.43592657 | 1.052781501 | 2.837777839 | 2.837777839 | 4.44E-14 | 1.33E-12 | UP |
| Ttc9 | 2.233053 | 0.098548954 | 3.646831912 | 3.646831912 | 4.81E-14 | 1.44E-12 | UP |
| Aqp5 | 9.678551157 | 0.47586557 | 3.518534823 | 3.518534823 | 5.04E-14 | 1.50E-12 | UP |
| Ankrd13b | 6.142330468 | 0.345008101 | 3.285105378 | 3.285105378 | 5.10E-14 | 1.52E-12 | UP |
| Cd200 | 7.69899878 | 0.317498814 | 3.728923105 | 3.728923105 | 5.51E-14 | 1.64E-12 | UP |
| Dpep1 | 10.80023439 | 0.94768914 | 2.67626565 | 2.67626565 | 5.54E-14 | 1.64E-12 | UP |
| Tagln2 | 185.4044952 | 20.64604337 | 2.304204313 | 2.304204313 | 6.36E-14 | 1.88E-12 | UP |
| Postn | 3.651516615 | 0 | 10.4467828 | 10.4467828 | 6.36E-14 | 1.88E-12 | UP |
| Pmepa1 | 22.80710422 | 1.381583141 | 3.145567721 | 3.145567721 | 6.39E-14 | 1.89E-12 | UP |
| Timp2 | 88.62196067 | 10.76801421 | 2.180217957 | 2.180217957 | 6.44E-14 | 1.90E-12 | UP |
| Eps8l1 | 1.273072643 | 0 | 8.916475003 | 8.916475003 | 6.90E-14 | 2.03E-12 | UP |
| Trpc6 | 1.49559406 | 0.021924327 | 5.130602144 | 5.130602144 | 7.03E-14 | 2.06E-12 | UP |
| F2r | 22.14517456 | 2.048520167 | 2.541272411 | 2.541272411 | 7.11E-14 | 2.08E-12 | UP |
| C1qtnf6 | 4.187757114 | 0.242214741 | 3.25107752 | 3.25107752 | 7.31E-14 | 2.14E-12 | UP |
| Fmnl3 | 9.887799887 | 1.143803187 | 2.257117866 | 2.257117866 | 8.64E-14 | 2.52E-12 | UP |
| Tspan18 | 6.080237233 | 0.424234743 | 3.021015056 | 3.021015056 | 8.73E-14 | 2.55E-12 | UP |
| Cyfip2 | 3.227610449 | 0.371251831 | 2.254703997 | 2.254703997 | 8.91E-14 | 2.59E-12 | UP |
| Polg2 | 3.952033295 | 11.59533543 | -2.358395198 | 2.358395198 | 9.05E-14 | 2.63E-12 | DOWN |
| Gdf6 | 1.324676928 | 0.01168935 | 5.78604354 | 5.78604354 | 9.10E-14 | 2.64E-12 | UP |
| Gstm3 | 15.35087966 | 0.898428671 | 3.216895051 | 3.216895051 | 9.44E-14 | 2.74E-12 | UP |
| Grin2c | 1.498459776 | 0.00885903 | 6.473662861 | 6.473662861 | 9.56E-14 | 2.77E-12 | UP |
| Pcp4 | 5.60236859 | 0 | 8.497802719 | 8.497802719 | 9.86E-14 | 2.85E-12 | UP |
| Rnf8 | 5.528236866 | 13.87484557 | -2.155732887 | 2.155732887 | 1.01E-13 | 2.90E-12 | DOWN |
| Cdkn2b | 7.305433162 | 0.142108 | 4.716153451 | 4.716153451 | 1.05E-13 | 3.02E-12 | UP |
| LOC102551539 | 9.619180934 | 26.86497021 | -2.304983091 | 2.304983091 | 1.06E-13 | 3.05E-12 | DOWN |
| Tpm4 | 104.0679373 | 12.39387406 | 2.20551929 | 2.20551929 | 1.08E-13 | 3.09E-12 | UP |
| Rtn1 | 4.174585106 | 0.201902835 | 3.49316629 | 3.49316629 | 1.09E-13 | 3.12E-12 | UP |
| Ghr | 45.24109786 | 146.3802382 | -2.50046035 | 2.50046035 | 1.11E-13 | 3.17E-12 | DOWN |
| Ugdh | 333.8729686 | 43.82291738 | 2.082433296 | 2.082433296 | 1.11E-13 | 3.17E-12 | UP |
| Mgat3 | 5.898733973 | 0.145147648 | 4.428013935 | 4.428013935 | 1.12E-13 | 3.20E-12 | UP |
| Ptger3 | 4.359473421 | 16.0066368 | -2.643344223 | 2.643344223 | 1.18E-13 | 3.34E-12 | DOWN |
| Fcrl2 | 6.983188305 | 0.080394124 | 5.491504389 | 5.491504389 | 1.23E-13 | 3.48E-12 | UP |
| Pde5a | 6.381554963 | 0.483672367 | 2.866665491 | 2.866665491 | 1.23E-13 | 3.49E-12 | UP |
| Adam19 | 5.62674169 | 0.48556892 | 2.682056723 | 2.682056723 | 1.25E-13 | 3.54E-12 | UP |
| Fxyd6 | 9.464178478 | 0.788542122 | 2.752924378 | 2.752924378 | 1.26E-13 | 3.56E-12 | UP |
| Cyp2a3 | 27.90255091 | 91.16647226 | -2.499599996 | 2.499599996 | 1.32E-13 | 3.73E-12 | DOWN |
| Igsf1 | 2.295778159 | 0.03733095 | 5.060236846 | 5.060236846 | 1.37E-13 | 3.86E-12 | UP |
| Afap1 | 2.682678385 | 0.23593493 | 2.643451606 | 2.643451606 | 1.38E-13 | 3.90E-12 | UP |
| Angpt1 | 8.535847287 | 0.681471964 | 2.782259638 | 2.782259638 | 1.43E-13 | 4.02E-12 | UP |
| Sdc4 | 82.66613805 | 204.1749982 | -2.14268216 | 2.14268216 | 1.65E-13 | 4.64E-12 | DOWN |
| Il2rg | 6.138220959 | 0.514545173 | 2.698636219 | 2.698636219 | 1.69E-13 | 4.74E-12 | UP |
| AABR07006691.1 | 1.608583511 | 10.63941293 | -3.478022765 | 3.478022765 | 1.73E-13 | 4.83E-12 | DOWN |
| Fst | 10.80614778 | 0.07480739 | 6.171116311 | 6.171116311 | 1.77E-13 | 4.95E-12 | UP |
| Eml5 | 0.460030275 | 1.59053692 | -2.605812844 | 2.605812844 | 1.81E-13 | 5.04E-12 | DOWN |
| Hagh | 32.323489 | 115.638805 | -2.609639037 | 2.609639037 | 1.82E-13 | 5.07E-12 | DOWN |
| Col5a2 | 17.98260249 | 1.24586327 | 2.969894581 | 2.969894581 | 1.84E-13 | 5.13E-12 | UP |
| B3galt5 | 1.663609686 | 0.027055646 | 5.027206737 | 5.027206737 | 1.86E-13 | 5.15E-12 | UP |
| RGD1563692 | 1.208250367 | 0.004910908 | 6.725039224 | 6.725039224 | 1.95E-13 | 5.42E-12 | UP |
| Rnd1 | 10.36639024 | 0.865625515 | 2.743006889 | 2.743006889 | 2.00E-13 | 5.53E-12 | UP |
| Cpe | 6.868857504 | 0.17577858 | 4.44518751 | 4.44518751 | 2.06E-13 | 5.70E-12 | UP |
| Trpm4 | 6.104930182 | 0.757182893 | 2.158145061 | 2.158145061 | 2.45E-13 | 6.77E-12 | UP |
| Phldb1 | 3.131335368 | 0.313119787 | 2.483578285 | 2.483578285 | 2.46E-13 | 6.80E-12 | UP |
| Tlr5 | 2.158691372 | 0.072067757 | 4.02597324 | 4.02597324 | 2.57E-13 | 7.07E-12 | UP |
| Gpr183 | 5.675818115 | 0.146590523 | 4.356574795 | 4.356574795 | 2.76E-13 | 7.58E-12 | UP |
| Gulo | 79.96331546 | 270.3873007 | -2.550746574 | 2.550746574 | 2.79E-13 | 7.68E-12 | DOWN |
| F3 | 17.49769555 | 0.728528548 | 3.667309948 | 3.667309948 | 2.80E-13 | 7.69E-12 | UP |
| Stmn1 | 19.96664504 | 2.341100244 | 2.23337442 | 2.23337442 | 2.81E-13 | 7.69E-12 | UP |
| Cntfr | 0.180156505 | 2.098439924 | -4.322487702 | 4.322487702 | 2.84E-13 | 7.76E-12 | DOWN |
| Phyh | 447.987235 | 1815.037349 | -2.799850275 | 2.799850275 | 2.85E-13 | 7.78E-12 | DOWN |
| Parm1 | 4.847268701 | 0.225677522 | 3.499946654 | 3.499946654 | 2.97E-13 | 8.09E-12 | UP |
| Slc25a32 | 11.07009035 | 28.11121767 | -2.178001408 | 2.178001408 | 2.97E-13 | 8.09E-12 | DOWN |
| Ckmt1 | 5.746948086 | 0 | 10.18138727 | 10.18138727 | 3.01E-13 | 8.19E-12 | UP |
| RGD1307603 | 5.170480595 | 101.5151564 | -4.972197546 | 4.972197546 | 3.09E-13 | 8.40E-12 | DOWN |
| Ech1 | 181.8985131 | 448.1781226 | -2.127778302 | 2.127778302 | 3.16E-13 | 8.57E-12 | DOWN |
| Fzd2 | 7.146909067 | 0.214175323 | 4.144851229 | 4.144851229 | 3.22E-13 | 8.72E-12 | UP |
| Nid2 | 7.24659047 | 0.721241621 | 2.478386807 | 2.478386807 | 3.30E-13 | 8.93E-12 | UP |
| Acss1 | 7.48184202 | 0.545425049 | 2.914578681 | 2.914578681 | 3.39E-13 | 9.15E-12 | UP |
| Ninl | 2.96643068 | 0.117650566 | 3.748308378 | 3.748308378 | 3.46E-13 | 9.32E-12 | UP |
| Vwf | 12.04667181 | 1.207556613 | 2.498121417 | 2.498121417 | 3.81E-13 | 1.02E-11 | UP |
| Bgn | 444.2448635 | 57.24973284 | 2.088644853 | 2.088644853 | 3.87E-13 | 1.04E-11 | UP |
| Tgfb1i1 | 9.318500488 | 0.697968869 | 2.863905028 | 2.863905028 | 4.02E-13 | 1.08E-11 | UP |
| Tmem184c | 12.03164097 | 1.375497742 | 2.263808422 | 2.263808422 | 4.06E-13 | 1.09E-11 | UP |
| Igfals | 11.79211435 | 118.8119235 | -4.041086324 | 4.041086324 | 4.10E-13 | 1.10E-11 | DOWN |
| Ccdc141 | 2.862393949 | 0.276604397 | 2.51878073 | 2.51878073 | 4.23E-13 | 1.13E-11 | UP |
| Dhrs9 | 4.253960116 | 0.09425393 | 4.562607576 | 4.562607576 | 4.25E-13 | 1.13E-11 | UP |
| Cdkn2a | 5.877526677 | 0.043891012 | 6.029451742 | 6.029451742 | 4.24E-13 | 1.13E-11 | UP |
| Col6a2 | 45.10151122 | 4.653870715 | 2.409467295 | 2.409467295 | 4.35E-13 | 1.16E-11 | UP |
| Dync2li1 | 3.744091851 | 0.064484506 | 4.896788078 | 4.896788078 | 4.35E-13 | 1.16E-11 | UP |
| Dexi | 11.03623084 | 27.94785228 | -2.143208869 | 2.143208869 | 4.39E-13 | 1.16E-11 | DOWN |
| Incenp | 3.672511343 | 0.255938534 | 2.977145995 | 2.977145995 | 4.39E-13 | 1.16E-11 | UP |
| Espl1 | 1.151487786 | 0.053983085 | 3.548684522 | 3.548684522 | 4.41E-13 | 1.17E-11 | UP |
| Rfx5 | 8.655338761 | 1.099173098 | 2.114160929 | 2.114160929 | 4.44E-13 | 1.17E-11 | UP |
| Pde4c | 7.874204069 | 0 | 9.849624041 | 9.849624041 | 4.72E-13 | 1.24E-11 | UP |
| Efemp2 | 18.65365016 | 1.821179576 | 2.485068851 | 2.485068851 | 4.75E-13 | 1.25E-11 | UP |
| Cdc20 | 7.021871333 | 0.504103613 | 2.944472518 | 2.944472518 | 4.74E-13 | 1.25E-11 | UP |
| Rassf9 | 1.772807623 | 0.074842 | 3.699884268 | 3.699884268 | 4.91E-13 | 1.29E-11 | UP |
| Ccl2 | 11.67375991 | 0.220001091 | 4.774654229 | 4.774654229 | 4.92E-13 | 1.29E-11 | UP |
| Dpysl2 | 19.76863635 | 2.657924803 | 2.044482017 | 2.044482017 | 4.95E-13 | 1.29E-11 | UP |
| Soat1 | 26.24957216 | 2.211653153 | 2.659751844 | 2.659751844 | 5.09E-13 | 1.33E-11 | UP |
| Slc10a1 | 49.09192338 | 305.4276236 | -3.404428152 | 3.404428152 | 5.21E-13 | 1.36E-11 | DOWN |
| Disp1 | 9.290762027 | 0.893658106 | 2.543836424 | 2.543836424 | 5.34E-13 | 1.39E-11 | UP |
| Lpar1 | 2.709128159 | 0.093357682 | 3.964199602 | 3.964199602 | 5.39E-13 | 1.40E-11 | UP |
| Cdca3 | 6.676088446 | 0.328575609 | 3.472071946 | 3.472071946 | 5.79E-13 | 1.50E-11 | UP |
| Mrc2 | 3.902530215 | 0.17304729 | 3.67065852 | 3.67065852 | 5.83E-13 | 1.51E-11 | UP |
| Pon1 | 216.8837126 | 923.0750351 | -2.866653972 | 2.866653972 | 5.85E-13 | 1.51E-11 | DOWN |
| Gjc2 | 2.92614843 | 0.059098247 | 4.750887007 | 4.750887007 | 5.94E-13 | 1.53E-11 | UP |
| Ano1 | 8.357706102 | 0.144786662 | 4.940653918 | 4.940653918 | 5.96E-13 | 1.54E-11 | UP |
| Prtfdc1 | 15.37995627 | 1.63603522 | 2.365629 | 2.365629 | 6.01E-13 | 1.55E-11 | UP |
| Maged2 | 12.34547513 | 1.407759631 | 2.307510558 | 2.307510558 | 6.11E-13 | 1.57E-11 | UP |
| Lcp1 | 43.97048852 | 5.371621028 | 2.159286823 | 2.159286823 | 6.11E-13 | 1.57E-11 | UP |
| Nr0b2 | 9.081374572 | 39.77329078 | -2.885997058 | 2.885997058 | 6.15E-13 | 1.58E-11 | DOWN |
| Tuba1b | 235.6113471 | 26.97025964 | 2.23910181 | 2.23910181 | 6.19E-13 | 1.59E-11 | UP |
| Col3a1 | 299.2034589 | 26.07659462 | 2.631657493 | 2.631657493 | 6.57E-13 | 1.68E-11 | UP |
| Herpud1 | 75.86081725 | 184.861522 | -2.096614364 | 2.096614364 | 6.58E-13 | 1.68E-11 | DOWN |
| Atf5 | 159.7457905 | 634.2274004 | -2.768099384 | 2.768099384 | 6.66E-13 | 1.69E-11 | DOWN |
| Rcn2 | 31.63520113 | 3.336108355 | 2.347542663 | 2.347542663 | 6.81E-13 | 1.73E-11 | UP |
| Ndc80 | 2.567116625 | 0.043110494 | 4.97327822 | 4.97327822 | 6.88E-13 | 1.74E-11 | UP |
| Fzd4 | 7.312391564 | 17.00338964 | -2.028679814 | 2.028679814 | 6.92E-13 | 1.75E-11 | DOWN |
| Fads1 | 37.66763049 | 119.8319478 | -2.49663376 | 2.49663376 | 7.06E-13 | 1.79E-11 | DOWN |
| Shtn1 | 21.21410392 | 2.724491082 | 2.083021038 | 2.083021038 | 7.11E-13 | 1.80E-11 | UP |
| Pcyox1l | 7.43439633 | 0.276227882 | 3.857354588 | 3.857354588 | 7.53E-13 | 1.90E-11 | UP |
| Lrrcc1 | 4.080110925 | 0.202591333 | 3.424372734 | 3.424372734 | 7.59E-13 | 1.91E-11 | UP |
| Bok | 11.47745863 | 0.572020488 | 3.468639979 | 3.468639979 | 7.90E-13 | 1.99E-11 | UP |
| Osbpl5 | 3.802549437 | 0.241708175 | 3.094634376 | 3.094634376 | 8.35E-13 | 2.10E-11 | UP |
| Epn3 | 1.512180918 | 0 | 9.016469047 | 9.016469047 | 8.40E-13 | 2.11E-11 | UP |
| Ramp3 | 4.363505139 | 0.016726737 | 6.836761314 | 6.836761314 | 8.62E-13 | 2.16E-11 | UP |
| Ccl19 | 23.3215608 | 1.126399642 | 3.472474674 | 3.472474674 | 8.84E-13 | 2.21E-11 | UP |
| Gstz1 | 41.82375646 | 146.2266328 | -2.582750113 | 2.582750113 | 9.26E-13 | 2.31E-11 | DOWN |
| Htatip2 | 42.26643935 | 4.041720288 | 2.534787128 | 2.534787128 | 9.31E-13 | 2.32E-11 | UP |
| Kntc1 | 1.901268898 | 0.107247794 | 3.268361524 | 3.268361524 | 9.36E-13 | 2.33E-11 | UP |
| Cyp1a2 | 18.40036493 | 148.0371315 | -3.731926544 | 3.731926544 | 9.41E-13 | 2.34E-11 | DOWN |
| Ifi47 | 21.46681442 | 2.879180495 | 2.037043417 | 2.037043417 | 9.47E-13 | 2.35E-11 | UP |
| Rtn4rl2 | 3.999850158 | 17.76174961 | -2.908996476 | 2.908996476 | 9.73E-13 | 2.41E-11 | DOWN |
| Apoc1 | 26.97693005 | 152.0108308 | -3.231757176 | 3.231757176 | 9.77E-13 | 2.42E-11 | DOWN |
| Rps6ka2 | 3.030028714 | 0.28054602 | 2.582399826 | 2.582399826 | 9.84E-13 | 2.44E-11 | UP |
| Atrn | 26.70039665 | 65.02943971 | -2.095645246 | 2.095645246 | 9.92E-13 | 2.45E-11 | DOWN |
| Kifc1 | 2.448049443 | 0.102374065 | 3.713212001 | 3.713212001 | 1.02E-12 | 2.52E-11 | UP |
| Tnfsf18 | 3.582245527 | 0 | 9.452768689 | 9.452768689 | 1.05E-12 | 2.59E-11 | UP |
| Slc25a47 | 48.98880136 | 263.8678534 | -3.178160062 | 3.178160062 | 1.06E-12 | 2.60E-11 | DOWN |
| Igfbp7 | 604.7646063 | 72.83830748 | 2.177970511 | 2.177970511 | 1.07E-12 | 2.63E-11 | UP |
| Fam111a | 16.03828497 | 0.083219879 | 6.908026093 | 6.908026093 | 1.11E-12 | 2.72E-11 | UP |
| Dgat2 | 36.45697135 | 101.9042642 | -2.276921445 | 2.276921445 | 1.17E-12 | 2.87E-11 | DOWN |
| Gramd1c | 1.28243675 | 3.090137921 | -2.074792055 | 2.074792055 | 1.18E-12 | 2.89E-11 | DOWN |
| Tmem43 | 17.87628745 | 2.357165837 | 2.044660872 | 2.044660872 | 1.20E-12 | 2.94E-11 | UP |
| Hdgfl3 | 7.349874259 | 0.640219963 | 2.644254896 | 2.644254896 | 1.24E-12 | 3.04E-11 | UP |
| Enpep | 7.588656457 | 20.23969868 | -2.223976816 | 2.223976816 | 1.27E-12 | 3.09E-11 | DOWN |
| Sparcl1 | 43.96868908 | 5.093727134 | 2.266766945 | 2.266766945 | 1.27E-12 | 3.10E-11 | UP |
| Sncg | 6.572290272 | 0.1436974 | 4.649061585 | 4.649061585 | 1.39E-12 | 3.38E-11 | UP |
| Lrrc1 | 5.761143939 | 0.199493337 | 3.925972235 | 3.925972235 | 1.43E-12 | 3.46E-11 | UP |
| Tmem53 | 5.019346462 | 13.88819363 | -2.260697014 | 2.260697014 | 1.43E-12 | 3.46E-11 | DOWN |
| Gpc6 | 4.684404628 | 0.151695441 | 4.047643021 | 4.047643021 | 1.43E-12 | 3.46E-11 | UP |
| Tusc3 | 15.06685541 | 1.662174603 | 2.302134476 | 2.302134476 | 1.47E-12 | 3.55E-11 | UP |
| LOC685849 | 1.899704349 | 0.029468612 | 5.06226009 | 5.06226009 | 1.47E-12 | 3.56E-11 | UP |
| Hist2h4a | 15.49975287 | 39.38057789 | -2.172305856 | 2.172305856 | 1.50E-12 | 3.62E-11 | DOWN |
| Misp | 5.316163856 | 0.023699333 | 6.74099992 | 6.74099992 | 1.55E-12 | 3.72E-11 | UP |
| Egf | 0.242303536 | 1.31580307 | -3.22211915 | 3.22211915 | 1.60E-12 | 3.85E-11 | DOWN |
| Ankrd22 | 4.673347866 | 0.075029632 | 5.019652912 | 5.019652912 | 1.61E-12 | 3.88E-11 | UP |
| Akr1c1 | 3.368337642 | 35.51501383 | -4.277636803 | 4.277636803 | 1.63E-12 | 3.91E-11 | DOWN |
| Nnmt | 25.40405027 | 131.867868 | -3.13648835 | 3.13648835 | 1.67E-12 | 4.00E-11 | DOWN |
| Atp8b2 | 6.675484929 | 0.706122232 | 2.348025206 | 2.348025206 | 1.72E-12 | 4.11E-11 | UP |
| Cxcl10 | 27.66570498 | 3.159400889 | 2.282589691 | 2.282589691 | 1.72E-12 | 4.11E-11 | UP |
| Crym | 3.489182545 | 14.50986968 | -2.893900817 | 2.893900817 | 1.79E-12 | 4.27E-11 | DOWN |
| Amy1a | 55.73686339 | 261.3614105 | -2.977284521 | 2.977284521 | 1.82E-12 | 4.34E-11 | DOWN |
| Sult1c2a | 8.287946578 | 44.86693026 | -3.227871229 | 3.227871229 | 1.88E-12 | 4.48E-11 | DOWN |
| Retsat | 206.1696816 | 717.8313235 | -2.570974149 | 2.570974149 | 2.00E-12 | 4.75E-11 | DOWN |
| Anks6 | 1.160239219 | 0.039154822 | 4.005069804 | 4.005069804 | 2.18E-12 | 5.15E-11 | UP |
| Fzd6 | 8.912465106 | 0.873161225 | 2.466285085 | 2.466285085 | 2.19E-12 | 5.19E-11 | UP |
| Scara5 | 4.637524415 | 0.26919264 | 3.237010834 | 3.237010834 | 2.24E-12 | 5.28E-11 | UP |
| Acot5 | 4.853074433 | 13.75053471 | -2.381264102 | 2.381264102 | 2.40E-12 | 5.66E-11 | DOWN |
| Prkar2b | 2.377682265 | 0.063547563 | 4.306901837 | 4.306901837 | 2.41E-12 | 5.67E-11 | UP |
| Car9 | 3.219454431 | 0.079613475 | 4.425201998 | 4.425201998 | 2.41E-12 | 5.67E-11 | UP |
| Reck | 5.905817844 | 0.594691548 | 2.446885321 | 2.446885321 | 2.45E-12 | 5.76E-11 | UP |
| Cryl1 | 53.35548381 | 6.08966056 | 2.355278753 | 2.355278753 | 2.48E-12 | 5.82E-11 | UP |
| Sgo2 | 1.902951143 | 0.039501147 | 4.657672283 | 4.657672283 | 2.54E-12 | 5.95E-11 | UP |
| Rbp4 | 941.5955995 | 3173.618141 | -2.525262266 | 2.525262266 | 2.64E-12 | 6.18E-11 | DOWN |
| Tmem107 | 4.540899712 | 0.039383569 | 5.781812802 | 5.781812802 | 2.66E-12 | 6.20E-11 | UP |
| AABR07028488.1 | 6.110939081 | 0.306344601 | 3.468831876 | 3.468831876 | 2.68E-12 | 6.25E-11 | UP |
| Pecr | 99.78695093 | 237.4677438 | -2.064203395 | 2.064203395 | 2.68E-12 | 6.25E-11 | DOWN |
| Tor3a | 9.837550574 | 24.18431472 | -2.110360113 | 2.110360113 | 2.72E-12 | 6.34E-11 | DOWN |
| Hltf | 13.2065148 | 1.73812782 | 2.048800613 | 2.048800613 | 2.81E-12 | 6.53E-11 | UP |
| Sema7a | 3.124564808 | 0.062552959 | 4.690548726 | 4.690548726 | 3.19E-12 | 7.39E-11 | UP |
| Clic1 | 79.0659185 | 8.311033572 | 2.375673319 | 2.375673319 | 3.28E-12 | 7.59E-11 | UP |
| Cpa3 | 18.85498509 | 0.418862726 | 4.633771787 | 4.633771787 | 3.35E-12 | 7.73E-11 | UP |
| Cthrc1 | 4.066853314 | 0.016698105 | 6.669026703 | 6.669026703 | 3.42E-12 | 7.89E-11 | UP |
| Cyp2e1 | 168.683555 | 1212.101357 | -3.583916333 | 3.583916333 | 3.45E-12 | 7.94E-11 | DOWN |
| Clec11a | 8.828391712 | 0.610930335 | 2.991730945 | 2.991730945 | 3.48E-12 | 8.02E-11 | UP |
| Pllp | 6.100489567 | 0.013756705 | 7.517415849 | 7.517415849 | 3.62E-12 | 8.32E-11 | UP |
| Myadm | 67.15518666 | 8.977285919 | 2.031401509 | 2.031401509 | 3.80E-12 | 8.71E-11 | UP |
| B4galnt1 | 10.07480574 | 30.75057735 | -2.411863813 | 2.411863813 | 3.83E-12 | 8.79E-11 | DOWN |
| Sfxn3 | 12.09821111 | 1.094428511 | 2.576209763 | 2.576209763 | 3.86E-12 | 8.83E-11 | UP |
| LOC102553715 | 46.03787064 | 1.524634487 | 4.085409732 | 4.085409732 | 3.87E-12 | 8.86E-11 | UP |
| Frzb | 7.702402174 | 0.5312934 | 2.985926232 | 2.985926232 | 3.95E-12 | 9.03E-11 | UP |
| Apoc2 | 121.2844982 | 743.983298 | -3.34231501 | 3.34231501 | 4.00E-12 | 9.12E-11 | DOWN |
| Lgals4 | 98.41418803 | 0 | 13.20121971 | 13.20121971 | 4.03E-12 | 9.19E-11 | UP |
| Neurl3 | 20.30140557 | 2.600499606 | 2.113585072 | 2.113585072 | 4.11E-12 | 9.35E-11 | UP |
| Dbn1 | 6.979726693 | 0.400862213 | 3.219014367 | 3.219014367 | 4.18E-12 | 9.50E-11 | UP |
| Aldh1a3 | 43.77477271 | 4.508119077 | 2.428079073 | 2.428079073 | 4.26E-12 | 9.68E-11 | UP |
| Rab7b | 5.193262606 | 0.323963111 | 3.136941809 | 3.136941809 | 4.31E-12 | 9.78E-11 | UP |
| Sgcg | 2.045979107 | 0 | 8.302943007 | 8.302943007 | 4.39E-12 | 9.92E-11 | UP |
| Tymp | 7.924978719 | 35.40179675 | -2.945219915 | 2.945219915 | 4.39E-12 | 9.92E-11 | DOWN |
| Ttpa | 138.9692918 | 484.3595567 | -2.595987429 | 2.595987429 | 4.43E-12 | 1.00E-10 | DOWN |
| LOC102549542 | 5.913052181 | 0.392401893 | 3.027153209 | 3.027153209 | 4.52E-12 | 1.02E-10 | UP |
| LOC498222 | 4.350471864 | 0.078212061 | 4.868260248 | 4.868260248 | 4.53E-12 | 1.02E-10 | UP |
| Usp2 | 2.377564248 | 12.66549809 | -3.174943245 | 3.174943245 | 4.65E-12 | 1.05E-10 | DOWN |
| Cyp2d5 | 108.9866533 | 338.1832888 | -2.416275456 | 2.416275456 | 4.68E-12 | 1.05E-10 | DOWN |
| Tacc2 | 7.618299721 | 0.895910291 | 2.195200908 | 2.195200908 | 4.70E-12 | 1.06E-10 | UP |
| Rarres2 | 179.7905746 | 605.968804 | -2.522763823 | 2.522763823 | 4.84E-12 | 1.08E-10 | DOWN |
| Gucy1b2 | 1.03742504 | 3.7158802 | -2.633012607 | 2.633012607 | 4.90E-12 | 1.10E-10 | DOWN |
| Gclc | 319.4162993 | 44.36650647 | 2.000795193 | 2.000795193 | 4.94E-12 | 1.11E-10 | UP |
| Slc19a2 | 7.403580649 | 20.6550067 | -2.325041427 | 2.325041427 | 5.05E-12 | 1.13E-10 | DOWN |
| Fstl3 | 4.922188117 | 0.151465321 | 4.148089084 | 4.148089084 | 5.06E-12 | 1.13E-10 | UP |
| Spata6 | 7.462633806 | 0.531692345 | 2.942665601 | 2.942665601 | 5.10E-12 | 1.14E-10 | UP |
| Cdr2 | 10.15923763 | 0.863575292 | 2.686173625 | 2.686173625 | 5.16E-12 | 1.15E-10 | UP |
| AABR07064312.1 | 0.137161465 | 3.892820276 | -5.437540507 | 5.437540507 | 5.17E-12 | 1.15E-10 | DOWN |
| Casc4 | 3.670591272 | 0.176391855 | 3.465739616 | 3.465739616 | 5.45E-12 | 1.21E-10 | UP |
| Coro6 | 2.578753771 | 0.098710797 | 3.880160571 | 3.880160571 | 5.48E-12 | 1.21E-10 | UP |
| Cd55 | 9.564851222 | 0.065794429 | 6.183248072 | 6.183248072 | 5.49E-12 | 1.21E-10 | UP |
| AABR07057436.1 | 64.0914526 | 350.1657653 | -3.186277926 | 3.186277926 | 5.60E-12 | 1.24E-10 | DOWN |
| Dcxr | 20.11436117 | 69.6581035 | -2.558393196 | 2.558393196 | 5.74E-12 | 1.27E-10 | DOWN |
| Mrvi1 | 3.2244908 | 0.268189299 | 2.72737753 | 2.72737753 | 5.85E-12 | 1.29E-10 | UP |
| Bcl2l14 | 4.3469688 | 0.067609912 | 5.048408521 | 5.048408521 | 6.54E-12 | 1.44E-10 | UP |
| LOC102556347 | 11.47598788 | 0.868758169 | 2.857530937 | 2.857530937 | 6.54E-12 | 1.44E-10 | UP |
| Kif2c | 1.81594076 | 0.067968935 | 3.852547638 | 3.852547638 | 6.56E-12 | 1.44E-10 | UP |
| Paqr7 | 15.52274503 | 60.39346655 | -2.7252005 | 2.7252005 | 6.59E-12 | 1.45E-10 | DOWN |
| Col15a1 | 3.835449416 | 0.011733292 | 7.269001089 | 7.269001089 | 7.09E-12 | 1.55E-10 | UP |
| Lpin1 | 7.748799121 | 47.15826837 | -3.41813928 | 3.41813928 | 7.28E-12 | 1.59E-10 | DOWN |
| Me2 | 12.30803322 | 1.677553624 | 2.007102992 | 2.007102992 | 7.80E-12 | 1.70E-10 | UP |
| Pik3cg | 4.306429149 | 0.491972628 | 2.252115355 | 2.252115355 | 7.95E-12 | 1.73E-10 | UP |
| Edn1 | 3.848832366 | 0.059249901 | 5.057932144 | 5.057932144 | 8.00E-12 | 1.74E-10 | UP |
| Sntb1 | 7.571816781 | 0.90253384 | 2.238705377 | 2.238705377 | 8.15E-12 | 1.77E-10 | UP |
| Rhoj | 11.39645693 | 1.514315834 | 2.075219489 | 2.075219489 | 8.17E-12 | 1.78E-10 | UP |
| Crybg2 | 3.017255388 | 9.11821671 | -2.389386942 | 2.389386942 | 8.31E-12 | 1.80E-10 | DOWN |
| Ms4a2 | 2.50757849 | 0.068528699 | 4.275245265 | 4.275245265 | 8.39E-12 | 1.82E-10 | UP |
| Tmprss4 | 2.15410714 | 0 | 9.14263553 | 9.14263553 | 8.45E-12 | 1.83E-10 | UP |
| Lrrk2 | 3.984672563 | 0.426381132 | 2.373603428 | 2.373603428 | 8.59E-12 | 1.86E-10 | UP |
| Wnt7b | 1.483057126 | 0.017671499 | 5.418231263 | 5.418231263 | 8.67E-12 | 1.87E-10 | UP |
| Habp2 | 22.21541523 | 60.25670337 | -2.236062131 | 2.236062131 | 8.72E-12 | 1.88E-10 | DOWN |
| Capn5 | 10.20423035 | 1.141923524 | 2.283564932 | 2.283564932 | 8.83E-12 | 1.90E-10 | UP |
| Cldn14 | 6.038915484 | 20.8655483 | -2.549902411 | 2.549902411 | 8.90E-12 | 1.91E-10 | DOWN |
| LOC102552659 | 4.958438328 | 0.096103052 | 4.768835815 | 4.768835815 | 8.94E-12 | 1.92E-10 | UP |
| E2f8 | 1.964721256 | 0.039166483 | 4.731694256 | 4.731694256 | 8.99E-12 | 1.93E-10 | UP |
| Tpsb2 | 4.253545437 | 0.044736406 | 5.605253801 | 5.605253801 | 9.24E-12 | 1.98E-10 | UP |
| Grhpr | 30.26601065 | 115.4947347 | -2.689212721 | 2.689212721 | 9.33E-12 | 2.00E-10 | DOWN |
| Tgfb2 | 6.69201936 | 0.508275798 | 2.811035335 | 2.811035335 | 9.55E-12 | 2.04E-10 | UP |
| Cyp2d2 | 156.9483175 | 791.740357 | -3.078329834 | 3.078329834 | 9.92E-12 | 2.12E-10 | DOWN |
| Car4 | 15.98496485 | 0 | 10.89363896 | 10.89363896 | 1.01E-11 | 2.15E-10 | UP |
| Acot4 | 13.27475861 | 39.13256648 | -2.35525879 | 2.35525879 | 1.03E-11 | 2.19E-10 | DOWN |
| Depdc1 | 2.023893823 | 0.037262348 | 4.808629889 | 4.808629889 | 1.04E-11 | 2.21E-10 | UP |
| Chrm3 | 1.382150185 | 0.01140996 | 5.873191175 | 5.873191175 | 1.05E-11 | 2.24E-10 | UP |
| Unc13b | 2.061441042 | 0.27876948 | 2.034505936 | 2.034505936 | 1.17E-11 | 2.47E-10 | UP |
| Pkp1 | 1.233666784 | 0 | 9.065897174 | 9.065897174 | 1.20E-11 | 2.54E-10 | UP |
| Cyp51 | 30.33110098 | 70.71342812 | -2.047896349 | 2.047896349 | 1.20E-11 | 2.55E-10 | DOWN |
| Cyp1b1 | 5.041995661 | 0.055019921 | 5.585191513 | 5.585191513 | 1.25E-11 | 2.65E-10 | UP |
| Dtl | 1.371798841 | 0.012743187 | 5.69335094 | 5.69335094 | 1.26E-11 | 2.66E-10 | UP |
| Thy1 | 6.617394483 | 0.185481074 | 4.272551005 | 4.272551005 | 1.26E-11 | 2.66E-10 | UP |
| Hmgcs2 | 420.1743036 | 1309.546354 | -2.426620368 | 2.426620368 | 1.28E-11 | 2.69E-10 | DOWN |
| Ehd2 | 14.89613896 | 1.309611136 | 2.625175802 | 2.625175802 | 1.28E-11 | 2.70E-10 | UP |
| Map4k4 | 15.24510941 | 1.701285441 | 2.31129875 | 2.31129875 | 1.28E-11 | 2.70E-10 | UP |
| Plk4 | 2.606495839 | 0.159277071 | 3.150602692 | 3.150602692 | 1.33E-11 | 2.80E-10 | UP |
| Msantd3 | 1.190030747 | 0.032146442 | 4.340884925 | 4.340884925 | 1.48E-11 | 3.09E-10 | UP |
| Ahcy | 215.1087671 | 599.6623798 | -2.26800169 | 2.26800169 | 1.49E-11 | 3.12E-10 | DOWN |
| Baat | 121.4261619 | 528.8769399 | -2.8811411 | 2.8811411 | 1.50E-11 | 3.12E-10 | DOWN |
| Cyp2d4 | 8.07319265 | 25.30524624 | -2.417505313 | 2.417505313 | 1.50E-11 | 3.12E-10 | DOWN |
| Slc25a22 | 42.29210908 | 134.2945948 | -2.435876904 | 2.435876904 | 1.49E-11 | 3.12E-10 | DOWN |
| Acsm5 | 11.39354873 | 64.64000818 | -3.302904568 | 3.302904568 | 1.50E-11 | 3.13E-10 | DOWN |
| Abcc6 | 8.520115379 | 36.10540667 | -2.828430747 | 2.828430747 | 1.54E-11 | 3.20E-10 | DOWN |
| Evi2a | 7.026917424 | 0.378347641 | 3.32535828 | 3.32535828 | 1.59E-11 | 3.30E-10 | UP |
| Mboat1 | 23.45286382 | 1.671193806 | 2.898887071 | 2.898887071 | 1.62E-11 | 3.36E-10 | UP |
| Zfp827 | 1.089185335 | 0 | 8.413693021 | 8.413693021 | 1.75E-11 | 3.63E-10 | UP |
| Spon1 | 5.47343264 | 0.22270424 | 3.731348611 | 3.731348611 | 1.75E-11 | 3.64E-10 | UP |
| Cnksr1 | 1.690709656 | 0.04112029 | 4.452944211 | 4.452944211 | 1.79E-11 | 3.70E-10 | UP |
| Apoc3 | 266.7952268 | 1701.239096 | -3.391633435 | 3.391633435 | 1.81E-11 | 3.75E-10 | DOWN |
| Acot9 | 25.93796948 | 2.590409032 | 2.424853594 | 2.424853594 | 1.87E-11 | 3.86E-10 | UP |
| Kif4a | 2.019311424 | 0.099708566 | 3.487432199 | 3.487432199 | 1.95E-11 | 4.01E-10 | UP |
| Adamts5 | 4.542409559 | 0.444109828 | 2.519325768 | 2.519325768 | 2.03E-11 | 4.17E-10 | UP |
| Svop | 1.036658744 | 0 | 8.366547846 | 8.366547846 | 2.06E-11 | 4.23E-10 | UP |
| Pck1 | 437.6419834 | 1608.95367 | -2.63885655 | 2.63885655 | 2.12E-11 | 4.33E-10 | DOWN |
| Ndrg2 | 65.66739261 | 217.0835021 | -2.505964338 | 2.505964338 | 2.15E-11 | 4.38E-10 | DOWN |
| Gal3st1 | 1.657647174 | 0.012181736 | 6.038123501 | 6.038123501 | 2.16E-11 | 4.41E-10 | UP |
| Tff3 | 51.59150137 | 0.597406006 | 5.47281169 | 5.47281169 | 2.16E-11 | 4.41E-10 | UP |
| Ttc22 | 2.40941475 | 0.025410605 | 5.573374476 | 5.573374476 | 2.18E-11 | 4.43E-10 | UP |
| Setd7 | 5.21371263 | 0.331479157 | 3.100445243 | 3.100445243 | 2.26E-11 | 4.60E-10 | UP |
| Racgap1 | 1.293039618 | 0.006901732 | 6.303741086 | 6.303741086 | 2.28E-11 | 4.62E-10 | UP |
| Lcat | 112.4813111 | 516.3421822 | -2.941852548 | 2.941852548 | 2.36E-11 | 4.78E-10 | DOWN |
| Nlrc4 | 8.709550985 | 0.426093672 | 3.457155755 | 3.457155755 | 2.46E-11 | 4.97E-10 | UP |
| Cth | 66.81902345 | 356.7788626 | -3.14832819 | 3.14832819 | 2.46E-11 | 4.97E-10 | DOWN |
| Fmo3 | 100.3858898 | 291.5866949 | -2.332866442 | 2.332866442 | 2.48E-11 | 5.00E-10 | DOWN |
| Col1a2 | 98.66743741 | 5.858132788 | 3.174926001 | 3.174926001 | 2.59E-11 | 5.22E-10 | UP |
| Slc7a5 | 6.301051446 | 0.770084056 | 2.167406774 | 2.167406774 | 2.61E-11 | 5.25E-10 | UP |
| Synj2 | 4.316028276 | 0.459076568 | 2.396803399 | 2.396803399 | 2.63E-11 | 5.28E-10 | UP |
| Kyat3 | 32.97957013 | 87.54990106 | -2.200075764 | 2.200075764 | 2.63E-11 | 5.28E-10 | DOWN |
| Ranbp17 | 0.271746052 | 1.173003216 | -2.930113057 | 2.930113057 | 2.70E-11 | 5.43E-10 | DOWN |
| Slc6a13 | 7.840445328 | 45.22706885 | -3.247121054 | 3.247121054 | 2.80E-11 | 5.63E-10 | DOWN |
| Pemt | 46.40949699 | 182.7957751 | -2.724717236 | 2.724717236 | 2.87E-11 | 5.75E-10 | DOWN |
| Rasa3 | 10.79583424 | 1.336825697 | 2.140055538 | 2.140055538 | 2.90E-11 | 5.81E-10 | UP |
| Slc26a1 | 19.36767894 | 85.81975714 | -2.889279412 | 2.889279412 | 3.01E-11 | 6.00E-10 | DOWN |
| Map1b | 5.491358324 | 0.252431893 | 3.5642713 | 3.5642713 | 3.01E-11 | 6.00E-10 | UP |
| C7 | 58.20672694 | 3.23330711 | 3.326818493 | 3.326818493 | 3.01E-11 | 6.00E-10 | UP |
| Shf | 2.712155181 | 7.26766535 | -2.237693898 | 2.237693898 | 3.03E-11 | 6.04E-10 | DOWN |
| Cavin1 | 31.29298127 | 4.068862266 | 2.110132788 | 2.110132788 | 3.08E-11 | 6.12E-10 | UP |
| Ptpn14 | 1.362973912 | 0.059366716 | 3.667606974 | 3.667606974 | 3.11E-11 | 6.18E-10 | UP |
| Bdh1 | 50.47603415 | 141.2038843 | -2.268147995 | 2.268147995 | 3.14E-11 | 6.24E-10 | DOWN |
| Fut4 | 6.16164135 | 0.634479428 | 2.413008558 | 2.413008558 | 3.18E-11 | 6.31E-10 | UP |
| Clic6 | 2.131578421 | 0 | 10.10062897 | 10.10062897 | 3.18E-11 | 6.31E-10 | UP |
| Kcnk1 | 1.733535517 | 0.044261105 | 4.351289597 | 4.351289597 | 3.23E-11 | 6.39E-10 | UP |
| Adgrg6 | 12.14941905 | 1.443809729 | 2.195428087 | 2.195428087 | 3.29E-11 | 6.50E-10 | UP |
| Fmo1 | 15.20845048 | 35.26812266 | -2.025260324 | 2.025260324 | 3.30E-11 | 6.52E-10 | DOWN |
| Cyp4f1 | 74.82542264 | 259.3299515 | -2.557529062 | 2.557529062 | 3.31E-11 | 6.53E-10 | DOWN |
| Mafb | 26.28833939 | 62.11516619 | -2.070387665 | 2.070387665 | 3.32E-11 | 6.54E-10 | DOWN |
| Tmem54 | 3.005395977 | 0 | 9.487590516 | 9.487590516 | 3.44E-11 | 6.78E-10 | UP |
| Fabp5 | 49.49537389 | 5.26135027 | 2.360085193 | 2.360085193 | 3.49E-11 | 6.86E-10 | UP |
| Slfn13 | 5.020922268 | 0.629186507 | 2.119721757 | 2.119721757 | 3.50E-11 | 6.88E-10 | UP |
| Gadd45g | 11.08484931 | 42.70216708 | -2.770043875 | 2.770043875 | 3.55E-11 | 6.98E-10 | DOWN |
| Knstrn | 3.428258895 | 0.079257538 | 4.48764088 | 4.48764088 | 3.56E-11 | 6.98E-10 | UP |
| Chac1 | 3.684635215 | 16.05746423 | -2.951015192 | 2.951015192 | 3.60E-11 | 7.05E-10 | DOWN |
| Pald1 | 6.819558442 | 21.0425234 | -2.394896164 | 2.394896164 | 3.61E-11 | 7.08E-10 | DOWN |
| Myh14 | 8.165872282 | 0.41074293 | 3.430840965 | 3.430840965 | 3.69E-11 | 7.22E-10 | UP |
| Ptger4 | 6.517094411 | 0.319698723 | 3.444875133 | 3.444875133 | 3.73E-11 | 7.29E-10 | UP |
| Jag2 | 1.877432328 | 0.094795299 | 3.407405045 | 3.407405045 | 3.78E-11 | 7.37E-10 | UP |
| Serpine1 | 21.56317025 | 1.146345522 | 3.356942279 | 3.356942279 | 3.81E-11 | 7.41E-10 | UP |
| Chchd10 | 90.34783117 | 302.8636621 | -2.510547393 | 2.510547393 | 3.83E-11 | 7.46E-10 | DOWN |
| LOC103691744 | 4.433107654 | 21.21538331 | -2.993872285 | 2.993872285 | 3.85E-11 | 7.49E-10 | DOWN |
| Hebp2 | 6.470476145 | 0.321545831 | 3.462614869 | 3.462614869 | 3.90E-11 | 7.57E-10 | UP |
| Amacr | 26.24464329 | 116.4496476 | -2.897803529 | 2.897803529 | 3.99E-11 | 7.74E-10 | DOWN |
| Ace2 | 3.330805164 | 0.221114181 | 3.051655914 | 3.051655914 | 4.05E-11 | 7.84E-10 | UP |
| Extl1 | 1.74009784 | 6.358922358 | -2.626148018 | 2.626148018 | 4.11E-11 | 7.94E-10 | DOWN |
| Ddr2 | 2.579040205 | 0.09803905 | 3.831780668 | 3.831780668 | 4.13E-11 | 7.98E-10 | UP |
| Micall2 | 3.097183089 | 0.244190735 | 2.825851139 | 2.825851139 | 4.17E-11 | 8.04E-10 | UP |
| Csf3r | 2.491675138 | 0.114889601 | 3.518653878 | 3.518653878 | 4.20E-11 | 8.10E-10 | UP |
| Gpr160 | 4.365830202 | 0.191491237 | 3.628547675 | 3.628547675 | 4.27E-11 | 8.22E-10 | UP |
| Atoh8 | 5.207184069 | 14.94157682 | -2.295117695 | 2.295117695 | 4.31E-11 | 8.30E-10 | DOWN |
| Pxmp2 | 41.50723256 | 137.1038014 | -2.495118242 | 2.495118242 | 4.32E-11 | 8.30E-10 | DOWN |
| Il1r1 | 12.48559837 | 29.64009569 | -2.050914113 | 2.050914113 | 4.39E-11 | 8.42E-10 | DOWN |
| Erbb2 | 1.626010131 | 0.036790477 | 4.503901738 | 4.503901738 | 4.55E-11 | 8.72E-10 | UP |
| Sestd1 | 11.51042918 | 1.200559373 | 2.362643483 | 2.362643483 | 4.73E-11 | 9.05E-10 | UP |
| Ctbp2 | 6.348227728 | 0.783112153 | 2.15586566 | 2.15586566 | 4.77E-11 | 9.11E-10 | UP |
| Pak1 | 16.2204575 | 2.041265222 | 2.100599232 | 2.100599232 | 4.95E-11 | 9.44E-10 | UP |
| Pradc1 | 7.922355307 | 18.30774052 | -2.015773804 | 2.015773804 | 4.97E-11 | 9.48E-10 | DOWN |
| Dpys | 34.11005438 | 126.7915541 | -2.65037649 | 2.65037649 | 5.03E-11 | 9.59E-10 | DOWN |
| Gramd1b | 5.313388828 | 0.508983578 | 2.529077963 | 2.529077963 | 5.11E-11 | 9.73E-10 | UP |
| Defa5 | 7.253285778 | 0 | 8.436415781 | 8.436415781 | 5.29E-11 | 1.00E-09 | UP |
| Hjurp | 2.541172783 | 0.107649453 | 3.689980657 | 3.689980657 | 5.60E-11 | 1.06E-09 | UP |
| Gls | 13.62800849 | 1.375104723 | 2.394737024 | 2.394737024 | 5.78E-11 | 1.09E-09 | UP |
| Ptprd | 4.052450227 | 15.69862551 | -2.723790713 | 2.723790713 | 6.07E-11 | 1.15E-09 | DOWN |
| Vnn3 | 2.853730133 | 23.54052693 | -3.739251968 | 3.739251968 | 6.11E-11 | 1.15E-09 | DOWN |
| Evc | 3.945797122 | 9.614042771 | -2.104352719 | 2.104352719 | 6.24E-11 | 1.18E-09 | DOWN |
| Ccl21 | 96.63156872 | 3.831967187 | 3.847303886 | 3.847303886 | 6.25E-11 | 1.18E-09 | UP |
| Gpr176 | 2.564043825 | 0.075138524 | 4.19470692 | 4.19470692 | 6.35E-11 | 1.20E-09 | UP |
| Fahd1 | 12.57572913 | 35.12449331 | -2.276838878 | 2.276838878 | 6.65E-11 | 1.25E-09 | DOWN |
| Tgfbi | 40.74346466 | 97.96247764 | -2.067412486 | 2.067412486 | 6.69E-11 | 1.26E-09 | DOWN |
| Ttc21b | 9.942575131 | 1.258347898 | 2.114269609 | 2.114269609 | 6.82E-11 | 1.28E-09 | UP |
| AC128848.1 | 13.76252159 | 81.5184874 | -3.320112538 | 3.320112538 | 6.84E-11 | 1.28E-09 | DOWN |
| Azgp1 | 96.42093246 | 602.6284169 | -3.370329477 | 3.370329477 | 6.91E-11 | 1.29E-09 | DOWN |
| Basp1 | 15.40394605 | 1.365247217 | 2.630715211 | 2.630715211 | 6.92E-11 | 1.29E-09 | UP |
| AABR07025140.1 | 3.91245631 | 0.397044401 | 2.445390163 | 2.445390163 | 6.95E-11 | 1.30E-09 | UP |
| Cfhr1 | 111.2482673 | 420.637184 | -2.690829755 | 2.690829755 | 7.08E-11 | 1.32E-09 | DOWN |
| Orai2 | 3.315815076 | 0.276759343 | 2.708515836 | 2.708515836 | 7.22E-11 | 1.34E-09 | UP |
| Sh3rf2 | 2.262200909 | 0 | 9.016415405 | 9.016415405 | 7.48E-11 | 1.39E-09 | UP |
| RGD1563400 | 12.18113504 | 0.388116736 | 4.051783201 | 4.051783201 | 7.55E-11 | 1.40E-09 | UP |
| Apom | 30.90248995 | 138.1296511 | -2.902920418 | 2.902920418 | 7.59E-11 | 1.40E-09 | DOWN |
| P2ry2 | 14.27053712 | 40.54549667 | -2.279041694 | 2.279041694 | 7.84E-11 | 1.45E-09 | DOWN |
| Dcbld2 | 6.563783165 | 0.462671253 | 2.931344702 | 2.931344702 | 7.87E-11 | 1.45E-09 | UP |
| Car3 | 2.441587868 | 394.5305104 | -7.957731829 | 7.957731829 | 7.95E-11 | 1.47E-09 | DOWN |
| A2ml1 | 0.173020228 | 7.62097612 | -6.421461993 | 6.421461993 | 8.13E-11 | 1.50E-09 | DOWN |
| Asgr2 | 32.66055863 | 88.24323348 | -2.215939939 | 2.215939939 | 8.26E-11 | 1.52E-09 | DOWN |
| LOC100911564 | 1.815237062 | 27.15867872 | -4.58054131 | 4.58054131 | 9.02E-11 | 1.65E-09 | DOWN |
| Grem2 | 5.491116546 | 0.451035227 | 2.821497239 | 2.821497239 | 9.05E-11 | 1.65E-09 | UP |
| Sapcd2 | 2.32578231 | 0 | 8.43784851 | 8.43784851 | 9.34E-11 | 1.71E-09 | UP |
| Cideb | 30.60115013 | 80.85293876 | -2.181935942 | 2.181935942 | 9.35E-11 | 1.71E-09 | DOWN |
| Afm | 131.2843701 | 590.4426179 | -2.918607848 | 2.918607848 | 9.51E-11 | 1.73E-09 | DOWN |
| Proc | 68.31928296 | 215.1720776 | -2.421797184 | 2.421797184 | 9.94E-11 | 1.81E-09 | DOWN |
| Olfr12 | 0.063271203 | 2.201970936 | -5.720018907 | 5.720018907 | 1.02E-10 | 1.85E-09 | DOWN |
| Txnrd1 | 238.143965 | 32.20795067 | 2.007348881 | 2.007348881 | 1.02E-10 | 1.86E-09 | UP |
| AC114343.1 | 9.86835249 | 53.5217466 | -3.205512833 | 3.205512833 | 1.02E-10 | 1.86E-09 | DOWN |
| Acadm | 119.168794 | 297.8686773 | -2.111256425 | 2.111256425 | 1.04E-10 | 1.88E-09 | DOWN |
| Sptssb | 4.226551748 | 0.009298721 | 7.548903249 | 7.548903249 | 1.04E-10 | 1.88E-09 | UP |
| Pm20d1 | 5.432400647 | 13.40334173 | -2.096376971 | 2.096376971 | 1.06E-10 | 1.91E-09 | DOWN |
| Col8a1 | 8.593201545 | 0.703954771 | 2.754216777 | 2.754216777 | 1.06E-10 | 1.91E-09 | UP |
| Aqp3 | 3.720392959 | 0.011399929 | 7.063568617 | 7.063568617 | 1.07E-10 | 1.93E-09 | UP |
| Pfkfb1 | 13.43034805 | 33.71298813 | -2.112590205 | 2.112590205 | 1.09E-10 | 1.97E-09 | DOWN |
| Itih1 | 129.5241289 | 453.1189503 | -2.565496883 | 2.565496883 | 1.09E-10 | 1.97E-09 | DOWN |
| Cep55 | 2.796829725 | 0.05736796 | 4.665317331 | 4.665317331 | 1.10E-10 | 1.98E-09 | UP |
| RT1-Ba | 49.90734237 | 5.871190942 | 2.22080561 | 2.22080561 | 1.10E-10 | 1.98E-09 | UP |
| Slco2b1 | 15.65153277 | 44.39191452 | -2.276342146 | 2.276342146 | 1.11E-10 | 1.99E-09 | DOWN |
| Mybl1 | 8.349176938 | 0.903326791 | 2.33711579 | 2.33711579 | 1.13E-10 | 2.03E-09 | UP |
| UST4r | 12.59116407 | 45.38199351 | -2.620419282 | 2.620419282 | 1.16E-10 | 2.08E-09 | DOWN |
| Slc27a2 | 171.1448161 | 423.0662997 | -2.104583486 | 2.104583486 | 1.17E-10 | 2.09E-09 | DOWN |
| NEWGENE_2134 | 204.302443 | 1061.731034 | -3.116182683 | 3.116182683 | 1.18E-10 | 2.11E-09 | DOWN |
| Cxcr4 | 10.34855942 | 0.782359576 | 2.806341053 | 2.806341053 | 1.21E-10 | 2.16E-09 | UP |
| Osbpl10 | 2.969629371 | 0.173322127 | 3.209240572 | 3.209240572 | 1.22E-10 | 2.17E-09 | UP |
| Ucp2 | 174.0892142 | 18.08953855 | 2.402250785 | 2.402250785 | 1.23E-10 | 2.19E-09 | UP |
| Nav1 | 3.67830307 | 0.507596989 | 2.004369336 | 2.004369336 | 1.25E-10 | 2.22E-09 | UP |
| Prr15l | 13.89947 | 0.201092359 | 5.108058037 | 5.108058037 | 1.31E-10 | 2.32E-09 | UP |
| Prss8 | 9.730361039 | 0.576836215 | 3.191177477 | 3.191177477 | 1.35E-10 | 2.38E-09 | UP |
| Slc22a7 | 9.714156935 | 37.7089748 | -2.701818257 | 2.701818257 | 1.35E-10 | 2.38E-09 | DOWN |
| Efna5 | 4.285557513 | 12.17828447 | -2.360464431 | 2.360464431 | 1.43E-10 | 2.51E-09 | DOWN |
| Nuf2 | 4.346944351 | 0.150779125 | 3.97714443 | 3.97714443 | 1.43E-10 | 2.52E-09 | UP |
| AC123346.1 | 0.901928666 | 2.326189924 | -2.21157403 | 2.21157403 | 1.43E-10 | 2.52E-09 | DOWN |
| Zfp462 | 1.335247408 | 0.130319937 | 2.505402161 | 2.505402161 | 1.44E-10 | 2.52E-09 | UP |
| Gcnt1 | 9.532714297 | 0.903923596 | 2.510738708 | 2.510738708 | 1.46E-10 | 2.56E-09 | UP |
| LOC100910708 | 3.204820146 | 0 | 8.163471821 | 8.163471821 | 1.49E-10 | 2.61E-09 | UP |
| RT1-Da | 167.8061287 | 16.80061842 | 2.453470109 | 2.453470109 | 1.49E-10 | 2.61E-09 | UP |
| Elovl6 | 5.417773875 | 0.276579372 | 3.413474023 | 3.413474023 | 1.51E-10 | 2.64E-09 | UP |
| Etv4 | 2.375820194 | 0.024446981 | 5.565363742 | 5.565363742 | 1.58E-10 | 2.76E-09 | UP |
| Pkdcc | 19.34321608 | 49.28946412 | -2.147619959 | 2.147619959 | 1.59E-10 | 2.77E-09 | DOWN |
| Fopnl | 12.4947791 | 1.533796722 | 2.16342946 | 2.16342946 | 1.60E-10 | 2.78E-09 | UP |
| Gbp5 | 7.266508286 | 0.835005826 | 2.284325784 | 2.284325784 | 1.64E-10 | 2.85E-09 | UP |
| Oxct1 | 6.96055574 | 0.626201453 | 2.569484792 | 2.569484792 | 1.67E-10 | 2.89E-09 | UP |
| Loxl2 | 11.40898777 | 1.323299855 | 2.270056929 | 2.270056929 | 1.70E-10 | 2.94E-09 | UP |
| RatNP-3b | 3.217028077 | 0.023484285 | 5.927575245 | 5.927575245 | 1.71E-10 | 2.96E-09 | UP |
| Cep72 | 1.28656692 | 0.009432428 | 5.882890773 | 5.882890773 | 1.73E-10 | 2.99E-09 | UP |
| Pycr1 | 5.129020026 | 0.278888703 | 3.339408932 | 3.339408932 | 1.73E-10 | 2.99E-09 | UP |
| Apoh | 1011.745668 | 2935.295436 | -2.310734048 | 2.310734048 | 1.78E-10 | 3.08E-09 | DOWN |
| Specc1 | 3.525906815 | 0.463584055 | 2.053277211 | 2.053277211 | 1.81E-10 | 3.12E-09 | UP |
| Grik5 | 1.80359826 | 0.108955661 | 3.192587262 | 3.192587262 | 1.82E-10 | 3.14E-09 | UP |
| Slc38a2 | 36.12584551 | 88.04085211 | -2.156275957 | 2.156275957 | 1.83E-10 | 3.16E-09 | DOWN |
| Plac8 | 77.85800776 | 8.228799783 | 2.348618589 | 2.348618589 | 1.86E-10 | 3.20E-09 | UP |
| Hal | 43.67445909 | 146.3583748 | -2.520333253 | 2.520333253 | 1.88E-10 | 3.23E-09 | DOWN |
| Nme3 | 10.20626901 | 26.07625774 | -2.152370963 | 2.152370963 | 1.88E-10 | 3.23E-09 | DOWN |
| RT1-DMa | 12.34922153 | 1.628382483 | 2.06191068 | 2.06191068 | 1.89E-10 | 3.25E-09 | UP |
| Gpr146 | 8.802948207 | 21.8196237 | -2.093321469 | 2.093321469 | 1.90E-10 | 3.26E-09 | DOWN |
| Aspn | 4.962993435 | 0.26905366 | 3.295491206 | 3.295491206 | 1.90E-10 | 3.26E-09 | UP |
| Slc41a2 | 28.49911192 | 73.77241695 | -2.161282909 | 2.161282909 | 1.93E-10 | 3.30E-09 | DOWN |
| Ano6 | 10.30694027 | 1.213768564 | 2.195265367 | 2.195265367 | 1.96E-10 | 3.35E-09 | UP |
| Serpinb6a | 66.12763831 | 6.636795931 | 2.405095268 | 2.405095268 | 2.02E-10 | 3.45E-09 | UP |
| Adra1b | 3.110148739 | 13.9139989 | -2.903422013 | 2.903422013 | 2.07E-10 | 3.53E-09 | DOWN |
| Hsd17b10 | 57.13163562 | 162.5315742 | -2.283719287 | 2.283719287 | 2.09E-10 | 3.56E-09 | DOWN |
| Smim3 | 7.409625447 | 0.492670429 | 3.010413663 | 3.010413663 | 2.14E-10 | 3.63E-09 | UP |
| Osbpl6 | 1.255408133 | 0.056137993 | 3.557981351 | 3.557981351 | 2.15E-10 | 3.64E-09 | UP |
| Capn8 | 1.168034345 | 0 | 8.692385914 | 8.692385914 | 2.23E-10 | 3.78E-09 | UP |
| Camk2d | 8.485200892 | 0.984872085 | 2.214452134 | 2.214452134 | 2.30E-10 | 3.88E-09 | UP |
| Spp2 | 438.017943 | 994.4148815 | -2.01203478 | 2.01203478 | 2.33E-10 | 3.94E-09 | DOWN |
| Hunk | 1.189362879 | 0 | 7.993614759 | 7.993614759 | 2.34E-10 | 3.94E-09 | UP |
| Rcl1 | 21.49139592 | 52.11017381 | -2.062007942 | 2.062007942 | 2.41E-10 | 4.06E-09 | DOWN |
| Serpinb8 | 7.303102489 | 0.975888597 | 2.03490825 | 2.03490825 | 2.50E-10 | 4.21E-09 | UP |
| Eif5a2 | 2.663773408 | 0.150917832 | 3.232902636 | 3.232902636 | 2.53E-10 | 4.25E-09 | UP |
| Tdrp | 2.744976926 | 0.237016189 | 2.680659224 | 2.680659224 | 2.53E-10 | 4.25E-09 | UP |
| Cfb | 1.217820237 | 37.7361533 | -5.825736107 | 5.825736107 | 2.58E-10 | 4.33E-09 | DOWN |
| G0s2 | 39.25904795 | 283.4402594 | -3.5489091 | 3.5489091 | 2.64E-10 | 4.43E-09 | DOWN |
| Alpk1 | 3.972245945 | 0.522387118 | 2.102192081 | 2.102192081 | 2.69E-10 | 4.50E-09 | UP |
| Pbk | 3.777693311 | 0.234518387 | 3.148886119 | 3.148886119 | 2.81E-10 | 4.69E-09 | UP |
| Abhd14b | 60.92213225 | 140.8360402 | -2.003961706 | 2.003961706 | 2.87E-10 | 4.80E-09 | DOWN |
| Ebp | 42.09094017 | 101.1558005 | -2.056974909 | 2.056974909 | 2.92E-10 | 4.86E-09 | DOWN |
| Map1s | 6.2956744 | 0.598126277 | 2.544517236 | 2.544517236 | 2.95E-10 | 4.91E-09 | UP |
| Iqgap1 | 32.21960342 | 4.140302343 | 2.070858981 | 2.070858981 | 2.97E-10 | 4.95E-09 | UP |
| Cfap300 | 3.485304957 | 0.093937893 | 4.308008641 | 4.308008641 | 3.03E-10 | 5.03E-09 | UP |
| Fut2 | 3.883755321 | 0 | 10.05180605 | 10.05180605 | 3.05E-10 | 5.06E-09 | UP |
| Cachd1 | 2.876943404 | 0.160578637 | 3.314699873 | 3.314699873 | 3.07E-10 | 5.09E-09 | UP |
| Dennd2d | 9.567342022 | 0.987066825 | 2.399895807 | 2.399895807 | 3.13E-10 | 5.18E-09 | UP |
| Cenpw | 2.305869311 | 0.025375446 | 5.438680208 | 5.438680208 | 3.33E-10 | 5.50E-09 | UP |
| Egflam | 3.265705671 | 0.055684653 | 4.974016469 | 4.974016469 | 3.36E-10 | 5.54E-09 | UP |
| Cabp2 | 1.511524537 | 18.1025752 | -4.265622289 | 4.265622289 | 3.39E-10 | 5.60E-09 | DOWN |
| Tox3 | 3.470749128 | 0.174446378 | 3.434834288 | 3.434834288 | 3.45E-10 | 5.68E-09 | UP |
| Sdc2 | 122.1717332 | 278.3381666 | -2.003960244 | 2.003960244 | 3.63E-10 | 5.96E-09 | DOWN |
| Mthfd1l | 4.024438275 | 0.275167621 | 2.990718238 | 2.990718238 | 3.70E-10 | 6.07E-09 | UP |
| Samd11 | 1.312826586 | 4.243641403 | -2.514100813 | 2.514100813 | 3.70E-10 | 6.07E-09 | DOWN |
| Tshz2 | 6.264325482 | 0.791188453 | 2.137830646 | 2.137830646 | 3.79E-10 | 6.20E-09 | UP |
| Cma1 | 5.142481006 | 0.124893317 | 4.44185423 | 4.44185423 | 3.84E-10 | 6.28E-09 | UP |
| Stc1 | 2.107936746 | 0.117351386 | 3.295892379 | 3.295892379 | 3.88E-10 | 6.34E-09 | UP |
| Tmprss6 | 60.41421656 | 147.7046915 | -2.073178144 | 2.073178144 | 3.99E-10 | 6.51E-09 | DOWN |
| Alb | 14645.0823 | 35561.22308 | -2.071770624 | 2.071770624 | 4.05E-10 | 6.61E-09 | DOWN |
| S100b | 4.349333397 | 0.124687276 | 4.235539978 | 4.235539978 | 4.07E-10 | 6.63E-09 | UP |
| Rad51 | 1.690775165 | 0.03783521 | 4.581027642 | 4.581027642 | 4.11E-10 | 6.67E-09 | UP |
| Ltbp3 | 8.200190516 | 0.79766358 | 2.509504773 | 2.509504773 | 4.23E-10 | 6.86E-09 | UP |
| S100a9 | 23.98154106 | 1.668590642 | 2.984103924 | 2.984103924 | 4.45E-10 | 7.20E-09 | UP |
| Akr1c19 | 42.74260664 | 3.755839004 | 2.600379836 | 2.600379836 | 4.59E-10 | 7.40E-09 | UP |
| Stk26 | 3.012302413 | 0.140224568 | 3.506270111 | 3.506270111 | 4.77E-10 | 7.68E-09 | UP |
| Cyp2f4 | 6.557305525 | 17.57073708 | -2.191676561 | 2.191676561 | 4.80E-10 | 7.72E-09 | DOWN |
| Susd2 | 3.954480315 | 0.455550208 | 2.295124147 | 2.295124147 | 4.91E-10 | 7.90E-09 | UP |
| Ckap2l | 2.279096541 | 0.066507178 | 4.180994278 | 4.180994278 | 4.93E-10 | 7.92E-09 | UP |
| AABR07070117.1 | 16.34844459 | 40.4712069 | -2.096465248 | 2.096465248 | 5.03E-10 | 8.07E-09 | DOWN |
| Tcf24 | 0.370016634 | 3.914722568 | -4.175042854 | 4.175042854 | 5.39E-10 | 8.62E-09 | DOWN |
| Ttc26 | 3.259194436 | 0.25549787 | 2.80160818 | 2.80160818 | 5.40E-10 | 8.62E-09 | UP |
| Kif3a | 5.616086839 | 0.546927142 | 2.47419592 | 2.47419592 | 5.44E-10 | 8.67E-09 | UP |
| Gnmt | 20.54976819 | 186.2938286 | -3.863189933 | 3.863189933 | 5.51E-10 | 8.79E-09 | DOWN |
| Esco2 | 1.799569456 | 0.081258187 | 3.558720982 | 3.558720982 | 5.66E-10 | 9.02E-09 | UP |
| Fap | 1.229133827 | 0 | 8.130824976 | 8.130824976 | 5.80E-10 | 9.21E-09 | UP |
| Mastl | 1.517811567 | 0.07916006 | 3.361552416 | 3.361552416 | 5.87E-10 | 9.32E-09 | UP |
| Lrp8 | 2.115323373 | 0.074330425 | 3.929720722 | 3.929720722 | 5.94E-10 | 9.41E-09 | UP |
| Asgr1 | 66.10784563 | 179.7674084 | -2.21574132 | 2.21574132 | 5.97E-10 | 9.46E-09 | DOWN |
| F12 | 110.871923 | 315.1460321 | -2.273821331 | 2.273821331 | 6.00E-10 | 9.50E-09 | DOWN |
| Thbs2 | 8.236834189 | 0.720897226 | 2.59690929 | 2.59690929 | 6.05E-10 | 9.57E-09 | UP |
| Rgs10 | 18.4450082 | 1.456355894 | 2.794679166 | 2.794679166 | 6.07E-10 | 9.60E-09 | UP |
| Fhl3 | 10.21018573 | 0.953600173 | 2.523633415 | 2.523633415 | 6.16E-10 | 9.73E-09 | UP |
| Igsf9 | 1.454038528 | 0.055951184 | 3.788991575 | 3.788991575 | 6.63E-10 | 1.04E-08 | UP |
| Mme | 4.991998501 | 0.359254731 | 2.921787966 | 2.921787966 | 6.66E-10 | 1.05E-08 | UP |
| Creb3l3 | 121.3005549 | 280.927521 | -2.00012159 | 2.00012159 | 6.67E-10 | 1.05E-08 | DOWN |
| Pir | 39.64791882 | 4.401561892 | 2.300204659 | 2.300204659 | 6.72E-10 | 1.06E-08 | UP |
| Aspg | 7.02310696 | 60.41436054 | -3.793118877 | 3.793118877 | 6.77E-10 | 1.06E-08 | DOWN |
| Nudt11 | 2.984553163 | 0.073070386 | 4.412873243 | 4.412873243 | 6.85E-10 | 1.07E-08 | UP |
| Mpzl2 | 15.66783139 | 1.696438251 | 2.300305478 | 2.300305478 | 6.96E-10 | 1.09E-08 | UP |
| Etnppl | 8.004501607 | 53.53042677 | -3.518589587 | 3.518589587 | 7.00E-10 | 1.09E-08 | DOWN |
| Acta1 | 1.8339202 | 0 | 8.005775423 | 8.005775423 | 7.02E-10 | 1.10E-08 | UP |
| Rnase4 | 90.61957055 | 226.9175863 | -2.107720391 | 2.107720391 | 7.34E-10 | 1.14E-08 | DOWN |
| Stil | 1.248409616 | 0.042927603 | 3.981517871 | 3.981517871 | 7.40E-10 | 1.15E-08 | UP |
| Dlgap5 | 4.37471622 | 0.193674501 | 3.597395151 | 3.597395151 | 7.41E-10 | 1.15E-08 | UP |
| Klra2 | 2.68293657 | 0.145780808 | 3.296253631 | 3.296253631 | 7.54E-10 | 1.17E-08 | UP |
| Cd302 | 150.9646364 | 387.5279108 | -2.173362282 | 2.173362282 | 7.70E-10 | 1.20E-08 | DOWN |
| Rnf207 | 1.25957994 | 0.016374885 | 5.214021941 | 5.214021941 | 7.78E-10 | 1.21E-08 | UP |
| Slc10a4 | 1.998746379 | 0 | 8.027830496 | 8.027830496 | 7.83E-10 | 1.21E-08 | UP |
| Scml2 | 1.140143271 | 0.033013122 | 4.196081022 | 4.196081022 | 7.91E-10 | 1.22E-08 | UP |
| Tmem163 | 0.730732287 | 3.312894845 | -2.992035879 | 2.992035879 | 7.91E-10 | 1.22E-08 | DOWN |
| Slc25a33 | 5.340746821 | 12.7627851 | -2.057792175 | 2.057792175 | 7.93E-10 | 1.23E-08 | DOWN |
| Cyp2c6v1 | 56.82630544 | 239.4867165 | -2.860060778 | 2.860060778 | 8.00E-10 | 1.24E-08 | DOWN |
| Gpr34 | 6.4513621 | 0.203451033 | 4.098169102 | 4.098169102 | 8.39E-10 | 1.29E-08 | UP |
| Padi4 | 3.920222062 | 0 | 9.671794707 | 9.671794707 | 8.65E-10 | 1.33E-08 | UP |
| Bhlhe22 | 2.780882448 | 0.144072 | 3.390589347 | 3.390589347 | 8.69E-10 | 1.34E-08 | UP |
| Ncf1 | 13.6261251 | 1.685671813 | 2.13233932 | 2.13233932 | 9.15E-10 | 1.40E-08 | UP |
| LOC103691699 | 0.351878665 | 1.359036399 | -2.738098581 | 2.738098581 | 9.56E-10 | 1.47E-08 | DOWN |
| Usp43 | 1.10632431 | 0.032867901 | 4.153701684 | 4.153701684 | 9.76E-10 | 1.49E-08 | UP |
| Mcm10 | 2.018327435 | 5.008331608 | -2.105848096 | 2.105848096 | 1.03E-09 | 1.57E-08 | DOWN |
| Sesn2 | 8.836231996 | 21.49796294 | -2.09249343 | 2.09249343 | 1.05E-09 | 1.60E-08 | DOWN |
| LOC680875 | 0.661771362 | 6.06259558 | -3.91315069 | 3.91315069 | 1.09E-09 | 1.67E-08 | DOWN |
| Cxcl3 | 6.925125099 | 0 | 9.936661918 | 9.936661918 | 1.11E-09 | 1.69E-08 | UP |
| Nts | 4.366522383 | 0 | 9.367057709 | 9.367057709 | 1.15E-09 | 1.75E-08 | UP |
| Srpx2 | 6.773945468 | 0.132556518 | 4.785265434 | 4.785265434 | 1.16E-09 | 1.76E-08 | UP |
| Il1a | 1.445950649 | 5.517709355 | -2.755865635 | 2.755865635 | 1.16E-09 | 1.76E-08 | DOWN |
| Agt | 135.8687601 | 662.1756784 | -3.005620211 | 3.005620211 | 1.17E-09 | 1.77E-08 | DOWN |
| Ush1c | 2.509830512 | 0 | 9.005270746 | 9.005270746 | 1.21E-09 | 1.83E-08 | UP |
| Shcbp1 | 2.741311803 | 0.051196351 | 4.799536665 | 4.799536665 | 1.23E-09 | 1.86E-08 | UP |
| Edem1 | 93.59321947 | 216.9429075 | -2.01459818 | 2.01459818 | 1.25E-09 | 1.90E-08 | DOWN |
| Tenm3 | 2.818635085 | 0.259681503 | 2.567311653 | 2.567311653 | 1.26E-09 | 1.90E-08 | UP |
| Sult1c2 | 0.16143732 | 1.289887239 | -3.721891896 | 3.721891896 | 1.28E-09 | 1.93E-08 | DOWN |
| Paqr8 | 8.598413333 | 0.767135108 | 2.639454606 | 2.639454606 | 1.29E-09 | 1.94E-08 | UP |
| Heyl | 4.77464949 | 0.583754258 | 2.182171655 | 2.182171655 | 1.30E-09 | 1.96E-08 | UP |
| Entpd8 | 2.231984926 | 6.310580137 | -2.274713671 | 2.274713671 | 1.31E-09 | 1.97E-08 | DOWN |
| Kcnip4 | 2.189400443 | 0.069664705 | 4.057966779 | 4.057966779 | 1.31E-09 | 1.97E-08 | UP |
| Adcy2 | 1.29769191 | 0.019138887 | 5.062736795 | 5.062736795 | 1.36E-09 | 2.05E-08 | UP |
| Por | 81.26860544 | 195.7465871 | -2.047976171 | 2.047976171 | 1.38E-09 | 2.07E-08 | DOWN |
| MGC105649 | 43.90650594 | 0.83497125 | 4.741586597 | 4.741586597 | 1.38E-09 | 2.07E-08 | UP |
| LOC501038 | 0.537223979 | 5.175122168 | -4.012968397 | 4.012968397 | 1.39E-09 | 2.09E-08 | DOWN |
| Aspnl1 | 5.803720854 | 0.215801209 | 3.834825616 | 3.834825616 | 1.45E-09 | 2.17E-08 | UP |
| LOC108348139 | 15.35047394 | 2.082952658 | 2.029908879 | 2.029908879 | 1.46E-09 | 2.17E-08 | UP |
| Pik3c2b | 2.083413846 | 0.211771543 | 2.416595681 | 2.416595681 | 1.46E-09 | 2.17E-08 | UP |
| Clip3 | 1.004661205 | 0 | 7.759094556 | 7.759094556 | 1.55E-09 | 2.30E-08 | UP |
| Abca6 | 14.30798055 | 41.53595674 | -2.301018075 | 2.301018075 | 1.56E-09 | 2.31E-08 | DOWN |
| Dsn1 | 2.594762912 | 0.189972476 | 2.888357278 | 2.888357278 | 1.58E-09 | 2.34E-08 | UP |
| Igfbp4 | 260.9074971 | 691.3117939 | -2.175258136 | 2.175258136 | 1.58E-09 | 2.34E-08 | DOWN |
| Acox2 | 46.32394834 | 157.6603994 | -2.51270852 | 2.51270852 | 1.59E-09 | 2.35E-08 | DOWN |
| Syn2 | 1.256090995 | 0.015573543 | 5.337622501 | 5.337622501 | 1.60E-09 | 2.36E-08 | UP |
| Cys1 | 1.924748483 | 0.06889991 | 3.92478401 | 3.92478401 | 1.60E-09 | 2.36E-08 | UP |
| Zc3h12d | 1.857603958 | 0.12187979 | 3.062498254 | 3.062498254 | 1.62E-09 | 2.39E-08 | UP |
| AABR07032520.1 | 1.873834281 | 8.232641093 | -2.886073901 | 2.886073901 | 1.63E-09 | 2.41E-08 | DOWN |
| Crp | 317.8944327 | 1478.596255 | -2.958550219 | 2.958550219 | 1.67E-09 | 2.45E-08 | DOWN |
| Gltpd2 | 21.56998649 | 75.27561961 | -2.551634473 | 2.551634473 | 1.67E-09 | 2.45E-08 | DOWN |
| Obsl1 | 1.651106158 | 0.10623327 | 3.08372689 | 3.08372689 | 1.73E-09 | 2.54E-08 | UP |
| Cyp4f4 | 23.33953379 | 79.06266426 | -2.507653575 | 2.507653575 | 1.75E-09 | 2.56E-08 | DOWN |
| LOC108348128 | 1.213573285 | 0.014767159 | 5.423786782 | 5.423786782 | 1.77E-09 | 2.59E-08 | UP |
| Lilrb4 | 5.086054965 | 0.198167061 | 3.764947039 | 3.764947039 | 1.83E-09 | 2.67E-08 | UP |
| Dusp4 | 6.405028021 | 0.354920591 | 3.237288805 | 3.237288805 | 1.86E-09 | 2.71E-08 | UP |
| Cdca2 | 1.862281506 | 0.101276302 | 3.360352844 | 3.360352844 | 1.87E-09 | 2.73E-08 | UP |
| Lbh | 13.35493814 | 0.975074096 | 2.93111057 | 2.93111057 | 1.87E-09 | 2.73E-08 | UP |
| Gcat | 10.71004727 | 28.49883278 | -2.187973948 | 2.187973948 | 1.90E-09 | 2.76E-08 | DOWN |
| Vwa2 | 2.276642915 | 0.037051568 | 4.995473128 | 4.995473128 | 1.91E-09 | 2.77E-08 | UP |
| Mt1 | 86.45435793 | 486.0299259 | -3.224521314 | 3.224521314 | 1.92E-09 | 2.78E-08 | DOWN |
| Hao1 | 59.0787207 | 208.7275326 | -2.568930042 | 2.568930042 | 1.98E-09 | 2.86E-08 | DOWN |
| Meiob | 0.15285576 | 1.198572879 | -3.770726883 | 3.770726883 | 1.99E-09 | 2.87E-08 | DOWN |
| Thrsp | 18.45596425 | 78.7040266 | -2.84584505 | 2.84584505 | 2.00E-09 | 2.88E-08 | DOWN |
| Evi2b | 11.75222194 | 1.350300882 | 2.255506475 | 2.255506475 | 2.02E-09 | 2.91E-08 | UP |
| Limch1 | 3.373971984 | 0.282441039 | 2.765057836 | 2.765057836 | 2.05E-09 | 2.94E-08 | UP |
| Arhgef6 | 8.591690574 | 1.026844943 | 2.188939822 | 2.188939822 | 2.05E-09 | 2.94E-08 | UP |
| Spag5 | 1.930920192 | 0.099321056 | 3.367737206 | 3.367737206 | 2.05E-09 | 2.94E-08 | UP |
| RGD1559459 | 50.64271183 | 6.310294007 | 2.178746445 | 2.178746445 | 2.10E-09 | 3.00E-08 | UP |
| Zfp367 | 2.561740885 | 0.24077291 | 2.55762641 | 2.55762641 | 2.11E-09 | 3.03E-08 | UP |
| Mapk13 | 4.507421232 | 0.098849657 | 4.582153976 | 4.582153976 | 2.15E-09 | 3.07E-08 | UP |
| Ttll5 | 3.115102392 | 0.346817741 | 2.306613592 | 2.306613592 | 2.16E-09 | 3.10E-08 | UP |
| Cited1 | 4.126199588 | 0.066273114 | 4.991674586 | 4.991674586 | 2.20E-09 | 3.14E-08 | UP |
| Slc7a6 | 4.164481229 | 0.37543769 | 2.569195347 | 2.569195347 | 2.21E-09 | 3.15E-08 | UP |
| Unc93a | 1.970733557 | 0.014810282 | 5.90809747 | 5.90809747 | 2.24E-09 | 3.19E-08 | UP |
| Tspo | 56.97042947 | 4.312777361 | 2.809250926 | 2.809250926 | 2.27E-09 | 3.23E-08 | UP |
| Irx3 | 2.38669501 | 0 | 8.614749549 | 8.614749549 | 2.37E-09 | 3.37E-08 | UP |
| Mmp23 | 6.226883123 | 0.50506897 | 2.74396206 | 2.74396206 | 2.38E-09 | 3.37E-08 | UP |
| LOC100361492 | 47.10989235 | 1.147769109 | 4.501041342 | 4.501041342 | 2.47E-09 | 3.49E-08 | UP |
| Mettl7b | 106.3606934 | 262.9470711 | -2.079471516 | 2.079471516 | 2.50E-09 | 3.54E-08 | DOWN |
| Fam84a | 1.111457322 | 0.038190285 | 3.981161209 | 3.981161209 | 2.57E-09 | 3.63E-08 | UP |
| Spc25 | 4.815575481 | 0.22806552 | 3.512158029 | 3.512158029 | 2.62E-09 | 3.69E-08 | UP |
| C8b | 90.38612263 | 229.3028235 | -2.120716569 | 2.120716569 | 2.68E-09 | 3.76E-08 | DOWN |
| Capn6 | 2.230007578 | 0.064039112 | 4.257251902 | 4.257251902 | 2.69E-09 | 3.78E-08 | UP |
| Tuft1 | 2.359872331 | 0.197994863 | 2.680513249 | 2.680513249 | 2.71E-09 | 3.79E-08 | UP |
| Gpbar1 | 2.219408907 | 0 | 7.754593077 | 7.754593077 | 2.72E-09 | 3.81E-08 | UP |
| Gpm6b | 6.044302601 | 0.410852 | 2.973520756 | 2.973520756 | 2.80E-09 | 3.92E-08 | UP |
| Cpm | 3.417592672 | 0.450594003 | 2.061207075 | 2.061207075 | 2.83E-09 | 3.95E-08 | UP |
| Pcyt2 | 38.33606565 | 89.61348471 | -2.003444491 | 2.003444491 | 2.88E-09 | 4.01E-08 | DOWN |
| Ctla2a | 30.63819443 | 3.960850837 | 2.072764746 | 2.072764746 | 2.88E-09 | 4.01E-08 | UP |
| Psca | 11.89883524 | 0 | 8.905353888 | 8.905353888 | 2.94E-09 | 4.09E-08 | UP |
| Ch25h | 5.634375137 | 0.435115297 | 2.814654907 | 2.814654907 | 3.10E-09 | 4.30E-08 | UP |
| LOC103690070 | 9.633419643 | 0.058024 | 6.348965281 | 6.348965281 | 3.12E-09 | 4.33E-08 | UP |
| Duox2 | 7.482751407 | 0.007313085 | 8.87923773 | 8.87923773 | 3.19E-09 | 4.42E-08 | UP |
| Inhbe | 29.0110392 | 157.6576127 | -3.157473577 | 3.157473577 | 3.25E-09 | 4.49E-08 | DOWN |
| Ltc4s | 1.766013919 | 18.25016788 | -4.123500838 | 4.123500838 | 3.31E-09 | 4.56E-08 | DOWN |
| Slc25a15 | 87.1206142 | 264.0510449 | -2.3866079 | 2.3866079 | 3.48E-09 | 4.79E-08 | DOWN |
| Mecr | 6.222014863 | 16.14751108 | -2.148442106 | 2.148442106 | 3.58E-09 | 4.92E-08 | DOWN |
| LOC685680 | 1.479266382 | 0 | 7.658641583 | 7.658641583 | 3.71E-09 | 5.08E-08 | UP |
| Uhrf1 | 2.121630067 | 0.145003726 | 2.99784411 | 2.99784411 | 3.71E-09 | 5.08E-08 | UP |
| Mall | 4.581826138 | 0.023263673 | 6.515176224 | 6.515176224 | 3.82E-09 | 5.22E-08 | UP |
| Adgrg3 | 1.423402351 | 0.045960687 | 4.039520711 | 4.039520711 | 3.83E-09 | 5.23E-08 | UP |
| Pttg1 | 5.972746945 | 0.276089723 | 3.551738217 | 3.551738217 | 3.86E-09 | 5.26E-08 | UP |
| Aspdh | 7.483379616 | 39.28930627 | -3.097049423 | 3.097049423 | 3.88E-09 | 5.29E-08 | DOWN |
| Cbr3 | 14.29668756 | 0.65734432 | 3.542361942 | 3.542361942 | 4.01E-09 | 5.45E-08 | UP |
| Slamf9 | 7.819488661 | 0.69010595 | 2.608558985 | 2.608558985 | 4.05E-09 | 5.49E-08 | UP |
| Gprc5a | 6.901241961 | 0.058787501 | 5.872142086 | 5.872142086 | 4.10E-09 | 5.56E-08 | UP |
| Aard | 2.355900868 | 0 | 8.139265304 | 8.139265304 | 4.23E-09 | 5.70E-08 | UP |
| Vstm4 | 4.193524959 | 0.320364643 | 2.846123148 | 2.846123148 | 4.23E-09 | 5.70E-08 | UP |
| Itih3 | 346.1920558 | 1047.020778 | -2.362388335 | 2.362388335 | 4.37E-09 | 5.88E-08 | DOWN |
| Gxylt2 | 2.289124949 | 0.030431527 | 5.158285657 | 5.158285657 | 4.55E-09 | 6.12E-08 | UP |
| Cabcoco1 | 4.999724166 | 0.350253809 | 2.969161649 | 2.969161649 | 4.56E-09 | 6.13E-08 | UP |
| Zfp36 | 34.40269231 | 81.7496279 | -2.069730078 | 2.069730078 | 4.57E-09 | 6.13E-08 | DOWN |
| Rxrg | 3.178581902 | 7.754851103 | -2.067304388 | 2.067304388 | 4.58E-09 | 6.15E-08 | DOWN |
| Ckap4 | 21.60931763 | 1.061119269 | 3.538149185 | 3.538149185 | 4.80E-09 | 6.42E-08 | UP |
| Tmprss2 | 10.7364513 | 0.751872521 | 2.905610951 | 2.905610951 | 4.81E-09 | 6.43E-08 | UP |
| Lypd1 | 8.3480816 | 0.92871052 | 2.295633395 | 2.295633395 | 4.83E-09 | 6.46E-08 | UP |
| Cdkn2c | 5.863910049 | 0.486976959 | 2.72064604 | 2.72064604 | 4.89E-09 | 6.52E-08 | UP |
| Shroom4 | 2.116109168 | 0.259397931 | 2.186467552 | 2.186467552 | 5.04E-09 | 6.72E-08 | UP |
| Blnk | 8.09692565 | 0.535677762 | 3.05032189 | 3.05032189 | 5.13E-09 | 6.83E-08 | UP |
| LOC691083 | 3.632360234 | 12.21727295 | -2.511455136 | 2.511455136 | 5.20E-09 | 6.91E-08 | DOWN |
| Galnt7 | 2.674846027 | 0.242865538 | 2.573949774 | 2.573949774 | 5.47E-09 | 7.24E-08 | UP |
| LOC100360143 | 0 | 3.271639939 | -7.469399328 | 7.469399328 | 5.72E-09 | 7.54E-08 | DOWN |
| Cyp1a1 | 7.99629503 | 0.273137786 | 4.136460892 | 4.136460892 | 5.74E-09 | 7.56E-08 | UP |
| Cpb2 | 161.8964031 | 398.0010156 | -2.092647565 | 2.092647565 | 5.95E-09 | 7.82E-08 | DOWN |
| N4bp3 | 1.90303809 | 0.112184125 | 3.222435786 | 3.222435786 | 6.11E-09 | 8.01E-08 | UP |
| Bcat1 | 2.330673712 | 0.053775767 | 4.454045472 | 4.454045472 | 6.23E-09 | 8.16E-08 | UP |
| Arhgap44 | 2.899993408 | 0.171204444 | 3.219143681 | 3.219143681 | 6.27E-09 | 8.20E-08 | UP |
| Cdc45 | 1.656553178 | 0.070538945 | 3.615850325 | 3.615850325 | 6.31E-09 | 8.25E-08 | UP |
| Cbs | 13.31093015 | 93.85432283 | -3.513854962 | 3.513854962 | 6.42E-09 | 8.38E-08 | DOWN |
| Car5a | 7.820488259 | 27.29404915 | -2.550120703 | 2.550120703 | 6.51E-09 | 8.49E-08 | DOWN |
| Ftcd | 80.70050956 | 222.7282552 | -2.226370852 | 2.226370852 | 6.64E-09 | 8.64E-08 | DOWN |
| Gcdh | 67.27301424 | 161.4261615 | -2.039496631 | 2.039496631 | 6.78E-09 | 8.82E-08 | DOWN |
| Dok4 | 5.24985162 | 0.485040178 | 2.582313503 | 2.582313503 | 6.84E-09 | 8.89E-08 | UP |
| Hacd4 | 8.156675886 | 0.611623588 | 2.848649665 | 2.848649665 | 7.08E-09 | 9.19E-08 | UP |
| Nudt8 | 6.250243129 | 16.82911597 | -2.259635994 | 2.259635994 | 7.28E-09 | 9.44E-08 | DOWN |
| Apbb1ip | 7.433747041 | 0.970453174 | 2.060593971 | 2.060593971 | 7.43E-09 | 9.61E-08 | UP |
| Ppm1k | 15.64009547 | 42.76147514 | -2.244254067 | 2.244254067 | 7.57E-09 | 9.79E-08 | DOWN |
| Mal | 3.535621305 | 0.26177412 | 2.908557395 | 2.908557395 | 7.65E-09 | 9.88E-08 | UP |
| Melk | 2.051740327 | 0.0574055 | 4.201548527 | 4.201548527 | 7.67E-09 | 9.89E-08 | UP |
| Sfxn2 | 0.768284469 | 1.901873845 | -2.143656106 | 2.143656106 | 7.67E-09 | 9.89E-08 | DOWN |
| Slc16a10 | 5.673533029 | 20.07536721 | -2.589149335 | 2.589149335 | 7.71E-09 | 9.93E-08 | DOWN |
| Mdfi | 2.697021233 | 0.089291722 | 4.020299911 | 4.020299911 | 7.84E-09 | 1.01E-07 | UP |
| Slc7a2 | 63.53213812 | 273.1464325 | -2.835218528 | 2.835218528 | 7.94E-09 | 1.02E-07 | DOWN |
| Rgs2 | 32.64366154 | 3.791944786 | 2.220507795 | 2.220507795 | 8.05E-09 | 1.03E-07 | UP |
| Ambp | 912.9101196 | 2765.806157 | -2.350852243 | 2.350852243 | 8.13E-09 | 1.04E-07 | DOWN |
| Efhd1 | 8.678765928 | 0.888071692 | 2.413561847 | 2.413561847 | 8.34E-09 | 1.07E-07 | UP |
| Cyp2j10 | 11.97914568 | 32.47081198 | -2.229817379 | 2.229817379 | 8.65E-09 | 1.10E-07 | DOWN |
| Pimreg | 1.274473155 | 0 | 7.512869921 | 7.512869921 | 8.86E-09 | 1.13E-07 | UP |
| Glis2 | 5.997637736 | 0.487623837 | 2.784876636 | 2.784876636 | 9.17E-09 | 1.17E-07 | UP |
| Slc30a10 | 8.30056574 | 28.49879326 | -2.531419115 | 2.531419115 | 9.79E-09 | 1.24E-07 | DOWN |
| Khk | 30.75997391 | 105.3317807 | -2.512902616 | 2.512902616 | 9.96E-09 | 1.26E-07 | DOWN |
| Abcb11 | 38.34946456 | 110.3221612 | -2.280399833 | 2.280399833 | 9.99E-09 | 1.26E-07 | DOWN |
| Spink1l | 88.16536799 | 270.0217657 | -2.361312313 | 2.361312313 | 1.00E-08 | 1.26E-07 | DOWN |
| Hes6 | 2.834139652 | 7.160242081 | -2.147929284 | 2.147929284 | 1.00E-08 | 1.27E-07 | DOWN |
| Lzts2 | 4.074062981 | 0.523162052 | 2.095973933 | 2.095973933 | 1.01E-08 | 1.28E-07 | UP |
| Rnf208 | 2.026331399 | 0 | 7.81434151 | 7.81434151 | 1.03E-08 | 1.30E-07 | UP |
| Mecom | 4.80220876 | 0.424528639 | 2.600090031 | 2.600090031 | 1.05E-08 | 1.33E-07 | UP |
| Fgd3 | 3.610185262 | 0.444080524 | 2.177792023 | 2.177792023 | 1.05E-08 | 1.33E-07 | UP |
| Cdc25c | 2.732183838 | 0.228128417 | 2.706005546 | 2.706005546 | 1.06E-08 | 1.33E-07 | UP |
| Atf7ip2 | 1.962808476 | 0 | 7.807484862 | 7.807484862 | 1.06E-08 | 1.33E-07 | UP |
| Trip13 | 2.129965206 | 0.087632421 | 3.683482824 | 3.683482824 | 1.07E-08 | 1.34E-07 | UP |
| Arhgap19 | 1.857291052 | 0.182169364 | 2.48524027 | 2.48524027 | 1.10E-08 | 1.38E-07 | UP |
| F11 | 27.30307838 | 73.72934655 | -2.215753985 | 2.215753985 | 1.10E-08 | 1.38E-07 | DOWN |
| Notch3 | 4.409015008 | 0.355958474 | 2.765078686 | 2.765078686 | 1.16E-08 | 1.45E-07 | UP |
| RGD1563354 | 2.161564053 | 0.131019941 | 3.138170796 | 3.138170796 | 1.18E-08 | 1.48E-07 | UP |
| Ddah2 | 5.545988364 | 0.486891323 | 2.653191225 | 2.653191225 | 1.20E-08 | 1.50E-07 | UP |
| Tnc | 5.139408355 | 0.03132068 | 6.360064328 | 6.360064328 | 1.21E-08 | 1.51E-07 | UP |
| Rcn1 | 43.94334733 | 4.884938295 | 2.357788703 | 2.357788703 | 1.21E-08 | 1.51E-07 | UP |
| Antxr1 | 7.004590044 | 0.897350142 | 2.07666356 | 2.07666356 | 1.23E-08 | 1.53E-07 | UP |
| Selenom | 18.71112478 | 1.730459461 | 2.589363131 | 2.589363131 | 1.25E-08 | 1.55E-07 | UP |
| Trem2 | 1.927827614 | 0 | 7.754765751 | 7.754765751 | 1.26E-08 | 1.57E-07 | UP |
| Ddrgk1 | 9.696202256 | 31.06785068 | -2.433307736 | 2.433307736 | 1.29E-08 | 1.61E-07 | DOWN |
| Slc1a5 | 5.710305448 | 0.320965265 | 3.289710017 | 3.289710017 | 1.31E-08 | 1.62E-07 | UP |
| Rundc3a | 2.48226649 | 0.064500004 | 4.319049854 | 4.319049854 | 1.31E-08 | 1.62E-07 | UP |
| RGD1311946 | 6.192643327 | 0.489750142 | 2.80095141 | 2.80095141 | 1.31E-08 | 1.62E-07 | UP |
| Tfr2 | 40.00892018 | 111.084776 | -2.227305089 | 2.227305089 | 1.31E-08 | 1.63E-07 | DOWN |
| Clec1b | 1.750948359 | 6.395637571 | -2.648900863 | 2.648900863 | 1.32E-08 | 1.63E-07 | DOWN |
| Ndufv3 | 11.51061214 | 31.04028013 | -2.215548949 | 2.215548949 | 1.35E-08 | 1.66E-07 | DOWN |
| Cyp8b1 | 163.6365236 | 530.1506671 | -2.455840118 | 2.455840118 | 1.36E-08 | 1.67E-07 | DOWN |
| Ifit1bl | 3.64017184 | 0.310575088 | 2.670008433 | 2.670008433 | 1.36E-08 | 1.68E-07 | UP |
| RGD1564614 | 36.24727178 | 94.7632994 | -2.169142152 | 2.169142152 | 1.40E-08 | 1.72E-07 | DOWN |
| Crlf1 | 4.675648129 | 0.048702567 | 5.554249329 | 5.554249329 | 1.40E-08 | 1.72E-07 | UP |
| Tekt1 | 1.929917149 | 0 | 8.07509371 | 8.07509371 | 1.44E-08 | 1.78E-07 | UP |
| Ube2c | 2.631147504 | 0.109895961 | 3.653266753 | 3.653266753 | 1.47E-08 | 1.81E-07 | UP |
| Trabd2b | 1.756149462 | 0.013339558 | 5.870409223 | 5.870409223 | 1.50E-08 | 1.85E-07 | UP |
| Chi3l1 | 4.424666163 | 0.313760738 | 2.911871443 | 2.911871443 | 1.55E-08 | 1.91E-07 | UP |
| Akr1c3 | 0.382146918 | 6.37585317 | -4.725293077 | 4.725293077 | 1.57E-08 | 1.92E-07 | DOWN |
| Ms4a8 | 23.39911351 | 0.01751876 | 9.078175173 | 9.078175173 | 1.58E-08 | 1.94E-07 | UP |
| Fcgbp | 1.259793393 | 0.0180022 | 5.19034634 | 5.19034634 | 1.66E-08 | 2.03E-07 | UP |
| Dusp18 | 4.991736701 | 0.647151507 | 2.07055019 | 2.07055019 | 1.71E-08 | 2.08E-07 | UP |
| Rrm2 | 4.793546959 | 0.208454808 | 3.645667673 | 3.645667673 | 1.75E-08 | 2.13E-07 | UP |
| Phf19 | 0.934802302 | 3.048349919 | -2.556957655 | 2.556957655 | 1.75E-08 | 2.13E-07 | DOWN |
| Sgcb | 6.35712734 | 0.526858295 | 2.69739942 | 2.69739942 | 1.79E-08 | 2.17E-07 | UP |
| Gas7 | 2.509794994 | 0.109464091 | 3.638050672 | 3.638050672 | 1.80E-08 | 2.18E-07 | UP |
| Kcne3 | 2.691742531 | 0 | 8.209439522 | 8.209439522 | 1.83E-08 | 2.22E-07 | UP |
| A1bg | 4.337634179 | 0.143632945 | 4.049556767 | 4.049556767 | 1.86E-08 | 2.25E-07 | UP |
| Cpn2 | 34.36309702 | 99.97497334 | -2.288106586 | 2.288106586 | 1.91E-08 | 2.31E-07 | DOWN |
| Ak4 | 14.4684456 | 39.36446806 | -2.192032232 | 2.192032232 | 1.92E-08 | 2.31E-07 | DOWN |
| Fcer1a | 2.105371772 | 0.044221559 | 4.600050188 | 4.600050188 | 1.99E-08 | 2.40E-07 | UP |
| Elf3 | 15.30142281 | 1.049533258 | 2.959857284 | 2.959857284 | 2.01E-08 | 2.41E-07 | UP |
| Plekhs1 | 3.271682303 | 0.202916894 | 3.163305861 | 3.163305861 | 2.02E-08 | 2.42E-07 | UP |
| Serpina3m | 39.66285081 | 244.1037983 | -3.318935766 | 3.318935766 | 2.05E-08 | 2.46E-07 | DOWN |
| Tff1 | 39.37333854 | 0 | 10.68785338 | 10.68785338 | 2.07E-08 | 2.48E-07 | UP |
| Faah | 9.426939424 | 27.4622275 | -2.293940075 | 2.293940075 | 2.11E-08 | 2.52E-07 | DOWN |
| Numbl | 1.877538817 | 0.094396429 | 3.457051677 | 3.457051677 | 2.19E-08 | 2.62E-07 | UP |
| Tacstd2 | 1.784749898 | 0 | 8.168741771 | 8.168741771 | 2.22E-08 | 2.65E-07 | UP |
| Col4a3 | 1.452163225 | 0.165930925 | 2.270144484 | 2.270144484 | 2.26E-08 | 2.68E-07 | UP |
| Agmat | 21.14442253 | 51.49136952 | -2.045761822 | 2.045761822 | 2.26E-08 | 2.69E-07 | DOWN |
| Alox15 | 42.39335388 | 0.537293641 | 5.475171119 | 5.475171119 | 2.28E-08 | 2.70E-07 | UP |
| Mpv17l | 0.36354419 | 2.25097742 | -3.353382681 | 3.353382681 | 2.36E-08 | 2.79E-07 | DOWN |
| Piezo2 | 1.251198919 | 0.105344 | 2.690140046 | 2.690140046 | 2.37E-08 | 2.81E-07 | UP |
| Ctrc | 2.105930661 | 0.048128527 | 4.467064392 | 4.467064392 | 2.45E-08 | 2.89E-07 | UP |
| Dhtkd1 | 28.64409646 | 74.87730293 | -2.159424353 | 2.159424353 | 2.45E-08 | 2.90E-07 | DOWN |
| Ly75 | 3.228140173 | 0.066750446 | 4.634097365 | 4.634097365 | 2.56E-08 | 3.02E-07 | UP |
| Phgdh | 17.40799399 | 1.074220754 | 3.15022766 | 3.15022766 | 2.65E-08 | 3.13E-07 | UP |
| F9 | 204.9666637 | 479.4848309 | -2.024837193 | 2.024837193 | 2.73E-08 | 3.22E-07 | DOWN |
| Clba1 | 2.908724048 | 0.176928401 | 3.191190599 | 3.191190599 | 2.83E-08 | 3.32E-07 | UP |
| Mat1a | 256.5948282 | 907.0345972 | -2.570816866 | 2.570816866 | 2.87E-08 | 3.37E-07 | DOWN |
| Foxa3 | 7.06953322 | 17.5188097 | -2.066546992 | 2.066546992 | 2.92E-08 | 3.42E-07 | DOWN |
| Inmt | 0.132983794 | 10.99145109 | -7.132514454 | 7.132514454 | 2.99E-08 | 3.49E-07 | DOWN |
| Ces4a | 1.799531322 | 4.344209377 | -2.077302704 | 2.077302704 | 2.99E-08 | 3.50E-07 | DOWN |
| Galnt12 | 5.697509173 | 0.236235568 | 3.725353534 | 3.725353534 | 3.00E-08 | 3.50E-07 | UP |
| Cd180 | 7.099471886 | 0.8116166 | 2.292676254 | 2.292676254 | 3.02E-08 | 3.52E-07 | UP |
| Mtcl1 | 1.187756446 | 0.022363528 | 4.80699847 | 4.80699847 | 3.04E-08 | 3.54E-07 | UP |
| LOC360919 | 22.25305538 | 74.78748751 | -2.506567035 | 2.506567035 | 3.11E-08 | 3.62E-07 | DOWN |
| Smpd3 | 10.35506671 | 1.071039568 | 2.386097852 | 2.386097852 | 3.13E-08 | 3.64E-07 | UP |
| Fkbp2 | 19.24128384 | 54.94155935 | -2.288968785 | 2.288968785 | 3.23E-08 | 3.75E-07 | DOWN |
| Gpx8 | 9.678494854 | 1.041964898 | 2.33935129 | 2.33935129 | 3.34E-08 | 3.87E-07 | UP |
| Cenpi | 1.345288687 | 0.047603442 | 3.889343909 | 3.889343909 | 3.53E-08 | 4.07E-07 | UP |
| Uchl1 | 2.291610715 | 0 | 7.880112489 | 7.880112489 | 3.55E-08 | 4.09E-07 | UP |
| Syt17 | 1.177195454 | 0.052278837 | 3.62472654 | 3.62472654 | 3.57E-08 | 4.12E-07 | UP |
| Chst14 | 6.027536639 | 0.687895051 | 2.294206682 | 2.294206682 | 3.58E-08 | 4.13E-07 | UP |
| AABR07006269.1 | 6.30644124 | 0.302169543 | 3.458022507 | 3.458022507 | 3.60E-08 | 4.14E-07 | UP |
| Cacnb3 | 4.051218031 | 0.251773422 | 3.155125541 | 3.155125541 | 3.61E-08 | 4.16E-07 | UP |
| Gc | 2242.203906 | 5904.215944 | -2.170554016 | 2.170554016 | 3.62E-08 | 4.17E-07 | DOWN |
| Serpinc1 | 241.9283281 | 637.6126244 | -2.16416026 | 2.16416026 | 3.63E-08 | 4.17E-07 | DOWN |
| Miga1 | 1.646236786 | 0.064743708 | 3.766465657 | 3.766465657 | 3.72E-08 | 4.27E-07 | UP |
| Tead4 | 2.92383051 | 0.347516127 | 2.238273713 | 2.238273713 | 3.83E-08 | 4.37E-07 | UP |
| Rexo5 | 1.734316198 | 0.136502181 | 2.791151163 | 2.791151163 | 3.83E-08 | 4.37E-07 | UP |
| Runx1 | 3.140379566 | 0.307836065 | 2.468350823 | 2.468350823 | 4.10E-08 | 4.66E-07 | UP |
| Dzip1l | 2.234877542 | 0.110758547 | 3.494580897 | 3.494580897 | 4.20E-08 | 4.77E-07 | UP |
| Gab2 | 6.17529169 | 0.61329649 | 2.483851268 | 2.483851268 | 4.25E-08 | 4.82E-07 | UP |
| Tm4sf20 | 8.765886755 | 0.013290034 | 8.02961738 | 8.02961738 | 4.34E-08 | 4.92E-07 | UP |
| Klkb1 | 46.77269193 | 121.861278 | -2.158685293 | 2.158685293 | 4.38E-08 | 4.95E-07 | DOWN |
| Serpina11 | 21.84040422 | 67.43653019 | -2.367070626 | 2.367070626 | 4.43E-08 | 5.01E-07 | DOWN |
| Tpbg | 3.510902386 | 0.385958723 | 2.293994761 | 2.293994761 | 4.46E-08 | 5.03E-07 | UP |
| Cd74 | 824.6551643 | 100.5442817 | 2.186703755 | 2.186703755 | 4.48E-08 | 5.05E-07 | UP |
| Elmo1 | 7.643515764 | 0.977327017 | 2.10533816 | 2.10533816 | 4.54E-08 | 5.11E-07 | UP |
| Spata2L | 1.189795563 | 3.480742845 | -2.330357885 | 2.330357885 | 4.62E-08 | 5.18E-07 | DOWN |
| Fam213b | 5.118558314 | 12.38145842 | -2.070473048 | 2.070473048 | 4.70E-08 | 5.27E-07 | DOWN |
| Slc38a4 | 129.0587647 | 440.9077973 | -2.538326423 | 2.538326423 | 4.86E-08 | 5.43E-07 | DOWN |
| Lmnb1 | 7.074317452 | 0.914156538 | 2.097634796 | 2.097634796 | 4.92E-08 | 5.50E-07 | UP |
| Wdhd1 | 2.454316009 | 0.296098148 | 2.181446183 | 2.181446183 | 4.95E-08 | 5.53E-07 | UP |
| Vtn | 435.5322545 | 1550.011257 | -2.560624097 | 2.560624097 | 5.01E-08 | 5.58E-07 | DOWN |
| Mt1m | 41.24045307 | 159.561219 | -2.73267997 | 2.73267997 | 5.06E-08 | 5.63E-07 | DOWN |
| Palm2 | 0.639526338 | 3.259421998 | -3.08672409 | 3.08672409 | 5.16E-08 | 5.73E-07 | DOWN |
| Fam110c | 5.603222587 | 0.757056139 | 2.001670154 | 2.001670154 | 5.24E-08 | 5.82E-07 | UP |
| Abo | 4.275449426 | 0.306942319 | 2.934345564 | 2.934345564 | 5.39E-08 | 5.97E-07 | UP |
| Nradd | 1.998822516 | 0.034440309 | 4.84350258 | 4.84350258 | 5.41E-08 | 5.99E-07 | UP |
| Slc15a2 | 1.452825042 | 0.102309901 | 2.981540848 | 2.981540848 | 5.43E-08 | 6.01E-07 | UP |
| Kif3c | 2.760150718 | 0.209383013 | 2.823386773 | 2.823386773 | 5.49E-08 | 6.07E-07 | UP |
| Slc1a1 | 1.271567891 | 0 | 8.845651164 | 8.845651164 | 5.50E-08 | 6.07E-07 | UP |
| Cyp4f5 | 1.362085871 | 0.119018658 | 2.689625209 | 2.689625209 | 5.58E-08 | 6.16E-07 | UP |
| Ntng2 | 3.765234271 | 0.48158615 | 2.095278021 | 2.095278021 | 5.81E-08 | 6.40E-07 | UP |
| Krt80 | 1.354865742 | 0.006629088 | 6.395459857 | 6.395459857 | 5.88E-08 | 6.47E-07 | UP |
| Mcph1 | 2.01379415 | 0.168328947 | 2.697625232 | 2.697625232 | 6.18E-08 | 6.77E-07 | UP |
| AABR07054614.1 | 9.437142565 | 1.028777911 | 2.319016901 | 2.319016901 | 6.25E-08 | 6.83E-07 | UP |
| Tesc | 4.389240772 | 0 | 8.065148197 | 8.065148197 | 6.26E-08 | 6.84E-07 | UP |
| Brca2 | 1.155602149 | 0.156502538 | 2.042428778 | 2.042428778 | 6.28E-08 | 6.85E-07 | UP |
| Upp2 | 2.431075096 | 12.42843722 | -3.122087108 | 3.122087108 | 6.47E-08 | 7.04E-07 | DOWN |
| Fam83d | 1.462704118 | 0.038112447 | 4.385914917 | 4.385914917 | 6.73E-08 | 7.32E-07 | UP |
| Mad2l1 | 8.99810777 | 0.991175998 | 2.293161365 | 2.293161365 | 6.74E-08 | 7.32E-07 | UP |
| Cdh6 | 1.481133016 | 0 | 8.39699976 | 8.39699976 | 6.93E-08 | 7.52E-07 | UP |
| Ano9 | 1.702627076 | 0.027578261 | 4.921993023 | 4.921993023 | 6.96E-08 | 7.55E-07 | UP |
| Marveld1 | 28.32504167 | 3.698536431 | 2.068079532 | 2.068079532 | 7.11E-08 | 7.70E-07 | UP |
| AABR07067024.1 | 8.545281205 | 0.372097177 | 3.638469073 | 3.638469073 | 7.12E-08 | 7.71E-07 | UP |
| Psat1 | 9.736764829 | 0.946689147 | 2.492964032 | 2.492964032 | 7.16E-08 | 7.74E-07 | UP |
| Erich5 | 7.736091954 | 0.467322429 | 3.213228185 | 3.213228185 | 7.19E-08 | 7.77E-07 | UP |
| Glis3 | 3.810184621 | 0.101258451 | 4.371041986 | 4.371041986 | 7.20E-08 | 7.77E-07 | UP |
| Lepr | 0.283539934 | 2.21275116 | -3.887203542 | 3.887203542 | 7.25E-08 | 7.82E-07 | DOWN |
| Aass | 25.66453515 | 147.5505427 | -3.241346078 | 3.241346078 | 7.26E-08 | 7.83E-07 | DOWN |
| Enkur | 1.561940948 | 0.040154384 | 4.304894038 | 4.304894038 | 7.33E-08 | 7.90E-07 | UP |
| Gpr39 | 12.84923915 | 1.667258985 | 2.082221517 | 2.082221517 | 7.42E-08 | 7.98E-07 | UP |
| RGD1562392 | 0.564631419 | 2.189205745 | -2.697179335 | 2.697179335 | 7.46E-08 | 8.02E-07 | DOWN |
| LOC103694872 | 7.230990157 | 24.44266813 | -2.499428061 | 2.499428061 | 7.50E-08 | 8.06E-07 | DOWN |
| Prodh2 | 10.01697356 | 43.25236394 | -2.816475837 | 2.816475837 | 7.76E-08 | 8.32E-07 | DOWN |
| Gpx7 | 10.92760289 | 1.281958026 | 2.217597869 | 2.217597869 | 7.82E-08 | 8.38E-07 | UP |
| Rapgef3 | 2.363352599 | 0.182473169 | 2.863712398 | 2.863712398 | 7.85E-08 | 8.41E-07 | UP |
| St8sia4 | 6.112980075 | 0.602108264 | 2.466637098 | 2.466637098 | 7.97E-08 | 8.53E-07 | UP |
| Rassf2 | 4.047553073 | 0.474481582 | 2.215133073 | 2.215133073 | 8.19E-08 | 8.75E-07 | UP |
| Nags | 10.77553289 | 33.84726936 | -2.384444497 | 2.384444497 | 8.30E-08 | 8.86E-07 | DOWN |
| P3h4 | 2.411239112 | 0.11110568 | 3.552677496 | 3.552677496 | 8.41E-08 | 8.97E-07 | UP |
| Tnfrsf9 | 2.405569244 | 0.061953213 | 4.424205043 | 4.424205043 | 8.49E-08 | 9.04E-07 | UP |
| Rhbdl2 | 1.928072869 | 0 | 7.530601361 | 7.530601361 | 9.07E-08 | 9.63E-07 | UP |
| Tspan1 | 3.279449141 | 0 | 9.224902134 | 9.224902134 | 9.11E-08 | 9.66E-07 | UP |
| Tnfaip6 | 2.824938483 | 0.075236869 | 4.275880728 | 4.275880728 | 9.24E-08 | 9.79E-07 | UP |
| Galnt3 | 2.832780521 | 0.054390942 | 4.695247386 | 4.695247386 | 9.26E-08 | 9.80E-07 | UP |
| Igf1 | 60.19175027 | 149.2488602 | -2.081245422 | 2.081245422 | 9.37E-08 | 9.91E-07 | DOWN |
| Pcp4l1 | 18.02960518 | 2.519802811 | 2.039206441 | 2.039206441 | 9.41E-08 | 9.94E-07 | UP |
| Grhl2 | 2.298921144 | 0.294567496 | 2.123669896 | 2.123669896 | 9.50E-08 | 1.00E-06 | UP |
| Hr | 1.258018828 | 0.046405449 | 3.87547377 | 3.87547377 | 9.72E-08 | 1.02E-06 | UP |
| LOC100910418 | 11.15000024 | 0.706692848 | 3.049763232 | 3.049763232 | 9.83E-08 | 1.03E-06 | UP |
| Cyp2c12 | 396.6411704 | 32.52045827 | 2.893005203 | 2.893005203 | 1.01E-07 | 1.07E-06 | UP |
| F10 | 65.75944079 | 207.3845623 | -2.394198472 | 2.394198472 | 1.02E-07 | 1.07E-06 | DOWN |
| Myo1f | 9.036364286 | 1.231232625 | 2.023458449 | 2.023458449 | 1.08E-07 | 1.13E-06 | UP |
| Gamt | 71.24109898 | 173.5830739 | -2.042932404 | 2.042932404 | 1.10E-07 | 1.15E-06 | DOWN |
| Car13 | 1.790415354 | 0.020497522 | 5.355848355 | 5.355848355 | 1.11E-07 | 1.16E-06 | UP |
| Acsm3 | 5.934627291 | 18.05761206 | -2.347541863 | 2.347541863 | 1.12E-07 | 1.17E-06 | DOWN |
| Ccr1 | 2.553462723 | 0.188206572 | 2.908499996 | 2.908499996 | 1.16E-07 | 1.20E-06 | UP |
| Cd84 | 8.812166243 | 0.862258151 | 2.471775143 | 2.471775143 | 1.16E-07 | 1.21E-06 | UP |
| Cyp2c23 | 371.4953508 | 928.6162459 | -2.109027333 | 2.109027333 | 1.18E-07 | 1.22E-06 | DOWN |
| RGD1564865 | 1.930123871 | 16.53208702 | -3.847887953 | 3.847887953 | 1.18E-07 | 1.23E-06 | DOWN |
| AABR07001905.1 | 1.589637528 | 0 | 7.393010506 | 7.393010506 | 1.20E-07 | 1.24E-06 | UP |
| Slc26a8 | 2.305833264 | 6.418247917 | -2.288345642 | 2.288345642 | 1.20E-07 | 1.24E-06 | DOWN |
| Ptgds | 4.461863235 | 0.129186739 | 4.228452199 | 4.228452199 | 1.21E-07 | 1.25E-06 | UP |
| Prrx1 | 2.475059886 | 0.066835317 | 4.275780171 | 4.275780171 | 1.22E-07 | 1.26E-06 | UP |
| Nrip3 | 2.922682036 | 0.254974986 | 2.617166059 | 2.617166059 | 1.31E-07 | 1.35E-06 | UP |
| Mybl2 | 1.926912065 | 0.077531158 | 3.706961915 | 3.706961915 | 1.32E-07 | 1.36E-06 | UP |
| LOC103693210 | 7.276778028 | 0.187940764 | 4.316499606 | 4.316499606 | 1.35E-07 | 1.38E-06 | UP |
| Pmaip1 | 3.241401817 | 0.178525823 | 3.26217914 | 3.26217914 | 1.35E-07 | 1.38E-06 | UP |
| Card14 | 2.066071354 | 0.116158327 | 3.255904204 | 3.255904204 | 1.37E-07 | 1.40E-06 | UP |
| Bmp4 | 2.15146588 | 0.156019593 | 2.941131148 | 2.941131148 | 1.40E-07 | 1.43E-06 | UP |
| Bend6 | 2.253846763 | 0.059360554 | 4.253898828 | 4.253898828 | 1.43E-07 | 1.46E-06 | UP |
| Neurl2 | 2.098311196 | 5.942349146 | -2.294880561 | 2.294880561 | 1.44E-07 | 1.47E-06 | DOWN |
| Xylt1 | 1.282784818 | 0.049415031 | 3.827873151 | 3.827873151 | 1.46E-07 | 1.49E-06 | UP |
| Paqr6 | 1.212533999 | 0.075969491 | 3.085876661 | 3.085876661 | 1.49E-07 | 1.52E-06 | UP |
| Hmgb2 | 28.25898757 | 3.718775632 | 2.065547637 | 2.065547637 | 1.57E-07 | 1.59E-06 | UP |
| Rad18 | 3.695924681 | 0.323252185 | 2.617920198 | 2.617920198 | 1.60E-07 | 1.61E-06 | UP |
| Fhdc1 | 1.721081203 | 0.084532156 | 3.446745209 | 3.446745209 | 1.62E-07 | 1.64E-06 | UP |
| Cplx2 | 3.367487972 | 0.397720114 | 2.221640327 | 2.221640327 | 1.63E-07 | 1.64E-06 | UP |
| Apln | 3.320575276 | 0.326907211 | 2.485683262 | 2.485683262 | 1.67E-07 | 1.68E-06 | UP |
| Hk2 | 5.480072296 | 0.433629404 | 2.750127271 | 2.750127271 | 1.68E-07 | 1.69E-06 | UP |
| Hhip | 0.545619711 | 1.963450961 | -2.725076192 | 2.725076192 | 1.74E-07 | 1.74E-06 | DOWN |
| Prr5l | 2.344576745 | 0.194282593 | 2.72564828 | 2.72564828 | 1.79E-07 | 1.79E-06 | UP |
| Etfb | 54.96555725 | 139.5886063 | -2.099404193 | 2.099404193 | 1.81E-07 | 1.81E-06 | DOWN |
| Mmp28 | 2.51702825 | 0.064677258 | 4.304745105 | 4.304745105 | 1.81E-07 | 1.81E-06 | UP |
| Syt13 | 1.311746185 | 0.017620296 | 5.191705529 | 5.191705529 | 1.86E-07 | 1.86E-06 | UP |
| Tspan5 | 6.428177592 | 0.881015816 | 2.01355298 | 2.01355298 | 1.87E-07 | 1.86E-06 | UP |
| Triqk | 3.343653425 | 0.280800373 | 2.701942855 | 2.701942855 | 1.88E-07 | 1.87E-06 | UP |
| Phactr2 | 4.798799551 | 0.665021251 | 2.015965064 | 2.015965064 | 1.88E-07 | 1.88E-06 | UP |
| Gbp4 | 4.069176321 | 0.38872985 | 2.542483754 | 2.542483754 | 1.90E-07 | 1.90E-06 | UP |
| Mmp9 | 1.422844942 | 0.079040644 | 3.261550936 | 3.261550936 | 1.93E-07 | 1.92E-06 | UP |
| Tpm2 | 8.955402964 | 0.810849859 | 2.59063885 | 2.59063885 | 1.95E-07 | 1.93E-06 | UP |
| Cdc6 | 1.200597162 | 0.046678791 | 3.769829 | 3.769829 | 1.97E-07 | 1.96E-06 | UP |
| NEWGENE_621351 | 2.767886863 | 0.119259569 | 3.587503062 | 3.587503062 | 1.99E-07 | 1.97E-06 | UP |
| Ms4a7 | 4.152970603 | 0.172985435 | 3.626481691 | 3.626481691 | 1.99E-07 | 1.97E-06 | UP |
| RGD1309350 | 39.65240272 | 105.2600508 | -2.153208135 | 2.153208135 | 2.00E-07 | 1.98E-06 | DOWN |
| Gpd1 | 99.60117099 | 252.2395755 | -2.094963941 | 2.094963941 | 2.01E-07 | 1.98E-06 | DOWN |
| Necab1 | 1.201531319 | 0.078971106 | 3.021717431 | 3.021717431 | 2.04E-07 | 2.01E-06 | UP |
| Sult2a6 | 49.07149907 | 223.7943781 | -2.912556792 | 2.912556792 | 2.08E-07 | 2.05E-06 | DOWN |
| Slc27a5 | 39.00889825 | 247.035812 | -3.35793561 | 3.35793561 | 2.12E-07 | 2.08E-06 | DOWN |
| Bspry | 8.977681195 | 0.808473201 | 2.564985616 | 2.564985616 | 2.12E-07 | 2.08E-06 | UP |
| Ccdc34 | 3.131002402 | 0.266455711 | 2.676300912 | 2.676300912 | 2.20E-07 | 2.16E-06 | UP |
| Col8a2 | 2.064109479 | 0.141737665 | 3.01158969 | 3.01158969 | 2.22E-07 | 2.18E-06 | UP |
| Coro2a | 2.757709963 | 0.066036784 | 4.390360754 | 4.390360754 | 2.23E-07 | 2.18E-06 | UP |
| Stk39 | 1.879571701 | 0.143840654 | 2.80681345 | 2.80681345 | 2.25E-07 | 2.20E-06 | UP |
| Arhgap27 | 8.97564353 | 1.192083617 | 2.058299998 | 2.058299998 | 2.28E-07 | 2.23E-06 | UP |
| Ccdc88a | 3.105284755 | 0.383177068 | 2.122987286 | 2.122987286 | 2.29E-07 | 2.24E-06 | UP |
| Des | 9.501100921 | 1.175076842 | 2.152494236 | 2.152494236 | 2.33E-07 | 2.27E-06 | UP |
| Apoe | 1610.683672 | 4879.684649 | -2.341419406 | 2.341419406 | 2.34E-07 | 2.29E-06 | DOWN |
| Cep162 | 1.894868625 | 0.248849076 | 2.067828824 | 2.067828824 | 2.40E-07 | 2.34E-06 | UP |
| Kif18a | 1.429550843 | 0.075110386 | 3.319717965 | 3.319717965 | 2.47E-07 | 2.41E-06 | UP |
| Sgca | 1.232892447 | 0 | 7.718653563 | 7.718653563 | 2.49E-07 | 2.42E-06 | UP |
| Tmem173 | 5.190298986 | 0.520329022 | 2.435052193 | 2.435052193 | 2.58E-07 | 2.50E-06 | UP |
| Lxn | 6.054754116 | 0.362029616 | 3.136388424 | 3.136388424 | 2.59E-07 | 2.51E-06 | UP |
| Esr1 | 1.01062366 | 5.92952121 | -3.243815777 | 3.243815777 | 2.60E-07 | 2.52E-06 | DOWN |
| Mbl1 | 38.75216648 | 106.1097096 | -2.191625918 | 2.191625918 | 2.64E-07 | 2.56E-06 | DOWN |
| Matn4 | 2.040187906 | 0.071561227 | 3.884467905 | 3.884467905 | 2.66E-07 | 2.58E-06 | UP |
| Slc44a4 | 11.72683343 | 1.052924202 | 2.570886923 | 2.570886923 | 2.70E-07 | 2.61E-06 | UP |
| Nol4l | 3.934429459 | 0.213527539 | 3.289369498 | 3.289369498 | 2.76E-07 | 2.66E-06 | UP |
| Zfp579 | 1.488494077 | 0.057388451 | 3.804962486 | 3.804962486 | 2.78E-07 | 2.68E-06 | UP |
| Sdcbp2 | 3.870953033 | 0 | 8.964071523 | 8.964071523 | 2.86E-07 | 2.75E-06 | UP |
| Syt11 | 1.700122127 | 0.196998992 | 2.259072152 | 2.259072152 | 2.86E-07 | 2.75E-06 | UP |
| Ercc6l | 1.236259463 | 0.041492274 | 3.953458044 | 3.953458044 | 2.92E-07 | 2.80E-06 | UP |
| Scn7a | 1.113786373 | 0.071870058 | 3.11602789 | 3.11602789 | 2.92E-07 | 2.80E-06 | UP |
| P2rx5 | 1.617731229 | 0.073163595 | 3.616400446 | 3.616400446 | 2.94E-07 | 2.82E-06 | UP |
| Ntf3 | 0.548934002 | 2.493696828 | -2.976331675 | 2.976331675 | 3.05E-07 | 2.91E-06 | DOWN |
| Nkain1 | 5.967501808 | 0.328553928 | 3.271440849 | 3.271440849 | 3.06E-07 | 2.93E-06 | UP |
| Atad5 | 1.18108456 | 0.12808063 | 2.340372639 | 2.340372639 | 3.06E-07 | 2.93E-06 | UP |
| Zfp521 | 1.544270039 | 0.140475111 | 2.643775219 | 2.643775219 | 3.07E-07 | 2.93E-06 | UP |
| Ahsp | 10.87475294 | 0.626182939 | 3.240929898 | 3.240929898 | 3.15E-07 | 3.00E-06 | UP |
| AC114111.1 | 0.950125889 | 6.476933055 | -3.550509634 | 3.550509634 | 3.16E-07 | 3.01E-06 | DOWN |
| Slc15a5 | 1.237753267 | 0 | 7.8314297 | 7.8314297 | 3.20E-07 | 3.05E-06 | UP |
| Lgals1 | 27.7160766 | 1.8308952 | 3.017128764 | 3.017128764 | 3.20E-07 | 3.05E-06 | UP |
| Cenph | 3.133720447 | 0.146357409 | 3.518747212 | 3.518747212 | 3.22E-07 | 3.06E-06 | UP |
| Mmp24 | 3.714148474 | 0.490554814 | 2.039227114 | 2.039227114 | 3.24E-07 | 3.07E-06 | UP |
| Fam169a | 1.791766648 | 0.031150837 | 4.836664159 | 4.836664159 | 3.25E-07 | 3.09E-06 | UP |
| Mest | 15.97991639 | 2.073794806 | 2.113648981 | 2.113648981 | 3.27E-07 | 3.10E-06 | UP |
| Ptpre | 5.30271817 | 0.544664818 | 2.41894641 | 2.41894641 | 3.29E-07 | 3.12E-06 | UP |
| AABR07049821.2 | 1.003698244 | 2.263039607 | -2.014900831 | 2.014900831 | 3.40E-07 | 3.21E-06 | DOWN |
| Ankrd33b | 1.458876958 | 4.448807243 | -2.399356472 | 2.399356472 | 3.54E-07 | 3.33E-06 | DOWN |
| C1qtnf3 | 0.034140753 | 1.411319041 | -6.06038438 | 6.06038438 | 3.57E-07 | 3.36E-06 | DOWN |
| Zbed5 | 12.92494285 | 32.77245664 | -2.126152358 | 2.126152358 | 3.59E-07 | 3.38E-06 | DOWN |
| Spdl1 | 2.2425397 | 0.178666042 | 2.752278474 | 2.752278474 | 3.60E-07 | 3.38E-06 | UP |
| Igf2bp3 | 3.396970582 | 9.928559341 | -2.363617744 | 2.363617744 | 3.61E-07 | 3.39E-06 | DOWN |
| Rbms3 | 3.999730573 | 0.383987402 | 2.519247538 | 2.519247538 | 3.67E-07 | 3.44E-06 | UP |
| Shc2 | 1.29174745 | 0.067952227 | 3.378273411 | 3.378273411 | 3.68E-07 | 3.45E-06 | UP |
| Mthfd2 | 6.506410307 | 0.747542167 | 2.257573192 | 2.257573192 | 3.79E-07 | 3.55E-06 | UP |
| Sez6 | 2.128769398 | 12.33015247 | -3.214135456 | 3.214135456 | 3.82E-07 | 3.58E-06 | DOWN |
| B3gnt7 | 3.250050192 | 0.348227612 | 2.342017645 | 2.342017645 | 3.91E-07 | 3.65E-06 | UP |
| Cxcr2 | 1.655492817 | 0.052845371 | 4.015917113 | 4.015917113 | 3.92E-07 | 3.65E-06 | UP |
| AABR07030359.1 | 3.540851004 | 0 | 8.121636345 | 8.121636345 | 3.94E-07 | 3.67E-06 | UP |
| Tpd52 | 31.37065146 | 3.638114358 | 2.208024382 | 2.208024382 | 4.03E-07 | 3.75E-06 | UP |
| Igf1r | 1.530538569 | 0.124745876 | 2.715950962 | 2.715950962 | 4.05E-07 | 3.76E-06 | UP |
| Zwilch | 5.825793574 | 0.559805247 | 2.477313614 | 2.477313614 | 4.06E-07 | 3.77E-06 | UP |
| Olfm1 | 2.486674309 | 0.065933125 | 4.298727681 | 4.298727681 | 4.06E-07 | 3.77E-06 | UP |
| Igdcc4 | 1.157118051 | 0.061923601 | 3.368626474 | 3.368626474 | 4.07E-07 | 3.78E-06 | UP |
| Bmp8b | 3.156993604 | 0 | 8.455188338 | 8.455188338 | 4.18E-07 | 3.87E-06 | UP |
| Zc2hc1a | 9.607577621 | 1.180082771 | 2.126770443 | 2.126770443 | 4.30E-07 | 3.97E-06 | UP |
| Ppp1r3g | 2.117969617 | 18.44069874 | -4.012948375 | 4.012948375 | 4.31E-07 | 3.98E-06 | DOWN |
| Pdxp | 1.20333881 | 3.112320429 | -2.181107269 | 2.181107269 | 4.33E-07 | 3.99E-06 | DOWN |
| MGC109340 | 1.012521683 | 0 | 7.318620091 | 7.318620091 | 4.38E-07 | 4.04E-06 | UP |
| Hsd17b6 | 11.64546779 | 68.46184078 | -3.246482881 | 3.246482881 | 4.52E-07 | 4.15E-06 | DOWN |
| C4bpb | 158.1422802 | 451.7578207 | -2.276969272 | 2.276969272 | 4.59E-07 | 4.22E-06 | DOWN |
| Oasl2 | 8.081261397 | 1.12344701 | 2.002403821 | 2.002403821 | 4.63E-07 | 4.25E-06 | UP |
| Apold1 | 3.069691264 | 7.899619254 | -2.205480045 | 2.205480045 | 4.67E-07 | 4.28E-06 | DOWN |
| Tcp11l1 | 4.122083885 | 0.518551151 | 2.138701992 | 2.138701992 | 4.67E-07 | 4.28E-06 | UP |
| Sftpd | 2.231510192 | 0.019628391 | 5.832931911 | 5.832931911 | 4.97E-07 | 4.53E-06 | UP |
| Plppr1 | 0.526184279 | 2.897127056 | -3.321165761 | 3.321165761 | 5.04E-07 | 4.59E-06 | DOWN |
| Dctd | 2.592110014 | 0.210271491 | 2.778443919 | 2.778443919 | 5.06E-07 | 4.60E-06 | UP |
| Rgs12 | 1.389326744 | 0.178512309 | 2.117865253 | 2.117865253 | 5.08E-07 | 4.61E-06 | UP |
| Rgs9bp | 0.202071938 | 2.035952499 | -4.027043692 | 4.027043692 | 5.38E-07 | 4.87E-06 | DOWN |
| Mpp7 | 2.244032099 | 0.204959092 | 2.577394737 | 2.577394737 | 5.40E-07 | 4.88E-06 | UP |
| Itm2a | 6.639707415 | 0.442476095 | 2.984005199 | 2.984005199 | 5.42E-07 | 4.90E-06 | UP |
| Apoc4 | 63.37193616 | 198.1419623 | -2.365111017 | 2.365111017 | 5.45E-07 | 4.92E-06 | DOWN |
| Fam83f | 1.396714636 | 0.040290075 | 4.182704667 | 4.182704667 | 5.49E-07 | 4.95E-06 | UP |
| Ccnd2 | 34.51665302 | 3.157705166 | 2.516178399 | 2.516178399 | 5.55E-07 | 4.99E-06 | UP |
| Ephb3 | 1.797518502 | 0.168188607 | 2.537063648 | 2.537063648 | 5.57E-07 | 5.02E-06 | UP |
| Hcn2 | 1.981753112 | 0.15271936 | 2.809534036 | 2.809534036 | 5.58E-07 | 5.02E-06 | UP |
| Slc4a11 | 1.618892377 | 0.033649275 | 4.600817352 | 4.600817352 | 5.82E-07 | 5.22E-06 | UP |
| Lat | 6.482215587 | 0.688585134 | 2.392610342 | 2.392610342 | 5.86E-07 | 5.24E-06 | UP |
| Trpv2 | 3.599619673 | 0.446452886 | 2.122465926 | 2.122465926 | 5.95E-07 | 5.31E-06 | UP |
| Chst1 | 1.79497811 | 0.129629635 | 2.920784847 | 2.920784847 | 5.97E-07 | 5.32E-06 | UP |
| Hs3st1 | 2.923381421 | 0.25529703 | 2.703367664 | 2.703367664 | 5.97E-07 | 5.33E-06 | UP |
| Ksr2 | 0.726893563 | 2.710471898 | -2.646726319 | 2.646726319 | 6.01E-07 | 5.35E-06 | DOWN |
| Reg3b | 21.28699123 | 0 | 10.55107192 | 10.55107192 | 6.10E-07 | 5.43E-06 | UP |
| Slc13a4 | 3.834752133 | 9.485073242 | -2.117392079 | 2.117392079 | 6.18E-07 | 5.49E-06 | DOWN |
| AABR07051450.1 | 1.594146522 | 0.078403976 | 3.459451058 | 3.459451058 | 6.20E-07 | 5.50E-06 | UP |
| Ska1 | 1.79520164 | 0.034064302 | 4.694585423 | 4.694585423 | 6.27E-07 | 5.56E-06 | UP |
| Fam222a | 0.82984188 | 3.429935468 | -2.811843842 | 2.811843842 | 6.52E-07 | 5.77E-06 | DOWN |
| Gjb3 | 5.172988357 | 0.049970589 | 5.569876211 | 5.569876211 | 6.65E-07 | 5.87E-06 | UP |
| Pmf1 | 5.815810359 | 0.705091706 | 2.19479675 | 2.19479675 | 6.69E-07 | 5.91E-06 | UP |
| Pou2f2 | 1.14201187 | 0.09807701 | 2.686651681 | 2.686651681 | 6.83E-07 | 6.02E-06 | UP |
| Got1 | 105.5319997 | 283.82546 | -2.196548992 | 2.196548992 | 7.07E-07 | 6.22E-06 | DOWN |
| Wt1 | 1.464764117 | 0.070737954 | 3.501901815 | 3.501901815 | 7.19E-07 | 6.32E-06 | UP |
| Kif15 | 1.659504086 | 0.128600247 | 2.774887423 | 2.774887423 | 7.22E-07 | 6.34E-06 | UP |
| Cdca8 | 6.7645319 | 0.848883011 | 2.121846316 | 2.121846316 | 7.24E-07 | 6.35E-06 | UP |
| Ovol2 | 1.822913793 | 0 | 7.733289108 | 7.733289108 | 7.38E-07 | 6.47E-06 | UP |
| Tmem255a | 1.428023914 | 0 | 7.9819084 | 7.9819084 | 7.57E-07 | 6.63E-06 | UP |
| Hspb2 | 3.093502262 | 0.037231462 | 5.141708131 | 5.141708131 | 8.11E-07 | 7.06E-06 | UP |
| Sytl1 | 2.616722454 | 0.118014823 | 3.589119182 | 3.589119182 | 8.18E-07 | 7.13E-06 | UP |
| Col13a1 | 0.394794312 | 1.308088115 | -2.507040755 | 2.507040755 | 8.51E-07 | 7.40E-06 | DOWN |
| Pdpn | 4.193962961 | 0.131799353 | 4.065561482 | 4.065561482 | 8.67E-07 | 7.53E-06 | UP |
| Gfpt2 | 2.085622892 | 0.205322634 | 2.473445705 | 2.473445705 | 8.70E-07 | 7.55E-06 | UP |
| Itk | 1.927595546 | 0.129845947 | 2.978042793 | 2.978042793 | 9.02E-07 | 7.80E-06 | UP |
| Cep126 | 1.317025658 | 0.098505011 | 2.863670447 | 2.863670447 | 9.17E-07 | 7.92E-06 | UP |
| Gdpd5 | 2.562602061 | 0.280066609 | 2.364715469 | 2.364715469 | 9.25E-07 | 7.98E-06 | UP |
| Np4 | 2.183889436 | 0 | 7.721418252 | 7.721418252 | 9.44E-07 | 8.13E-06 | UP |
| Ropn1 | 1.516903348 | 0 | 7.410207033 | 7.410207033 | 9.48E-07 | 8.16E-06 | UP |
| AABR07040840.1 | 8.623747753 | 21.24453856 | -2.08544821 | 2.08544821 | 9.72E-07 | 8.34E-06 | DOWN |
| Clec4a | 3.57129204 | 0.216044458 | 3.104808878 | 3.104808878 | 9.92E-07 | 8.49E-06 | UP |
| Sowaha | 2.747084998 | 0.160795021 | 3.199165917 | 3.199165917 | 1.01E-06 | 8.67E-06 | UP |
| Trps1 | 1.651780409 | 0.156653267 | 2.514656936 | 2.514656936 | 1.02E-06 | 8.69E-06 | UP |
| Krt12 | 3.967835838 | 0.044675903 | 5.693604964 | 5.693604964 | 1.03E-06 | 8.78E-06 | UP |
| Ly49si3 | 7.225695803 | 0.507458118 | 2.918523541 | 2.918523541 | 1.03E-06 | 8.79E-06 | UP |
| Mt2A | 216.9202707 | 672.7497833 | -2.414651984 | 2.414651984 | 1.05E-06 | 8.95E-06 | DOWN |
| Elf4 | 6.361998327 | 0.851188774 | 2.055583158 | 2.055583158 | 1.06E-06 | 8.99E-06 | UP |
| LOC108348083 | 3.983403315 | 0.225119236 | 3.286083525 | 3.286083525 | 1.11E-06 | 9.39E-06 | UP |
| LOC100362350 | 22.8772409 | 124.6534335 | -3.128577931 | 3.128577931 | 1.11E-06 | 9.40E-06 | DOWN |
| Cenpn | 1.310074308 | 0.112969951 | 2.646834329 | 2.646834329 | 1.11E-06 | 9.41E-06 | UP |
| LOC685048 | 2.322154028 | 0.036500129 | 4.901833417 | 4.901833417 | 1.12E-06 | 9.44E-06 | UP |
| Tmem45b | 9.184183355 | 0.011758636 | 8.451488647 | 8.451488647 | 1.14E-06 | 9.62E-06 | UP |
| Pmvk | 4.26812867 | 10.72304645 | -2.118667073 | 2.118667073 | 1.16E-06 | 9.75E-06 | DOWN |
| Dkk2 | 7.814128724 | 0.405487989 | 3.429360532 | 3.429360532 | 1.18E-06 | 9.97E-06 | UP |
| Fsip1 | 1.456708386 | 0.048878388 | 3.958764209 | 3.958764209 | 1.19E-06 | 1.00E-05 | UP |
| Guca2b | 5.70894525 | 0 | 8.115880317 | 8.115880317 | 1.20E-06 | 1.01E-05 | UP |
| Ankrd1 | 4.904213466 | 0.440122287 | 2.564963363 | 2.564963363 | 1.21E-06 | 1.01E-05 | UP |
| Fgl1 | 95.02457484 | 261.889681 | -2.216804247 | 2.216804247 | 1.23E-06 | 1.03E-05 | DOWN |
| Dusp13 | 1.220130331 | 0.043355117 | 3.947335815 | 3.947335815 | 1.24E-06 | 1.04E-05 | UP |
| Gjc3 | 1.062543235 | 5.101115446 | -3.023737835 | 3.023737835 | 1.26E-06 | 1.05E-05 | DOWN |
| Clec12a | 15.50409219 | 1.240044507 | 2.721631624 | 2.721631624 | 1.27E-06 | 1.06E-05 | UP |
| Cytip | 8.269841597 | 0.994908397 | 2.210352024 | 2.210352024 | 1.32E-06 | 1.10E-05 | UP |
| Alox5 | 2.754731285 | 0.161935194 | 3.230674719 | 3.230674719 | 1.33E-06 | 1.11E-05 | UP |
| Cntrob | 1.034708002 | 0.080505409 | 2.806126197 | 2.806126197 | 1.35E-06 | 1.12E-05 | UP |
| Serpinb5 | 1.885182026 | 0.047536199 | 4.31115959 | 4.31115959 | 1.37E-06 | 1.14E-05 | UP |
| Bend5 | 1.975221198 | 0.07812887 | 3.734323467 | 3.734323467 | 1.41E-06 | 1.17E-05 | UP |
| Dhcr24 | 99.48613658 | 269.5393928 | -2.183212425 | 2.183212425 | 1.45E-06 | 1.20E-05 | DOWN |
| Hpd | 284.2326774 | 719.494064 | -2.093111968 | 2.093111968 | 1.49E-06 | 1.23E-05 | DOWN |
| Adap1 | 3.006234621 | 0.234797525 | 2.777642685 | 2.777642685 | 1.50E-06 | 1.24E-05 | UP |
| Cdh11 | 2.66244593 | 0.249673751 | 2.523463949 | 2.523463949 | 1.52E-06 | 1.25E-05 | UP |
| AABR07028615.1 | 5.048061003 | 0.692371553 | 2.02699674 | 2.02699674 | 1.54E-06 | 1.26E-05 | UP |
| S100a4 | 9.670455072 | 0.849172236 | 2.636101006 | 2.636101006 | 1.55E-06 | 1.27E-05 | UP |
| Pdgfb | 5.08620583 | 0.684313455 | 2.034887741 | 2.034887741 | 1.56E-06 | 1.28E-05 | UP |
| RGD1559600 | 7.024854473 | 21.25141486 | -2.33860864 | 2.33860864 | 1.56E-06 | 1.28E-05 | DOWN |
| Grb10 | 6.719743883 | 0.743763065 | 2.268092272 | 2.268092272 | 1.57E-06 | 1.29E-05 | UP |
| Mcpt8 | 1.388593237 | 0.064710094 | 3.536069354 | 3.536069354 | 1.59E-06 | 1.30E-05 | UP |
| Tbc1d19 | 4.819632167 | 0.622102959 | 2.077649708 | 2.077649708 | 1.62E-06 | 1.32E-05 | UP |
| Fam19a5 | 1.097293475 | 0.016019679 | 5.078577769 | 5.078577769 | 1.62E-06 | 1.32E-05 | UP |
| LOC679149 | 4.149587431 | 0.043275584 | 5.516938644 | 5.516938644 | 1.62E-06 | 1.32E-05 | UP |
| Rbpjl | 1.17909177 | 0.039174683 | 3.976578676 | 3.976578676 | 1.62E-06 | 1.32E-05 | UP |
| Slc23a1 | 5.452480071 | 14.29967579 | -2.124331914 | 2.124331914 | 1.63E-06 | 1.33E-05 | DOWN |
| Fignl1 | 1.623147959 | 0.070482218 | 3.612308223 | 3.612308223 | 1.65E-06 | 1.34E-05 | UP |
| Cabp1 | 0.957873534 | 3.307520789 | -2.531513902 | 2.531513902 | 1.66E-06 | 1.35E-05 | DOWN |
| Ccl12 | 2.040880581 | 0 | 7.071381107 | 7.071381107 | 1.66E-06 | 1.35E-05 | UP |
| Ly6i | 1.36955925 | 0 | 7.255210605 | 7.255210605 | 1.71E-06 | 1.39E-05 | UP |
| Fgf21 | 13.63932711 | 132.5718815 | -4.077121316 | 4.077121316 | 1.72E-06 | 1.39E-05 | DOWN |
| Cdkn3 | 5.054692055 | 0.372275507 | 2.891130524 | 2.891130524 | 1.81E-06 | 1.47E-05 | UP |
| Ube2t | 3.838420096 | 0.165546552 | 3.597694533 | 3.597694533 | 1.82E-06 | 1.47E-05 | UP |
| Asl | 140.4744027 | 342.8121254 | -2.038504234 | 2.038504234 | 1.83E-06 | 1.48E-05 | DOWN |
| Cd248 | 1.97826423 | 0.135676848 | 2.987174229 | 2.987174229 | 1.84E-06 | 1.48E-05 | UP |
| Clec4a2 | 2.722974535 | 0.127736649 | 3.472612038 | 3.472612038 | 1.85E-06 | 1.49E-05 | UP |
| Cdt1 | 3.00839771 | 0.238244676 | 2.79690639 | 2.79690639 | 1.89E-06 | 1.53E-05 | UP |
| Fam198a | 0.398533464 | 1.841328378 | -3.026325493 | 3.026325493 | 1.91E-06 | 1.54E-05 | DOWN |
| Nim1k | 0.424163817 | 3.074694646 | -3.571277766 | 3.571277766 | 1.99E-06 | 1.60E-05 | DOWN |
| LOC100912471 | 6.975039023 | 0.727311572 | 2.384805482 | 2.384805482 | 2.00E-06 | 1.60E-05 | UP |
| LOC500712 | 376.3538397 | 926.989829 | -2.06314318 | 2.06314318 | 2.03E-06 | 1.62E-05 | DOWN |
| Mmp11 | 1.497836151 | 0.080599075 | 3.292895041 | 3.292895041 | 2.14E-06 | 1.70E-05 | UP |
| Glb1l2 | 3.531198875 | 0.287255805 | 2.810563599 | 2.810563599 | 2.19E-06 | 1.74E-05 | UP |
| Crip1 | 45.36572013 | 3.716608532 | 2.728967221 | 2.728967221 | 2.28E-06 | 1.80E-05 | UP |
| Gjb5 | 1.672713051 | 0 | 7.031671718 | 7.031671718 | 2.30E-06 | 1.82E-05 | UP |
| LOC497796 | 3.67803951 | 0.296648916 | 2.742699996 | 2.742699996 | 2.34E-06 | 1.85E-05 | UP |
| Daam2 | 2.176969994 | 0.26821771 | 2.169361663 | 2.169361663 | 2.39E-06 | 1.88E-05 | UP |
| Tex36 | 7.573592859 | 1.053982324 | 2.014669703 | 2.014669703 | 2.40E-06 | 1.90E-05 | UP |
| Qpct | 3.038332591 | 0.229512056 | 2.87223364 | 2.87223364 | 2.44E-06 | 1.92E-05 | UP |
| Aldh3b1 | 2.820789704 | 0.314730213 | 2.289965185 | 2.289965185 | 2.44E-06 | 1.92E-05 | UP |
| Tmem119 | 4.241995223 | 0.463492636 | 2.294954143 | 2.294954143 | 2.46E-06 | 1.94E-05 | UP |
| Haao | 37.39264245 | 101.871873 | -2.172407654 | 2.172407654 | 2.46E-06 | 1.94E-05 | DOWN |
| Sec14l2 | 17.18752197 | 42.60821283 | -2.043867252 | 2.043867252 | 2.47E-06 | 1.94E-05 | DOWN |
| Fibin | 2.149245322 | 0.107831771 | 3.361267719 | 3.361267719 | 2.48E-06 | 1.95E-05 | UP |
| Uox | 197.7658286 | 528.7110934 | -2.171398709 | 2.171398709 | 2.51E-06 | 1.97E-05 | DOWN |
| Rgcc | 3.743151767 | 0.116196942 | 4.123000611 | 4.123000611 | 2.51E-06 | 1.97E-05 | UP |
| Syt8 | 4.055978634 | 0.015838453 | 6.753011739 | 6.753011739 | 2.60E-06 | 2.03E-05 | UP |
| Ppp1r3c | 21.02411763 | 50.98600293 | -2.034816161 | 2.034816161 | 2.64E-06 | 2.06E-05 | DOWN |
| Clec4g | 5.019900449 | 12.456737 | -2.083167021 | 2.083167021 | 2.71E-06 | 2.11E-05 | DOWN |
| Ankrd13d | 1.473632969 | 0.050485817 | 3.92154685 | 3.92154685 | 2.73E-06 | 2.12E-05 | UP |
| AABR07053500.2 | 19.97449112 | 0 | 9.155566442 | 9.155566442 | 2.73E-06 | 2.12E-05 | UP |
| Tagln | 66.07347053 | 7.240660053 | 2.337520297 | 2.337520297 | 2.75E-06 | 2.14E-05 | UP |
| Ecscr | 4.015651543 | 0.233571026 | 3.213634677 | 3.213634677 | 2.78E-06 | 2.16E-05 | UP |
| Ephb2 | 1.499760588 | 0.071361798 | 3.520616867 | 3.520616867 | 2.80E-06 | 2.18E-05 | UP |
| AABR07030861.1 | 5.049554874 | 0.634734527 | 2.116031221 | 2.116031221 | 2.81E-06 | 2.18E-05 | UP |
| Ppp1r9a | 1.080651141 | 0.064451733 | 3.244863009 | 3.244863009 | 2.98E-06 | 2.30E-05 | UP |
| RT1-M5 | 1.122244309 | 0.02967574 | 4.207197064 | 4.207197064 | 3.08E-06 | 2.38E-05 | UP |
| Tdo2 | 307.4979743 | 840.636247 | -2.208612449 | 2.208612449 | 3.18E-06 | 2.45E-05 | DOWN |
| Ncmap | 2.191851975 | 0 | 6.811379584 | 6.811379584 | 3.36E-06 | 2.57E-05 | UP |
| Syt3 | 0.471873979 | 1.137705032 | -2.129067879 | 2.129067879 | 3.42E-06 | 2.62E-05 | DOWN |
| AABR07054319.1 | 13.39845782 | 1.372853537 | 2.471852952 | 2.471852952 | 3.42E-06 | 2.62E-05 | UP |
| Rbp7 | 21.33399553 | 2.021018556 | 2.634486026 | 2.634486026 | 3.44E-06 | 2.63E-05 | UP |
| Has2 | 1.285984233 | 0.019462358 | 5.011151328 | 5.011151328 | 3.46E-06 | 2.64E-05 | UP |
| Tcaf1 | 3.098503926 | 0.348671705 | 2.318958847 | 2.318958847 | 3.54E-06 | 2.70E-05 | UP |
| Cpne7 | 2.626169148 | 0.286596914 | 2.366499073 | 2.366499073 | 3.62E-06 | 2.76E-05 | UP |
| Ppp1r36 | 3.209324677 | 0.165648196 | 3.372178245 | 3.372178245 | 3.71E-06 | 2.82E-05 | UP |
| Hepacam | 2.107085266 | 0.0491768 | 4.568492108 | 4.568492108 | 3.75E-06 | 2.84E-05 | UP |
| Il2ra | 1.749359442 | 0.098606348 | 3.225114786 | 3.225114786 | 3.80E-06 | 2.88E-05 | UP |
| Smpdl3b | 1.731100481 | 0.166333368 | 2.518622609 | 2.518622609 | 3.87E-06 | 2.93E-05 | UP |
| Tedc1 | 1.88583603 | 0.223584403 | 2.18900691 | 2.18900691 | 3.93E-06 | 2.97E-05 | UP |
| Apof | 40.87380682 | 146.4513957 | -2.59737308 | 2.59737308 | 3.97E-06 | 3.00E-05 | DOWN |
| Kmo | 26.37694545 | 66.96715023 | -2.103637512 | 2.103637512 | 3.99E-06 | 3.01E-05 | DOWN |
| Eda2r | 1.191068059 | 0.042038731 | 3.991672349 | 3.991672349 | 4.02E-06 | 3.03E-05 | UP |
| Npr2 | 1.905319527 | 0.22477565 | 2.249317332 | 2.249317332 | 4.03E-06 | 3.03E-05 | UP |
| Hrg | 146.2588132 | 502.3657363 | -2.482808337 | 2.482808337 | 4.07E-06 | 3.06E-05 | DOWN |
| Gpr153 | 4.254036648 | 0.482322288 | 2.272944 | 2.272944 | 4.07E-06 | 3.06E-05 | UP |
| Cysltr1 | 1.382007007 | 0.143528513 | 2.409596982 | 2.409596982 | 4.23E-06 | 3.17E-05 | UP |
| LOC100362176 | 1.859970027 | 0 | 7.051304136 | 7.051304136 | 4.23E-06 | 3.18E-05 | UP |
| AABR07027870.1 | 1.686294368 | 0.139529427 | 2.71743019 | 2.71743019 | 4.26E-06 | 3.19E-05 | UP |
| Ier5l | 5.495798321 | 0.731175817 | 2.073872878 | 2.073872878 | 4.66E-06 | 3.47E-05 | UP |
| Lin7a | 0.485378755 | 2.639501934 | -3.170966897 | 3.170966897 | 4.80E-06 | 3.57E-05 | DOWN |
| Ttc36 | 16.1353488 | 79.63498499 | -2.975679272 | 2.975679272 | 4.85E-06 | 3.60E-05 | DOWN |
| B4galt2 | 1.866673963 | 0.121470896 | 3.016176747 | 3.016176747 | 4.98E-06 | 3.70E-05 | UP |
| Pdgfc | 18.41737817 | 2.094194639 | 2.313116725 | 2.313116725 | 5.15E-06 | 3.81E-05 | UP |
| AABR07002065.1 | 3.105945857 | 9.715474439 | -2.38862397 | 2.38862397 | 5.17E-06 | 3.82E-05 | DOWN |
| Igfbp2 | 10.85787215 | 1.538190037 | 2.033217595 | 2.033217595 | 5.19E-06 | 3.83E-05 | UP |
| Kcne4 | 1.784558167 | 0 | 6.792655463 | 6.792655463 | 5.24E-06 | 3.87E-05 | UP |
| Gins1 | 3.881733781 | 0.379526043 | 2.511628438 | 2.511628438 | 5.32E-06 | 3.92E-05 | UP |
| RGD1559962 | 12.9471667 | 1.231902879 | 2.485408123 | 2.485408123 | 5.36E-06 | 3.94E-05 | UP |
| Rimkla | 2.274615178 | 0.090477059 | 3.743309008 | 3.743309008 | 5.39E-06 | 3.96E-05 | UP |
| Itgb7 | 5.384190257 | 0.350158521 | 3.03587504 | 3.03587504 | 5.39E-06 | 3.97E-05 | UP |
| Fosl1 | 3.03466496 | 0.024429398 | 5.80963077 | 5.80963077 | 5.42E-06 | 3.98E-05 | UP |
| Lypd8 | 57.57003441 | 0 | 12.45708478 | 12.45708478 | 5.46E-06 | 4.01E-05 | UP |
| LOC685203 | 5.148109083 | 0.063086643 | 5.154989511 | 5.154989511 | 5.49E-06 | 4.02E-05 | UP |
| Ptpn7 | 2.244743371 | 0.306422777 | 2.026152162 | 2.026152162 | 5.59E-06 | 4.09E-05 | UP |
| Katnal2 | 0.262097088 | 1.221312265 | -2.984825987 | 2.984825987 | 5.70E-06 | 4.16E-05 | DOWN |
| Rassf10 | 2.125294372 | 0.120426297 | 3.275943705 | 3.275943705 | 5.79E-06 | 4.21E-05 | UP |
| Calcb | 1.319384081 | 0 | 6.960424071 | 6.960424071 | 5.80E-06 | 4.22E-05 | UP |
| Kif21b | 1.93643212 | 0.218044985 | 2.285923491 | 2.285923491 | 5.91E-06 | 4.29E-05 | UP |
| Pwwp2b | 3.423526505 | 0.412477012 | 2.189096505 | 2.189096505 | 5.91E-06 | 4.29E-05 | UP |
| Nrtn | 2.603057543 | 5.8856561 | -2.021366273 | 2.021366273 | 5.93E-06 | 4.30E-05 | DOWN |
| Tmem178a | 5.21034984 | 0.627231208 | 2.181253803 | 2.181253803 | 5.97E-06 | 4.33E-05 | UP |
| Ttc12 | 4.036788356 | 0.286159788 | 2.893905659 | 2.893905659 | 6.03E-06 | 4.37E-05 | UP |
| Ccdc68 | 3.538786648 | 0.355909505 | 2.423145555 | 2.423145555 | 6.38E-06 | 4.60E-05 | UP |
| Lpcat4 | 4.317126237 | 0.365738766 | 2.632190444 | 2.632190444 | 6.58E-06 | 4.73E-05 | UP |
| Arhgef25 | 1.724865618 | 0.078294212 | 3.548570211 | 3.548570211 | 6.66E-06 | 4.79E-05 | UP |
| Fancd2 | 2.197064421 | 0.243149948 | 2.307629591 | 2.307629591 | 6.89E-06 | 4.95E-05 | UP |
| Efcab7 | 3.495078777 | 0.465830939 | 2.050965108 | 2.050965108 | 7.08E-06 | 5.07E-05 | UP |
| Csdc2 | 1.003235331 | 0.016570792 | 4.903160002 | 4.903160002 | 7.15E-06 | 5.12E-05 | UP |
| Agpat4 | 3.641900175 | 0.42388405 | 2.25359506 | 2.25359506 | 7.20E-06 | 5.15E-05 | UP |
| Cd28 | 1.703623391 | 0.048291079 | 4.133634408 | 4.133634408 | 7.27E-06 | 5.20E-05 | UP |
| Shisa4 | 3.06475848 | 0.068821793 | 4.403771289 | 4.403771289 | 7.49E-06 | 5.34E-05 | UP |
| Edar | 0.581904524 | 1.454535122 | -2.125564015 | 2.125564015 | 7.65E-06 | 5.45E-05 | DOWN |
| Dscc1 | 1.582819778 | 0.090402743 | 3.245019161 | 3.245019161 | 7.66E-06 | 5.46E-05 | UP |
| Slco2a1 | 24.98383549 | 73.39657404 | -2.263937747 | 2.263937747 | 7.83E-06 | 5.58E-05 | DOWN |
| LOC108348266 | 1.79734212 | 4.136702845 | -2.060794817 | 2.060794817 | 7.84E-06 | 5.58E-05 | DOWN |
| Cubn | 1.050441127 | 0.029454274 | 4.210334373 | 4.210334373 | 7.92E-06 | 5.63E-05 | UP |
| Smarcd3 | 2.902906449 | 0.329838431 | 2.272610465 | 2.272610465 | 8.49E-06 | 6.01E-05 | UP |
| Bard1 | 1.105800499 | 0.08265795 | 2.841810871 | 2.841810871 | 8.77E-06 | 6.19E-05 | UP |
| Nat8f2 | 2.35951669 | 6.135744636 | -2.164284106 | 2.164284106 | 8.81E-06 | 6.22E-05 | DOWN |
| Cmahp | 1.477818154 | 0.127575212 | 2.682246268 | 2.682246268 | 8.91E-06 | 6.28E-05 | UP |
| Zfp185 | 1.084598837 | 0.014013041 | 5.029788515 | 5.029788515 | 8.99E-06 | 6.33E-05 | UP |
| Tmtc1 | 2.144004681 | 0.245852264 | 2.25458846 | 2.25458846 | 9.00E-06 | 6.33E-05 | UP |
| Pou2af1 | 2.537900143 | 0.186109369 | 2.858469254 | 2.858469254 | 9.14E-06 | 6.42E-05 | UP |
| Mtmr11 | 2.994420471 | 0.317347961 | 2.339119229 | 2.339119229 | 9.19E-06 | 6.45E-05 | UP |
| AABR07062183.1 | 10.92608486 | 1.210763747 | 2.257070797 | 2.257070797 | 9.55E-06 | 6.69E-05 | UP |
| Hemgn | 2.040309515 | 0.062967998 | 4.098892291 | 4.098892291 | 9.63E-06 | 6.73E-05 | UP |
| Spire2 | 1.672387229 | 0.054403778 | 3.950960341 | 3.950960341 | 9.77E-06 | 6.81E-05 | UP |
| Serpind1 | 106.0377295 | 260.650931 | -2.039937747 | 2.039937747 | 9.92E-06 | 6.90E-05 | DOWN |
| Cbarp | 2.558801635 | 0.247205806 | 2.459299844 | 2.459299844 | 1.02E-05 | 7.11E-05 | UP |
| LOC688286 | 0.95281659 | 2.56292233 | -2.212230286 | 2.212230286 | 1.02E-05 | 7.11E-05 | DOWN |
| Foxs1 | 2.493969309 | 0.161310689 | 3.081574275 | 3.081574275 | 1.03E-05 | 7.16E-05 | UP |
| Rab19 | 1.186775732 | 0.067893985 | 3.193129988 | 3.193129988 | 1.05E-05 | 7.26E-05 | UP |
| Tnk1 | 1.754184679 | 0.080161081 | 3.530698264 | 3.530698264 | 1.05E-05 | 7.29E-05 | UP |
| Mtbp | 1.096698304 | 0.105802901 | 2.526291759 | 2.526291759 | 1.09E-05 | 7.54E-05 | UP |
| Gkn2 | 8.6439905 | 0 | 9.139293915 | 9.139293915 | 1.09E-05 | 7.55E-05 | UP |
| Reep1 | 2.695359304 | 0.322267499 | 2.245366016 | 2.245366016 | 1.11E-05 | 7.68E-05 | UP |
| Tnfrsf4 | 2.35628167 | 0.08073528 | 3.87126354 | 3.87126354 | 1.12E-05 | 7.75E-05 | UP |
| Skp2 | 2.441160466 | 0.323712449 | 2.034753085 | 2.034753085 | 1.15E-05 | 7.93E-05 | UP |
| Sema3f | 3.37890027 | 0.366478104 | 2.404061586 | 2.404061586 | 1.19E-05 | 8.17E-05 | UP |
| Kctd1 | 1.704981771 | 0.232518304 | 2.024529491 | 2.024529491 | 1.23E-05 | 8.43E-05 | UP |
| Gls2 | 34.71974606 | 111.0107067 | -2.403931532 | 2.403931532 | 1.24E-05 | 8.47E-05 | DOWN |
| F13a1 | 2.023809548 | 0.111896244 | 3.285639904 | 3.285639904 | 1.24E-05 | 8.48E-05 | UP |
| Adm | 9.500765972 | 0.943781693 | 2.418284354 | 2.418284354 | 1.25E-05 | 8.54E-05 | UP |
| LOC100910526 | 0.73380055 | 3.388427546 | -2.907329229 | 2.907329229 | 1.25E-05 | 8.54E-05 | DOWN |
| Calml4 | 17.70893356 | 1.14503443 | 3.093234589 | 3.093234589 | 1.26E-05 | 8.58E-05 | UP |
| Tmem136 | 1.277332995 | 0.111212039 | 2.655877117 | 2.655877117 | 1.29E-05 | 8.75E-05 | UP |
| Ddias | 1.605621593 | 0.129766777 | 2.763561762 | 2.763561762 | 1.29E-05 | 8.77E-05 | UP |
| Cip2a | 1.563082347 | 0.126951722 | 2.727905517 | 2.727905517 | 1.29E-05 | 8.77E-05 | UP |
| Tubb4a | 1.754992409 | 0.056785634 | 4.05314226 | 4.05314226 | 1.31E-05 | 8.85E-05 | UP |
| Ptpru | 1.957130841 | 0.278962781 | 2.026892421 | 2.026892421 | 1.32E-05 | 8.95E-05 | UP |
| Sass6 | 1.522906038 | 0.122957044 | 2.733257382 | 2.733257382 | 1.33E-05 | 8.98E-05 | UP |
| Ly49s7 | 1.522649757 | 0.115650543 | 2.855219023 | 2.855219023 | 1.33E-05 | 8.99E-05 | UP |
| Pld2 | 3.873024962 | 0.512509283 | 2.054847437 | 2.054847437 | 1.35E-05 | 9.11E-05 | UP |
| LOC103690354 | 2.40313236 | 0 | 6.739714301 | 6.739714301 | 1.36E-05 | 9.14E-05 | UP |
| Cdc42ep5 | 14.87045873 | 1.245237532 | 2.646077665 | 2.646077665 | 1.36E-05 | 9.17E-05 | UP |
| Sh3bgr | 1.324891933 | 0.035554074 | 4.2368544 | 4.2368544 | 1.39E-05 | 9.35E-05 | UP |
| Tubb3 | 2.855714646 | 0.139617219 | 3.445459365 | 3.445459365 | 1.39E-05 | 9.36E-05 | UP |
| Msln | 46.10473912 | 0.874875519 | 4.859305467 | 4.859305467 | 1.40E-05 | 9.38E-05 | UP |
| Enah | 3.264176697 | 0.380794864 | 2.210014882 | 2.210014882 | 1.44E-05 | 9.61E-05 | UP |
| Ccl7 | 3.746914362 | 0.071683511 | 4.654393247 | 4.654393247 | 1.47E-05 | 9.80E-05 | UP |
| Cilp | 1.724804808 | 0.113854643 | 3.004355147 | 3.004355147 | 1.48E-05 | 9.85E-05 | UP |
| Tmem116 | 1.949027002 | 0.154171559 | 2.768203872 | 2.768203872 | 1.50E-05 | 9.98E-05 | UP |
| RGD1565785 | 2.522565083 | 0.237232817 | 2.543227457 | 2.543227457 | 1.57E-05 | 0.000104266 | UP |
| Nbl1 | 3.994424596 | 0.500859018 | 2.145215299 | 2.145215299 | 1.59E-05 | 0.00010575 | UP |
| Kcnn4 | 5.932270513 | 0.481592025 | 2.714436058 | 2.714436058 | 1.60E-05 | 0.000105866 | UP |
| Gpr27 | 1.538447312 | 0.036150398 | 4.379403197 | 4.379403197 | 1.62E-05 | 0.000107366 | UP |
| Star | 3.780008446 | 0.24769495 | 3.122348482 | 3.122348482 | 1.65E-05 | 0.000109176 | UP |
| Tgm1 | 4.499179714 | 0.285573082 | 3.029703543 | 3.029703543 | 1.71E-05 | 0.000112611 | UP |
| Paqr5 | 1.201505281 | 0.066256116 | 3.298350463 | 3.298350463 | 1.73E-05 | 0.000113953 | UP |
| Stc2 | 1.429323862 | 0.065462772 | 3.534088361 | 3.534088361 | 1.74E-05 | 0.000114234 | UP |
| Prkcq | 1.101742227 | 0.0486688 | 3.528223118 | 3.528223118 | 1.76E-05 | 0.000115769 | UP |
| Rnase1 | 2.170103296 | 0 | 6.615098349 | 6.615098349 | 1.82E-05 | 0.000119431 | UP |
| Mcpt1l1 | 3.17864203 | 0.039366365 | 5.33766364 | 5.33766364 | 1.87E-05 | 0.000122145 | UP |
| Aoah | 3.222536334 | 0.416258049 | 2.079805132 | 2.079805132 | 1.89E-05 | 0.000123435 | UP |
| Ntn4 | 2.134795041 | 0.196184513 | 2.554683227 | 2.554683227 | 1.91E-05 | 0.000124789 | UP |
| Syne4 | 2.198562846 | 0.080505731 | 3.946574284 | 3.946574284 | 1.95E-05 | 0.000127327 | UP |
| Neto2 | 5.090191072 | 0.453932577 | 2.55198257 | 2.55198257 | 2.03E-05 | 0.00013162 | UP |
| Pde9a | 1.049176173 | 0.036032818 | 3.85243718 | 3.85243718 | 2.06E-05 | 0.000132973 | UP |
| Col16a1 | 1.954413244 | 0.264886409 | 2.027182427 | 2.027182427 | 2.07E-05 | 0.000133906 | UP |
| Il18r1 | 1.261933053 | 0.077094379 | 3.118058434 | 3.118058434 | 2.08E-05 | 0.000134499 | UP |
| AABR07005618.1 | 3.587543999 | 9.19032247 | -2.182527619 | 2.182527619 | 2.11E-05 | 0.000135969 | DOWN |
| Chp2 | 2.170346999 | 0.152585518 | 2.929987276 | 2.929987276 | 2.13E-05 | 0.000137408 | UP |
| Pf4 | 18.26054454 | 2.447913917 | 2.033155089 | 2.033155089 | 2.14E-05 | 0.000137719 | UP |
| Sema3c | 13.62623445 | 0.718240347 | 3.318402391 | 3.318402391 | 2.15E-05 | 0.000138152 | UP |
| LOC24906 | 2.262449827 | 0.246764827 | 2.32603778 | 2.32603778 | 2.17E-05 | 0.00013943 | UP |
| Asf1b | 2.183570487 | 0.180020202 | 2.734234768 | 2.734234768 | 2.17E-05 | 0.000139507 | UP |
| 6-Sep | 2.539862539 | 0.316590454 | 2.105196744 | 2.105196744 | 2.19E-05 | 0.00014062 | UP |
| LOC103694381 | 3.66702724 | 0.391990601 | 2.371265771 | 2.371265771 | 2.22E-05 | 0.000142456 | UP |
| Ly49si1 | 7.575850152 | 0.968173754 | 2.032875519 | 2.032875519 | 2.25E-05 | 0.000143773 | UP |
| Anxa8 | 8.20535296 | 0.412164952 | 3.419956634 | 3.419956634 | 2.38E-05 | 0.000151469 | UP |
| Dact3 | 1.864980771 | 0.167335611 | 2.573348008 | 2.573348008 | 2.43E-05 | 0.000154779 | UP |
| Fbxl16 | 0.482573413 | 1.651442668 | -2.593854455 | 2.593854455 | 2.45E-05 | 0.000155862 | DOWN |
| Marco | 7.950706692 | 30.66252729 | -2.692927749 | 2.692927749 | 2.50E-05 | 0.000158775 | DOWN |
| Slc38a1 | 2.604844113 | 0.183962967 | 2.949798355 | 2.949798355 | 2.50E-05 | 0.000158902 | UP |
| Hes2 | 1.170940383 | 0 | 6.24483528 | 6.24483528 | 2.52E-05 | 0.000159618 | UP |
| Clmp | 1.720538551 | 0.099783453 | 3.211219194 | 3.211219194 | 2.53E-05 | 0.000160013 | UP |
| Ly49si2 | 1.117127573 | 0 | 6.529440406 | 6.529440406 | 2.55E-05 | 0.000161196 | UP |
| AC134204.1 | 0.413489183 | 1.000749816 | -2.120030852 | 2.120030852 | 2.59E-05 | 0.00016381 | DOWN |
| LOC100911440 | 0.367918281 | 1.166728549 | -2.424015894 | 2.424015894 | 2.63E-05 | 0.000166019 | DOWN |
| Cd3e | 4.776570618 | 0.27128613 | 3.182827723 | 3.182827723 | 2.66E-05 | 0.000167617 | UP |
| Tbc1d30 | 1.27204075 | 0.062547616 | 3.386934361 | 3.386934361 | 2.70E-05 | 0.000169584 | UP |
| Rad54l | 1.066625815 | 0.080559947 | 2.816544412 | 2.816544412 | 2.82E-05 | 0.000176519 | UP |
| Fam57a | 6.359345328 | 0.699702665 | 2.290617779 | 2.290617779 | 2.87E-05 | 0.000179284 | UP |
| Dpysl3 | 5.484663762 | 0.739538427 | 2.055879912 | 2.055879912 | 2.88E-05 | 0.000179995 | UP |
| Slc7a1 | 1.129108207 | 0.116980623 | 2.410186436 | 2.410186436 | 2.92E-05 | 0.000182579 | UP |
| AABR07058124.2 | 6.350953878 | 0.117616122 | 4.829865876 | 4.829865876 | 2.95E-05 | 0.000184001 | UP |
| Gja5 | 2.36854384 | 0.325579719 | 2.02671118 | 2.02671118 | 2.96E-05 | 0.000184017 | UP |
| PCOLCE2 | 3.86555116 | 0.146225876 | 3.725648418 | 3.725648418 | 2.99E-05 | 0.000185674 | UP |
| Ska3 | 2.866072442 | 0.38418037 | 2.015834916 | 2.015834916 | 3.02E-05 | 0.000187665 | UP |
| Gmnn | 4.729704893 | 0.572317298 | 2.19337607 | 2.19337607 | 3.05E-05 | 0.000189533 | UP |
| AABR07017902.1 | 3.064683013 | 0.382173846 | 2.08152829 | 2.08152829 | 3.06E-05 | 0.000189894 | UP |
| Fam69c | 0.272448828 | 1.19742376 | -2.966562777 | 2.966562777 | 3.07E-05 | 0.000190687 | DOWN |
| Scd | 42.0565976 | 132.2429663 | -2.540554651 | 2.540554651 | 3.14E-05 | 0.000194722 | DOWN |
| Rasl10b | 4.463299629 | 0.642756953 | 2.025049621 | 2.025049621 | 3.21E-05 | 0.000198347 | UP |
| St8sia1 | 0.7746146 | 2.812817542 | -2.642315805 | 2.642315805 | 3.32E-05 | 0.000204226 | DOWN |
| AABR07026112.1 | 2.798195917 | 0.37539598 | 2.033875146 | 2.033875146 | 3.40E-05 | 0.000208672 | UP |
| Prrt1 | 1.456761797 | 0.186871866 | 2.115742479 | 2.115742479 | 3.54E-05 | 0.000216902 | UP |
| Ccne1 | 3.404583568 | 0.356162702 | 2.378942366 | 2.378942366 | 3.55E-05 | 0.000217504 | UP |
| Apcdd1 | 1.665122439 | 0.123337108 | 2.858900705 | 2.858900705 | 3.56E-05 | 0.000217663 | UP |
| Gins4 | 4.380914907 | 0.54229819 | 2.142942869 | 2.142942869 | 3.61E-05 | 0.00022031 | UP |
| Trpv4 | 1.328830768 | 0.108585951 | 2.710475308 | 2.710475308 | 3.81E-05 | 0.000231461 | UP |
| Cacna2d4 | 0.333662748 | 1.285610908 | -2.667779191 | 2.667779191 | 3.81E-05 | 0.000231461 | DOWN |
| Plaur | 3.906302266 | 0.300597082 | 2.766591431 | 2.766591431 | 3.93E-05 | 0.00023722 | UP |
| Auts2 | 1.043828075 | 0.073158758 | 2.987720708 | 2.987720708 | 4.21E-05 | 0.000252036 | UP |
| Ffar2 | 1.005959521 | 0 | 6.605488598 | 6.605488598 | 4.34E-05 | 0.00025897 | UP |
| Dsel | 2.384371507 | 0.249367748 | 2.361650627 | 2.361650627 | 4.38E-05 | 0.000261082 | UP |
| Pclaf | 10.20226505 | 0.981949725 | 2.474916522 | 2.474916522 | 4.41E-05 | 0.000263002 | UP |
| Krt23 | 2.81991644 | 0.289979202 | 2.408787552 | 2.408787552 | 4.47E-05 | 0.000266102 | UP |
| Chaf1b | 1.957577654 | 0.130169891 | 3.051279225 | 3.051279225 | 4.55E-05 | 0.000269953 | UP |
| Ndrg4 | 1.072593242 | 0.073524389 | 2.98499223 | 2.98499223 | 4.69E-05 | 0.000277385 | UP |
| Cd69 | 2.43131116 | 0.163613171 | 2.962934647 | 2.962934647 | 4.75E-05 | 0.000280548 | UP |
| Acot11 | 1.286472858 | 0.057929804 | 3.493342571 | 3.493342571 | 4.75E-05 | 0.000280615 | UP |
| Tnfrsf14 | 4.508743239 | 0.367150154 | 2.716824134 | 2.716824134 | 4.80E-05 | 0.000283279 | UP |
| Prr7 | 6.940597403 | 0.526885773 | 2.761212163 | 2.761212163 | 4.89E-05 | 0.000287898 | UP |
| Cdc7 | 1.172926577 | 0.105236295 | 2.579753426 | 2.579753426 | 4.97E-05 | 0.000292475 | UP |
| Wdr31 | 3.07081704 | 0.406394838 | 2.062681326 | 2.062681326 | 4.98E-05 | 0.000292475 | UP |
| Atp6v0d2 | 0.21991799 | 1.071072324 | -3.012323021 | 3.012323021 | 5.12E-05 | 0.000299954 | DOWN |
| RGD1565844 | 1.083263559 | 0 | 6.164031769 | 6.164031769 | 5.15E-05 | 0.000301567 | UP |
| Clec4e | 1.279108297 | 0 | 6.851802678 | 6.851802678 | 5.48E-05 | 0.000318771 | UP |
| Slco3a1 | 2.15015819 | 0.268849447 | 2.131305377 | 2.131305377 | 5.49E-05 | 0.000319152 | UP |
| Pcdhga7 | 1.012336909 | 0.06748066 | 3.026666832 | 3.026666832 | 5.62E-05 | 0.000325922 | UP |
| Ucma | 0.309568382 | 1.359851274 | -2.871390793 | 2.871390793 | 5.66E-05 | 0.00032795 | DOWN |
| RGD1311744 | 1.430306971 | 0.159603492 | 2.274482963 | 2.274482963 | 5.67E-05 | 0.000328249 | UP |
| Lcn12 | 1.059713426 | 2.895898803 | -2.207614081 | 2.207614081 | 5.79E-05 | 0.000334313 | DOWN |
| Ogdhl | 1.628615649 | 0.136021681 | 2.732057934 | 2.732057934 | 6.04E-05 | 0.000347956 | UP |
| Rgs1 | 21.18493617 | 2.108086014 | 2.386641563 | 2.386641563 | 6.05E-05 | 0.000348776 | UP |
| Tbc1d16 | 1.816511465 | 0.195954456 | 2.312844287 | 2.312844287 | 6.18E-05 | 0.000355563 | UP |
| Kcnj16 | 1.64571857 | 0.162653495 | 2.495433378 | 2.495433378 | 6.25E-05 | 0.000359115 | UP |
| Muc13 | 8.746934611 | 0 | 11.84322435 | 11.84322435 | 6.26E-05 | 0.000359815 | UP |
| Bean1 | 2.030587138 | 0.165019073 | 2.706896175 | 2.706896175 | 6.30E-05 | 0.000362064 | UP |
| Slc16a3 | 2.224291482 | 0.161891887 | 2.88178565 | 2.88178565 | 6.36E-05 | 0.000364397 | UP |
| RGD1559588 | 4.607633451 | 0.213692699 | 3.464321963 | 3.464321963 | 6.38E-05 | 0.000365104 | UP |
| AABR07028997.1 | 3.278006839 | 0.209570414 | 3.069190119 | 3.069190119 | 6.42E-05 | 0.000367213 | UP |
| Tmc3 | 0.520518604 | 2.687781826 | -3.087450725 | 3.087450725 | 6.44E-05 | 0.000368255 | DOWN |
| Ptpn18 | 5.558014117 | 0.751199912 | 2.03391626 | 2.03391626 | 6.50E-05 | 0.000371069 | UP |
| Tm4sf5 | 4.287148522 | 0 | 8.529936296 | 8.529936296 | 6.52E-05 | 0.000371954 | UP |
| Fam83e | 1.565911836 | 0 | 7.708115922 | 7.708115922 | 6.80E-05 | 0.000386546 | UP |
| Mmp8 | 2.132608771 | 0.158881418 | 2.867066303 | 2.867066303 | 7.05E-05 | 0.00039961 | UP |
| AABR07054000.1 | 1.555153867 | 3.878564449 | -2.108395619 | 2.108395619 | 7.22E-05 | 0.000408778 | DOWN |
| Hmgb2l1 | 8.237698452 | 0.638833189 | 2.753877181 | 2.753877181 | 7.32E-05 | 0.000413611 | UP |
| Cd109 | 1.053065989 | 0.089676782 | 2.706055557 | 2.706055557 | 7.45E-05 | 0.000420511 | UP |
| Cdca7l | 1.12005273 | 0.081926335 | 2.833387796 | 2.833387796 | 7.79E-05 | 0.000437494 | UP |
| Ms4a12 | 1.8993369 | 0 | 7.134797154 | 7.134797154 | 8.01E-05 | 0.000449377 | UP |
| Pkib | 7.290599583 | 0.798570575 | 2.32744734 | 2.32744734 | 8.16E-05 | 0.000456758 | UP |
| Il21r | 2.873860298 | 0.332427961 | 2.195721345 | 2.195721345 | 8.27E-05 | 0.000462469 | UP |
| Emp3 | 19.67994244 | 2.715022918 | 2.000564721 | 2.000564721 | 8.35E-05 | 0.000466387 | UP |
| Camp | 1.129627221 | 0 | 6.386695641 | 6.386695641 | 8.36E-05 | 0.000466951 | UP |
| Itpripl1 | 2.224923384 | 0.305177287 | 2.026043974 | 2.026043974 | 8.44E-05 | 0.000471018 | UP |
| Duoxa2 | 5.157215468 | 0.084776946 | 4.839784118 | 4.839784118 | 8.50E-05 | 0.000473632 | UP |
| Agr2 | 6.040033318 | 0 | 10.0349544 | 10.0349544 | 8.63E-05 | 0.000480116 | UP |
| Cyp26a1 | 2.8760497 | 0.308586667 | 2.458099352 | 2.458099352 | 8.75E-05 | 0.000486503 | UP |
| Map3k6 | 2.166836277 | 0.235303585 | 2.380757728 | 2.380757728 | 9.15E-05 | 0.000507025 | UP |
| Chst11 | 1.051447309 | 0.119766726 | 2.261251707 | 2.261251707 | 9.41E-05 | 0.000520286 | UP |
| Crmp1 | 1.54760154 | 0.108270643 | 2.934535331 | 2.934535331 | 9.70E-05 | 0.000534867 | UP |
| Camkk2 | 1.366903223 | 0.143868074 | 2.384911292 | 2.384911292 | 9.83E-05 | 0.000540921 | UP |
| Clec3b | 4.932162949 | 0.398468416 | 2.778608156 | 2.778608156 | 9.94E-05 | 0.00054563 | UP |
| P2ry10 | 3.33197616 | 0.378533816 | 2.226365655 | 2.226365655 | 9.98E-05 | 0.00054789 | UP |
| Cdx2 | 4.682878104 | 0.040014104 | 5.813246764 | 5.813246764 | 1.00E-04 | 0.000548495 | UP |
| B4galt4 | 3.940952369 | 0.200911938 | 3.343550177 | 3.343550177 | 0.000100362 | 0.000550365 | UP |
| Cd207 | 1.002915148 | 0.028061609 | 4.189786903 | 4.189786903 | 0.000101461 | 0.000556077 | UP |
| Slc14a1 | 2.397028003 | 0.12849951 | 3.278977691 | 3.278977691 | 0.000101841 | 0.000557841 | UP |
| Hcar2 | 1.320281473 | 0.075365319 | 3.207576362 | 3.207576362 | 0.000102004 | 0.000558576 | UP |
| Nek2l1 | 1.653175611 | 0.123511634 | 2.862640744 | 2.862640744 | 0.000106201 | 0.000579582 | UP |
| Cd2 | 2.944869384 | 0.286970774 | 2.442316012 | 2.442316012 | 0.00010756 | 0.000586003 | UP |
| Zap70 | 2.981293288 | 0.367615502 | 2.139746281 | 2.139746281 | 0.000108053 | 0.000588188 | UP |
| Ints6l | 1.674815443 | 0.184093401 | 2.29043282 | 2.29043282 | 0.000110738 | 0.000601279 | UP |
| Gkn1 | 4.470754869 | 0 | 8.162515891 | 8.162515891 | 0.00011086 | 0.000601772 | UP |
| Slc51a | 2.636666191 | 0.07625105 | 4.121315376 | 4.121315376 | 0.000111056 | 0.000602495 | UP |
| LOC100910057 | 0.273180298 | 1.223634852 | -2.852868476 | 2.852868476 | 0.000113457 | 0.000614313 | DOWN |
| RT1-DOa | 1.241954363 | 0.110073914 | 2.587152083 | 2.587152083 | 0.000113965 | 0.000616719 | UP |
| Icos | 1.928137906 | 0.206341246 | 2.332153546 | 2.332153546 | 0.000117348 | 0.00063254 | UP |
| Fanca | 1.00305533 | 0.136491119 | 2.022378284 | 2.022378284 | 0.000118117 | 0.000636327 | UP |
| Tmem241 | 3.040915506 | 0.285615669 | 2.558150248 | 2.558150248 | 0.000120382 | 0.000647626 | UP |
| Spock2 | 1.490862254 | 0.095930119 | 3.037630805 | 3.037630805 | 0.00012123 | 0.000651639 | UP |
| Il1r2 | 2.189673399 | 0.056720389 | 4.240570613 | 4.240570613 | 0.000121916 | 0.000654965 | UP |
| Rab27b | 2.358684077 | 0.138307297 | 3.150137847 | 3.150137847 | 0.00012334 | 0.000661505 | UP |
| AABR07058998.1 | 2.367851122 | 0.094029297 | 3.639791764 | 3.639791764 | 0.000132989 | 0.000706575 | UP |
| Spef1 | 2.44916789 | 0.230540474 | 2.552682955 | 2.552682955 | 0.000136926 | 0.000724497 | UP |
| Hrct1 | 6.856199589 | 0.319931819 | 3.449569521 | 3.449569521 | 0.000138392 | 0.00073165 | UP |
| AABR07006278.1 | 1.097220718 | 0.023365818 | 4.34305279 | 4.34305279 | 0.00013851 | 0.000732074 | UP |
| Gng13 | 1.760631152 | 0 | 6.020736424 | 6.020736424 | 0.000140017 | 0.000738624 | UP |
| Gpihbp1 | 4.21389208 | 0.173867297 | 3.646420987 | 3.646420987 | 0.000140367 | 0.000740267 | UP |
| Cd40 | 2.341658457 | 0.283404548 | 2.214249037 | 2.214249037 | 0.000142368 | 0.000749386 | UP |
| Slc1a2 | 2.072594138 | 6.441541176 | -2.350352379 | 2.350352379 | 0.000142556 | 0.000750172 | DOWN |
| LOC688459 | 1.202624663 | 0 | 7.223976272 | 7.223976272 | 0.000143998 | 0.000756105 | UP |
| Gpr65 | 4.822505923 | 0.491200057 | 2.404089854 | 2.404089854 | 0.000147215 | 0.000771108 | UP |
| Itgad | 3.116663134 | 0.387134624 | 2.183368426 | 2.183368426 | 0.000147711 | 0.000772866 | UP |
| Mogat2 | 1.457974684 | 0.093505635 | 3.000464781 | 3.000464781 | 0.000151169 | 0.000787966 | UP |
| Cst6 | 1.724266798 | 0.024317241 | 4.875408913 | 4.875408913 | 0.000151449 | 0.000789215 | UP |
| Cbr1 | 1.058022627 | 0.118283564 | 2.288232293 | 2.288232293 | 0.000155982 | 0.000810426 | UP |
| Bhlhe41 | 2.258777946 | 0.257049787 | 2.262265302 | 2.262265302 | 0.000156576 | 0.000813075 | UP |
| Slc52a3 | 1.637996129 | 0.225152952 | 2.021589281 | 2.021589281 | 0.000157578 | 0.000818055 | UP |
| Mis18bp1 | 2.301734403 | 0.294311111 | 2.054592258 | 2.054592258 | 0.000159288 | 0.000826044 | UP |
| Galnt6 | 1.737469924 | 0.169825466 | 2.454527511 | 2.454527511 | 0.000167847 | 0.000866466 | UP |
| Map6 | 1.408880785 | 0.181952144 | 2.07774404 | 2.07774404 | 0.000175292 | 0.000901791 | UP |
| Tmem117 | 2.190088325 | 0.238153702 | 2.285887994 | 2.285887994 | 0.000177825 | 0.000913331 | UP |
| Tmem45al | 1.421569992 | 0.103372249 | 2.875547522 | 2.875547522 | 0.000177923 | 0.000913592 | UP |
| Slc10a2 | 1.623444357 | 0.1346487 | 2.658893798 | 2.658893798 | 0.000178758 | 0.000917144 | UP |
| LOC688335 | 0 | 1.226350861 | -6.083186495 | 6.083186495 | 0.000180151 | 0.000923063 | DOWN |
| Tnik | 1.292974326 | 0.168686936 | 2.108522057 | 2.108522057 | 0.000218177 | 0.001098058 | UP |
| Troap | 1.086158475 | 0.099309203 | 2.558977358 | 2.558977358 | 0.00022237 | 0.001117412 | UP |
| Camkmt | 2.564649845 | 0.161449948 | 3.043588445 | 3.043588445 | 0.000227662 | 0.001141329 | UP |
| Egr1 | 19.39378633 | 51.96777939 | -2.198327058 | 2.198327058 | 0.000236764 | 0.001181122 | DOWN |
| AC119762.3 | 1.318946114 | 0.106892456 | 2.758597024 | 2.758597024 | 0.000241792 | 0.001202942 | UP |
| Tpsab1 | 1.125942729 | 0 | 7.105539188 | 7.105539188 | 0.000244855 | 0.001215511 | UP |
| Slc16a11 | 1.058793192 | 2.679721569 | -2.126505911 | 2.126505911 | 0.000244924 | 0.00121554 | DOWN |
| AABR07054264.1 | 2.621792938 | 0 | 5.687328344 | 5.687328344 | 0.000245599 | 0.001218264 | UP |
| Zfp449 | 1.260508446 | 0.142074619 | 2.27897378 | 2.27897378 | 0.000247261 | 0.001224932 | UP |
| Tmem171 | 2.011666554 | 0.201125258 | 2.422941697 | 2.422941697 | 0.000247577 | 0.001226095 | UP |
| Sytl3 | 1.511660906 | 0.138976389 | 2.557733237 | 2.557733237 | 0.000248877 | 0.001231356 | UP |
| Mlc1 | 0.48767779 | 6.914571723 | -4.468555726 | 4.468555726 | 0.00024922 | 0.001232503 | DOWN |
| Igf2 | 5.741057737 | 0.20151654 | 3.862198024 | 3.862198024 | 0.000249976 | 0.001235528 | UP |
| Scin | 1.713633879 | 0.171971602 | 2.398679198 | 2.398679198 | 0.000261516 | 0.00128597 | UP |
| Cbln3 | 0.587634669 | 2.586062235 | -2.853693432 | 2.853693432 | 0.00027194 | 0.001330442 | DOWN |
| Sult2b1 | 3.024313027 | 0.213406235 | 2.925081482 | 2.925081482 | 0.000277917 | 0.001355898 | UP |
| Stap1 | 2.476101357 | 0.261153076 | 2.341220482 | 2.341220482 | 0.000281827 | 0.001372199 | UP |
| Ackr2 | 1.105828836 | 2.828420702 | -2.160218982 | 2.160218982 | 0.000287633 | 0.001396972 | DOWN |
| Cst7 | 4.373202486 | 0.593326499 | 2.012793438 | 2.012793438 | 0.000290139 | 0.001407337 | UP |
| Chaf1a | 2.14825211 | 0.297855608 | 2.027727221 | 2.027727221 | 0.000293422 | 0.00142076 | UP |
| LOC688553 | 1.855319396 | 0.170363782 | 2.544390909 | 2.544390909 | 0.000297139 | 0.001436955 | UP |
| Cyp26b1 | 1.634989482 | 0.158414739 | 2.619765292 | 2.619765292 | 0.00030516 | 0.001470949 | UP |
| Sds | 45.61845857 | 150.6060138 | -2.433394827 | 2.433394827 | 0.000305911 | 0.001474201 | DOWN |
| Rpp25 | 3.936351079 | 0.417647856 | 2.393775288 | 2.393775288 | 0.000309542 | 0.001490577 | UP |
| Car12 | 2.946317667 | 0.253524431 | 2.737256329 | 2.737256329 | 0.000312381 | 0.001503499 | UP |
| Cenpk | 1.443961876 | 0.113004409 | 2.825672534 | 2.825672534 | 0.000323117 | 0.001550141 | UP |
| Pla2g2a | 13.10190778 | 1.419830607 | 2.504933664 | 2.504933664 | 0.000324773 | 0.001556925 | UP |
| Mst1r | 2.130632127 | 0.218468877 | 2.3945851 | 2.3945851 | 0.000325348 | 0.001559295 | UP |
| Angpt2 | 3.058137382 | 0.369494605 | 2.1612689 | 2.1612689 | 0.000342934 | 0.001635449 | UP |
| Gimap7 | 4.501561668 | 0.533027398 | 2.159080995 | 2.159080995 | 0.000343564 | 0.001637969 | UP |
| Nrgn | 3.112762529 | 0.343127056 | 2.344113065 | 2.344113065 | 0.000344478 | 0.001641593 | UP |
| RGD1359634 | 3.670598418 | 0.472046398 | 2.115169345 | 2.115169345 | 0.000344999 | 0.001643669 | UP |
| Rtn2 | 1.073890921 | 0.053195148 | 3.345502648 | 3.345502648 | 0.000348321 | 0.001657447 | UP |
| Oip5 | 2.303074509 | 0.149723041 | 2.983860847 | 2.983860847 | 0.00036184 | 0.001713738 | UP |
| Pfn2 | 1.269748625 | 0.10753975 | 2.705404903 | 2.705404903 | 0.000363851 | 0.00172115 | UP |
| Gng8 | 1.933246965 | 0.228471392 | 2.207719094 | 2.207719094 | 0.000383411 | 0.00180218 | UP |
| Cdca5 | 1.122952587 | 0.08275961 | 2.849624121 | 2.849624121 | 0.000391852 | 0.001838598 | UP |
| Zmynd15 | 1.252534358 | 0.11365154 | 2.590428092 | 2.590428092 | 0.0003971 | 0.00186063 | UP |
| Cadm4 | 1.800724743 | 4.769105013 | -2.320817079 | 2.320817079 | 0.000402308 | 0.001881827 | DOWN |
| Pdlim3 | 1.677348314 | 0.06509404 | 3.744950768 | 3.744950768 | 0.000411347 | 0.001919917 | UP |
| Runx3 | 2.159699838 | 0.290266328 | 2.05348755 | 2.05348755 | 0.000423285 | 0.001971821 | UP |
| Prlr | 5.064071763 | 14.87752663 | -2.359989331 | 2.359989331 | 0.000423663 | 0.001972629 | DOWN |
| Mcemp1 | 1.085749971 | 0.073196004 | 2.921540992 | 2.921540992 | 0.000443577 | 0.002052463 | UP |
| Ypel1 | 1.464160017 | 0 | 5.814792764 | 5.814792764 | 0.00046307 | 0.002131402 | UP |
| S100g | 5.487847997 | 0 | 7.656892908 | 7.656892908 | 0.000468059 | 0.002148901 | UP |
| Rnase1l1 | 2.803105662 | 0 | 7.004516243 | 7.004516243 | 0.000478893 | 0.002193237 | UP |
| Cdyl2 | 1.42722699 | 0.190294595 | 2.023004004 | 2.023004004 | 0.000479453 | 0.002195283 | UP |
| Kctd14 | 1.520242332 | 0.151795707 | 2.45149064 | 2.45149064 | 0.000517611 | 0.00235157 | UP |
| Fam72a | 2.788813804 | 0.230594405 | 2.674680844 | 2.674680844 | 0.000522293 | 0.002371163 | UP |
| Sfta2 | 2.319873117 | 0 | 6.346354351 | 6.346354351 | 0.000536941 | 0.002429087 | UP |
| Mrgprx3 | 1.007052382 | 0.047731753 | 3.380877243 | 3.380877243 | 0.000547875 | 0.002472748 | UP |
| Stard6 | 1.131668503 | 0.078066936 | 2.886995706 | 2.886995706 | 0.000548459 | 0.002474223 | UP |
| Actg2 | 2.71562832 | 0.272828594 | 2.404411256 | 2.404411256 | 0.000549222 | 0.002477088 | UP |
| Spc24 | 2.635510444 | 0.306999578 | 2.226947031 | 2.226947031 | 0.000561777 | 0.002526621 | UP |
| Zfp40 | 1.327215748 | 0.151258656 | 2.229053578 | 2.229053578 | 0.000603213 | 0.002694129 | UP |
| Tmem8b | 1.565968102 | 0.158973684 | 2.384965392 | 2.384965392 | 0.000619204 | 0.002757881 | UP |
| Mmp7 | 1.457595453 | 0.018605805 | 5.030481185 | 5.030481185 | 0.00062676 | 0.002787031 | UP |
| Cd3g | 6.267447797 | 0.529966383 | 2.613862968 | 2.613862968 | 0.000640448 | 0.002841998 | UP |
| Ms4a4c | 2.301422336 | 0.09298606 | 3.637004933 | 3.637004933 | 0.000647311 | 0.00286849 | UP |
| Akr1cl | 65.2900571 | 6.935071918 | 2.346267468 | 2.346267468 | 0.000659721 | 0.002914777 | UP |
| Tmeff2 | 1.013779012 | 0.083272841 | 2.702141424 | 2.702141424 | 0.000678249 | 0.002984779 | UP |
| Rac3 | 0.796732115 | 2.022849495 | -2.148717548 | 2.148717548 | 0.000687026 | 0.003017436 | DOWN |
| Socs1 | 3.723397612 | 0.30593621 | 2.658801826 | 2.658801826 | 0.000688229 | 0.00302203 | UP |
| Ydjc | 1.046844921 | 0.068294141 | 3.045527564 | 3.045527564 | 0.0006931 | 0.003042034 | UP |
| AABR07062138.2 | 3.072682631 | 0.309903596 | 2.406922 | 2.406922 | 0.000710985 | 0.003112032 | UP |
| Cyp4a8 | 1.66276336 | 4.109623622 | -2.040025057 | 2.040025057 | 0.000723345 | 0.003156815 | DOWN |
| Kctd15 | 2.005904809 | 0.255394896 | 2.132806989 | 2.132806989 | 0.000728736 | 0.003177466 | UP |
| Rad51ap1 | 1.739764757 | 0.216226372 | 2.158916552 | 2.158916552 | 0.00074294 | 0.003230632 | UP |
| Akr1c12l1 | 0.823184058 | 1.79006704 | -2.026687477 | 2.026687477 | 0.000752009 | 0.003261975 | DOWN |
| Smim31 | 1.498918372 | 0 | 6.370966508 | 6.370966508 | 0.000765113 | 0.003312856 | UP |
| Susd3 | 2.055566739 | 0.19172313 | 2.582681327 | 2.582681327 | 0.000770815 | 0.003336796 | UP |
| Myo1a | 1.76337434 | 0.030474423 | 4.826743228 | 4.826743228 | 0.000780113 | 0.003368725 | UP |
| Clec4d | 1.328547773 | 0.097400244 | 2.820199261 | 2.820199261 | 0.000792682 | 0.003416117 | UP |
| Batf | 1.849006264 | 0.123350124 | 2.984730581 | 2.984730581 | 0.000798013 | 0.003435249 | UP |
| Mustn1 | 2.426958313 | 0.224948737 | 2.538405365 | 2.538405365 | 0.000818149 | 0.00350435 | UP |
| Clec2e | 2.47565005 | 0.031389612 | 5.146798599 | 5.146798599 | 0.00086328 | 0.003675993 | UP |
| Reg3g | 1.799356354 | 0 | 6.992942141 | 6.992942141 | 0.000872383 | 0.003711476 | UP |
| Cd8b | 1.648847863 | 0.125108056 | 2.767450859 | 2.767450859 | 0.000894192 | 0.003794214 | UP |
| Trem1 | 2.039274898 | 0.138429275 | 2.966629639 | 2.966629639 | 0.00091011 | 0.003856666 | UP |
| Sh2d2a | 1.648419262 | 0.10421464 | 3.015996433 | 3.015996433 | 0.000953564 | 0.004013463 | UP |
| Wdr54 | 2.471593618 | 0.230255014 | 2.488100864 | 2.488100864 | 0.000953405 | 0.004013463 | UP |
| Camk2n2 | 1.057551876 | 3.530751315 | -2.56530204 | 2.56530204 | 0.000963637 | 0.004051437 | DOWN |
| Atp1b2 | 1.26861951 | 0.114956308 | 2.603429842 | 2.603429842 | 0.000993045 | 0.004166898 | UP |

**Table S2 Differentially expressed genes between the PZH administration group and the DEN model group.**

| **GENE** | **MOD** | **PZH** | **log2FC** | **Absolute value** | **Pvalue** | **Qvalue** | **updown** |
| --- | --- | --- | --- | --- | --- | --- | --- |
| Mup4 | 30.6209063 | 0.056999889 | 9.642343135 | 9.642343135 | 1.39E-76 | 2.72E-72 | UP |
| Cyp2c11 | 351.9627157 | 0.818756234 | 9.386675293 | 9.386675293 | 8.08E-71 | 7.91E-67 | UP |
| AABR07047899.1 | 101.426944 | 0 | 13.23663337 | 13.23663337 | 5.33E-67 | 3.48E-63 | UP |
| AABR07048474.1 | 461.2591798 | 0.190117401 | 11.94679043 | 11.94679043 | 3.42E-57 | 1.67E-53 | UP |
| LOC298111 | 51.39815323 | 0.021061179 | 11.50614984 | 11.50614984 | 2.05E-53 | 8.00E-50 | UP |
| LOC259244 | 1215.327582 | 0.489797306 | 11.9616638 | 11.9616638 | 1.45E-41 | 4.72E-38 | UP |
| Mup4 | 288.0491533 | 0.048046763 | 12.99244672 | 12.99244672 | 3.02E-41 | 8.44E-38 | UP |
| Ust5r | 57.78209657 | 2.511068999 | 5.140767588 | 5.140767588 | 5.59E-41 | 1.37E-37 | UP |
| RGD1566134 | 70.88121471 | 0.025070532 | 11.73742676 | 11.73742676 | 1.58E-38 | 3.43E-35 | UP |
| AABR07048463.1 | 48.99155035 | 0 | 11.61090023 | 11.61090023 | 1.08E-37 | 2.11E-34 | UP |
| Dhrs7l1 | 107.5659254 | 2.171158766 | 6.264376988 | 6.264376988 | 8.22E-35 | 1.46E-31 | UP |
| LOC500473 | 86.31710994 | 0.050101769 | 11.27021561 | 11.27021561 | 1.04E-32 | 1.70E-29 | UP |
| Cyp4a2 | 370.5477891 | 15.08403697 | 5.309959801 | 5.309959801 | 1.73E-30 | 2.60E-27 | UP |
| Serpina3c | 1894.701572 | 200.5085651 | 3.929642082 | 3.929642082 | 7.30E-28 | 9.52E-25 | UP |
| RGD1559960 | 8.750017394 | 1.085141578 | 3.688849644 | 3.688849644 | 5.11E-27 | 6.25E-24 | UP |
| AABR07048487.2 | 41.64829231 | 0 | 10.94562541 | 10.94562541 | 7.31E-23 | 7.94E-20 | UP |
| Fndc1 | 0.73771509 | 12.49165835 | -3.390521555 | 3.390521555 | 1.38E-21 | 1.42E-18 | DOWN |
| LOC100365958 | 64.07289596 | 13.10926015 | 2.937456886 | 2.937456886 | 4.02E-21 | 3.93E-18 | UP |
| Cyp2c13 | 571.3816713 | 34.71862188 | 4.657781286 | 4.657781286 | 7.90E-21 | 7.36E-18 | UP |
| Zfp354a | 8.150955654 | 1.816100072 | 2.834979883 | 2.834979883 | 4.06E-20 | 3.46E-17 | UP |
| Slco1a4 | 54.01537421 | 5.443983131 | 3.924561573 | 3.924561573 | 3.89E-20 | 3.46E-17 | UP |
| Akr1b7 | 0.830497146 | 34.61943527 | -4.825246338 | 4.825246338 | 6.11E-20 | 4.98E-17 | DOWN |
| LOC100912405 | 395.9430108 | 8.675862196 | 6.036815782 | 6.036815782 | 1.10E-19 | 8.60E-17 | UP |
| Hao2 | 85.58400432 | 8.493707721 | 3.931207865 | 3.931207865 | 1.32E-19 | 9.94E-17 | UP |
| LOC100360095 | 1056.228161 | 1.885382883 | 9.608949031 | 9.608949031 | 2.29E-19 | 1.66E-16 | UP |
| Acnat2 | 43.22661701 | 2.201233575 | 4.937687283 | 4.937687283 | 4.14E-19 | 2.89E-16 | UP |
| Dio1 | 61.07241668 | 10.27849732 | 3.183664492 | 3.183664492 | 5.46E-19 | 3.68E-16 | UP |
| Kynu | 61.22632528 | 15.33194283 | 2.630404734 | 2.630404734 | 3.88E-18 | 2.53E-15 | UP |
| Mreg | 45.94093729 | 7.266154541 | 3.270094645 | 3.270094645 | 4.51E-18 | 2.85E-15 | UP |
| Dclk1 | 0.38327689 | 3.522643445 | -2.530208616 | 2.530208616 | 5.72E-18 | 3.49E-15 | DOWN |
| Lama5 | 1.307551449 | 13.17278586 | -2.633038063 | 2.633038063 | 6.29E-18 | 3.73E-15 | DOWN |
| Tsku | 85.4365652 | 20.54926207 | 2.700499345 | 2.700499345 | 7.35E-18 | 4.23E-15 | UP |
| Rup2 | 4658.845754 | 329.7284429 | 4.38924793 | 4.38924793 | 1.35E-17 | 7.54E-15 | UP |
| Nox4 | 4.81043821 | 0.532644317 | 3.906665795 | 3.906665795 | 4.62E-17 | 2.51E-14 | UP |
| Lcn2 | 11.15708819 | 115.9978935 | -2.712610978 | 2.712610978 | 1.02E-16 | 5.39E-14 | DOWN |
| Cyp2c7 | 745.6385492 | 57.38813167 | 4.280026185 | 4.280026185 | 1.50E-16 | 7.72E-14 | UP |
| Sult1c3 | 56.20472214 | 4.573509026 | 4.228799188 | 4.228799188 | 2.56E-16 | 1.28E-13 | UP |
| Gabrp | 1.039684663 | 19.43403066 | -3.547286765 | 3.547286765 | 4.21E-16 | 2.05E-13 | DOWN |
| LOC680406 | 1605.25698 | 109.5640575 | 4.418234451 | 4.418234451 | 4.30E-16 | 2.05E-13 | UP |
| LOC297568 | 995.886538 | 168.0974803 | 3.189885873 | 3.189885873 | 5.86E-16 | 2.73E-13 | UP |
| Inmt | 6.745593692 | 0.132983794 | 6.299789222 | 6.299789222 | 7.26E-16 | 3.30E-13 | UP |
| Apoa2 | 771.3982419 | 102.6841893 | 3.539652631 | 3.539652631 | 2.36E-15 | 1.05E-12 | UP |
| Pglyrp1 | 0.144533113 | 8.982506161 | -5.231281162 | 5.231281162 | 3.04E-15 | 1.32E-12 | DOWN |
| Degs2 | 0.523089302 | 14.91529766 | -4.16053437 | 4.16053437 | 5.71E-15 | 2.43E-12 | DOWN |
| Serpina4 | 466.2301097 | 73.52570457 | 3.263824222 | 3.263824222 | 9.39E-15 | 3.91E-12 | UP |
| Lrtm2 | 4.049728566 | 0.174124459 | 5.111909062 | 5.111909062 | 1.68E-14 | 6.70E-12 | UP |
| Slc25a25 | 58.86479815 | 15.94778043 | 2.512855142 | 2.512855142 | 1.78E-14 | 6.88E-12 | UP |
| Cyp3a18 | 201.3817508 | 19.44303205 | 3.936063696 | 3.936063696 | 2.59E-14 | 9.73E-12 | UP |
| LOC100910235 | 142.188753 | 11.29579604 | 4.250041863 | 4.250041863 | 3.42E-14 | 1.26E-11 | UP |
| Notum | 8.981631204 | 1.197031961 | 3.527231208 | 3.527231208 | 6.97E-14 | 2.53E-11 | UP |
| Mbl2 | 61.13365392 | 10.29327039 | 3.140881925 | 3.140881925 | 8.69E-14 | 3.09E-11 | UP |
| Klf5 | 0.40819416 | 17.62810835 | -4.694444938 | 4.694444938 | 1.18E-13 | 4.05E-11 | DOWN |
| Cyp3a23/3a1 | 335.9986668 | 4.09577722 | 6.856605489 | 6.856605489 | 1.40E-13 | 4.71E-11 | UP |
| Apon | 112.8754909 | 21.4773775 | 2.989046994 | 2.989046994 | 1.83E-13 | 6.08E-11 | UP |
| Aplnr | 0.732053932 | 14.05050599 | -3.650748818 | 3.650748818 | 2.37E-13 | 7.73E-11 | DOWN |
| Crot | 61.71330776 | 18.18758939 | 2.406943679 | 2.406943679 | 3.51E-13 | 1.13E-10 | UP |
| Olr59 | 3.48692203 | 0.417797928 | 3.671523991 | 3.671523991 | 4.17E-13 | 1.32E-10 | UP |
| Espn | 0.48100752 | 5.609435598 | -2.88084877 | 2.88084877 | 4.26E-13 | 1.32E-10 | DOWN |
| Krt20 | 0.771077783 | 17.88012655 | -3.793284993 | 3.793284993 | 4.53E-13 | 1.38E-10 | DOWN |
| Gckr | 39.47386351 | 13.61125504 | 2.17292775 | 2.17292775 | 4.61E-13 | 1.39E-10 | UP |
| Slco1b2 | 185.362164 | 35.42721032 | 2.966790352 | 2.966790352 | 4.92E-13 | 1.46E-10 | UP |
| AABR07069878.1 | 93.60203068 | 0.953810189 | 7.187899059 | 7.187899059 | 5.07E-13 | 1.48E-10 | UP |
| LOC100361547 | 80.43937231 | 7.355345229 | 4.000954706 | 4.000954706 | 5.65E-13 | 1.63E-10 | UP |
| Slc15a1 | 0.306859505 | 12.41830268 | -4.606816102 | 4.606816102 | 6.16E-13 | 1.72E-10 | DOWN |
| A3galt2 | 1.128180511 | 14.24858753 | -2.932115344 | 2.932115344 | 6.91E-13 | 1.90E-10 | DOWN |
| Agmo | 133.5976973 | 47.22017952 | 2.140257723 | 2.140257723 | 7.83E-13 | 2.13E-10 | UP |
| Zbtb16 | 9.902258988 | 2.38193492 | 2.764738044 | 2.764738044 | 8.05E-13 | 2.16E-10 | UP |
| Prag1 | 0.38851509 | 5.347971398 | -3.094594178 | 3.094594178 | 8.32E-13 | 2.20E-10 | DOWN |
| Mfsd2a | 66.73732718 | 18.19184631 | 2.492597184 | 2.492597184 | 8.61E-13 | 2.24E-10 | UP |
| Alox15 | 0.234566644 | 42.39335388 | -6.850378967 | 6.850378967 | 1.02E-12 | 2.62E-10 | DOWN |
| Ttr | 4446.548915 | 604.3258113 | 3.503435121 | 3.503435121 | 1.71E-12 | 4.35E-10 | UP |
| Defa5 | 0 | 7.253285778 | -8.590791851 | 8.590791851 | 2.04E-12 | 5.11E-10 | DOWN |
| Insig1 | 127.2514355 | 37.81190087 | 2.36789954 | 2.36789954 | 2.87E-12 | 7.02E-10 | UP |
| Cldn4 | 1.199002289 | 14.77205916 | -2.911006814 | 2.911006814 | 3.57E-12 | 8.42E-10 | DOWN |
| Krt19 | 10.12456687 | 273.1523389 | -4.014584937 | 4.014584937 | 3.55E-12 | 8.42E-10 | DOWN |
| AABR07005821.1 | 9.6358824 | 85.11951041 | -2.442787899 | 2.442787899 | 3.51E-12 | 8.42E-10 | DOWN |
| S100a11 | 21.39741487 | 250.5303675 | -2.795763416 | 2.795763416 | 3.75E-12 | 8.74E-10 | DOWN |
| Sult1c2a | 40.37813697 | 8.287946578 | 2.853936096 | 2.853936096 | 3.85E-12 | 8.86E-10 | UP |
| Avpr1a | 14.54343825 | 0.574043708 | 5.178858446 | 5.178858446 | 3.96E-12 | 9.01E-10 | UP |
| AABR07048475.1 | 7.083900491 | 0 | 8.6458975 | 8.6458975 | 6.75E-12 | 1.52E-09 | UP |
| Kdelr3 | 3.137491947 | 24.66374904 | -2.29402657 | 2.29402657 | 7.50E-12 | 1.67E-09 | DOWN |
| LOC108348266 | 8.945725768 | 1.79734212 | 2.998291948 | 2.998291948 | 8.40E-12 | 1.85E-09 | UP |
| Gck | 3.389457746 | 0.209724085 | 4.561578517 | 4.561578517 | 8.78E-12 | 1.91E-09 | UP |
| Eps8l3 | 0.038216393 | 3.92199241 | -5.833485381 | 5.833485381 | 9.54E-12 | 2.05E-09 | DOWN |
| Tmem54 | 0.004845543 | 3.005395977 | -7.640643462 | 7.640643462 | 1.00E-11 | 2.13E-09 | DOWN |
| St14 | 2.810278069 | 24.07862891 | -2.381014527 | 2.381014527 | 1.08E-11 | 2.27E-09 | DOWN |
| AABR07044711.1 | 5.860650023 | 56.26659699 | -2.532356668 | 2.532356668 | 1.10E-11 | 2.29E-09 | DOWN |
| Anxa2 | 29.33404864 | 245.5178595 | -2.350497223 | 2.350497223 | 1.30E-11 | 2.64E-09 | DOWN |
| LOC100912026 | 5.135918806 | 0.075747727 | 6.342798355 | 6.342798355 | 1.30E-11 | 2.64E-09 | UP |
| Nlrp12 | 4.610254357 | 0.723744977 | 3.357720593 | 3.357720593 | 1.37E-11 | 2.73E-09 | UP |
| RatNP-3b | 0 | 3.217028077 | -8.301160093 | 8.301160093 | 1.69E-11 | 3.33E-09 | DOWN |
| Ppp1r3b | 20.18932186 | 4.544656281 | 2.723652996 | 2.723652996 | 1.77E-11 | 3.47E-09 | UP |
| Vwa1 | 0.907709441 | 9.024220427 | -2.594770361 | 2.594770361 | 1.85E-11 | 3.59E-09 | DOWN |
| Car4 | 0.01885428 | 15.98496485 | -8.28217006 | 8.28217006 | 1.88E-11 | 3.61E-09 | DOWN |
| Sgpp2 | 0.610247272 | 7.473283764 | -2.940181957 | 2.940181957 | 2.37E-11 | 4.50E-09 | DOWN |
| F2rl1 | 0.147349552 | 5.211546916 | -4.396924143 | 4.396924143 | 2.82E-11 | 5.31E-09 | DOWN |
| Mug2 | 126.2240234 | 32.84701323 | 2.556558629 | 2.556558629 | 3.01E-11 | 5.61E-09 | UP |
| Tymp | 27.97679079 | 7.924978719 | 2.492983082 | 2.492983082 | 3.51E-11 | 6.48E-09 | UP |
| Cd44 | 3.202023639 | 26.0985411 | -2.33468243 | 2.33468243 | 4.12E-11 | 7.53E-09 | DOWN |
| Stac3 | 2.408597797 | 0 | 9.429093369 | 9.429093369 | 4.18E-11 | 7.57E-09 | UP |
| Itga3 | 1.044671655 | 10.65925942 | -2.606914481 | 2.606914481 | 5.06E-11 | 9.09E-09 | DOWN |
| Sult1e1 | 522.7041356 | 7.335427523 | 6.70604111 | 6.70604111 | 5.29E-11 | 9.41E-09 | UP |
| Pde4c | 0.039019161 | 7.874204069 | -6.368195208 | 6.368195208 | 5.47E-11 | 9.64E-09 | DOWN |
| Slc22a8 | 56.44429405 | 14.43085614 | 2.647277437 | 2.647277437 | 5.69E-11 | 9.93E-09 | UP |
| Fblim1 | 1.640820861 | 14.26803578 | -2.406987846 | 2.406987846 | 6.57E-11 | 1.14E-08 | DOWN |
| Vegfd | 2.021410967 | 16.58961885 | -2.353351972 | 2.353351972 | 7.62E-11 | 1.29E-08 | DOWN |
| Scd | 287.4150024 | 42.0565976 | 3.407968832 | 3.407968832 | 7.65E-11 | 1.29E-08 | UP |
| Errfi1 | 315.5128983 | 121.272167 | 2.046556819 | 2.046556819 | 7.70E-11 | 1.29E-08 | UP |
| Lpin1 | 25.25560578 | 7.748799121 | 2.362638828 | 2.362638828 | 8.54E-11 | 1.42E-08 | UP |
| Cyp4a8 | 10.00344082 | 1.66276336 | 3.165267529 | 3.165267529 | 9.00E-11 | 1.47E-08 | UP |
| AABR07061950.1 | 244.0737032 | 76.02061182 | 2.299085097 | 2.299085097 | 8.97E-11 | 1.47E-08 | UP |
| Cdh3 | 0.586116218 | 5.026977848 | -2.39873224 | 2.39873224 | 1.10E-10 | 1.76E-08 | DOWN |
| Hsd3b5 | 70.50897536 | 4.601849636 | 4.696686594 | 4.696686594 | 1.25E-10 | 1.95E-08 | UP |
| Pfkfb1 | 38.84924073 | 13.43034805 | 2.143122775 | 2.143122775 | 1.29E-10 | 1.99E-08 | UP |
| Pnpla5 | 1.551818404 | 0.094848782 | 4.65710471 | 4.65710471 | 1.28E-10 | 1.99E-08 | UP |
| Olfml2b | 1.528984685 | 13.6357972 | -2.428697519 | 2.428697519 | 1.36E-10 | 2.09E-08 | DOWN |
| Misp | 0.094782666 | 5.316163856 | -4.954750759 | 4.954750759 | 1.58E-10 | 2.40E-08 | DOWN |
| Ehf | 1.650804477 | 15.32832023 | -2.515207554 | 2.515207554 | 1.74E-10 | 2.62E-08 | DOWN |
| Col11a1 | 0.341107875 | 3.334342163 | -2.588156228 | 2.588156228 | 2.24E-10 | 3.35E-08 | DOWN |
| Lad1 | 0.700095919 | 10.41130995 | -3.124804515 | 3.124804515 | 2.55E-10 | 3.77E-08 | DOWN |
| Serinc2 | 2.231486031 | 19.84483032 | -2.485526054 | 2.485526054 | 2.62E-10 | 3.83E-08 | DOWN |
| Ampd3 | 1.319511263 | 15.80468498 | -2.886340721 | 2.886340721 | 2.63E-10 | 3.83E-08 | DOWN |
| Ehhadh | 209.473383 | 79.73906502 | 2.064422217 | 2.064422217 | 2.93E-10 | 4.22E-08 | UP |
| Dusp1 | 91.94489985 | 31.96382893 | 2.246685229 | 2.246685229 | 3.52E-10 | 5.02E-08 | UP |
| Cxcl3 | 0 | 6.925125099 | -10.09142778 | 10.09142778 | 3.84E-10 | 5.44E-08 | DOWN |
| Ms4a8 | 0 | 23.39911351 | -11.44653852 | 11.44653852 | 4.18E-10 | 5.88E-08 | DOWN |
| Paqr9 | 26.12077399 | 7.825496866 | 2.332264128 | 2.332264128 | 4.22E-10 | 5.89E-08 | UP |
| Egf | 1.344517739 | 0.242303536 | 3.093163948 | 3.093163948 | 4.41E-10 | 6.11E-08 | UP |
| Slc25a24 | 3.451174715 | 32.54120335 | -2.515712244 | 2.515712244 | 4.48E-10 | 6.18E-08 | DOWN |
| Smlr1 | 45.91127148 | 11.87666655 | 2.557527807 | 2.557527807 | 4.55E-10 | 6.22E-08 | UP |
| Gpld1 | 68.5942457 | 20.40787356 | 2.384556546 | 2.384556546 | 4.65E-10 | 6.31E-08 | UP |
| Slco1a1 | 113.9751129 | 31.42189041 | 2.445509794 | 2.445509794 | 4.75E-10 | 6.41E-08 | UP |
| Amy1a | 229.7338613 | 55.73686339 | 2.625073001 | 2.625073001 | 4.98E-10 | 6.67E-08 | UP |
| 1-Mar | 86.22609527 | 25.13007068 | 2.401694164 | 2.401694164 | 5.10E-10 | 6.77E-08 | UP |
| Sorcs2 | 0.446547648 | 3.00757002 | -2.119512079 | 2.119512079 | 5.41E-10 | 7.10E-08 | DOWN |
| Cdo1 | 584.0995363 | 135.3782395 | 2.689315208 | 2.689315208 | 5.62E-10 | 7.27E-08 | UP |
| Anxa13 | 3.094476655 | 28.25678231 | -2.466237891 | 2.466237891 | 5.80E-10 | 7.46E-08 | DOWN |
| Fads2 | 132.0050597 | 40.16981091 | 2.381024285 | 2.381024285 | 6.54E-10 | 8.31E-08 | UP |
| Gpx2 | 15.79712757 | 179.7040842 | -2.804888946 | 2.804888946 | 7.55E-10 | 9.46E-08 | DOWN |
| Plet1 | 0.78680401 | 21.55115315 | -4.045375009 | 4.045375009 | 7.61E-10 | 9.49E-08 | DOWN |
| AC114343.1 | 52.35368607 | 9.86835249 | 2.983111485 | 2.983111485 | 8.15E-10 | 1.00E-07 | UP |
| Loxl1 | 2.719502343 | 18.93956306 | -2.078018005 | 2.078018005 | 8.49E-10 | 1.04E-07 | DOWN |
| Lamc2 | 2.103258772 | 15.55293421 | -2.189967735 | 2.189967735 | 8.60E-10 | 1.04E-07 | DOWN |
| B4galnt1 | 28.20581902 | 10.07480574 | 2.109152515 | 2.109152515 | 8.70E-10 | 1.04E-07 | UP |
| Acnat1 | 26.44567686 | 1.583513181 | 4.755401227 | 4.755401227 | 8.70E-10 | 1.04E-07 | UP |
| Cdkn2a | 0.13859976 | 5.877526677 | -4.547493065 | 4.547493065 | 9.11E-10 | 1.09E-07 | DOWN |
| Gcnt3 | 1.02953866 | 20.92189642 | -3.562092306 | 3.562092306 | 9.41E-10 | 1.12E-07 | DOWN |
| Adhfe1 | 78.41908183 | 22.17912848 | 2.409672101 | 2.409672101 | 1.08E-09 | 1.27E-07 | UP |
| Pon1 | 757.1754835 | 216.8837126 | 2.39353968 | 2.39353968 | 1.11E-09 | 1.30E-07 | UP |
| Sox9 | 1.32327674 | 8.995739558 | -2.10703186 | 2.10703186 | 1.13E-09 | 1.31E-07 | DOWN |
| Cyp2e1 | 1005.145274 | 168.683555 | 3.142475707 | 3.142475707 | 1.14E-09 | 1.32E-07 | UP |
| Impdh1 | 1.758033849 | 13.67823445 | -2.221963428 | 2.221963428 | 1.21E-09 | 1.39E-07 | DOWN |
| RGD1564865 | 13.80622481 | 1.930123871 | 3.418130511 | 3.418130511 | 1.27E-09 | 1.46E-07 | UP |
| Elf3 | 1.070024575 | 15.30142281 | -3.06904337 | 3.06904337 | 1.32E-09 | 1.50E-07 | DOWN |
| Tspan8 | 7.133598323 | 124.5769976 | -3.384419585 | 3.384419585 | 1.37E-09 | 1.54E-07 | DOWN |
| Cyp2d3 | 147.7293433 | 42.94688931 | 2.410618709 | 2.410618709 | 1.36E-09 | 1.54E-07 | UP |
| Gulo | 213.4393676 | 79.96331546 | 2.035904942 | 2.035904942 | 1.39E-09 | 1.55E-07 | UP |
| Col8a1 | 1.222028912 | 8.593201545 | -2.10619534 | 2.10619534 | 1.44E-09 | 1.60E-07 | DOWN |
| Prodh1 | 10.81672769 | 2.157661202 | 2.925078319 | 2.925078319 | 1.52E-09 | 1.67E-07 | UP |
| Aox3 | 1.345827785 | 0.065103079 | 4.969181531 | 4.969181531 | 1.57E-09 | 1.72E-07 | UP |
| Kcnn2 | 2.09067363 | 0.345681137 | 3.220806538 | 3.220806538 | 1.59E-09 | 1.73E-07 | UP |
| Cdcp1 | 0.333623845 | 3.259002029 | -2.619582538 | 2.619582538 | 1.65E-09 | 1.78E-07 | DOWN |
| Gprc5b | 0.170432503 | 2.440006916 | -3.196903149 | 3.196903149 | 1.65E-09 | 1.78E-07 | DOWN |
| Tmc5 | 0.086923702 | 3.113237087 | -4.474763942 | 4.474763942 | 1.69E-09 | 1.80E-07 | DOWN |
| Tm7sf2 | 36.75675491 | 11.57900995 | 2.301361687 | 2.301361687 | 1.75E-09 | 1.86E-07 | UP |
| Inhbc | 27.75438934 | 7.525565066 | 2.472072186 | 2.472072186 | 1.76E-09 | 1.86E-07 | UP |
| RGD1565367 | 0.87161352 | 8.080270094 | -2.558077721 | 2.558077721 | 1.87E-09 | 1.97E-07 | DOWN |
| Sidt1 | 1.814384578 | 0.61505004 | 2.196973129 | 2.196973129 | 1.93E-09 | 2.02E-07 | UP |
| Klf15 | 34.71278099 | 12.48482874 | 2.084437234 | 2.084437234 | 2.05E-09 | 2.13E-07 | UP |
| Lamb3 | 0.649779698 | 6.973639998 | -2.695305546 | 2.695305546 | 2.07E-09 | 2.15E-07 | DOWN |
| Slc10a1 | 217.538329 | 49.09192338 | 2.733916088 | 2.733916088 | 2.10E-09 | 2.16E-07 | UP |
| Cpa1 | 0.219576475 | 17.62684974 | -5.726902702 | 5.726902702 | 2.15E-09 | 2.20E-07 | DOWN |
| Tmem86b | 46.92835743 | 11.12753481 | 2.658672456 | 2.658672456 | 2.43E-09 | 2.47E-07 | UP |
| Lipo1 | 6.742932414 | 1.222845079 | 3.027875197 | 3.027875197 | 2.52E-09 | 2.54E-07 | UP |
| Lgals4 | 0.248045022 | 98.41418803 | -7.793302584 | 7.793302584 | 2.62E-09 | 2.62E-07 | DOWN |
| Cxcl6 | 1.383951805 | 15.48548336 | -2.80224572 | 2.80224572 | 2.81E-09 | 2.76E-07 | DOWN |
| Acsl1 | 441.9322997 | 170.682412 | 2.002341886 | 2.002341886 | 2.80E-09 | 2.76E-07 | UP |
| Trhde | 1.332404353 | 0.414414387 | 2.347064525 | 2.347064525 | 3.19E-09 | 3.09E-07 | UP |
| Scara3 | 0.103994636 | 1.566939884 | -3.259905659 | 3.259905659 | 3.23E-09 | 3.11E-07 | DOWN |
| Akr1c1 | 18.13052738 | 3.368337642 | 3.159557214 | 3.159557214 | 3.38E-09 | 3.22E-07 | UP |
| Rgs4 | 1.816851631 | 13.8531084 | -2.296376478 | 2.296376478 | 3.45E-09 | 3.27E-07 | DOWN |
| Baat | 463.4917877 | 121.4261619 | 2.506590554 | 2.506590554 | 3.89E-09 | 3.64E-07 | UP |
| Fscn1 | 1.913407002 | 13.43592657 | -2.157952167 | 2.157952167 | 3.95E-09 | 3.68E-07 | DOWN |
| Ttpa | 427.8282013 | 138.9692918 | 2.208371791 | 2.208371791 | 4.09E-09 | 3.77E-07 | UP |
| Acta2 | 4.279668599 | 59.82393706 | -3.02209253 | 3.02209253 | 4.33E-09 | 3.96E-07 | DOWN |
| Pkm | 2.655284706 | 22.12485391 | -2.305797346 | 2.305797346 | 4.51E-09 | 4.10E-07 | DOWN |
| Spta1 | 0.128970533 | 1.786821535 | -3.161277653 | 3.161277653 | 5.19E-09 | 4.63E-07 | DOWN |
| Prom1 | 1.08765526 | 10.78585842 | -2.642359944 | 2.642359944 | 5.36E-09 | 4.74E-07 | DOWN |
| Fam19a4 | 0.069728711 | 1.864885096 | -3.936814168 | 3.936814168 | 6.21E-09 | 5.42E-07 | DOWN |
| Apom | 123.0949645 | 30.90248995 | 2.562949901 | 2.562949901 | 6.95E-09 | 5.99E-07 | UP |
| Tat | 457.8044134 | 167.8261281 | 2.050472309 | 2.050472309 | 7.55E-09 | 6.45E-07 | UP |
| Plat | 3.570787413 | 33.1788553 | -2.482110121 | 2.482110121 | 8.12E-09 | 6.88E-07 | DOWN |
| Itgb4 | 0.964918168 | 8.657841861 | -2.41051059 | 2.41051059 | 8.23E-09 | 6.91E-07 | DOWN |
| Cldn7 | 1.270978381 | 18.58305837 | -3.148452123 | 3.148452123 | 8.22E-09 | 6.91E-07 | DOWN |
| Fxyd3 | 3.094293373 | 39.47230315 | -2.902164395 | 2.902164395 | 8.29E-09 | 6.93E-07 | DOWN |
| AABR07060293.1 | 17.15473623 | 4.492659793 | 2.53585791 | 2.53585791 | 8.74E-09 | 7.21E-07 | UP |
| Ptger3 | 13.0880258 | 4.359473421 | 2.182368621 | 2.182368621 | 9.10E-09 | 7.48E-07 | UP |
| Cdhr2 | 0.424513238 | 6.64017683 | -3.226911502 | 3.226911502 | 9.37E-09 | 7.64E-07 | DOWN |
| Cyp7a1 | 28.4786777 | 10.61597261 | 2.015914801 | 2.015914801 | 9.47E-09 | 7.68E-07 | UP |
| Olfr12 | 2.007245493 | 0.063271203 | 5.356602709 | 5.356602709 | 9.91E-09 | 8.01E-07 | UP |
| Bcas1 | 0.252489196 | 2.610171573 | -2.65746207 | 2.65746207 | 1.01E-08 | 8.13E-07 | DOWN |
| Etv4 | 0.074496611 | 2.375820194 | -4.214495748 | 4.214495748 | 1.03E-08 | 8.22E-07 | DOWN |
| Tmc4 | 0.378110186 | 4.415681372 | -2.875673478 | 2.875673478 | 1.04E-08 | 8.27E-07 | DOWN |
| Vil1 | 0.497417297 | 7.411289615 | -3.15959309 | 3.15959309 | 1.08E-08 | 8.51E-07 | DOWN |
| Fkbp10 | 0.722922661 | 5.130325765 | -2.110193169 | 2.110193169 | 1.09E-08 | 8.54E-07 | DOWN |
| Btnl2 | 0.34145416 | 3.933327904 | -2.90129862 | 2.90129862 | 1.31E-08 | 1.01E-06 | DOWN |
| AC130970.1 | 0.875290037 | 11.94810958 | -3.060086595 | 3.060086595 | 1.35E-08 | 1.03E-06 | DOWN |
| Crym | 12.73104319 | 3.489182545 | 2.582665974 | 2.582665974 | 1.34E-08 | 1.03E-06 | UP |
| Cyp2a3 | 73.49582555 | 27.90255091 | 2.026766013 | 2.026766013 | 1.39E-08 | 1.06E-06 | UP |
| Nov | 0.991089452 | 9.217149204 | -2.529893921 | 2.529893921 | 1.41E-08 | 1.07E-06 | DOWN |
| Cth | 281.9293906 | 66.81902345 | 2.633588628 | 2.633588628 | 1.51E-08 | 1.14E-06 | UP |
| Thrsp | 99.5257433 | 18.45596425 | 3.027192598 | 3.027192598 | 1.61E-08 | 1.21E-06 | UP |
| Ctgf | 5.974453174 | 46.56353038 | -2.242435853 | 2.242435853 | 1.62E-08 | 1.21E-06 | DOWN |
| Esrp1 | 0.258665446 | 4.543664897 | -3.427267791 | 3.427267791 | 1.64E-08 | 1.22E-06 | DOWN |
| C8g | 92.83648959 | 23.81925722 | 2.593762 | 2.593762 | 1.67E-08 | 1.23E-06 | UP |
| Pof1b | 0.100160888 | 3.072733506 | -4.21631305 | 4.21631305 | 1.76E-08 | 1.30E-06 | DOWN |
| Kif23 | 0.623430021 | 5.124886423 | -2.342541132 | 2.342541132 | 1.82E-08 | 1.33E-06 | DOWN |
| Gadd45g | 37.29322125 | 11.08484931 | 2.461699494 | 2.461699494 | 1.83E-08 | 1.34E-06 | UP |
| Dcdc2 | 1.955753682 | 12.63630912 | -2.045572348 | 2.045572348 | 1.90E-08 | 1.38E-06 | DOWN |
| Cpe | 0.72589995 | 6.868857504 | -2.657175469 | 2.657175469 | 2.03E-08 | 1.47E-06 | DOWN |
| Pxmp2 | 119.5928009 | 41.50723256 | 2.118169219 | 2.118169219 | 2.12E-08 | 1.52E-06 | UP |
| Ace | 0.499622061 | 4.066131452 | -2.325584902 | 2.325584902 | 2.12E-08 | 1.52E-06 | DOWN |
| Vwa2 | 0.134872247 | 2.276642915 | -3.348588448 | 3.348588448 | 2.42E-08 | 1.70E-06 | DOWN |
| Tnfsf18 | 0.11044681 | 3.582245527 | -4.176427014 | 4.176427014 | 2.55E-08 | 1.78E-06 | DOWN |
| Car3 | 121.4340806 | 2.441587868 | 6.099722957 | 6.099722957 | 2.64E-08 | 1.83E-06 | UP |
| Prss22 | 0.453821157 | 10.00262734 | -3.703859324 | 3.703859324 | 2.90E-08 | 1.99E-06 | DOWN |
| S100a6 | 18.20080203 | 212.5938747 | -2.790520932 | 2.790520932 | 2.97E-08 | 2.03E-06 | DOWN |
| Plac8 | 11.69651727 | 77.85800776 | -2.027362847 | 2.027362847 | 3.02E-08 | 2.06E-06 | DOWN |
| Rpp21 | 7.985078007 | 3.137071783 | 2.032987177 | 2.032987177 | 3.67E-08 | 2.48E-06 | UP |
| Fermt1 | 0.1508325 | 2.516265344 | -3.403368866 | 3.403368866 | 3.87E-08 | 2.58E-06 | DOWN |
| LOC103691744 | 24.25835143 | 4.433107654 | 3.068999104 | 3.068999104 | 4.03E-08 | 2.68E-06 | UP |
| Sult1c2 | 1.14270374 | 0.16143732 | 3.373912235 | 3.373912235 | 4.43E-08 | 2.91E-06 | UP |
| Plod2 | 2.542809881 | 19.47913496 | -2.222132754 | 2.222132754 | 4.69E-08 | 3.05E-06 | DOWN |
| Np4 | 0 | 2.183889436 | -7.875601796 | 7.875601796 | 4.89E-08 | 3.16E-06 | DOWN |
| Ptprz1 | 0.188364827 | 2.831694382 | -3.272240201 | 3.272240201 | 5.14E-08 | 3.31E-06 | DOWN |
| MGC105649 | 1.524733576 | 43.90650594 | -4.063531673 | 4.063531673 | 5.18E-08 | 3.31E-06 | DOWN |
| Gjb3 | 0.030194678 | 5.172988357 | -5.998780214 | 5.998780214 | 6.17E-08 | 3.88E-06 | DOWN |
| Pald1 | 17.65108244 | 6.819558442 | 2.000707891 | 2.000707891 | 6.62E-08 | 4.12E-06 | UP |
| Apoa4 | 335.7826195 | 57.29811506 | 3.173393738 | 3.173393738 | 6.86E-08 | 4.26E-06 | UP |
| Tm4sf20 | 0.028010508 | 8.765886755 | -7.486453772 | 7.486453772 | 7.05E-08 | 4.34E-06 | DOWN |
| Acsm5 | 37.48273033 | 11.39354873 | 2.417994161 | 2.417994161 | 7.04E-08 | 4.34E-06 | UP |
| Pllp | 0.209420234 | 6.100489567 | -4.080396449 | 4.080396449 | 7.14E-08 | 4.37E-06 | DOWN |
| Duoxa2 | 0 | 5.157215468 | -8.990697753 | 8.990697753 | 7.53E-08 | 4.59E-06 | DOWN |
| Cntfr | 1.473471564 | 0.180156505 | 3.682650383 | 3.682650383 | 8.24E-08 | 4.97E-06 | UP |
| Ptprd | 12.22192607 | 4.052450227 | 2.162143945 | 2.162143945 | 9.27E-08 | 5.55E-06 | UP |
| Inhbb | 0.58399672 | 5.908770269 | -2.705971818 | 2.705971818 | 9.63E-08 | 5.73E-06 | DOWN |
| Gipc2 | 1.412199499 | 12.31491638 | -2.43854005 | 2.43854005 | 1.00E-07 | 5.93E-06 | DOWN |
| Wnt7b | 0.097684876 | 1.483057126 | -3.217801369 | 3.217801369 | 1.02E-07 | 5.98E-06 | DOWN |
| Sdcbp2 | 0 | 3.870953033 | -9.118737632 | 9.118737632 | 1.02E-07 | 5.98E-06 | DOWN |
| Smpd3 | 1.318689318 | 10.35506671 | -2.231751341 | 2.231751341 | 1.05E-07 | 6.07E-06 | DOWN |
| S100a9 | 2.152259635 | 23.98154106 | -2.719384235 | 2.719384235 | 1.05E-07 | 6.07E-06 | DOWN |
| Ano1 | 0.794673908 | 8.357706102 | -2.651129264 | 2.651129264 | 1.12E-07 | 6.35E-06 | DOWN |
| Mpv17l | 2.290977517 | 0.36354419 | 3.21691209 | 3.21691209 | 1.13E-07 | 6.42E-06 | UP |
| Slc4a1 | 0.32398934 | 3.181502815 | -2.572942401 | 2.572942401 | 1.17E-07 | 6.61E-06 | DOWN |
| Duox2 | 0.027144931 | 7.482751407 | -7.118925214 | 7.118925214 | 1.19E-07 | 6.67E-06 | DOWN |
| Olr1 | 1.525713173 | 17.85324796 | -2.819448556 | 2.819448556 | 1.19E-07 | 6.67E-06 | DOWN |
| Tmprss4 | 0.077943768 | 2.15410714 | -3.958177774 | 3.958177774 | 1.25E-07 | 6.99E-06 | DOWN |
| Tspan17 | 0.303081727 | 3.457056257 | -2.799625154 | 2.799625154 | 1.30E-07 | 7.20E-06 | DOWN |
| Ctse | 4.814678563 | 37.1760186 | -2.243486406 | 2.243486406 | 1.37E-07 | 7.58E-06 | DOWN |
| B4galnt4 | 0.213106807 | 2.634015226 | -2.922175672 | 2.922175672 | 1.39E-07 | 7.63E-06 | DOWN |
| AABR07006691.1 | 7.380408538 | 1.608583511 | 2.815459503 | 2.815459503 | 1.46E-07 | 7.97E-06 | UP |
| Slc6a13 | 31.03996853 | 7.840445328 | 2.568934679 | 2.568934679 | 1.57E-07 | 8.47E-06 | UP |
| Kcnn4 | 0.555363988 | 5.932270513 | -2.664886 | 2.664886 | 1.57E-07 | 8.47E-06 | DOWN |
| LOC108348083 | 0.508200847 | 3.983403315 | -2.245173285 | 2.245173285 | 1.59E-07 | 8.55E-06 | DOWN |
| Abca6 | 39.08246962 | 14.30798055 | 2.031364687 | 2.031364687 | 1.62E-07 | 8.70E-06 | UP |
| Tff3 | 2.061613459 | 51.59150137 | -3.896782707 | 3.896782707 | 1.72E-07 | 9.15E-06 | DOWN |
| Erbb2 | 0.126060099 | 1.626010131 | -2.976950734 | 2.976950734 | 1.73E-07 | 9.17E-06 | DOWN |
| Slc15a2 | 0.134714714 | 1.452825042 | -2.843758881 | 2.843758881 | 1.74E-07 | 9.19E-06 | DOWN |
| Crlf1 | 0.183510835 | 4.675648129 | -3.901018091 | 3.901018091 | 1.78E-07 | 9.32E-06 | DOWN |
| AABR07064312.1 | 4.439495039 | 0.137161465 | 5.545665725 | 5.545665725 | 1.78E-07 | 9.32E-06 | UP |
| Lyz2 | 59.99996848 | 413.1567917 | -2.045273576 | 2.045273576 | 1.96E-07 | 1.02E-05 | DOWN |
| Acsm3 | 22.31159189 | 5.934627291 | 2.469012215 | 2.469012215 | 1.96E-07 | 1.02E-05 | UP |
| RGD1563692 | 0.067205257 | 1.208250367 | -3.486858222 | 3.486858222 | 1.98E-07 | 1.02E-05 | DOWN |
| Rbp4 | 2655.645045 | 941.5955995 | 2.12466777 | 2.12466777 | 2.02E-07 | 1.04E-05 | UP |
| Flrt3 | 1.564806877 | 10.47954334 | -2.090383793 | 2.090383793 | 2.08E-07 | 1.07E-05 | DOWN |
| Afm | 456.9076974 | 131.2843701 | 2.363047844 | 2.363047844 | 2.14E-07 | 1.08E-05 | UP |
| Serpine1 | 2.783616672 | 21.56317025 | -2.207003606 | 2.207003606 | 2.16E-07 | 1.09E-05 | DOWN |
| Mybl2 | 0.149412939 | 1.926912065 | -2.949577123 | 2.949577123 | 2.21E-07 | 1.11E-05 | DOWN |
| Mapk13 | 0.229226523 | 4.507421232 | -3.507733648 | 3.507733648 | 2.23E-07 | 1.11E-05 | DOWN |
| Fjx1 | 0.445996508 | 4.092462012 | -2.512755087 | 2.512755087 | 2.31E-07 | 1.15E-05 | DOWN |
| Pfkp | 1.878728463 | 12.83643213 | -2.050653584 | 2.050653584 | 2.36E-07 | 1.17E-05 | DOWN |
| Cyp1b1 | 0.388646648 | 5.041995661 | -2.968372041 | 2.968372041 | 2.39E-07 | 1.18E-05 | DOWN |
| Lect2 | 51.46231634 | 19.12704467 | 2.096611096 | 2.096611096 | 2.50E-07 | 1.23E-05 | UP |
| Cthrc1 | 0.232056494 | 4.066853314 | -3.375878253 | 3.375878253 | 2.52E-07 | 1.24E-05 | DOWN |
| Cyp4f1 | 206.8610909 | 74.82542264 | 2.068682242 | 2.068682242 | 2.72E-07 | 1.33E-05 | UP |
| Galnt3 | 0.136503745 | 2.832780521 | -3.585001174 | 3.585001174 | 2.72E-07 | 1.33E-05 | DOWN |
| Reg3b | 0 | 21.28699123 | -10.70588328 | 10.70588328 | 2.76E-07 | 1.34E-05 | DOWN |
| Hemgn | 0.035555268 | 2.040309515 | -5.020663518 | 5.020663518 | 3.01E-07 | 1.45E-05 | DOWN |
| Dpys | 99.81691818 | 34.11005438 | 2.148316597 | 2.148316597 | 3.12E-07 | 1.49E-05 | UP |
| LOC102547056 | 0.290839879 | 1.881165966 | -2.023204451 | 2.023204451 | 3.24E-07 | 1.54E-05 | DOWN |
| LOC100360143 | 2.655520343 | 0 | 7.134632018 | 7.134632018 | 3.29E-07 | 1.56E-05 | UP |
| Elovl7 | 4.229855868 | 28.86874319 | -2.110682874 | 2.110682874 | 3.30E-07 | 1.56E-05 | DOWN |
| Cfhr1 | 308.3701819 | 111.2482673 | 2.062600804 | 2.062600804 | 3.40E-07 | 1.60E-05 | UP |
| Serpina3m | 218.4760666 | 39.66285081 | 3.023293274 | 3.023293274 | 3.59E-07 | 1.66E-05 | UP |
| Cd55 | 0.526234076 | 9.564851222 | -3.426574087 | 3.426574087 | 3.73E-07 | 1.71E-05 | DOWN |
| Fut2 | 0.034056496 | 3.883755321 | -6.080639809 | 6.080639809 | 3.78E-07 | 1.73E-05 | DOWN |
| Insc | 3.024172977 | 0.894682723 | 2.327894788 | 2.327894788 | 3.77E-07 | 1.73E-05 | UP |
| Capn8 | 0.042195655 | 1.168034345 | -4.099824765 | 4.099824765 | 3.85E-07 | 1.75E-05 | DOWN |
| Rin1 | 0.126187733 | 1.499335689 | -2.775477488 | 2.775477488 | 3.89E-07 | 1.76E-05 | DOWN |
| Akr1b10 | 54.8011521 | 344.5972112 | -2.001482182 | 2.001482182 | 4.09E-07 | 1.82E-05 | DOWN |
| LOC691352 | 0.019638618 | 5.204742149 | -6.618519696 | 6.618519696 | 4.13E-07 | 1.83E-05 | DOWN |
| Mlph | 0.168978296 | 2.873668502 | -3.446123126 | 3.446123126 | 4.29E-07 | 1.90E-05 | DOWN |
| Pcyox1l | 0.890159487 | 7.43439633 | -2.360150333 | 2.360150333 | 4.45E-07 | 1.95E-05 | DOWN |
| Tmem107 | 0.370363326 | 4.540899712 | -2.891864265 | 2.891864265 | 4.48E-07 | 1.95E-05 | DOWN |
| Usp2 | 7.088960699 | 2.377564248 | 2.147217044 | 2.147217044 | 4.51E-07 | 1.95E-05 | UP |
| Gprc5a | 0.218656255 | 6.901241961 | -4.158817011 | 4.158817011 | 4.57E-07 | 1.97E-05 | DOWN |
| Nlrc4 | 1.209270536 | 8.709550985 | -2.136005527 | 2.136005527 | 4.65E-07 | 1.99E-05 | DOWN |
| Cyp1a2 | 109.4934599 | 18.40036493 | 3.170390395 | 3.170390395 | 4.65E-07 | 1.99E-05 | UP |
| Il1a | 3.862232605 | 1.445950649 | 2.051767181 | 2.051767181 | 4.70E-07 | 2.00E-05 | UP |
| Irx3 | 0.073072867 | 2.38669501 | -4.295047367 | 4.295047367 | 4.71E-07 | 2.00E-05 | DOWN |
| Pdzk1ip1 | 1.200651173 | 10.48463371 | -2.417475864 | 2.417475864 | 4.75E-07 | 2.02E-05 | DOWN |
| LOC100911564 | 24.21908323 | 1.815237062 | 4.350238229 | 4.350238229 | 4.78E-07 | 2.02E-05 | UP |
| Krt7 | 0.615459815 | 12.41840621 | -3.505588563 | 3.505588563 | 5.10E-07 | 2.14E-05 | DOWN |
| Apoc3 | 1327.354895 | 266.7952268 | 2.920907949 | 2.920907949 | 5.18E-07 | 2.16E-05 | UP |
| Angptl6 | 8.217410368 | 2.145943335 | 2.673358519 | 2.673358519 | 5.52E-07 | 2.29E-05 | UP |
| Igf1 | 163.9334494 | 60.19175027 | 2.032259181 | 2.032259181 | 5.61E-07 | 2.32E-05 | UP |
| AABR07005844.1 | 24.7839196 | 231.8630928 | -2.531511815 | 2.531511815 | 5.76E-07 | 2.37E-05 | DOWN |
| Fstl3 | 0.563403922 | 4.922188117 | -2.430619987 | 2.430619987 | 5.88E-07 | 2.40E-05 | DOWN |
| AABR07032520.1 | 5.799289693 | 1.873834281 | 2.231592013 | 2.231592013 | 6.04E-07 | 2.46E-05 | UP |
| Tspan1 | 0.011169459 | 3.279449141 | -7.290511303 | 7.290511303 | 6.17E-07 | 2.50E-05 | DOWN |
| Slc4a11 | 0.040459763 | 1.618892377 | -4.500226099 | 4.500226099 | 6.71E-07 | 2.69E-05 | DOWN |
| Col1a1 | 15.98506875 | 141.4741431 | -2.39565198 | 2.39565198 | 6.83E-07 | 2.73E-05 | DOWN |
| Rtn1 | 0.612688441 | 4.174585106 | -2.078260292 | 2.078260292 | 7.01E-07 | 2.79E-05 | DOWN |
| LOC100910418 | 0.899682171 | 11.15000024 | -2.851574318 | 2.851574318 | 7.16E-07 | 2.84E-05 | DOWN |
| Tff1 | 0.14984006 | 39.37333854 | -7.110834765 | 7.110834765 | 7.37E-07 | 2.90E-05 | DOWN |
| Apoc1 | 274.368617 | 26.97693005 | 3.995856702 | 3.995856702 | 7.36E-07 | 2.90E-05 | UP |
| Lgals2 | 5.390113652 | 48.6290372 | -2.450554074 | 2.450554074 | 7.91E-07 | 3.10E-05 | DOWN |
| RGD1562392 | 2.207453996 | 0.564631419 | 2.532625576 | 2.532625576 | 7.93E-07 | 3.10E-05 | UP |
| Cdx2 | 0 | 4.682878104 | -9.913420615 | 9.913420615 | 8.21E-07 | 3.20E-05 | DOWN |
| Bmp8b | 0.010224096 | 3.156993604 | -6.625930994 | 6.625930994 | 8.41E-07 | 3.26E-05 | DOWN |
| LOC103691699 | 1.13738936 | 0.351878665 | 2.303314782 | 2.303314782 | 8.63E-07 | 3.34E-05 | UP |
| LOC100910057 | 1.876805677 | 0.273180298 | 3.310547023 | 3.310547023 | 8.79E-07 | 3.39E-05 | UP |
| Cyp2d2 | 495.509937 | 156.9483175 | 2.240478207 | 2.240478207 | 8.92E-07 | 3.42E-05 | UP |
| Myh14 | 1.037232237 | 8.165872282 | -2.20155647 | 2.20155647 | 8.90E-07 | 3.42E-05 | DOWN |
| Cdc45 | 0.131376163 | 1.656553178 | -2.932118517 | 2.932118517 | 9.09E-07 | 3.48E-05 | DOWN |
| Azgp1 | 412.3778918 | 96.42093246 | 2.670393866 | 2.670393866 | 9.65E-07 | 3.66E-05 | UP |
| Cyp3a9 | 16.71368813 | 2.920321756 | 3.11393512 | 3.11393512 | 9.85E-07 | 3.72E-05 | UP |
| Mtcl1 | 0.091548275 | 1.187756446 | -2.931174045 | 2.931174045 | 9.99E-07 | 3.76E-05 | DOWN |
| AABR07053500.2 | 0 | 19.97449112 | -9.310272924 | 9.310272924 | 1.01E-06 | 3.81E-05 | DOWN |
| Enpp2 | 44.35781117 | 16.17171439 | 2.034184255 | 2.034184255 | 1.05E-06 | 3.90E-05 | UP |
| Spp1 | 3.160717988 | 39.39168789 | -2.947540804 | 2.947540804 | 1.06E-06 | 3.92E-05 | DOWN |
| Gltpd2 | 62.42532076 | 21.56998649 | 2.093124217 | 2.093124217 | 1.08E-06 | 3.95E-05 | UP |
| Ttc22 | 0.176779457 | 2.40941475 | -3.078824198 | 3.078824198 | 1.17E-06 | 4.29E-05 | DOWN |
| Sptssb | 0.095505222 | 4.226551748 | -4.78457671 | 4.78457671 | 1.26E-06 | 4.54E-05 | DOWN |
| Kcnk1 | 0.188059345 | 1.733535517 | -2.554300158 | 2.554300158 | 1.31E-06 | 4.72E-05 | DOWN |
| Slc13a3 | 7.405895531 | 2.097137808 | 2.505923004 | 2.505923004 | 1.40E-06 | 5.01E-05 | UP |
| Fabp1 | 901.6137111 | 142.456571 | 3.281887477 | 3.281887477 | 1.40E-06 | 5.01E-05 | UP |
| Pkp1 | 0.069445542 | 1.233666784 | -3.344989927 | 3.344989927 | 1.48E-06 | 5.23E-05 | DOWN |
| Gmds | 1.680868337 | 12.96498326 | -2.244247187 | 2.244247187 | 1.52E-06 | 5.32E-05 | DOWN |
| Runx1 | 0.421140699 | 3.140379566 | -2.169635173 | 2.169635173 | 1.57E-06 | 5.50E-05 | DOWN |
| Pemt | 135.1004881 | 46.40949699 | 2.151335918 | 2.151335918 | 1.58E-06 | 5.52E-05 | UP |
| Fcrl2 | 0.384312894 | 6.983188305 | -3.531265165 | 3.531265165 | 1.64E-06 | 5.71E-05 | DOWN |
| Muc1 | 2.086987966 | 17.06220012 | -2.372995384 | 2.372995384 | 1.79E-06 | 6.16E-05 | DOWN |
| Slc51a | 0.048464126 | 2.636666191 | -4.983952765 | 4.983952765 | 1.87E-06 | 6.36E-05 | DOWN |
| Cx3cr1 | 1.481935657 | 10.17612108 | -2.088257046 | 2.088257046 | 1.87E-06 | 6.36E-05 | DOWN |
| Ramp3 | 0.211917628 | 4.363505139 | -3.572393103 | 3.572393103 | 1.95E-06 | 6.57E-05 | DOWN |
| Sftpd | 0.048995594 | 2.231510192 | -4.730525452 | 4.730525452 | 2.02E-06 | 6.80E-05 | DOWN |
| Spink1l | 254.2839372 | 88.16536799 | 2.090602869 | 2.090602869 | 2.07E-06 | 6.92E-05 | UP |
| Abcc6 | 22.7560972 | 8.520115379 | 2.011656348 | 2.011656348 | 2.10E-06 | 7.00E-05 | UP |
| LOC102552659 | 0.572000788 | 4.958438328 | -2.433419152 | 2.433419152 | 2.13E-06 | 7.07E-05 | DOWN |
| Galnt12 | 0.596232388 | 5.697509173 | -2.567977214 | 2.567977214 | 2.19E-06 | 7.24E-05 | DOWN |
| Crp | 1056.102055 | 317.8944327 | 2.299345739 | 2.299345739 | 2.28E-06 | 7.47E-05 | UP |
| LOC100910526 | 3.678684079 | 0.73380055 | 2.854777417 | 2.854777417 | 2.29E-06 | 7.50E-05 | UP |
| Ces1d | 235.5063139 | 90.05556663 | 2.026515643 | 2.026515643 | 2.31E-06 | 7.53E-05 | UP |
| Cdc25b | 0.492138239 | 3.806879084 | -2.247750954 | 2.247750954 | 2.35E-06 | 7.66E-05 | DOWN |
| Rundc3a | 0.140790678 | 2.48226649 | -3.356025933 | 3.356025933 | 2.46E-06 | 7.96E-05 | DOWN |
| Rab27b | 0.16638477 | 2.358684077 | -3.221032949 | 3.221032949 | 2.82E-06 | 8.98E-05 | DOWN |
| Car1 | 0.1749296 | 4.52056884 | -3.920154415 | 3.920154415 | 2.86E-06 | 9.09E-05 | DOWN |
| RGD1559600 | 22.35348563 | 7.024854473 | 2.238478279 | 2.238478279 | 2.90E-06 | 9.18E-05 | UP |
| Sh3rf2 | 0.115948137 | 2.262200909 | -3.556133173 | 3.556133173 | 2.91E-06 | 9.18E-05 | DOWN |
| Ahsp | 0.99688799 | 10.87475294 | -2.757701716 | 2.757701716 | 2.99E-06 | 9.39E-05 | DOWN |
| Myo1a | 0.010146758 | 1.76337434 | -6.389370227 | 6.389370227 | 3.09E-06 | 9.64E-05 | DOWN |
| Aoc1 | 0.714832697 | 4.846648149 | -2.018413631 | 2.018413631 | 3.11E-06 | 9.67E-05 | DOWN |
| Mmp9 | 0.117760175 | 1.422844942 | -2.862171267 | 2.862171267 | 3.16E-06 | 9.81E-05 | DOWN |
| Snrpn | 0.229882453 | 2.763804204 | -3.023352805 | 3.023352805 | 3.25E-06 | 0.000100179 | DOWN |
| Tox | 0.703352737 | 6.862395632 | -2.589071358 | 2.589071358 | 3.34E-06 | 0.000102448 | DOWN |
| Lrrc1 | 0.796855564 | 5.761143939 | -2.167345007 | 2.167345007 | 3.41E-06 | 0.000104409 | DOWN |
| Fhdc1 | 0.105612883 | 1.721081203 | -3.161479096 | 3.161479096 | 3.58E-06 | 0.000108199 | DOWN |
| Sulf1 | 0.744566108 | 4.981634346 | -2.010241819 | 2.010241819 | 3.64E-06 | 0.000109518 | DOWN |
| Slc7a11 | 3.200728978 | 57.97816165 | -3.461599465 | 3.461599465 | 3.64E-06 | 0.000109518 | DOWN |
| Eps8l1 | 0.119127055 | 1.273072643 | -2.722779094 | 2.722779094 | 3.74E-06 | 0.000112087 | DOWN |
| Kif1a | 0.139905112 | 1.761964029 | -3.029463935 | 3.029463935 | 4.02E-06 | 0.000118909 | DOWN |
| Mall | 0.126240314 | 4.581826138 | -4.356043377 | 4.356043377 | 4.12E-06 | 0.000121447 | DOWN |
| Vcan | 0.136973157 | 1.897802833 | -3.017707439 | 3.017707439 | 4.24E-06 | 0.000123976 | DOWN |
| LOC360919 | 64.92668093 | 22.25305538 | 2.133106412 | 2.133106412 | 4.29E-06 | 0.000125188 | UP |
| Cyp2c6v1 | 149.6796923 | 56.82630544 | 2.001358682 | 2.001358682 | 4.49E-06 | 0.000130185 | UP |
| Dtl | 0.140471893 | 1.371798841 | -2.601697712 | 2.601697712 | 4.51E-06 | 0.000130221 | DOWN |
| Tnc | 0.119150434 | 5.139408355 | -4.604290137 | 4.604290137 | 4.50E-06 | 0.000130221 | DOWN |
| Lypd8 | 0 | 57.57003441 | -12.61195318 | 12.61195318 | 4.54E-06 | 0.000130802 | DOWN |
| Hcn2 | 0.135449827 | 1.981753112 | -3.077844283 | 3.077844283 | 4.59E-06 | 0.000131279 | DOWN |
| Rbpjl | 0.097291177 | 1.17909177 | -2.844204856 | 2.844204856 | 4.60E-06 | 0.000131279 | DOWN |
| Ankrd1 | 0.628123051 | 4.904213466 | -2.235209445 | 2.235209445 | 4.73E-06 | 0.000133939 | DOWN |
| Grb10 | 0.961802716 | 6.719743883 | -2.081475175 | 2.081475175 | 4.85E-06 | 0.000136586 | DOWN |
| Lxn | 0.702713793 | 6.054754116 | -2.355504827 | 2.355504827 | 4.94E-06 | 0.000138787 | DOWN |
| Fst | 0.975817952 | 10.80614778 | -2.707671729 | 2.707671729 | 5.32E-06 | 0.00014769 | DOWN |
| Ap1m2 | 0.707860972 | 7.551797248 | -2.771865538 | 2.771865538 | 5.33E-06 | 0.000147796 | DOWN |
| Dhcr24 | 274.3046293 | 99.48613658 | 2.036791569 | 2.036791569 | 5.40E-06 | 0.000149056 | UP |
| Hrg | 551.6666157 | 146.2588132 | 2.445555898 | 2.445555898 | 5.45E-06 | 0.00015002 | UP |
| Igsf1 | 0.225415997 | 2.295778159 | -2.749868357 | 2.749868357 | 5.83E-06 | 0.000158444 | DOWN |
| LOC680875 | 3.710430063 | 0.661771362 | 3.12001556 | 3.12001556 | 5.87E-06 | 0.000158762 | UP |
| Gnmt | 100.8028339 | 20.54976819 | 2.844348576 | 2.844348576 | 5.96E-06 | 0.000160586 | UP |
| Slc16a11 | 3.137241869 | 1.058793192 | 2.243965266 | 2.243965266 | 6.03E-06 | 0.000161706 | UP |
| Notch3 | 0.633281268 | 4.409015008 | -2.042791306 | 2.042791306 | 6.12E-06 | 0.000163459 | DOWN |
| Vnn3 | 11.69404259 | 2.853730133 | 2.594208309 | 2.594208309 | 6.14E-06 | 0.000163753 | UP |
| Itga2 | 0.255084246 | 2.551121081 | -2.608937745 | 2.608937745 | 6.24E-06 | 0.0001655 | DOWN |
| Pcdh17 | 0.474101528 | 3.663779241 | -2.326039884 | 2.326039884 | 6.25E-06 | 0.000165711 | DOWN |
| Dyrk3 | 0.102781466 | 1.439010501 | -3.155663352 | 3.155663352 | 6.84E-06 | 0.00017927 | DOWN |
| Sat2 | 2.229688014 | 0.593681 | 2.62662551 | 2.62662551 | 6.88E-06 | 0.000179588 | UP |
| S100a8 | 2.685254225 | 18.63590707 | -2.139379104 | 2.139379104 | 6.96E-06 | 0.000180982 | DOWN |
| RGD1559588 | 0.260777467 | 4.607633451 | -3.3099445 | 3.3099445 | 7.14E-06 | 0.00018457 | DOWN |
| Hba-a2 | 49.32993826 | 520.7684275 | -2.71049504 | 2.71049504 | 7.18E-06 | 0.000185171 | DOWN |
| Thy1 | 0.83167252 | 6.617394483 | -2.317128962 | 2.317128962 | 7.37E-06 | 0.000189555 | DOWN |
| Anxa8 | 1.02243415 | 8.20535296 | -2.263667622 | 2.263667622 | 7.45E-06 | 0.000191037 | DOWN |
| Gpbar1 | 0.122008408 | 2.219408907 | -3.554043417 | 3.554043417 | 7.49E-06 | 0.000191428 | DOWN |
| Ckmt1 | 0.197113738 | 5.746948086 | -4.224005888 | 4.224005888 | 7.57E-06 | 0.000192487 | DOWN |
| LOC103694857 | 30.79075399 | 295.7883644 | -2.629806498 | 2.629806498 | 7.56E-06 | 0.000192487 | DOWN |
| Rab25 | 1.491495383 | 12.40413669 | -2.400063674 | 2.400063674 | 7.72E-06 | 0.000195235 | DOWN |
| Slc7a2 | 201.8347306 | 63.53213812 | 2.211053778 | 2.211053778 | 7.73E-06 | 0.000195458 | UP |
| Igfals | 53.95318006 | 11.79211435 | 2.807370906 | 2.807370906 | 8.24E-06 | 0.000206333 | UP |
| Palm2 | 2.344711444 | 0.639526338 | 2.452659684 | 2.452659684 | 8.30E-06 | 0.000207647 | UP |
| Sfn | 1.786140495 | 13.46773735 | -2.172306326 | 2.172306326 | 8.35E-06 | 0.000208246 | DOWN |
| Matn4 | 0.203354437 | 2.040187906 | -2.621304774 | 2.621304774 | 8.57E-06 | 0.000213192 | DOWN |
| Gpihbp1 | 0.166883049 | 4.21389208 | -3.869648494 | 3.869648494 | 9.46E-06 | 0.000230822 | DOWN |
| Chaf1b | 0.18821999 | 1.957577654 | -2.674936222 | 2.674936222 | 9.53E-06 | 0.000232139 | DOWN |
| Cldn6 | 0.697596405 | 5.746450634 | -2.354815837 | 2.354815837 | 9.78E-06 | 0.000236249 | DOWN |
| NEWGENE_2134 | 739.8955089 | 204.302443 | 2.474639756 | 2.474639756 | 1.02E-05 | 0.000243551 | UP |
| Irx1 | 1.678512457 | 0.227447284 | 3.497362154 | 3.497362154 | 1.04E-05 | 0.000246962 | UP |
| Krt80 | 0.045198098 | 1.354865742 | -4.090775972 | 4.090775972 | 1.04E-05 | 0.000247358 | DOWN |
| Fxyd1 | 41.68346461 | 16.66257971 | 2.014678337 | 2.014678337 | 1.09E-05 | 0.000257488 | UP |
| LOC103693210 | 0.686584594 | 7.276778028 | -2.669031543 | 2.669031543 | 1.13E-05 | 0.000264464 | DOWN |
| Alas2 | 1.07242951 | 11.78298772 | -2.823779712 | 2.823779712 | 1.14E-05 | 0.000265932 | DOWN |
| Fap | 0.063466571 | 1.229133827 | -3.570346336 | 3.570346336 | 1.19E-05 | 0.000276243 | DOWN |
| Ninl | 0.454345238 | 2.96643068 | -2.003511428 | 2.003511428 | 1.20E-05 | 0.000278829 | DOWN |
| Elfn1 | 0.29592796 | 2.316133881 | -2.330511322 | 2.330511322 | 1.20E-05 | 0.000278904 | DOWN |
| Cbr3 | 1.599877276 | 14.29668756 | -2.438758065 | 2.438758065 | 1.21E-05 | 0.000280224 | DOWN |
| Nat8f2 | 6.964567274 | 2.35951669 | 2.202580929 | 2.202580929 | 1.24E-05 | 0.000285792 | UP |
| Slc14a1 | 0.113583956 | 2.397028003 | -3.56794841 | 3.56794841 | 1.26E-05 | 0.000289671 | DOWN |
| Aoah | 0.448930186 | 3.222536334 | -2.148372973 | 2.148372973 | 1.26E-05 | 0.000290857 | DOWN |
| B3galt5 | 0.220362189 | 1.663609686 | -2.27175257 | 2.27175257 | 1.27E-05 | 0.000291862 | DOWN |
| Bcat1 | 0.187400728 | 2.330673712 | -2.860636569 | 2.860636569 | 1.30E-05 | 0.000297186 | DOWN |
| Psca | 0.39926632 | 11.89883524 | -3.997803934 | 3.997803934 | 1.32E-05 | 0.000300691 | DOWN |
| Dusp4 | 0.843853871 | 6.405028021 | -2.175770747 | 2.175770747 | 1.35E-05 | 0.000307407 | DOWN |
| Prap1 | 0 | 1.584289773 | -6.752079922 | 6.752079922 | 1.38E-05 | 0.000312783 | DOWN |
| Heph | 2.099965397 | 15.33832685 | -2.202847927 | 2.202847927 | 1.42E-05 | 0.000319717 | DOWN |
| Igf2 | 0.119390818 | 5.741057737 | -4.763281305 | 4.763281305 | 1.59E-05 | 0.000352317 | DOWN |
| AC119762.3 | 0.08251858 | 1.318946114 | -3.168983789 | 3.168983789 | 1.59E-05 | 0.000352317 | DOWN |
| Ccnd2 | 4.369651135 | 34.51665302 | -2.220232807 | 2.220232807 | 1.60E-05 | 0.000352592 | DOWN |
| Rasef | 0.388692306 | 3.303296315 | -2.432877882 | 2.432877882 | 1.66E-05 | 0.000364486 | DOWN |
| LOC100362350 | 120.477152 | 22.8772409 | 2.934826826 | 2.934826826 | 1.67E-05 | 0.000365522 | UP |
| Aqp3 | 0.185696887 | 3.720392959 | -3.5709938 | 3.5709938 | 1.73E-05 | 0.000375613 | DOWN |
| Mgat3 | 0.865330214 | 5.898733973 | -2.054690613 | 2.054690613 | 1.75E-05 | 0.000379465 | DOWN |
| Adra1b | 8.423322446 | 3.110148739 | 2.020403928 | 2.020403928 | 1.82E-05 | 0.000392875 | UP |
| Ankrd22 | 0.578488955 | 4.673347866 | -2.363721278 | 2.363721278 | 1.90E-05 | 0.000407332 | DOWN |
| Rflnb | 1.842517451 | 12.58970237 | -2.050449125 | 2.050449125 | 1.90E-05 | 0.000407336 | DOWN |
| Prr15l | 1.240190264 | 13.89947 | -2.71025833 | 2.71025833 | 1.94E-05 | 0.000414891 | DOWN |
| Vtcn1 | 0.160019752 | 1.341811895 | -2.403170553 | 2.403170553 | 1.96E-05 | 0.000418428 | DOWN |
| AABR07031918.1 | 1.890026159 | 0.32682673 | 3.140541224 | 3.140541224 | 1.97E-05 | 0.000420172 | UP |
| Agt | 394.8534904 | 135.8687601 | 2.105180936 | 2.105180936 | 2.02E-05 | 0.000427555 | UP |
| Mmp8 | 0.155809276 | 2.132608771 | -3.154647865 | 3.154647865 | 2.03E-05 | 0.000428266 | DOWN |
| Jag2 | 0.257425426 | 1.877432328 | -2.1252875 | 2.1252875 | 2.05E-05 | 0.000431838 | DOWN |
| Spic | 0.45014008 | 4.21174711 | -2.546252063 | 2.546252063 | 2.08E-05 | 0.000436627 | DOWN |
| Col15a1 | 0.204688458 | 3.835449416 | -3.506416582 | 3.506416582 | 2.14E-05 | 0.000447241 | DOWN |
| Hsd17b6 | 58.05675868 | 11.64546779 | 2.854929025 | 2.854929025 | 2.17E-05 | 0.000452388 | UP |
| Fcgbp | 0.07303224 | 1.259793393 | -3.344157183 | 3.344157183 | 2.22E-05 | 0.000460521 | DOWN |
| N4bp3 | 0.283645593 | 1.90303809 | -2.048126798 | 2.048126798 | 2.24E-05 | 0.000462134 | DOWN |
| Ksr2 | 2.366625435 | 0.726893563 | 2.35147831 | 2.35147831 | 2.24E-05 | 0.000462134 | UP |
| Gkn2 | 0.017040161 | 8.6439905 | -7.33828383 | 7.33828383 | 2.27E-05 | 0.00046692 | DOWN |
| Pou2f2 | 0.159143616 | 1.14201187 | -2.102041865 | 2.102041865 | 2.28E-05 | 0.000468952 | DOWN |
| Ptk7 | 0.625724669 | 4.356347434 | -2.094435359 | 2.094435359 | 2.30E-05 | 0.000470944 | DOWN |
| Sncg | 0.47492655 | 6.572290272 | -2.968822835 | 2.968822835 | 2.36E-05 | 0.000481804 | DOWN |
| Mtmr7 | 1.522228434 | 0.520039853 | 2.136106669 | 2.136106669 | 2.38E-05 | 0.00048373 | UP |
| Tnfrsf14 | 0.510938616 | 4.508743239 | -2.347492014 | 2.347492014 | 2.58E-05 | 0.000517741 | DOWN |
| LOC103690070 | 0.452035377 | 9.633419643 | -3.666095419 | 3.666095419 | 2.76E-05 | 0.00054737 | DOWN |
| Cyp2b2 | 18.05273527 | 6.279857392 | 2.110654861 | 2.110654861 | 2.79E-05 | 0.000551784 | UP |
| Cbarp | 0.337934725 | 2.558801635 | -2.160046367 | 2.160046367 | 2.94E-05 | 0.000574368 | DOWN |
| Muc13 | 0 | 8.746934611 | -11.99808491 | 11.99808491 | 3.05E-05 | 0.000592405 | DOWN |
| Clic6 | 0.061216445 | 2.131578421 | -4.466607684 | 4.466607684 | 3.09E-05 | 0.000598349 | DOWN |
| Pimreg | 0.051277422 | 1.274473155 | -3.501153115 | 3.501153115 | 3.47E-05 | 0.000661543 | DOWN |
| Nol4l | 0.463463575 | 3.934429459 | -2.361030128 | 2.361030128 | 3.49E-05 | 0.000664444 | DOWN |
| Ms4a12 | 0 | 1.8993369 | -7.288912371 | 7.288912371 | 3.63E-05 | 0.000686304 | DOWN |
| Chst11 | 0.136650974 | 1.051447309 | -2.257207205 | 2.257207205 | 3.68E-05 | 0.000693508 | DOWN |
| Cdhr5 | 0.64135541 | 7.495850699 | -2.843648039 | 2.843648039 | 3.72E-05 | 0.000697912 | DOWN |
| LOC498222 | 0.449171788 | 4.350471864 | -2.557729419 | 2.557729419 | 3.94E-05 | 0.00073306 | DOWN |
| Upp2 | 10.20413293 | 2.431075096 | 2.698955377 | 2.698955377 | 4.00E-05 | 0.000741738 | UP |
| Igsf9 | 0.19801121 | 1.454038528 | -2.1811983 | 2.1811983 | 4.06E-05 | 0.000749779 | DOWN |
| Ppp1r36 | 0.183768787 | 3.209324677 | -3.198997693 | 3.198997693 | 4.16E-05 | 0.000764195 | DOWN |
| LOC103694855 | 10.1352562 | 81.52683869 | -2.361910509 | 2.361910509 | 4.19E-05 | 0.000767566 | DOWN |
| Tsx | 5.069287187 | 1.000327017 | 2.922831371 | 2.922831371 | 4.22E-05 | 0.000771223 | UP |
| Amacr | 79.4500315 | 26.24464329 | 2.227787584 | 2.227787584 | 4.22E-05 | 0.000771223 | UP |
| Cbs | 49.9592903 | 13.31093015 | 2.44558125 | 2.44558125 | 4.23E-05 | 0.000771363 | UP |
| Ccl20 | 0.449081889 | 5.855709129 | -3.132827571 | 3.132827571 | 4.25E-05 | 0.000773919 | DOWN |
| Cdkn2b | 0.592171782 | 7.305433162 | -2.930954202 | 2.930954202 | 4.27E-05 | 0.000777219 | DOWN |
| Hba-a2 | 127.4705611 | 1181.925348 | -2.522162628 | 2.522162628 | 4.39E-05 | 0.000794591 | DOWN |
| Hk2 | 0.802938686 | 5.480072296 | -2.024968608 | 2.024968608 | 4.52E-05 | 0.000814027 | DOWN |
| Agr2 | 0 | 6.040033318 | -10.18972813 | 10.18972813 | 4.68E-05 | 0.000836352 | DOWN |
| LOC685048 | 0.118097182 | 2.322154028 | -3.57726613 | 3.57726613 | 4.69E-05 | 0.000838588 | DOWN |
| S100b | 0.633515602 | 4.349333397 | -2.128649459 | 2.128649459 | 4.77E-05 | 0.000849315 | DOWN |
| Tnnt2 | 0.596903558 | 5.27447705 | -2.34723044 | 2.34723044 | 4.79E-05 | 0.000850898 | DOWN |
| Egflam | 0.394134055 | 3.265705671 | -2.363561554 | 2.363561554 | 4.80E-05 | 0.000852363 | DOWN |
| Pou2af1 | 0.244821942 | 2.537900143 | -2.596748712 | 2.596748712 | 4.88E-05 | 0.000863772 | DOWN |
| Akr1c3 | 3.38496681 | 0.382146918 | 3.621600338 | 3.621600338 | 4.88E-05 | 0.000863772 | UP |
| Scin | 0.120234765 | 1.713633879 | -3.068882313 | 3.068882313 | 4.97E-05 | 0.000876613 | DOWN |
| RGD1307603 | 31.65654916 | 5.170480595 | 3.193179946 | 3.193179946 | 4.98E-05 | 0.000877244 | UP |
| Tmem45b | 0.058824427 | 9.184183355 | -6.524380224 | 6.524380224 | 5.01E-05 | 0.000881292 | DOWN |
| Tmem163 | 2.172241638 | 0.730732287 | 2.219771623 | 2.219771623 | 5.02E-05 | 0.000881827 | UP |
| G0s2 | 114.373207 | 39.25904795 | 2.064772418 | 2.064772418 | 5.06E-05 | 0.000886995 | UP |
| Sema7a | 0.309195807 | 3.124564808 | -2.552209348 | 2.552209348 | 5.19E-05 | 0.000906533 | DOWN |
| Ephb2 | 0.210963725 | 1.499760588 | -2.115381807 | 2.115381807 | 5.27E-05 | 0.000917839 | DOWN |
| Coro2a | 0.218093117 | 2.757709963 | -2.852293516 | 2.852293516 | 5.39E-05 | 0.000935665 | DOWN |
| Pnpla3 | 1.433610765 | 0.547982234 | 2.061389211 | 2.061389211 | 5.49E-05 | 0.000948808 | UP |
| Anks6 | 0.153996387 | 1.160239219 | -2.2597659 | 2.2597659 | 5.54E-05 | 0.0009552 | DOWN |
| Tesc | 0.209715651 | 4.389240772 | -3.544878034 | 3.544878034 | 6.19E-05 | 0.001048719 | DOWN |
| Adamts8 | 0.285289393 | 2.271638392 | -2.239748495 | 2.239748495 | 6.29E-05 | 0.001061733 | DOWN |
| Tubb4a | 0.155963636 | 1.754992409 | -2.882419644 | 2.882419644 | 6.31E-05 | 0.001064337 | DOWN |
| Olfm1 | 0.231452987 | 2.486674309 | -2.731456133 | 2.731456133 | 6.45E-05 | 0.001079724 | DOWN |
| Cfb | 13.25192955 | 1.217820237 | 4.029637521 | 4.029637521 | 6.59E-05 | 0.00109896 | UP |
| Cyp2s1 | 0.239176546 | 1.862049347 | -2.197091504 | 2.197091504 | 6.86E-05 | 0.001136601 | DOWN |
| Aspdh | 21.78456304 | 7.483379616 | 2.126361723 | 2.126361723 | 6.98E-05 | 0.001153657 | UP |
| Ms4a7 | 0.446415198 | 4.152970603 | -2.490979249 | 2.490979249 | 7.66E-05 | 0.00124603 | DOWN |
| Osbpl6 | 0.186058752 | 1.255408133 | -2.061216634 | 2.061216634 | 7.78E-05 | 0.001256349 | DOWN |
| Adap1 | 0.406702511 | 3.006234621 | -2.137104125 | 2.137104125 | 7.94E-05 | 0.00127582 | DOWN |
| AABR07006269.1 | 0.894305811 | 6.30644124 | -2.134186178 | 2.134186178 | 8.02E-05 | 0.001285943 | DOWN |
| Angpt2 | 0.369318023 | 3.058137382 | -2.347728083 | 2.347728083 | 8.29E-05 | 0.001325678 | DOWN |
| Ly75 | 0.306275631 | 3.228140173 | -2.63579606 | 2.63579606 | 8.42E-05 | 0.001341611 | DOWN |
| Sirpd | 0 | 1.568171325 | -7.508605403 | 7.508605403 | 8.59E-05 | 0.001366505 | DOWN |
| LOC103694855 | 10.28786757 | 77.18331529 | -2.240000221 | 2.240000221 | 8.76E-05 | 0.001388333 | DOWN |
| LOC688335 | 1.281977818 | 0 | 6.078728891 | 6.078728891 | 8.78E-05 | 0.001390057 | UP |
| Syt13 | 0.110579523 | 1.311746185 | -2.878454944 | 2.878454944 | 9.22E-05 | 0.00145129 | DOWN |
| Syt8 | 0.107988903 | 4.055978634 | -4.449576952 | 4.449576952 | 9.26E-05 | 0.001455486 | DOWN |
| Gpr176 | 0.364993383 | 2.564043825 | -2.143873135 | 2.143873135 | 9.39E-05 | 0.001470053 | DOWN |
| Bcl2l14 | 0.646352578 | 4.3469688 | -2.01167311 | 2.01167311 | 9.91E-05 | 0.001538407 | DOWN |
| Epn3 | 0.094182448 | 1.512180918 | -3.332018049 | 3.332018049 | 0.000101193 | 0.00156585 | DOWN |
| Clec2e | 0.02358667 | 2.47565005 | -5.629279686 | 5.629279686 | 0.000102079 | 0.001577061 | DOWN |
| Nudt11 | 0.403255546 | 2.984553163 | -2.187258018 | 2.187258018 | 0.00010545 | 0.001622742 | DOWN |
| AABR07034736.1 | 1.576192117 | 0 | 6.28861855 | 6.28861855 | 0.000107379 | 0.001647235 | UP |
| Csdc2 | 0.060131654 | 1.003235331 | -3.315250283 | 3.315250283 | 0.000109124 | 0.001670071 | DOWN |
| Slc30a3 | 2.959962884 | 1.179581093 | 2.038316662 | 2.038316662 | 0.000112801 | 0.001710056 | UP |
| Sez6 | 7.723238597 | 2.128769398 | 2.369211286 | 2.369211286 | 0.000112626 | 0.001710056 | UP |
| Fam84a | 0.142637957 | 1.111457322 | -2.317767259 | 2.317767259 | 0.000120203 | 0.001793937 | DOWN |
| Nrgn | 0.360443335 | 3.112762529 | -2.363735594 | 2.363735594 | 0.00012662 | 0.001870514 | DOWN |
| Dusp2 | 0.55543906 | 3.911039509 | -2.068873104 | 2.068873104 | 0.000126928 | 0.001873649 | DOWN |
| LOC679149 | 0.140285618 | 4.149587431 | -4.071994815 | 4.071994815 | 0.000130612 | 0.001920788 | DOWN |
| Fosl1 | 0.115676223 | 3.03466496 | -3.947279308 | 3.947279308 | 0.000130996 | 0.00192485 | DOWN |
| Cbln3 | 2.984763422 | 0.587634669 | 2.918763705 | 2.918763705 | 0.000137396 | 0.002002482 | UP |
| Cubn | 0.080046908 | 1.050441127 | -3.029889282 | 3.029889282 | 0.000137846 | 0.002006042 | DOWN |
| Rasl12 | 0.095416965 | 1.027725921 | -2.589824506 | 2.589824506 | 0.000139619 | 0.002027332 | DOWN |
| Nts | 0.307394871 | 4.366522383 | -3.260846226 | 3.260846226 | 0.000145782 | 0.002096207 | DOWN |
| Ildr1 | 0.866483329 | 5.593112641 | -2.048326638 | 2.048326638 | 0.000146311 | 0.002100407 | DOWN |
| Cabp2 | 7.461663418 | 1.511524537 | 2.917342298 | 2.917342298 | 0.000155866 | 0.002210727 | UP |
| Acta1 | 0.193434838 | 1.8339202 | -2.608376075 | 2.608376075 | 0.000160716 | 0.002271276 | DOWN |
| Pde9a | 0.05847429 | 1.049176173 | -3.428829344 | 3.428829344 | 0.000167729 | 0.002339948 | DOWN |
| Prr7 | 0.734644708 | 6.940597403 | -2.44559197 | 2.44559197 | 0.000167597 | 0.002339948 | DOWN |
| Slc27a5 | 137.4334502 | 39.00889825 | 2.342438963 | 2.342438963 | 0.000169462 | 0.002359073 | UP |
| E2f8 | 0.257239849 | 1.964721256 | -2.269628992 | 2.269628992 | 0.00017136 | 0.002378731 | DOWN |
| Adam12 | 0.222313973 | 1.824322652 | -2.22077061 | 2.22077061 | 0.000177128 | 0.002443189 | DOWN |
| Trem1 | 0.164640733 | 2.039274898 | -2.852121352 | 2.852121352 | 0.000185997 | 0.002540445 | DOWN |
| Auts2 | 0.152792158 | 1.043828075 | -2.065769248 | 2.065769248 | 0.000188013 | 0.002559041 | DOWN |
| AC128848.1 | 60.72854754 | 13.76252159 | 2.802491913 | 2.802491913 | 0.000200415 | 0.002697806 | UP |
| Lum | 0.389108041 | 4.651347186 | -2.856219108 | 2.856219108 | 0.000210404 | 0.002803327 | DOWN |
| Hamp | 637.1184196 | 264.0745045 | 2.032756328 | 2.032756328 | 0.000211101 | 0.00281071 | UP |
| Hunk | 0.147328813 | 1.189362879 | -2.316606172 | 2.316606172 | 0.000216516 | 0.002868232 | DOWN |
| Padi4 | 0.273707772 | 3.920222062 | -3.020196464 | 3.020196464 | 0.000218437 | 0.002886759 | DOWN |
| Krt12 | 0.106947091 | 3.967835838 | -4.476473257 | 4.476473257 | 0.000221623 | 0.002924911 | DOWN |
| Aspg | 24.29928328 | 7.02310696 | 2.345720941 | 2.345720941 | 0.000223523 | 0.002940067 | UP |
| Lcn12 | 2.939303366 | 1.059713426 | 2.106925032 | 2.106925032 | 0.000224768 | 0.002948519 | UP |
| Slc1a2 | 6.759250102 | 2.072594138 | 2.284170788 | 2.284170788 | 0.000233092 | 0.003043417 | UP |
| Gdf6 | 0.196173264 | 1.324676928 | -2.143053292 | 2.143053292 | 0.000234407 | 0.003052437 | DOWN |
| AABR07072184.1 | 11.22743671 | 0.68232192 | 4.587933483 | 4.587933483 | 0.000241305 | 0.00312977 | UP |
| Ush1c | 0.093676161 | 2.509830512 | -4.121561684 | 4.121561684 | 0.000244571 | 0.003161638 | DOWN |
| Itgb7 | 0.740994807 | 5.384190257 | -2.085641193 | 2.085641193 | 0.000249939 | 0.003216265 | DOWN |
| Ntf3 | 1.698880277 | 0.548934002 | 2.277688275 | 2.277688275 | 0.00026086 | 0.003330387 | UP |
| Ypel1 | 0 | 1.464160017 | -5.967337598 | 5.967337598 | 0.000262071 | 0.003343668 | DOWN |
| Cst6 | 0.044092442 | 1.724266798 | -3.988559689 | 3.988559689 | 0.000267361 | 0.003391252 | DOWN |
| LOC103689993 | 1.263756021 | 0 | 6.850198859 | 6.850198859 | 0.000269918 | 0.003414829 | UP |
| Mt1 | 472.7738112 | 86.45435793 | 3.104791936 | 3.104791936 | 0.000272866 | 0.003438778 | UP |
| LOC688459 | 0.009401468 | 1.202624663 | -5.406687344 | 5.406687344 | 0.000279721 | 0.003498125 | DOWN |
| LOC501038 | 2.453898455 | 0.537223979 | 2.729439887 | 2.729439887 | 0.000292058 | 0.003619998 | UP |
| Unc93a | 0.200978743 | 1.970733557 | -2.781777739 | 2.781777739 | 0.000305582 | 0.00376063 | DOWN |
| Rnase1l1 | 0 | 2.803105662 | -7.158247657 | 7.158247657 | 0.000313726 | 0.003837508 | DOWN |
| Tnfrsf9 | 0.331584878 | 2.405569244 | -2.138676986 | 2.138676986 | 0.000315156 | 0.003852589 | DOWN |
| Kcne3 | 0.172780471 | 2.691742531 | -3.197385129 | 3.197385129 | 0.000321596 | 0.003909324 | DOWN |
| AC114111.1 | 3.970492163 | 0.950125889 | 2.749032955 | 2.749032955 | 0.000328674 | 0.003974701 | UP |
| Ces2e | 0.317087775 | 12.24231688 | -4.438937894 | 4.438937894 | 0.000330444 | 0.003992065 | DOWN |
| Svop | 0.136238388 | 1.036658744 | -2.208057678 | 2.208057678 | 0.000344477 | 0.004116134 | DOWN |
| Atf7ip2 | 0.20758334 | 1.962808476 | -2.546059919 | 2.546059919 | 0.000344502 | 0.004116134 | DOWN |
| Ptger4 | 0.989696119 | 6.517094411 | -2.041646871 | 2.041646871 | 0.000364751 | 0.004315888 | DOWN |
| Mt1m | 185.1919572 | 41.24045307 | 2.845354437 | 2.845354437 | 0.000380833 | 0.004454968 | UP |
| Ovol2 | 0.112371569 | 1.822913793 | -3.210660586 | 3.210660586 | 0.000396945 | 0.004588562 | DOWN |
| Slc16a3 | 0.255444104 | 2.224291482 | -2.3238381 | 2.3238381 | 0.000406559 | 0.004669346 | DOWN |
| AABR07054000.1 | 3.980465115 | 1.555153867 | 2.012078664 | 2.012078664 | 0.000428376 | 0.004868455 | UP |
| Serpinb1a | 40.51098792 | 344.1522393 | -2.385817622 | 2.385817622 | 0.000441701 | 0.004999554 | DOWN |
| Slc1a1 | 0.071979971 | 1.271567891 | -3.40802165 | 3.40802165 | 0.000442055 | 0.005000666 | DOWN |
| Als2cr12 | 0.134905543 | 1.070937551 | -2.34564634 | 2.34564634 | 0.000450828 | 0.005091075 | DOWN |
| Miga1 | 0.246848592 | 1.646236786 | -2.016610694 | 2.016610694 | 0.000452117 | 0.00509975 | DOWN |
| LOC497796 | 0.553647933 | 3.67803951 | -2.095074684 | 2.095074684 | 0.000488361 | 0.005427185 | DOWN |
| LOC100911516 | 79.86119181 | 31.63963009 | 2.042295237 | 2.042295237 | 0.000512288 | 0.005622809 | UP |
| Guca2b | 0.216210104 | 5.70894525 | -3.80921492 | 3.80921492 | 0.000519842 | 0.005689751 | DOWN |
| Apoc2 | 338.9820486 | 121.2844982 | 2.106664659 | 2.106664659 | 0.000531257 | 0.005792007 | UP |
| LOC100911825 | 0.209445424 | 1.36525253 | -2.070681518 | 2.070681518 | 0.00055603 | 0.006021809 | DOWN |
| Reg3g | 0 | 1.799356354 | -7.146775124 | 7.146775124 | 0.000556451 | 0.006023031 | DOWN |
| Serpinb5 | 0.127556146 | 1.885182026 | -3.224939643 | 3.224939643 | 0.000562225 | 0.006076865 | DOWN |
| Nim1k | 1.242346248 | 0.424163817 | 2.131162488 | 2.131162488 | 0.000563696 | 0.006087974 | UP |
| Tcf24 | 1.460477504 | 0.370016634 | 2.657602458 | 2.657602458 | 0.000602979 | 0.006434081 | UP |
| B4galt2 | 0.214193979 | 1.866673963 | -2.277057618 | 2.277057618 | 0.000615365 | 0.006535244 | DOWN |
| Has2 | 0.107137371 | 1.285984233 | -2.879336096 | 2.879336096 | 0.000641383 | 0.006722837 | DOWN |
| Dpm3 | 6.863536473 | 1.584730352 | 2.800027727 | 2.800027727 | 0.000643797 | 0.006744526 | UP |
| Col13a1 | 1.193207972 | 0.394794312 | 2.275796454 | 2.275796454 | 0.000655168 | 0.006841663 | UP |
| Ugt2b10 | 115.6543632 | 35.96892094 | 2.206536206 | 2.206536206 | 0.000663513 | 0.006910979 | UP |
| Postn | 0.380423545 | 3.651516615 | -2.523489762 | 2.523489762 | 0.000668358 | 0.006942334 | DOWN |
| Cst7 | 0.620457586 | 4.373202486 | -2.110668571 | 2.110668571 | 0.000695966 | 0.007173093 | DOWN |
| Grhl3 | 0.119942663 | 1.128048118 | -2.424521439 | 2.424521439 | 0.00070934 | 0.007276732 | DOWN |
| Gjb5 | 0.094251625 | 1.672713051 | -3.395131749 | 3.395131749 | 0.000720612 | 0.007380205 | DOWN |
| Hepacam | 0.120271851 | 2.107085266 | -3.637637338 | 3.637637338 | 0.000753954 | 0.007652613 | DOWN |
| Apobec3 | 0.235431384 | 2.842087654 | -2.738881235 | 2.738881235 | 0.000764047 | 0.007730989 | DOWN |
| Gucy2c | 1.258484549 | 10.19120385 | -2.450107804 | 2.450107804 | 0.000811791 | 0.008125803 | DOWN |
| Hoxb7 | 0.153222434 | 1.421373044 | -2.666538243 | 2.666538243 | 0.000814129 | 0.008140876 | DOWN |
| Guca2a | 0.071189879 | 2.989360594 | -4.625024487 | 4.625024487 | 0.000827788 | 0.008247939 | DOWN |
| Mogat2 | 0.133139574 | 1.457974684 | -2.817907123 | 2.817907123 | 0.000850333 | 0.008446759 | DOWN |

**Table S3 Topological feature values of nodes in the interaction network of disease gene-drug target**

| **node_name** | **MCC** | **Degree** | **Closeness** | **Betweenness** | **Stress** | **ClusteringCoefficient** |
| --- | --- | --- | --- | --- | --- | --- |
| Slc23a1 | 36 | 12 | 598.8 | 3813.709 | 39634 | 0.15152 |
| Vwa1 | 37 | 13 | 613.1667 | 5865.846 | 93486 | 0.12821 |
| Npr2 | 61 | 14 | 611.25 | 2330.249 | 32804 | 0.18681 |
| Reck | 126 | 15 | 605.7333 | 3158.844 | 55914 | 0.21905 |
| Igfbp7 | 104 | 15 | 632.0167 | 2518.065 | 38198 | 0.2 |
| Slc16a2 | 59 | 18 | 588.5333 | 5044.818 | 52438 | 0.13072 |
| Fblim1 | 1082 | 20 | 635.7333 | 7502.981 | 125000 | 0.26842 |
| Dpysl3 | 160 | 15 | 597.8667 | 2288.353 | 27248 | 0.21905 |
| Tnc | 47245 | 26 | 663.6 | 2838.642 | 54410 | 0.30769 |
| Loxl2 | 6063124 | 21 | 604.4667 | 2718.19 | 46258 | 0.54286 |
| Tgfbi | 11541 | 15 | 614.1333 | 1875.882 | 44476 | 0.41905 |
| PCOLCE2 | 40338 | 20 | 619.0167 | 17179.93 | 308874 | 0.16842 |
| Col8a2 | 1.39E+12 | 20 | 604.8333 | 5283.997 | 122766 | 0.63158 |
| Itga11 | 1.46E+07 | 31 | 684.0833 | 5892.418 | 125124 | 0.42796 |
| Ltbp2 | 13699 | 19 | 622.9 | 3474.386 | 50570 | 0.29825 |
| Rtn4 | 39 | 19 | 602.3 | 6238.297 | 68248 | 0.08772 |
| Hs3st1 | 43 | 16 | 608.95 | 6629.633 | 75024 | 0.10833 |
| Tuba1a | 4.90E+07 | 29 | 685.3333 | 7919.719 | 161642 | 0.36207 |
| Dctd | 256 | 12 | 596.55 | 2602.781 | 38420 | 0.33333 |
| Tacstd2 | 48 | 15 | 619.9333 | 2136.315 | 29838 | 0.17143 |
| Mfap4 | 209658 | 21 | 624.1167 | 2391.071 | 43340 | 0.37619 |
| Esrp1 | 45 | 14 | 640.1167 | 4237.418 | 51724 | 0.17582 |
| Rgs1 | 276 | 15 | 604.6167 | 3072.109 | 34310 | 0.26667 |
| Fcer1a | 194 | 19 | 616.8167 | 3932.331 | 62778 | 0.22222 |
| Rgs4 | 159 | 14 | 633.55 | 3797.13 | 42716 | 0.20879 |
| Cdkn2b | 7339036 | 23 | 680.0167 | 6409.873 | 119074 | 0.35968 |
| Il1r1 | 328 | 14 | 585.3333 | 2073.53 | 35856 | 0.31868 |
| Il6r | 624 | 22 | 657.75 | 3089.099 | 67368 | 0.25974 |
| Cldn7 | 520 | 24 | 649.2167 | 9010.186 | 118128 | 0.21014 |
| Slc7a11 | 490 | 17 | 627.1333 | 2453.487 | 43980 | 0.27206 |
| Cd3g | 227462 | 27 | 626.05 | 7063.804 | 93440 | 0.28775 |
| Plaur | 1594319 | 55 | 736.6833 | 25321.89 | 485240 | 0.19798 |
| Ptpn18 | 12525 | 23 | 672.7833 | 6880.296 | 115428 | 0.26087 |
| Fzd2 | 7266 | 23 | 647.0667 | 2246.803 | 61222 | 0.38735 |
| Fzd1 | 3145 | 18 | 612.7333 | 3283.929 | 58068 | 0.40523 |
| Ptprz1 | 1929 | 20 | 676.7167 | 3068.216 | 60456 | 0.27895 |
| Por | 9.22E+13 | 39 | 652.5667 | 4207.522 | 107258 | 0.46154 |
| Prkcq | 86081 | 36 | 705.35 | 19350.15 | 269786 | 0.15556 |
| Itgad | 2905 | 30 | 673.1667 | 4014.938 | 83972 | 0.23908 |
| Vdr | 72 | 20 | 688.9167 | 3833.599 | 79566 | 0.13684 |
| Grin2c | 46 | 14 | 607.3667 | 3113.363 | 36134 | 0.16484 |
| Map1b | 88 | 24 | 655.55 | 9267.329 | 123960 | 0.09783 |
| Dab2 | 54 | 20 | 654.7167 | 5751.07 | 98262 | 0.09474 |
| Dab1 | 42 | 14 | 607 | 3842.736 | 71910 | 0.17582 |
| C8g | 54 | 12 | 584.2762 | 2631.534 | 37322 | 0.24242 |
| Tubb6 | 10986 | 19 | 656.0333 | 4588.618 | 87018 | 0.27485 |
| Wnt5a | 9835 | 37 | 689.4167 | 9417.794 | 199314 | 0.22222 |
| Cdx2 | 264 | 17 | 608.15 | 3641.854 | 48338 | 0.16176 |
| Wnt7b | 6532 | 22 | 640.3167 | 3997.087 | 84970 | 0.34199 |
| Col4a5 | 1.54E+12 | 44 | 684.9333 | 3005.549 | 86266 | 0.49366 |
| Smo | 142 | 14 | 646.5333 | 6864.627 | 91986 | 0.1978 |
| Rbpjl | 179 | 27 | 627.8833 | 11095.76 | 164214 | 0.07692 |
| Ppic | 5193 | 15 | 604.2833 | 3032.709 | 44400 | 0.29524 |
| Fbln2 | 7.38E+09 | 29 | 650.9833 | 2219.547 | 58342 | 0.49261 |
| Nid1 | 3.91E+10 | 39 | 672.5333 | 6775.398 | 151448 | 0.45479 |
| Nid2 | 1.11E+10 | 33 | 650.5333 | 3140.728 | 82884 | 0.46591 |
| Tnfrsf4 | 10456 | 15 | 609.9 | 2871.325 | 68070 | 0.42857 |
| Il2rg | 98274 | 29 | 664.3667 | 9036.326 | 139472 | 0.27586 |
| Gstz1 | 138 | 17 | 605.7 | 5463.348 | 61106 | 0.09559 |
| Il21r | 13926 | 30 | 673.85 | 8365.906 | 163858 | 0.24368 |
| Bcl6 | 47391 | 24 | 639.5167 | 7652.471 | 134326 | 0.25725 |
| Mapk13 | 999 | 42 | 715.8333 | 20468.28 | 284192 | 0.10685 |
| Pik3c2b | 252 | 17 | 659.1 | 3215.206 | 61698 | 0.25735 |
| Ccnd2 | 4613951 | 30 | 698.05 | 6811.822 | 174234 | 0.34023 |
| Fat1 | 66 | 18 | 668.1 | 10968.4 | 112052 | 0.13072 |
| Muc1 | 101 | 16 | 616.3667 | 14002.48 | 204230 | 0.18333 |
| Tacc2 | 3628804 | 14 | 598.9167 | 2592.133 | 41226 | 0.50549 |
| Gmnn | 9.22E+13 | 60 | 654.3167 | 5926.141 | 135360 | 0.71864 |
| Kif12 | 9.22E+13 | 26 | 599.65 | 3059.315 | 60744 | 0.64308 |
| Kif3a | 9.22E+13 | 32 | 616.4833 | 10136.5 | 187898 | 0.38306 |
| Kif3c | 6.28E+13 | 24 | 592.5167 | 2389.065 | 65536 | 0.5942 |
| Stil | 9.22E+13 | 48 | 631.9667 | 3816.511 | 104254 | 0.85816 |
| RGD1310335 | 9.22E+13 | 21 | 598.7667 | 3432.277 | 74060 | 0.8 |
| Mcph1 | 1.40E+10 | 28 | 619.9 | 3294.004 | 65034 | 0.44444 |
| Skp2 | 9.22E+13 | 31 | 658.05 | 4673.899 | 113976 | 0.53118 |
| Cdc7 | 9.22E+13 | 48 | 637.1833 | 3859.154 | 111090 | 0.79433 |
| Efna5 | 133 | 15 | 637.55 | 6318.693 | 100036 | 0.13333 |
| Kcnq1 | 43 | 16 | 602.8333 | 7136.73 | 76092 | 0.11667 |
| Rad18 | 2.01E+11 | 26 | 594.9833 | 4349.566 | 74820 | 0.58154 |
| Bard1 | 9.22E+13 | 63 | 696.0167 | 8285.816 | 222236 | 0.58167 |
| Brca2 | 9.22E+13 | 38 | 679.65 | 7206.54 | 173182 | 0.48649 |
| Lum | 3.57E+10 | 42 | 663.75 | 16383.61 | 351760 | 0.33914 |
| Chaf1a | 9.22E+13 | 60 | 663.2833 | 2208.13 | 59840 | 0.77062 |
| Mybl2 | 9.22E+13 | 73 | 664.7333 | 5263.418 | 122984 | 0.74163 |
| Rad54l | 9.22E+13 | 82 | 686.5333 | 3586.612 | 104252 | 0.7305 |
| Insig1 | 956 | 15 | 597.3 | 2708.283 | 53760 | 0.44762 |
| Ska3 | 9.22E+13 | 85 | 673.55 | 2225.244 | 49028 | 0.85378 |
| Meiob | 9.22E+13 | 36 | 626 | 4735.877 | 128580 | 0.62222 |
| Spc24 | 9.22E+13 | 80 | 665.4167 | 2292.089 | 59730 | 0.83671 |
| Ercc6l | 9.22E+13 | 66 | 669.15 | 6255.765 | 129248 | 0.73706 |
| Cdca7 | 9.22E+13 | 54 | 645.0667 | 3428.791 | 74012 | 0.85814 |
| Cblc | 777 | 17 | 641.8667 | 3035.056 | 48142 | 0.32353 |
| Itgbl1 | 4.50E+08 | 52 | 698.3333 | 18187.03 | 323056 | 0.25038 |
| Itgb4 | 3.09E+09 | 70 | 751.2333 | 12639.81 | 367814 | 0.27495 |
| Itgb8 | 1.09E+09 | 53 | 717.8333 | 4725.425 | 168698 | 0.28374 |
| Sfn | 9981 | 31 | 700.8833 | 9452.858 | 178684 | 0.2 |
| Ephb2 | 2.62E+12 | 28 | 684.3833 | 9539.717 | 257360 | 0.34921 |
| Ptk7 | 2119 | 31 | 662.0333 | 10600.21 | 167634 | 0.15914 |
| Zap70 | 450690 | 44 | 693.45 | 12040.7 | 210796 | 0.24524 |
| Blnk | 111 | 14 | 606.85 | 2806.508 | 39994 | 0.21978 |
| Myo1f | 125 | 15 | 601.7833 | 3630.555 | 52552 | 0.22857 |
| Arhgap27 | 1542 | 33 | 643.35 | 6652.778 | 93514 | 0.16856 |
| Espl1 | 9.22E+13 | 108 | 700.8667 | 4028.009 | 85576 | 0.72343 |
| Stmn1 | 9.22E+13 | 40 | 669.1 | 6945.771 | 168392 | 0.44103 |
| Cenpw | 9.22E+13 | 77 | 671.1667 | 5790.405 | 138094 | 0.82638 |
| Mis18bp1 | 9.22E+13 | 84 | 670.9833 | 1929.265 | 44658 | 0.83247 |
| Troap | 9.22E+13 | 74 | 673.7667 | 5036.551 | 106602 | 0.83265 |
| Rad51 | 9.22E+13 | 97 | 689.4167 | 4027.319 | 89650 | 0.7296 |
| Mastl | 9.22E+13 | 47 | 633.7667 | 3908.815 | 107712 | 0.81129 |
| Gins1 | 9.22E+13 | 62 | 646.6833 | 12242.86 | 282390 | 0.77419 |
| Spdl1 | 9.22E+13 | 71 | 653.9667 | 4208.173 | 123602 | 0.87968 |
| Asf1b | 9.22E+13 | 103 | 698.45 | 4845.124 | 119236 | 0.75176 |
| Ube2t | 9.22E+13 | 70 | 664.05 | 4316.362 | 108530 | 0.72505 |
| Fignl1 | 9.22E+13 | 66 | 662.2333 | 9631.588 | 206998 | 0.73566 |
| Arhgap11a | 9.22E+13 | 104 | 713.0667 | 13550.08 | 257720 | 0.64544 |
| Pld2 | 59 | 20 | 672.2 | 8143.655 | 119672 | 0.09474 |
| Cldn4 | 294 | 17 | 613.8667 | 3643.43 | 59312 | 0.25735 |
| Trem2 | 222 | 19 | 630.4333 | 4558.145 | 84140 | 0.25731 |
| Soat1 | 168 | 15 | 597.5167 | 6182.23 | 86322 | 0.2381 |
| Cldn1 | 193 | 17 | 646.1333 | 6778.909 | 81604 | 0.23529 |
| Gpc3 | 1004 | 23 | 664.9167 | 8308.131 | 114870 | 0.21344 |
| Cd2 | 180034 | 22 | 640.4 | 5717.88 | 95606 | 0.41126 |
| Gckr | 104 | 15 | 585.6833 | 5632.035 | 102578 | 0.21905 |
| Car2 | 63 | 13 | 633.3833 | 2069.163 | 29930 | 0.19231 |
| Creb3l3 | 409 | 16 | 620.4333 | 4737.12 | 66822 | 0.225 |
| Lrp8 | 3314 | 42 | 693.1833 | 17300.08 | 256602 | 0.14634 |
| Vldlr | 6.27E+09 | 39 | 699.1 | 10015.26 | 203292 | 0.22942 |
| LOC691984 | 1643 | 24 | 656.0667 | 9655.758 | 127028 | 0.17754 |
| Fzd4 | 4092 | 24 | 648.5833 | 6189.445 | 126318 | 0.27536 |
| Fzd6 | 4551 | 21 | 620.5 | 2808.422 | 61216 | 0.34762 |
| Enah | 179 | 20 | 645.75 | 3827.629 | 53480 | 0.13684 |
| Mst1r | 63276 | 57 | 731.85 | 24401.53 | 419172 | 0.1297 |
| Itk | 238105 | 48 | 715.3167 | 21898.37 | 386410 | 0.18174 |
| Apbb1ip | 62 | 16 | 642.05 | 5030.559 | 70340 | 0.16667 |
| RT1-Da | 91264 | 25 | 622.4167 | 9445.216 | 146548 | 0.24667 |
| Cd74 | 1397 | 24 | 644.3333 | 9229.695 | 149574 | 0.19928 |
| Prnp | 145 | 32 | 675.6167 | 16886.57 | 284446 | 0.08871 |
| Ccne1 | 9.22E+13 | 56 | 711.85 | 6269.806 | 220634 | 0.52987 |
| Il2ra | 555956 | 38 | 673.65 | 5891.277 | 126218 | 0.3229 |
| Gpnmb | 248 | 18 | 656.6833 | 3510.821 | 58392 | 0.21569 |
| Hltf | 5410 | 16 | 587.7 | 2697.702 | 49864 | 0.39167 |
| Tspan3 | 297 | 21 | 660.0333 | 7636.329 | 108650 | 0.15714 |
| Clec7a | 394 | 21 | 617.7833 | 3484.152 | 50132 | 0.19048 |
| Jag2 | 1173 | 19 | 653.8333 | 4216.058 | 78198 | 0.2807 |
| Dusp1 | 770 | 17 | 656.95 | 4209.342 | 70024 | 0.19853 |
| Ms4a2 | 44 | 13 | 589.4833 | 4234.83 | 50932 | 0.16667 |
| Fkbp10 | 458676 | 20 | 607.2167 | 3697.723 | 61784 | 0.37368 |
| Clic1 | 1499 | 16 | 606.7333 | 3891.553 | 41824 | 0.26667 |
| Kcnn4 | 58 | 16 | 610.45 | 7629.791 | 89320 | 0.15 |
| Ano1 | 95 | 13 | 651.35 | 3563.325 | 53080 | 0.26923 |
| Racgap1 | 9.22E+13 | 105 | 709.5833 | 4515.531 | 141284 | 0.70641 |
| Kif23 | 9.22E+13 | 130 | 745.55 | 21036.46 | 399326 | 0.56995 |
| Kif11 | 9.22E+13 | 139 | 735.6 | 11440.86 | 297120 | 0.54614 |
| Kif20a | 9.22E+13 | 121 | 714.6333 | 4148.517 | 130502 | 0.6562 |
| Kif2c | 9.22E+13 | 125 | 717.2667 | 6617.137 | 203346 | 0.62181 |
| Cdc20 | 9.22E+13 | 126 | 720.2 | 4133.465 | 126216 | 0.64317 |
| Mcm5 | 9.22E+13 | 108 | 712.1333 | 11480.55 | 245196 | 0.63413 |
| Cep55 | 9.22E+13 | 112 | 702.25 | 2487.086 | 113260 | 0.72699 |
| Ccna2 | 9.22E+13 | 135 | 765.95 | 20210.91 | 491552 | 0.57092 |
| Cdca8 | 9.22E+13 | 123 | 715.7833 | 3780.609 | 123874 | 0.66307 |
| Kif20b | 9.22E+13 | 107 | 702.4 | 5566.805 | 120708 | 0.68648 |
| Melk | 9.22E+13 | 116 | 716.1 | 6428.18 | 135952 | 0.70645 |
| Bub1 | 9.22E+13 | 129 | 720.8667 | 6789.764 | 179476 | 0.61265 |
| Nusap1 | 9.22E+13 | 111 | 710.9167 | 4633.898 | 115588 | 0.72826 |
| Shcbp1 | 9.22E+13 | 96 | 684.3333 | 4026.939 | 92598 | 0.81996 |
| Pbk | 9.22E+13 | 116 | 713.6167 | 8434.66 | 134262 | 0.6925 |
| Cenpe | 9.22E+13 | 120 | 722.9 | 10462.94 | 233516 | 0.62605 |
| Hjurp | 9.22E+13 | 94 | 696.6167 | 7142.539 | 149596 | 0.78701 |
| Fam83d | 9.22E+13 | 58 | 655.4167 | 2893.971 | 76016 | 0.85965 |
| Ttk | 9.22E+13 | 132 | 739.5667 | 17062.44 | 303466 | 0.57356 |
| Trip13 | 9.22E+13 | 105 | 696.0667 | 8083.184 | 206732 | 0.74359 |
| Cenpi | 9.22E+13 | 82 | 675.3167 | 5342.284 | 96120 | 0.85757 |
| Kifc1 | 9.22E+13 | 96 | 704.5667 | 8574.878 | 185890 | 0.65439 |
| Kntc1 | 9.22E+13 | 106 | 697.8 | 4295.693 | 77670 | 0.76137 |
| Cdca2 | 9.22E+13 | 91 | 682.6667 | 2016.369 | 42418 | 0.8381 |
| Kifc3 | 9.22E+13 | 55 | 660.8667 | 1924.678 | 52938 | 0.69562 |
| Cdca5 | 9.22E+13 | 108 | 699.7167 | 3013.571 | 69460 | 0.77103 |
| Cdc45 | 9.22E+13 | 118 | 716.7833 | 22828.24 | 392554 | 0.61466 |
| Cdc25c | 9.22E+13 | 85 | 729.5333 | 11453.46 | 289752 | 0.7056 |
| Cdca3 | 9.22E+13 | 98 | 690.1 | 3690.529 | 77448 | 0.76183 |
| Cdc6 | 9.22E+13 | 113 | 708.2333 | 5375.289 | 156336 | 0.67999 |
| Ns5atp9 | 9.22E+13 | 101 | 692.4 | 2992.122 | 66642 | 0.7798 |
| Ska1 | 9.22E+13 | 87 | 672.8667 | 3899.192 | 104674 | 0.82732 |
| Kif22 | 9.22E+13 | 94 | 686.9 | 7537.326 | 160560 | 0.72775 |
| Uhrf1 | 9.22E+13 | 105 | 698.7333 | 4951.242 | 88720 | 0.74249 |
| Ndc80 | 9.22E+13 | 124 | 717.15 | 3369.439 | 165698 | 0.64241 |
| Prc1 | 9.22E+13 | 118 | 714.9833 | 6838.029 | 167352 | 0.65189 |
| Mcm3 | 9.22E+13 | 105 | 709.35 | 8169.028 | 173240 | 0.67711 |
| Plk4 | 9.22E+13 | 108 | 702.3167 | 7057.609 | 154048 | 0.65888 |
| Ncaph | 9.22E+13 | 120 | 710.2167 | 11026.93 | 191852 | 0.66331 |
| Spag5 | 9.22E+13 | 105 | 696.9333 | 4171.038 | 101636 | 0.76813 |
| Dlgap5 | 9.22E+13 | 113 | 700.0167 | 9414.289 | 175902 | 0.71318 |
| Wdhd1 | 9.22E+13 | 83 | 686.55 | 4738.731 | 117138 | 0.7655 |
| Cdkn3 | 9.22E+13 | 89 | 703.15 | 7044.104 | 173972 | 0.71144 |
| Depdc1 | 9.22E+13 | 97 | 684.4167 | 6958.259 | 155534 | 0.76439 |
| Zwilch | 9.22E+13 | 86 | 678.5167 | 2718.518 | 53892 | 0.8446 |
| Fam64a | 9.22E+13 | 68 | 659.9167 | 4708.547 | 110586 | 0.88016 |
| Tpx2 | 9.22E+13 | 117 | 709.0833 | 2402.271 | 96420 | 0.68597 |
| Bub1b | 9.22E+13 | 128 | 726.4167 | 12814.54 | 261038 | 0.62537 |
| Aurkb | 9.22E+13 | 141 | 746.5 | 12943.08 | 348906 | 0.53141 |
| Kif18a | 9.22E+13 | 89 | 700.2667 | 3769.196 | 140106 | 0.70276 |
| Nek2 | 9.22E+13 | 113 | 709.65 | 12991.93 | 247928 | 0.63322 |
| Dtl | 9.22E+13 | 96 | 694.6 | 5823.138 | 115468 | 0.73969 |
| Pttg1 | 9.22E+13 | 94 | 700.8 | 5515.73 | 128096 | 0.76458 |
| Mcm6 | 9.22E+13 | 94 | 701.2167 | 7895.374 | 188768 | 0.70007 |
| Nuf2 | 9.22E+13 | 112 | 702 | 3832.748 | 112414 | 0.71252 |
| Anln | 9.22E+13 | 83 | 689.8167 | 6345.778 | 128028 | 0.80752 |
| Rad51ap1 | 9.22E+13 | 97 | 689.3 | 3633.39 | 67262 | 0.79596 |
| Fhl2 | 253 | 23 | 664.1167 | 6980.793 | 96676 | 0.16601 |
| Figf | 9.58E+08 | 23 | 658.3333 | 3521.214 | 72788 | 0.50198 |
| Grb10 | 46355 | 21 | 664.7333 | 5712.384 | 103856 | 0.27143 |
| Itga8 | 6.00E+10 | 63 | 718.4 | 12100.85 | 244704 | 0.33282 |
| Itga3 | 2.99E+10 | 61 | 739.7833 | 7338.287 | 229342 | 0.37104 |
| Notch3 | 14326 | 27 | 674.4667 | 5554.779 | 128902 | 0.29345 |
| Igfals | 102 | 20 | 638.9667 | 6155.055 | 80494 | 0.14737 |
| Fgl1 | 15915 | 22 | 622.9 | 3331.289 | 55134 | 0.28571 |
| Alox5 | 2.09E+13 | 23 | 604.2429 | 2100.719 | 50666 | 0.62055 |
| Fermt1 | 129 | 18 | 613.5262 | 6558.576 | 92136 | 0.14379 |
| Itga6 | 3.85E+10 | 71 | 743.6667 | 8781.919 | 263818 | 0.32435 |
| Fmo3 | 52374 | 19 | 596.7762 | 2075.118 | 29750 | 0.36257 |
| Pxdn | 127 | 12 | 601 | 2341.554 | 35430 | 0.18182 |
| Psat1 | 90 | 22 | 621.2833 | 9613.636 | 106144 | 0.11688 |
| Chaf1b | 9.22E+13 | 54 | 652.3667 | 6126.715 | 133104 | 0.66737 |
| Slc10a1 | 12830 | 23 | 659.85 | 2075.887 | 60950 | 0.41502 |
| Anxa5 | 1.34E+09 | 61 | 758.5667 | 18217.84 | 519160 | 0.2459 |
| Cd44 | 6.30E+12 | 135 | 846.4333 | 72321.45 | 1495892 | 0.17148 |
| Erbb2 | 4.63E+09 | 95 | 811.6167 | 37032.76 | 931670 | 0.16327 |
| Map2 | 901 | 26 | 703.0167 | 11947.57 | 193024 | 0.20308 |
| Gpx1 | 9.96E+10 | 40 | 692.9167 | 4314.515 | 160340 | 0.36795 |
| Prom1 | 2.21E+11 | 40 | 736.7667 | 3699.243 | 107156 | 0.46282 |
| Krt7 | 8716478 | 30 | 704.3667 | 6098.211 | 125436 | 0.32184 |
| Ncam1 | 2.72E+08 | 56 | 754.4 | 24729.03 | 402988 | 0.21688 |
| Cd28 | 1.30E+07 | 53 | 715.85 | 13907.02 | 303200 | 0.25689 |
| Kitlg | 6.05E+11 | 43 | 735.8833 | 14988.2 | 269330 | 0.33444 |
| Aqp4 | 319 | 15 | 653.3 | 5465.295 | 71596 | 0.29524 |
| Bmp4 | 1.56E+10 | 66 | 748.1333 | 35646.22 | 616240 | 0.21538 |
| Lgals3 | 5.24E+08 | 45 | 729.5333 | 17000.18 | 312256 | 0.23333 |
| LOC259244 | 92 | 13 | 629.5833 | 1991.056 | 36870 | 0.32051 |
| Krt8 | 1503592 | 26 | 699.0833 | 4159.613 | 96120 | 0.32923 |
| Igf2 | 1.60E+07 | 40 | 717.4167 | 15076.12 | 254896 | 0.27692 |
| Cd69 | 6063868 | 34 | 682.6167 | 2321.03 | 70948 | 0.40642 |
| Slc1a2 | 549 | 28 | 660.2167 | 12993.91 | 174992 | 0.14286 |
| Mup4 | 89 | 14 | 630.4833 | 3773.138 | 63824 | 0.25275 |
| Cd9 | 8.48E+07 | 38 | 704.3167 | 17330.89 | 299794 | 0.25462 |
| Ccnb1 | 9.22E+13 | 140 | 802.3833 | 42375.76 | 1237262 | 0.53155 |
| Cx3cr1 | 1.62E+07 | 32 | 671.0333 | 3875.33 | 101278 | 0.38105 |
| Lmnb1 | 9.22E+13 | 55 | 715.0167 | 12623.76 | 410960 | 0.74949 |
| Ntf3 | 80678 | 18 | 680.4667 | 6371.799 | 101588 | 0.29412 |
| Cdkn2a | 1.29E+10 | 41 | 735.5833 | 9874.135 | 312362 | 0.32927 |
| Slc5a1 | 234 | 25 | 681.8167 | 16089.87 | 223254 | 0.15 |
| Jag1 | 1.66E+10 | 42 | 725.1167 | 10920.4 | 248644 | 0.33566 |
| Ggt1 | 12255 | 15 | 639.15 | 2368.736 | 41092 | 0.4 |
| Slc1a5 | 574 | 27 | 683.55 | 13541.82 | 206710 | 0.18519 |
| Itga2 | 2.39E+10 | 67 | 745.85 | 16034.18 | 380724 | 0.29127 |
| Cd3e | 1957332 | 39 | 690.7167 | 13750.46 | 250398 | 0.29285 |
| Nefl | 352 | 26 | 691.8833 | 11964.55 | 156252 | 0.12923 |
| Aqp5 | 38 | 15 | 635.6333 | 3747.949 | 56066 | 0.14286 |
| Slc22a8 | 684 | 22 | 649.9 | 3238.448 | 78158 | 0.28571 |
| Pf4 | 1.84E+08 | 35 | 679.6167 | 7056.477 | 149330 | 0.39496 |
| Fut4 | 21037 | 18 | 660.5833 | 10294.54 | 175952 | 0.34641 |
| Dpysl2 | 145 | 22 | 667.2833 | 9004.208 | 121536 | 0.16017 |
| Cd276 | 247083 | 22 | 661.8333 | 4667.586 | 77210 | 0.45887 |
| Serpinb5 | 22770 | 22 | 671.9333 | 4598.081 | 82742 | 0.30303 |
| Serpinb8 | 20911 | 18 | 660.85 | 2938.173 | 55042 | 0.31373 |
| Uchl1 | 670 | 17 | 679.6833 | 1891.091 | 34948 | 0.27941 |
| Trpv4 | 196 | 20 | 652.3167 | 5570.681 | 93810 | 0.22105 |
| Apom | 7.79E+09 | 29 | 671.2833 | 3188.27 | 60168 | 0.48522 |
| Cdk1 | 9.22E+13 | 145 | 803.9167 | 40988.8 | 1102438 | 0.50105 |
| Alox15 | 9.22E+13 | 32 | 636.9595 | 5754.377 | 116634 | 0.46774 |
| Pla2g2a | 9.22E+13 | 33 | 678.8 | 15777.2 | 251070 | 0.35795 |
| Cyp1b1 | 9127571 | 34 | 644.1833 | 4298.58 | 115580 | 0.35829 |
| Cyp2c6v1 | 9.22E+13 | 48 | 669.85 | 3210.29 | 147780 | 0.52216 |
| Cyp2d3 | 1531086 | 31 | 667.7667 | 2224.279 | 113530 | 0.4086 |
| Gpld1 | 6.23E+09 | 29 | 683.7333 | 6569.635 | 126854 | 0.30049 |
| Cyp2c11 | 9.22E+13 | 53 | 678.8167 | 4365.937 | 166960 | 0.44993 |
| Cyp3a62 | 9.22E+13 | 54 | 684.1833 | 5865.784 | 242346 | 0.42697 |
| RGD1559459 | 9.22E+13 | 52 | 672.0833 | 4499.381 | 167110 | 0.39065 |
| Cyp1a1 | 9.22E+13 | 56 | 687.3333 | 5056.446 | 257418 | 0.44026 |
| Ugt2b10 | 9.22E+13 | 50 | 670.6667 | 4255.011 | 160068 | 0.40735 |
| Txnrd1 | 368278 | 28 | 647.9333 | 4977.898 | 89504 | 0.22222 |
| Gck | 789 | 25 | 675.8167 | 4935.19 | 97522 | 0.23333 |
| Akr7a3 | 494 | 21 | 587.1833 | 2476.27 | 41468 | 0.24762 |
| Cyp2e1 | 9.22E+13 | 79 | 716.5667 | 18901.5 | 518344 | 0.26355 |
| G6pc | 1454 | 28 | 677.95 | 4459.172 | 94170 | 0.24868 |
| Gclc | 1194932 | 43 | 686.0833 | 15364.73 | 274984 | 0.19601 |
| G6pd | 1902381 | 32 | 671.7 | 3140.883 | 69738 | 0.29032 |
| Gstm3 | 3.31E+11 | 45 | 695.9 | 4740.063 | 173234 | 0.4 |
| Hk2 | 259 | 19 | 591.7333 | 2216.591 | 28470 | 0.23392 |
| Arhgef16 | 43 | 12 | 614.8 | 2677.185 | 32954 | 0.16667 |
| Arhgef28 | 2946 | 31 | 682.6667 | 5925.283 | 100732 | 0.20645 |
| Ect2 | 9.22E+13 | 111 | 716.75 | 11295.47 | 236244 | 0.65094 |
| Rnd1 | 589 | 45 | 700 | 18521.66 | 314404 | 0.11111 |
| Rhoj | 5775 | 44 | 711.35 | 12746.6 | 243004 | 0.17336 |
| Akap13 | 324 | 16 | 644.8333 | 2345.771 | 34874 | 0.25 |
| Rrm2 | 9.22E+13 | 109 | 711.3833 | 10135.27 | 223292 | 0.68315 |
| Ampd3 | 43 | 12 | 640.9833 | 3485.295 | 51294 | 0.21212 |
| LOC100359539 | 9.22E+13 | 102 | 699.9333 | 4317.5 | 108402 | 0.7251 |
| Ak4 | 151 | 13 | 587.8 | 6373.767 | 81160 | 0.20513 |
| F13a1 | 55 | 12 | 606.9452 | 3656.359 | 55798 | 0.16667 |
| Alas2 | 256 | 16 | 596.6762 | 8147.288 | 93128 | 0.16667 |
| Anxa2 | 91258 | 41 | 713.6167 | 8817.144 | 189456 | 0.24146 |
| S100a10 | 4512 | 25 | 645.3667 | 5121.47 | 84832 | 0.26667 |
| Fosl1 | 157 | 13 | 645.4 | 4328.028 | 60372 | 0.21795 |
| Anxa1 | 15237 | 31 | 697.4 | 10680.94 | 185506 | 0.2172 |
| Nqo1 | 3681217 | 39 | 698.0167 | 12416.39 | 296320 | 0.30904 |
| S100a6 | 4772 | 25 | 637.9833 | 5279.282 | 114766 | 0.28667 |
| Cenpf | 9.22E+13 | 115 | 722.8667 | 9940.322 | 229636 | 0.67811 |
| S100b | 1045 | 22 | 681.2 | 3886.119 | 62786 | 0.28139 |
| Ahnak | 185 | 21 | 652.4167 | 4491.93 | 60110 | 0.13333 |
| Mthfd2 | 43 | 15 | 595.6833 | 2051.625 | 26128 | 0.17143 |
| Kif1a | 2.09E+13 | 27 | 647.9167 | 8540.472 | 133688 | 0.40171 |
| Phgdh | 102 | 19 | 639.1333 | 3273.449 | 56766 | 0.15789 |
| Mbl2 | 4.58E+11 | 42 | 669.35 | 3923.316 | 82752 | 0.4518 |
| Apof | 7.71E+09 | 34 | 657.1333 | 12722.97 | 174616 | 0.37611 |
| Rnase4 | 206649 | 16 | 606.25 | 3705.631 | 45002 | 0.48333 |
| Cyp3a1 | 9.22E+13 | 73 | 710.8 | 10950.95 | 422638 | 0.32686 |
| Nags | 334 | 23 | 636.7833 | 8404.881 | 121394 | 0.14625 |
| Plk1 | 9.22E+13 | 139 | 792.2 | 40366.03 | 826166 | 0.52727 |
| Ctbp2 | 55 | 20 | 646.2 | 14432.35 | 205892 | 0.08947 |
| Tat | 3.95E+10 | 56 | 709.4 | 8926.235 | 181896 | 0.27403 |
| Gulo | 177 | 17 | 644.4667 | 3441.84 | 61004 | 0.18382 |
| Got1 | 5478 | 35 | 674.9167 | 6104.794 | 124494 | 0.21345 |
| Cyp1a2 | 9.22E+13 | 74 | 718.5333 | 11294.97 | 355912 | 0.311 |
| Tdo2 | 14103 | 29 | 675.35 | 3757.885 | 74630 | 0.29064 |
| Mbl1 | 9.57E+11 | 30 | 676.5 | 3648.985 | 72380 | 0.54483 |
| Serpina3m | 2.49E+07 | 31 | 648.5333 | 2691.464 | 49388 | 0.52688 |
| C8b | 3.81E+11 | 50 | 688.35 | 10932.31 | 183444 | 0.33224 |
| Slc38a4 | 132 | 16 | 616.8167 | 4602.658 | 50234 | 0.225 |
| LOC297568 | 3.05E+07 | 31 | 672.2667 | 2247.226 | 47232 | 0.45161 |
| Sds | 616 | 22 | 600.7 | 1937.462 | 26218 | 0.30736 |
| Ncapg | 9.22E+13 | 127 | 718.3333 | 5938.116 | 138100 | 0.63242 |
| P2ry2 | 278 | 15 | 613.6167 | 3740.398 | 56970 | 0.21905 |
| Habp2 | 9.13E+11 | 50 | 716.75 | 18455.1 | 319176 | 0.30122 |
| Lpar1 | 228 | 20 | 687.0667 | 5719.5 | 92198 | 0.16842 |
| F2r | 2236 | 24 | 659.2333 | 10204.87 | 142180 | 0.21739 |
| Thbs1 | 1.53E+13 | 71 | 748.4167 | 8304.762 | 264924 | 0.37143 |
| Fgf21 | 2.45E+13 | 62 | 752.55 | 11678.82 | 295458 | 0.38075 |
| Egr1 | 6.23E+09 | 23 | 688.4667 | 2513.369 | 53424 | 0.47826 |
| Igf1r | 8.42E+09 | 71 | 776.8667 | 23974.78 | 456010 | 0.19839 |
| Cxcr2 | 1.15E+07 | 28 | 683.7833 | 3052.724 | 77208 | 0.4127 |
| Ptger4 | 41105 | 20 | 666.5 | 7177.556 | 121976 | 0.25789 |
| Cpa3 | 64 | 19 | 614.5333 | 5315.966 | 70092 | 0.1345 |
| Cpb2 | 9.72E+11 | 75 | 745.6167 | 28252.4 | 472092 | 0.24865 |
| Cftr | 426 | 46 | 713.95 | 36139.42 | 466074 | 0.0686 |
| Shc2 | 81838 | 39 | 702.4333 | 6097.531 | 138484 | 0.25506 |
| Cxcr4 | 8.67E+12 | 77 | 763.1333 | 16799.96 | 405004 | 0.28537 |
| Tff3 | 176 | 18 | 668.3333 | 10754.57 | 150748 | 0.16993 |
| Tff1 | 45 | 13 | 632.9 | 4493.806 | 67092 | 0.17949 |
| Pemt | 293 | 19 | 585.0762 | 3514.259 | 42158 | 0.20468 |
| Cyp2j10 | 9.22E+13 | 28 | 596.9262 | 1918.473 | 45310 | 0.69577 |
| Tm7sf2 | 93701 | 24 | 595.3667 | 2977.942 | 89606 | 0.35507 |
| Hsd3b5 | 9.22E+13 | 45 | 617.85 | 5369.4 | 83232 | 0.35152 |
| Cyp26a1 | 9.22E+13 | 42 | 651.3167 | 7265.448 | 151092 | 0.36353 |
| Cyp51 | 1428 | 25 | 597.2667 | 2090.075 | 35210 | 0.23 |
| Dhcr24 | 546 | 18 | 596.2833 | 2891.597 | 38770 | 0.2549 |
| Kynu | 1.15E+09 | 31 | 641.0667 | 4623.259 | 77610 | 0.36989 |
| Gls2 | 503 | 27 | 640.5333 | 7094.773 | 88658 | 0.19943 |
| Cth | 1678 | 31 | 620.5667 | 5567.732 | 74242 | 0.19355 |
| Espn | 132 | 14 | 631.4667 | 3873.794 | 47140 | 0.15385 |
| Pm20d1 | 44 | 13 | 600.7 | 3326.578 | 31540 | 0.15385 |
| Hal | 797 | 24 | 641.4333 | 4752.386 | 71742 | 0.19203 |
| Asl | 576 | 24 | 606.3333 | 3191.008 | 46438 | 0.24638 |
| Agmat | 325 | 30 | 629.1 | 9471.275 | 102216 | 0.13103 |
| Apoc3 | 7.93E+09 | 60 | 723.85 | 12212.12 | 265456 | 0.27119 |
| Spp2 | 9.69E+11 | 44 | 714.4667 | 12554.68 | 223020 | 0.37738 |
| Rbp4 | 8472319 | 35 | 682.95 | 9532.338 | 227964 | 0.24034 |
| Slco1b2 | 20244 | 31 | 687.8833 | 7870.113 | 178910 | 0.27742 |
| Serpina4 | 6.54E+09 | 47 | 691.9333 | 6698.786 | 123292 | 0.40703 |
| Serpina6 | 5.75E+07 | 26 | 658.8833 | 3359.842 | 54056 | 0.48 |
| Pon1 | 7.83E+09 | 40 | 682.1 | 4834.886 | 120106 | 0.34487 |
| Dpys | 39 | 20 | 608.05 | 7848.582 | 91438 | 0.08421 |
| Spint1 | 97 | 21 | 633.8833 | 5587.176 | 83792 | 0.11905 |
| Col6a2 | 1.44E+12 | 53 | 683.4333 | 5523.365 | 136086 | 0.39913 |
| RGD1564614 | 1.60E+07 | 26 | 677.3333 | 5927.405 | 99528 | 0.37846 |
| Cfhr1 | 3.27E+07 | 22 | 623.6833 | 4758.715 | 59208 | 0.54545 |
| Socs1 | 495 | 23 | 662.25 | 4502.29 | 69812 | 0.2253 |
| Aldh18a1 | 466 | 36 | 659.2167 | 12642.02 | 159048 | 0.13016 |
| LOC688286 | 75 | 16 | 590.55 | 2329.429 | 25534 | 0.19167 |
| Mybl1 | 80 | 13 | 603.7 | 3111.056 | 45686 | 0.17949 |
| Gstp1 | 1.56E+11 | 46 | 707.5667 | 10642.22 | 274816 | 0.34203 |
| Mat1a | 606 | 23 | 628.2 | 2728.111 | 44120 | 0.24111 |
| Oat | 891 | 27 | 627.2 | 3991.141 | 48328 | 0.23647 |
| Grhpr | 363 | 29 | 631.15 | 5733.536 | 76078 | 0.16256 |
| Prodh2 | 99 | 14 | 616.9333 | 2882.013 | 36994 | 0.25275 |
| Cbs | 3052 | 37 | 681.4667 | 11706.21 | 185610 | 0.14865 |
| Akr1cl | 6.28E+13 | 37 | 609.6667 | 3558.957 | 66492 | 0.38589 |
| Gclm | 733876 | 30 | 631.85 | 2874.755 | 75506 | 0.29195 |
| Rgs2 | 39 | 14 | 612.8833 | 3334.156 | 37866 | 0.13187 |
| Adcy2 | 549 | 21 | 615.4 | 7179.044 | 73332 | 0.2 |
| Gng13 | 182 | 21 | 632.6833 | 4351.499 | 64706 | 0.15238 |
| Gng8 | 180 | 22 | 657.4333 | 10244.02 | 127104 | 0.12987 |
| Camk2d | 50 | 25 | 660.4333 | 12065.19 | 163452 | 0.07 |
| Pkm | 571 | 33 | 707.5167 | 16909.82 | 223556 | 0.13068 |
| Nme3 | 426 | 22 | 648.8333 | 10801.05 | 167568 | 0.13853 |
| Dync2h1 | 535 | 21 | 604.7 | 11962.45 | 147788 | 0.22381 |
| Il1a | 7.27E+11 | 46 | 712.15 | 10562.77 | 246358 | 0.38744 |
| Adcy1 | 304 | 17 | 603.05 | 4057.209 | 40204 | 0.24265 |
| Atad5 | 9.22E+13 | 33 | 600.7667 | 2573.472 | 48342 | 0.61174 |
| Col15a1 | 1.51E+12 | 41 | 658.25 | 4102.225 | 106176 | 0.48902 |
| Sox9 | 1.39E+08 | 54 | 742.1333 | 18651.5 | 349076 | 0.24319 |
| Bgn | 7.43E+10 | 55 | 718.7333 | 12688.63 | 279342 | 0.34545 |
| Fbln1 | 4.21E+10 | 31 | 661.1333 | 2014.991 | 57102 | 0.54194 |
| Has2 | 2216 | 19 | 673.25 | 3244.551 | 55144 | 0.24561 |
| Etnppl | 66 | 18 | 617.75 | 7577.85 | 86984 | 0.11111 |
| Col16a1 | 1.49E+12 | 27 | 650.2333 | 2457.959 | 47530 | 0.63818 |
| Col4a2 | 1.58E+12 | 63 | 706.9833 | 7404.448 | 186990 | 0.36559 |
| Col4a1 | 1.60E+12 | 67 | 719.8833 | 10846.02 | 265868 | 0.37042 |
| Plod2 | 1.48E+12 | 26 | 608.4833 | 3776.025 | 60102 | 0.51692 |
| Pycr1 | 74 | 15 | 603.5833 | 3654.001 | 39430 | 0.1619 |
| Efemp2 | 1704257 | 24 | 628.1167 | 3752.024 | 63388 | 0.38406 |
| Loxl1 | 5766487 | 25 | 619.2667 | 3113.694 | 52940 | 0.45667 |
| Col11a1 | 1.52E+12 | 51 | 701.1167 | 12300.16 | 226688 | 0.37176 |
| Fbln5 | 5.13E+07 | 22 | 632.6 | 3461.04 | 73650 | 0.51948 |
| Col5a2 | 1.53E+12 | 62 | 697.2333 | 10935.2 | 273190 | 0.32681 |
| Pcolce | 8.77E+10 | 27 | 627.35 | 2260.968 | 37892 | 0.48718 |
| Thbs2 | 6.26E+09 | 59 | 708.4667 | 17354.76 | 361392 | 0.2782 |
| Adamts2 | 9.52E+07 | 32 | 646.0833 | 4794.5 | 85838 | 0.35282 |
| Spon1 | 5065 | 17 | 621.7333 | 3245.813 | 44340 | 0.21324 |
| Sema5a | 5053 | 17 | 612.1 | 4521.973 | 57204 | 0.18382 |
| Vcan | 6.08E+10 | 47 | 718 | 17014.56 | 291638 | 0.3802 |
| Mad2l1 | 9.22E+13 | 130 | 723.4833 | 10839.25 | 226736 | 0.58068 |
| Igfbp5 | 160 | 14 | 624.6333 | 2779.41 | 43190 | 0.26374 |
| Sdc4 | 2.28E+08 | 63 | 742.8667 | 16387.69 | 355786 | 0.23093 |
| Prkar2b | 74 | 24 | 694.2833 | 16281.14 | 226738 | 0.09783 |
| Adam12 | 2210 | 18 | 633.6667 | 6642.731 | 104934 | 0.29412 |
| Gas7 | 449 | 31 | 663.7 | 12431.2 | 188314 | 0.13333 |
| E2f8 | 9.22E+13 | 59 | 663.85 | 3748.285 | 80600 | 0.7744 |
| Ube2c | 9.22E+13 | 119 | 723.2167 | 16508.31 | 248058 | 0.64834 |
| Postn | 8.70E+10 | 67 | 728.6833 | 7000.654 | 222208 | 0.33288 |
| Krt19 | 5.85E+07 | 44 | 749.8167 | 19814.9 | 348526 | 0.24841 |
| Pdgfb | 2.12E+12 | 56 | 740.25 | 11825.48 | 240646 | 0.30584 |
| Cdh11 | 8225 | 20 | 680.7667 | 2506.173 | 53542 | 0.31053 |
| Top2a | 9.22E+13 | 133 | 756.4 | 16806.57 | 421472 | 0.57724 |
| Mki67 | 9.22E+13 | 117 | 797.5167 | 42512.19 | 1294200 | 0.5837 |
| Tubb3 | 84194 | 29 | 705.7667 | 6538.758 | 171476 | 0.2931 |
| Pdpn | 23494 | 25 | 683.4833 | 3072.204 | 59110 | 0.28333 |
| S100a4 | 969231 | 31 | 686.1333 | 4304.863 | 98160 | 0.35914 |
| Wt1 | 3484 | 24 | 695.6 | 9367.31 | 152248 | 0.25 |
| Itgb6 | 2.03E+10 | 84 | 760.5333 | 19438.13 | 546778 | 0.23982 |
| Mcam | 1.25E+10 | 28 | 698.8333 | 2298.968 | 52748 | 0.46032 |
| Thy1 | 8.62E+11 | 70 | 757.3333 | 12907.04 | 289996 | 0.294 |
| Dcn | 3.84E+10 | 70 | 749.55 | 24992.8 | 518804 | 0.24306 |
| Tnnt2 | 13027 | 24 | 683.0667 | 3757.811 | 76450 | 0.28261 |
| Ezr | 51338 | 61 | 749.6333 | 28853.25 | 528072 | 0.15902 |
| Flna | 106920 | 55 | 759.6167 | 20831 | 388914 | 0.15421 |
| Pak1 | 268959 | 39 | 727.1667 | 8396.79 | 206854 | 0.24291 |
| Actg2 | 327168 | 54 | 732.35 | 21025.24 | 351520 | 0.19287 |
| Flnc | 93758 | 45 | 685.6 | 20050.13 | 330762 | 0.16061 |
| Myh10 | 43525 | 35 | 670.7667 | 9748.911 | 169708 | 0.22857 |
| Lamc2 | 7.09E+09 | 42 | 703.55 | 4354.031 | 117072 | 0.42509 |
| Smarcd3 | 104 | 19 | 638.9333 | 7541.273 | 115784 | 0.18129 |
| Cdh3 | 350808 | 59 | 751.7667 | 23697.16 | 435406 | 0.14845 |
| Ptk2b | 1.00E+09 | 92 | 771.2167 | 57877.66 | 1052744 | 0.14955 |
| Tgfb1i1 | 1.00E+09 | 50 | 709.1667 | 9237.106 | 197160 | 0.27265 |
| Daam2 | 23616 | 28 | 650.8333 | 5001.27 | 84716 | 0.29365 |
| Rac3 | 793527 | 72 | 758.4 | 37981.85 | 779420 | 0.15532 |
| Iqgap3 | 9.22E+13 | 52 | 680.4167 | 8212.138 | 227350 | 0.52262 |
| Vim | 326864 | 45 | 750.9833 | 14207.23 | 298662 | 0.23737 |
| Anxa13 | 306 | 17 | 681.7333 | 2643.784 | 63392 | 0.22059 |
| Esr1 | 2.09E+11 | 94 | 812.1333 | 73617.99 | 1729382 | 0.13887 |
| Fbn1 | 7.58E+10 | 61 | 703.45 | 8871.513 | 209436 | 0.32787 |
| Pik3cg | 7546595 | 66 | 765.1833 | 28905.88 | 573674 | 0.17389 |
| Pfn2 | 2625 | 16 | 632.4333 | 2880.07 | 39086 | 0.39167 |
| Pdgfrb | 5.49E+11 | 71 | 762.75 | 8884.279 | 275250 | 0.31871 |
| Spta1 | 11381 | 25 | 657.4333 | 7918.571 | 123140 | 0.31 |
| Fmnl3 | 164 | 12 | 605.5 | 2756.1 | 39598 | 0.31818 |
| Tubb4a | 4763102 | 26 | 670.1667 | 7160.608 | 152462 | 0.29846 |
| Tagln | 85068 | 39 | 694.4833 | 8448.174 | 159914 | 0.20108 |
| Tpm2 | 25647 | 28 | 656.4 | 6456.268 | 94146 | 0.24339 |
| Epcam | 1.60E+08 | 52 | 751.4 | 15430.75 | 289652 | 0.2632 |
| Dbn1 | 309 | 22 | 664.5667 | 7573.944 | 107710 | 0.11688 |
| Iqgap1 | 32859 | 34 | 710.4167 | 5944.62 | 137748 | 0.26916 |
| Egflam | 1.00E+09 | 49 | 689.6 | 18779.42 | 333468 | 0.23724 |
| Col1a2 | 1.60E+12 | 85 | 729.0833 | 12518.71 | 388686 | 0.28852 |
| Fst | 1778 | 19 | 636.2333 | 4179.72 | 86474 | 0.26901 |
| Lcp1 | 11303 | 23 | 631.6 | 7759.467 | 104490 | 0.2332 |
| Diaph3 | 9.22E+13 | 65 | 686.5 | 5592 | 181054 | 0.59087 |
| Acta2 | 3.25E+07 | 86 | 797.0333 | 39021.46 | 896792 | 0.17401 |
| Tagln2 | 1829 | 19 | 632.95 | 3829.164 | 67694 | 0.31579 |
| Lama5 | 1.62E+10 | 40 | 681.9667 | 2480.607 | 72296 | 0.48205 |
| Lamb1 | 3.12E+10 | 47 | 707.45 | 8069.037 | 184666 | 0.41998 |
| Tspan8 | 438 | 17 | 652.3667 | 14470.25 | 205802 | 0.22059 |
| Myo1a | 412 | 16 | 589.6167 | 5761.648 | 97782 | 0.25 |
| Col1a1 | 1.63E+12 | 112 | 782.0167 | 27518.95 | 746618 | 0.22844 |
| Col3a1 | 1.61E+12 | 84 | 731.0333 | 18163.71 | 447846 | 0.27768 |
| Arhgef6 | 594940 | 36 | 689.6833 | 12705.92 | 200076 | 0.24921 |
| Tubb5 | 1.31E+12 | 32 | 668.9167 | 10773.31 | 219648 | 0.3629 |
| Acta1 | 349108 | 66 | 772.05 | 31423.4 | 650226 | 0.16503 |
| Aldh3b1 | 1545 | 20 | 621.7833 | 2288.459 | 37690 | 0.22632 |
| Aldh1a2 | 9.22E+13 | 41 | 660.1667 | 11716.97 | 193828 | 0.30366 |
| Apoa5 | 7.86E+09 | 50 | 702.5333 | 6168.806 | 147200 | 0.33633 |
| Aldh1a3 | 9.22E+13 | 39 | 669.7667 | 6759.832 | 163246 | 0.35223 |
| Cst7 | 118 | 20 | 626.6833 | 11659.71 | 163704 | 0.14211 |
| Aldh3a1 | 154121 | 42 | 711.2333 | 18654.74 | 405250 | 0.18699 |
| Haao | 1.37E+10 | 31 | 646.2167 | 2087.978 | 39164 | 0.48817 |
| Dhtkd1 | 76 | 20 | 634.9333 | 5395.544 | 70252 | 0.10526 |
| Ttc36 | 1093807 | 19 | 610.1167 | 4475.117 | 64334 | 0.36842 |
| Sult1c3 | 8.12E+07 | 32 | 630.1333 | 3491.877 | 67248 | 0.35484 |
| Kmo | 2.67E+08 | 51 | 669.15 | 9584.569 | 192348 | 0.28314 |
| Prodh | 169 | 19 | 602.6 | 2393.376 | 26692 | 0.20468 |
| Slc51a | 2.33E+07 | 39 | 657 | 15120.23 | 211674 | 0.24696 |
| Ftcd | 9.28E+11 | 53 | 683.6667 | 9994.091 | 160880 | 0.30479 |
| Pnpla3 | 539 | 16 | 585.9667 | 3835.006 | 70524 | 0.275 |
| Lipc | 6.71E+09 | 31 | 650.85 | 3398.765 | 64232 | 0.37204 |
| Cyp2c7 | 9.22E+13 | 51 | 646.2 | 2600.379 | 85140 | 0.44235 |
| Pdlim3 | 26656 | 37 | 675.65 | 12813.92 | 252892 | 0.23123 |
| Pdlim4 | 6348 | 25 | 651.0167 | 4645.603 | 89938 | 0.30667 |
| Cyp26b1 | 9.22E+13 | 34 | 633.5 | 3700.63 | 93752 | 0.45455 |
| Gpd1 | 108 | 16 | 596.3 | 3152.558 | 36746 | 0.20833 |
| Apoc2 | 1.05E+09 | 39 | 696.95 | 5960.839 | 128334 | 0.31039 |
| Incenp | 9.22E+13 | 91 | 707.9167 | 14429.09 | 297274 | 0.72747 |
| Cyp2d2 | 117615 | 24 | 605.1833 | 1939.291 | 40478 | 0.36232 |
| Acss1 | 155 | 17 | 617.2667 | 1962.052 | 26054 | 0.25 |
| Lpin1 | 3532 | 26 | 613.8833 | 5813.308 | 77816 | 0.24615 |
| Cyp8b1 | 27056 | 40 | 642.8667 | 3531.157 | 79052 | 0.24744 |
| Me2 | 2409 | 23 | 629.65 | 4688.593 | 66896 | 0.21739 |
| Cyp7a1 | 9.22E+13 | 73 | 720.35 | 20421.05 | 470692 | 0.22527 |
| Nr0b2 | 864013 | 40 | 687.1333 | 8009.227 | 136864 | 0.25256 |
| Rxrg | 1735 | 23 | 683.3833 | 9124.712 | 162784 | 0.18182 |
| Cyp3a18 | 9.22E+13 | 60 | 653.4 | 4891.214 | 120820 | 0.36328 |
| Pfkp | 71 | 21 | 626 | 5891.631 | 66104 | 0.10952 |
| Hao2 | 9.77E+07 | 47 | 667.2333 | 8958.268 | 143794 | 0.26179 |
| Amacr | 7.99E+07 | 33 | 645.0167 | 9526.367 | 133048 | 0.2803 |
| Hacl1 | 7.99E+07 | 31 | 625.6 | 2030.353 | 37508 | 0.31613 |
| Agxt | 3.13E+09 | 94 | 756.85 | 56819.03 | 767794 | 0.15854 |
| Acsm5 | 1094779 | 26 | 626.0333 | 2217.449 | 42132 | 0.32 |
| Acsm3 | 1100656 | 34 | 640.6 | 6014.078 | 97268 | 0.24242 |
| Fads2 | 4929 | 30 | 628.7333 | 9412.18 | 127690 | 0.24368 |
| Cyp4b1 | 9.22E+13 | 60 | 649.05 | 12567.9 | 253582 | 0.29831 |
| Hao1 | 7.99E+07 | 38 | 690.7167 | 7121.722 | 134010 | 0.28307 |
| Slco1a2 | 10796 | 21 | 648.2 | 6074.922 | 93872 | 0.32381 |
| Slco1a1 | 174738 | 26 | 591.8762 | 3544.106 | 62774 | 0.31385 |
| Myo5c | 15139 | 21 | 625 | 10621.34 | 157944 | 0.19048 |
| Cyp2c13 | 9.22E+13 | 49 | 628.6429 | 2228.827 | 71476 | 0.49065 |
| Uox | 369 | 24 | 665.3833 | 7319.634 | 119550 | 0.18478 |
| Baat | 3.99E+07 | 32 | 666.1667 | 8773.211 | 128802 | 0.27218 |
| Cxcl12 | 2.39E+13 | 81 | 770.4 | 14147.22 | 359330 | 0.29444 |
| Cd40 | 5.68E+07 | 60 | 718.6333 | 19624.53 | 368274 | 0.24463 |
| Itgax | 1.72E+07 | 70 | 758.6833 | 28522.53 | 547632 | 0.19296 |
| Itgb7 | 3.76E+10 | 82 | 764.7667 | 19208.59 | 544644 | 0.23517 |
| Cxcl10 | 1.23E+09 | 46 | 696.8 | 17828.09 | 318998 | 0.33816 |
| F9 | 9.63E+11 | 60 | 719.0333 | 10693.28 | 242204 | 0.32825 |
| Tmprss6 | 9.23E+11 | 43 | 676.1 | 13274.02 | 197394 | 0.299 |
| Ace2 | 3716353 | 34 | 695.65 | 12179.64 | 197896 | 0.18895 |
| Agt | 1.20E+13 | 85 | 784.2333 | 25107.07 | 595478 | 0.21961 |
| Mme | 1609176 | 37 | 705.9833 | 6988.343 | 133686 | 0.26577 |
| Edn1 | 1.83E+13 | 75 | 763.4 | 18790.02 | 405000 | 0.27027 |
| Slc15a1 | 622 | 26 | 602.2929 | 4223.695 | 63426 | 0.17538 |
| Ctgf | 2.07E+13 | 89 | 778.1333 | 26500.1 | 593964 | 0.28652 |
| Col8a1 | 1.40E+12 | 33 | 658.2833 | 6385.022 | 104450 | 0.33902 |
| Eln | 8.99E+12 | 65 | 743.8667 | 6556.396 | 213418 | 0.38413 |
| Nox4 | 1.39E+10 | 32 | 706.9667 | 8199.356 | 200152 | 0.37097 |
| Adm | 45498 | 17 | 631.0667 | 6472.406 | 117620 | 0.33824 |
| Gpx8 | 1.33E+11 | 53 | 718.9 | 8139.717 | 296052 | 0.31422 |
| Gpx7 | 1.33E+11 | 52 | 715.5667 | 7699.683 | 285984 | 0.32051 |
| Pde5a | 357 | 19 | 643.2 | 3865.239 | 57802 | 0.22222 |
| Spp1 | 2.35E+13 | 91 | 794.3833 | 20388.43 | 544036 | 0.28767 |
| Akr1b1 | 53054 | 28 | 662.4333 | 4372.294 | 101574 | 0.30423 |
| Myoz3 | 86 | 17 | 651.1 | 9032.996 | 105316 | 0.15441 |
| Cd34 | 2.24E+12 | 70 | 778.9667 | 8866.144 | 271240 | 0.33623 |
| Ccl2 | 2.45E+13 | 104 | 782.0167 | 29558.13 | 728052 | 0.22629 |
| Cav1 | 1.95E+10 | 75 | 781.4 | 44768.83 | 797050 | 0.18378 |
| Trpc6 | 476 | 22 | 657.4333 | 8256.448 | 128202 | 0.25108 |
| Egfr | 1.89E+13 | 173 | 886.4667 | 183379.2 | 3129330 | 0.10384 |
| Igf1 | 2.48E+13 | 110 | 813.0167 | 32964.53 | 790486 | 0.22185 |
| Lrrk2 | 288 | 44 | 741.0167 | 34193.11 | 473332 | 0.08245 |
| Ptgs2 | 5.38E+13 | 86 | 765.85 | 33786.38 | 893774 | 0.24104 |
| Slc15a2 | 170 | 23 | 593.6429 | 4047.91 | 64608 | 0.16206 |
| Ncf1 | 9.62E+08 | 33 | 702.1833 | 16070.84 | 301752 | 0.27652 |
| Gja1 | 7.00E+10 | 57 | 761.4333 | 25842.39 | 447712 | 0.2005 |
| Ace | 2.47E+11 | 49 | 731.55 | 8882.812 | 207960 | 0.32058 |
| Ech1 | 7.99E+07 | 26 | 618.1333 | 1976.251 | 31954 | 0.46769 |
| Hadh | 42088 | 32 | 649.3667 | 8891.542 | 152472 | 0.23387 |
| Acsl1 | 50240 | 49 | 694.3167 | 23448.22 | 393800 | 0.18622 |
| Slc27a5 | 23374 | 38 | 686.15 | 11593.05 | 193078 | 0.21195 |
| Etfb | 30347 | 18 | 603.3333 | 4018.598 | 62558 | 0.3268 |
| Hsd17b10 | 10090 | 17 | 604.6333 | 7499.414 | 104490 | 0.22059 |
| Hmgcs2 | 606860 | 56 | 712.85 | 16943.31 | 346580 | 0.21883 |
| Lpl | 7.79E+09 | 61 | 728.2167 | 21574.14 | 466484 | 0.24208 |
| Fabp4 | 57897 | 35 | 699.3667 | 7914.959 | 190542 | 0.28235 |
| Fabp5 | 36009 | 22 | 633.05 | 2147.483 | 38314 | 0.35931 |
| Pck1 | 35928 | 47 | 697.1667 | 9950.265 | 202158 | 0.22017 |
| Bcat1 | 492 | 28 | 636.45 | 4584.22 | 67166 | 0.19312 |
| Pecr | 7.99E+07 | 23 | 629.9 | 3510.276 | 54918 | 0.47826 |
| Oxct1 | 544 | 24 | 614.6333 | 6403.933 | 74854 | 0.21739 |
| Cdyl2 | 43458 | 19 | 598.3667 | 4114.39 | 55844 | 0.38596 |
| Acot4 | 3.99E+07 | 26 | 607.7333 | 2001.196 | 36066 | 0.37538 |
| Phyh | 7251 | 16 | 591.7167 | 4064.284 | 58860 | 0.43333 |
| Cyp4a1 | 9.22E+13 | 57 | 655.0667 | 3699.085 | 117650 | 0.37907 |
| Lce | 7053 | 24 | 596.3 | 2417.464 | 77780 | 0.38406 |
| Elovl6 | 6093 | 23 | 594.7333 | 2298.036 | 75992 | 0.38735 |
| Dgat2 | 23326 | 34 | 621.1667 | 5312.546 | 85382 | 0.28877 |
| Fads1 | 6664 | 24 | 588.25 | 2076.233 | 31574 | 0.34783 |
| Cyp4a2 | 9.22E+13 | 60 | 658.8667 | 5060.871 | 144110 | 0.35311 |
| Pdk4 | 2786 | 21 | 655.9667 | 4187.795 | 80808 | 0.30952 |
| Cryl1 | 10921 | 32 | 630.45 | 9145.347 | 128732 | 0.12298 |
| Eci1 | 53023 | 34 | 660.25 | 6638.784 | 109398 | 0.29412 |
| Acox2 | 7.99E+07 | 41 | 654.4667 | 3439.802 | 79350 | 0.33659 |
| Adhfe1 | 2402 | 56 | 673 | 25216.07 | 306356 | 0.1 |
| Fabp1 | 5.41E+08 | 60 | 716.15 | 18712.39 | 295364 | 0.25819 |
| Ehhadh | 7.99E+07 | 57 | 694.4 | 17964.96 | 286814 | 0.2099 |
| Hpd | 565 | 31 | 682.6667 | 7241.823 | 124196 | 0.15914 |
| Acadm | 93658 | 40 | 645.8167 | 5159.453 | 101700 | 0.29487 |
| Emp3 | 279 | 16 | 609.2333 | 4091.973 | 59382 | 0.19167 |
| Proc | 3.79E+11 | 47 | 714.4167 | 11729.2 | 199384 | 0.2803 |
| Cma1 | 1232494 | 25 | 666.4833 | 3638.335 | 85066 | 0.40333 |
| Mmp2 | 7.20E+12 | 88 | 779.75 | 13226.01 | 438898 | 0.29807 |
| Hamp | 280 | 13 | 628.9667 | 3320.592 | 52564 | 0.33333 |
| Mmp9 | 1.77E+13 | 119 | 823.8667 | 35810.69 | 968038 | 0.22888 |
| Apoe | 4.89E+11 | 88 | 786.9 | 44479.92 | 833882 | 0.19462 |
| Plat | 2.09E+09 | 38 | 704.1667 | 2695.157 | 72920 | 0.37696 |
| Serpind1 | 2.19E+10 | 44 | 689.35 | 5482.696 | 104160 | 0.41121 |
| F12 | 9.33E+11 | 60 | 736.7167 | 19295.21 | 389784 | 0.30113 |
| Lcat | 7.23E+09 | 30 | 677.9667 | 10003.89 | 157232 | 0.36552 |
| F11 | 3.91E+11 | 43 | 701.55 | 3183.671 | 88006 | 0.47619 |
| Hrg | 9.73E+11 | 70 | 733.2 | 13864.86 | 296272 | 0.30435 |
| Vtn | 4.88E+10 | 81 | 765.1667 | 21180.43 | 524642 | 0.22994 |
| Entpd1 | 1292 | 30 | 696.9167 | 13194.42 | 224974 | 0.21149 |
| Egf | 2.48E+13 | 150 | 852.4167 | 74374.86 | 1599972 | 0.15848 |
| F10 | 6.56E+07 | 35 | 690.4833 | 6380.189 | 129742 | 0.3395 |
| Vwf | 5.80E+12 | 84 | 773.0333 | 25631.43 | 523690 | 0.24125 |
| Mt1m | 5053 | 14 | 630.95 | 6781.19 | 90224 | 0.28571 |
| Itih1 | 1.63E+07 | 22 | 654.7667 | 4594.188 | 62916 | 0.45887 |
| Klkb1 | 5.82E+11 | 44 | 700.35 | 4603.626 | 123398 | 0.44609 |
| Lcn2 | 1.26E+08 | 33 | 692.6333 | 5423.72 | 115932 | 0.34848 |
| F3 | 9.07E+07 | 39 | 703.75 | 3341.484 | 87464 | 0.38596 |
| Mmp7 | 7.89E+12 | 42 | 717.5833 | 3688.393 | 89788 | 0.4216 |
| Serpina3c | 8.60E+08 | 36 | 678.9667 | 2828.799 | 60936 | 0.47302 |
| Timp1 | 2.40E+13 | 84 | 764.3333 | 20474.41 | 471680 | 0.29375 |
| Entpd8 | 776 | 24 | 640.0167 | 5403.574 | 81902 | 0.20652 |
| Apoh | 5.28E+09 | 55 | 714.5167 | 11505.1 | 203186 | 0.33737 |
| Apoa2 | 8.15E+09 | 60 | 726.9333 | 12458.87 | 270546 | 0.28814 |
| Timp2 | 3.91E+09 | 39 | 696.5833 | 2453.761 | 74206 | 0.44669 |
| Alb | 2.49E+13 | 237 | 938.35 | 331720.7 | 6446020 | 0.0969 |
| Serpinc1 | 9.73E+11 | 68 | 744.1667 | 18439.01 | 344890 | 0.29543 |
| Tgfb2 | 5.27E+07 | 32 | 685.65 | 8783.086 | 179380 | 0.37097 |
| Serpine1 | 2.25E+13 | 87 | 781.4833 | 15314.55 | 442078 | 0.27747 |
| Crp | 3.11E+10 | 60 | 730.95 | 12678.8 | 254192 | 0.25141 |
| Gab2 | 288 | 20 | 676.9833 | 6302.21 | 93296 | 0.18421 |
| Ttr | 1.96E+11 | 51 | 715.3667 | 14175.81 | 230038 | 0.28941 |
| Apoa4 | 9.52E+11 | 67 | 726.5333 | 13322.53 | 258894 | 0.31886 |
| A2m | 3.44E+08 | 47 | 704.0667 | 5067.375 | 119656 | 0.3062 |
| Ambp | 9.67E+09 | 48 | 708.1167 | 7453.056 | 138968 | 0.36348 |
| Cyp2c12 | 9.22E+13 | 53 | 649.6833 | 3381.482 | 102740 | 0.42525 |
| Itih3 | 5.15E+11 | 40 | 686.8333 | 5008.559 | 84244 | 0.46667 |
| Gc | 9.72E+11 | 62 | 738.8833 | 15007.87 | 301692 | 0.3284 |
| Azgp1 | 8.60E+07 | 28 | 661.5 | 8824.381 | 118824 | 0.48148 |

**Table S4 Functional enrichment analysis of PZH putative targets based on KEGG pathway**

| **Category** | **Term** |  | **Count** | **%** | **PValue** | **Benjamini** | **FDR** |
| --- | --- | --- | --- | --- | --- | --- | --- |
| KEGG_PATHWAY | rno05200: | Pathways in cancer | 65 | 10.25237 | 1.95E-11 | 1.46E-09 | 1.17E-09 |
| KEGG_PATHWAY | rno05225: | Hepatocellular carcinoma | 17 | 2.681388 | 0.015097 | 0.070529 | 0.056612 |
| KEGG_PATHWAY | rno05204: | Chemical carcinogenesis - DNA adducts | 16 | 2.523659 | 3.40E-06 | 6.77E-05 | 5.43E-05 |
| KEGG_PATHWAY | rno05207: | Chemical carcinogenesis - receptor activation | 19 | 2.996845 | 0.031852 | 0.129622 | 0.104045 |
| KEGG_PATHWAY | rno04115: | p53 signaling pathway | 10 | 1.577287 | 0.00925 | 0.051217 | 0.04111 |
| KEGG_PATHWAY | rno01521: | EGFR tyrosine kinase inhibitor resistance | 9 | 1.419558 | 0.042471 | 0.160745 | 0.129026 |
| KEGG_PATHWAY | rno01522: | Endocrine resistance | 15 | 2.365931 | 1.87E-04 | 0.001996 | 0.001602 |
| KEGG_PATHWAY | rno01100: | Metabolic pathways | 127 | 20.03155 | 1.10E-08 | 4.69E-07 | 3.76E-07 |
| KEGG_PATHWAY | rno05205: | Proteoglycans in cancer | 35 | 5.520505 | 3.97E-10 | 1.98E-08 | 1.59E-08 |
| KEGG_PATHWAY | rno00010: | Glycolysis / Gluconeogenesis | 8 | 1.26183 | 0.048944 | 0.180671 | 0.14502 |
| KEGG_PATHWAY | rno00982: | Drug metabolism - cytochrome P450 | 9 | 1.419558 | 0.02643 | 0.114531 | 0.091931 |
| KEGG_PATHWAY | rno05230: | Central carbon metabolism in cancer | 10 | 1.577287 | 0.006415 | 0.039147 | 0.031422 |
| KEGG_PATHWAY | rno00980: | Metabolism of xenobiotics by cytochrome P450 | 11 | 1.735016 | 0.003899 | 0.025909 | 0.020797 |
| KEGG_PATHWAY | rno04974: | Protein digestion and absorption | 22 | 3.470032 | 2.06E-08 | 7.69E-07 | 6.17E-07 |
| KEGG_PATHWAY | rno01200: | Carbon metabolism | 14 | 2.208202 | 0.005592 | 0.034836 | 0.027962 |
| KEGG_PATHWAY | rno00650: | Butanoate metabolism | 6 | 0.946372 | 0.010865 | 0.056629 | 0.045455 |
| KEGG_PATHWAY | rno04062: | Chemokine signaling pathway | 17 | 2.681388 | 0.017435 | 0.080199 | 0.064374 |
| KEGG_PATHWAY | rno04514: | Cell adhesion molecules | 17 | 2.681388 | 0.010586 | 0.056524 | 0.04537 |
| KEGG_PATHWAY | rno04750: | Inflammatory mediator regulation of TRP channels | 18 | 2.839117 | 4.33E-05 | 5.62E-04 | 4.51E-04 |
| KEGG_PATHWAY | rno04151: | PI3K-Akt signaling pathway | 48 | 7.570978 | 1.30E-10 | 7.79E-09 | 6.25E-09 |
| KEGG_PATHWAY | rno04512: | ECM-receptor interaction | 26 | 4.100946 | 2.63E-13 | 7.86E-11 | 6.31E-11 |
| KEGG_PATHWAY | rno04659: | Th17 cell differentiation | 12 | 1.892744 | 0.013082 | 0.064124 | 0.051471 |
| KEGG_PATHWAY | rno04610: | Complement and coagulation cascades | 24 | 3.785489 | 1.26E-11 | 1.25E-09 | 1.01E-09 |
| KEGG_PATHWAY | rno04066: | HIF-1 signaling pathway | 12 | 1.892744 | 0.024432 | 0.107429 | 0.08623 |
| KEGG_PATHWAY | rno04658: | Th1 and Th2 cell differentiation | 12 | 1.892744 | 0.00413 | 0.026844 | 0.021547 |
| KEGG_PATHWAY | rno04670: | Leukocyte transendothelial migration | 13 | 2.050473 | 0.012336 | 0.062517 | 0.050181 |
| KEGG_PATHWAY | rno04110: | Cell cycle | 24 | 3.785489 | 4.58E-08 | 1.37E-06 | 1.10E-06 |
| KEGG_PATHWAY | rno04510: | Focal adhesion | 37 | 5.835962 | 7.70E-12 | 1.15E-09 | 9.24E-10 |
| KEGG_PATHWAY | rno04014: | Ras signaling pathway | 19 | 2.996845 | 0.03545 | 0.139467 | 0.111946 |
| KEGG_PATHWAY | rno04015: | Rap1 signaling pathway | 18 | 2.839117 | 0.03356 | 0.133792 | 0.107392 |
| KEGG_PATHWAY | rno03320: | PPAR signaling pathway | 20 | 3.154574 | 2.47E-08 | 8.22E-07 | 6.60E-07 |
| KEGG_PATHWAY | rno04540: | Gap junction | 14 | 2.208202 | 3.05E-04 | 0.003144 | 0.002524 |
| KEGG_PATHWAY | rno04726: | Serotonergic synapse | 14 | 2.208202 | 0.007367 | 0.043188 | 0.034666 |
| KEGG_PATHWAY | rno01230: | Biosynthesis of amino acids | 15 | 2.365931 | 2.26E-05 | 3.55E-04 | 2.85E-04 |
| KEGG_PATHWAY | rno00380: | Tryptophan metabolism | 10 | 1.577287 | 6.47E-04 | 0.005861 | 0.004704 |
| KEGG_PATHWAY | rno00350: | Tyrosine metabolism | 6 | 0.946372 | 0.037212 | 0.1445 | 0.115987 |
| KEGG_PATHWAY | rno00360: | Phenylalanine metabolism | 5 | 0.788644 | 0.010224 | 0.055582 | 0.044614 |
| KEGG_PATHWAY | rno00260: | Glycine, serine and threonine metabolism | 9 | 1.419558 | 5.17E-04 | 0.004971 | 0.00399 |
| KEGG_PATHWAY | rno00480: | Glutathione metabolism | 10 | 1.577287 | 0.007734 | 0.04447 | 0.035695 |
| KEGG_PATHWAY | rno00270: | Cysteine and methionine metabolism | 11 | 1.735016 | 1.31E-04 | 0.001512 | 0.001213 |
| KEGG_PATHWAY | rno00591: | Linoleic acid metabolism | 12 | 1.892744 | 2.30E-06 | 4.91E-05 | 3.94E-05 |
| KEGG_PATHWAY | rno00590: | Arachidonic acid metabolism | 17 | 2.681388 | 1.88E-06 | 4.69E-05 | 3.76E-05 |
| KEGG_PATHWAY | rno00140: | Steroid hormone biosynthesis | 17 | 2.681388 | 2.23E-06 | 4.91E-05 | 3.94E-05 |
| KEGG_PATHWAY | rno04976: | Bile secretion | 17 | 2.681388 | 1.20E-05 | 1.99E-04 | 1.60E-04 |
| KEGG_PATHWAY | rno00071: | Fatty acid degradation | 7 | 1.104101 | 0.03208 | 0.129622 | 0.104045 |
| KEGG_PATHWAY | rno00120: | Primary bile acid biosynthesis | 6 | 0.946372 | 0.001421 | 0.010892 | 0.008743 |
| KEGG_PATHWAY | rno04979: | Cholesterol metabolism | 11 | 1.735016 | 1.10E-04 | 0.001314 | 0.001055 |
| KEGG_PATHWAY | rno04072: | Phospholipase D signaling pathway | 18 | 2.839117 | 0.001096 | 0.008856 | 0.007109 |
| KEGG_PATHWAY | rno03320: | PPAR signaling pathway | 20 | 3.154574 | 2.47E-08 | 8.22E-07 | 6.60E-07 |
| KEGG_PATHWAY | rno04146: | Peroxisome | 12 | 1.892744 | 0.003458 | 0.023498 | 0.018861 |
